# An Experimental Acidity Scale for Intramolecularly Stabilized Silyl Lewis Acids

**DOI:** 10.1002/chem.201903241

**Published:** 2019-10-31

**Authors:** Sandra Künzler, Saskia Rathjen, Anastasia Merk, Marc Schmidtmann, Thomas Müller

**Affiliations:** ^1^ Carl von Ossietzky Universität Oldenburg Carl von Ossietzky-Str. 9–11 26129 Oldenburg Germany, European Union

**Keywords:** chalcogens, Lewis acids, NMR spectroscopy, silicon, silyl cation

## Abstract

A new NMR‐based Lewis acidity scale is suggested and its application is demonstrated for a family of silyl Lewis acids. The reaction of *p*‐fluorobenzonitrile (FBN) with silyl cations that are internally stabilized by interaction with a remote chalcogenyl or halogen donor yields silylated nitrilium ions with the silicon atom in a trigonal bipyramidal coordination environment. The ^19^F NMR chemical shifts and the ^1^ *J*(CF) coupling constants of these nitrilium ions vary in a predictable manner with the donor capability of the stabilizing group. The spectroscopic parameters are suitable probes for scaling the acidity of Lewis acids. These new probes allow for the discrimination between very similar Lewis acids, which is not possible with conventional NMR tests, such as the well‐established Gutmann–Beckett method.

## Graphical abstract

**The basis of natural sciences** is to measure complex parameters, such as Lewis acidity. A new NMR‐based probe is suggested, which allows for the scaling of stabilized Lewis acids and complements the toolbox of methods for gauging Lewis acidity.

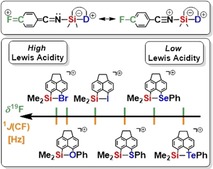

## Introduction

**Figure 1:**
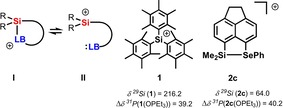
Intramolecularly stabilized silyl cation **I** and its non‐stabilized isomer **II**. Relevant NMR parameters of triaryl silylium ion **1**, of selenylether‐stabilized cation **2 c** and their complexes with OPEt_3_ [Δ*δ* ^31^P NMR of the OPEt_3_ complex relative to that of OPEt_3_ (*δ* ^31^P=46.2)].

Lewis acid (LA) catalysts are of widespread use in synthetic chemistry and catalysis.^1^ Consequently, there is a constant quest for new Lewis acids of particular strength and for Lewis acids with clearly defined acidity, which is adjusted to the synthetic challenge. Silyl Lewis acids are particularly interesting because they span a very broad spectrum of different acidity strengths, beginning with moderate LAs, such as trimethylsilyl chloride, to extremely strong examples, such as triarylsilylium ions or solvent complexes of trialkylsilylium ions.^2^ Recently, cationic silyl Lewis acids in particular have come into the focus of synthetic chemists due to their promising exceptional high Lewis acidity.2b, 3, 4 The requirement for their beneficial use in preparative work is however a clear control over their reactivity.^5^ In many cases, this was achieved by intramolecular coordination of weak Lewis bases (LB) to the cationic silicon center that results in tetra‐coordination for the silicon center (Figure 1). In saying that, the parallels to intramolecular Frustrated Lewis Pairs (FLPs) popularized by Erker and Stephan become obvious.1c, 1d, 3p, 6 The strength of the LA/LB interaction determines structure, spectroscopic properties and, in the context of this report most important, the Lewis acidity of these species. For synthetic purposes, a quantitative evaluation of the Lewis acidity is desirable and a clear ranking of similar Lewis acids is especially needed.^7^ Several experimental methods have been established that allow for the scaling of Lewis acidity. The most prominent ones are based on the change in NMR chemical shifts of a probe Lewis base upon coordination to a series of Lewis acids. The Gutmann–Beckett method uses the change of the ^31^P NMR chemical shift of the probe Lewis base OPEt_3_ upon complexation for scaling different Lewis acids.^8^ In Childs method the base is crotonaldehyde and the ^1^H NMR chemical shift of the γ‐proton is probed.^9^ Related to these methods, Hilt and co‐workers applied the ^2^H NMR chemical shifts of the γ‐deuterium of the Lewis acid complexes of perdeutero‐pyridine to gauge their acidity.^10^ Although Childs method could not be applied to cationic silyl Lewis acids, pyridine as well as phosphane oxides have been used to measure their Lewis acidity and these investigations revealed the high Lewis acidity of cationic silicon compounds.10c, 11 In addition, it was shown that these methods fail to gauge correctly the Lewis acidity of intramolecularly stabilized silyl cations **I**. In both cases, the interaction between the external probe base and the silyl Lewis acid cancels the intramolecular interaction between the stabilizing donor and the silyl group.10c, 11 Consequently, the authors did not report the Lewis acidity of the internally stabilized silyl Lewis acid **I** but, instead, that of a not relevant donor‐free species **II**. A comparison highlights this issue: triarylsilylium ion **1** 12 and silyl cation **2 c**,^13^ which is stabilized by the remote selenylether donor, are very different with respect to their electronic properties (as for example shown by their vastly different ^29^Si NMR chemical shift) and their reactivity. Nevertheless, the Gutmann–Beckett method assigns to both nearly the same Lewis acidity, expressed by almost identical Δ*δ* ^31^P values (Figure 1). Moreover, the selenyl‐stabilized silyl cation **2 c** is, according to the Gutmann–Beckett method, even slightly more Lewis acidic than silylium ion **1**. Clearly, the stabilization of the silyl cation **2 c** by the selenylether substituent is pushed back by the external base phosphine oxide and all information on the actual acidity of the stabilized cation **2 c** is lost. This example highlights the need for a less strong Lewis base as a probe, which would allow for the classification of subtle distinctions in the Lewis acidity of intramolecularly stabilized silyl cations. Here, we report our results by using *p*‐fluorobenzonitrile (FBN) as an NMR probe for the scaling of intramolecularly stabilized silyl Lewis acids.

## Results and Discussion

**Figure 2:**
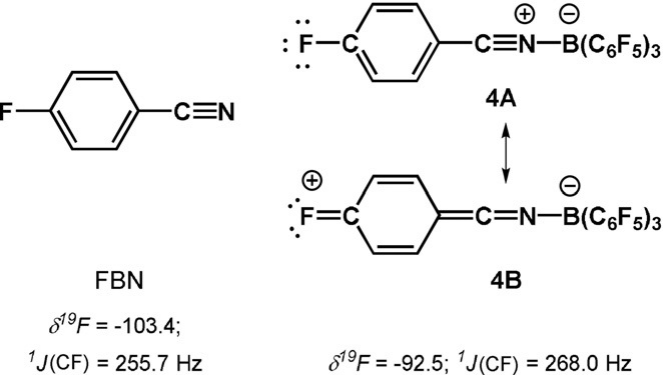
Selected NMR parameters of *p*‐fluorobenzonitrile (FBN) and of nitrilium ylide **4**.

**Figure 3:**
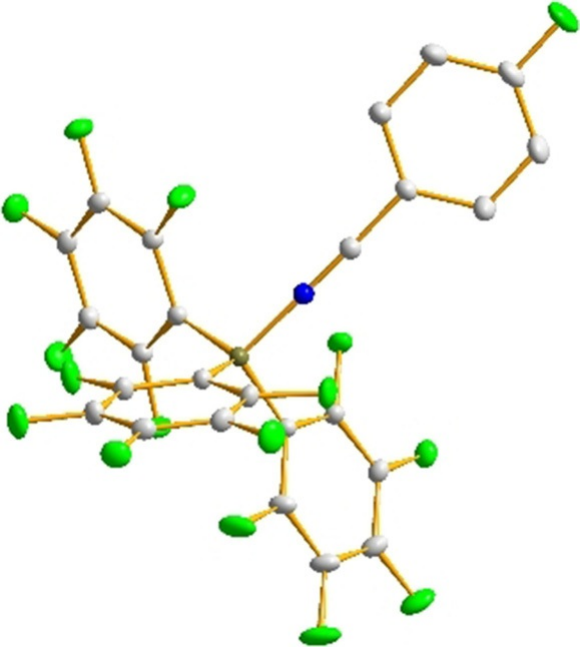
Molecular structure of complex **4** in the crystal. Pertinent bond lengths and angles: B−N 158.77(11) pm; C−N 114.56(10) pm; C−C^*ipso*^ 142.47(10) pm; B‐N‐C 179.732(77)°; Σ*α*(CBC)=340.0°. (Thermal ellipsoids drawn at 50 % probability, hydrogen atoms are omitted, color code: grey carbon; green fluorine; blue nitrogen, brown boron).

We found that FBN is only a weak donor towards typical Lewis acids. For example, it forms no Lewis acid–base complex with trityl cation **3**, [Ph_3_C]^+^, as suggested by the unchanged NMR parameters for both compounds when trityl *tetrakis*‐pentafluorophenyl borate **3**[B(C_6_F_5_)_4_] was mixed with FBN in methylene chloride (see Supporting Information). Stronger Lewis acids, such as *tris*‐pentafluorophenyl borane (BCF), however, form a stable complex with FBN upon mixing both compounds in methylene chloride. A relative sharp ^11^B NMR resonance at *δ* ^11^B=−10.0 (FWHH=275 Hz) for the complex **4** indicates tetracoordination for the boron atom (c.f.: [B(C_6_F_5_)_4_]^−^, w(1/2)=21 Hz). This is supported by the relative small separation of the ^19^F NMR signals for *p*‐ and *m*‐fluorine atoms of the C_6_F_5_‐groups in ylide **4** (**4**: Δ*δ* ^19^F^m/*p*^=7.2, c.f. [B(C_6_F_5_)_4_]^−^: Δ*δ* ^19^F^m/*p*^=3.9 and [B(C_6_F_5_)_3_]: Δ*δ* ^19^F^m/*p*^=20.1).^14^ The ^19^F NMR signal of the *p*‐fluorine atom is shifted upon formation of the complex **4** by Δ*δ* ^19^F=10.9 to lower field. The deshielding of the fluorine atom and the increased ^1^ *J*(CF) coupling constant indicates the importance of the quinoid resonance structure **4B** (Figure 2). Finally, formation of nitrilium ylide **4** was confirmed by X‐ray diffraction (XRD) analysis of suitable crystals. The molecular structure of ylide **4** reveals a tetra‐coordinated boron atom [Σ*α*(CBC)=340.0°] with a regular B−N bond (158.8 pm, sum of the predicted covalent radii: 156 pm)^15^ and the expected linear B‐N‐C unit (Figure 3).

Silylium ions are commonly accepted to be stronger Lewis acids than tricoordinated boron compounds.^11^ We prepared silylium ion **5** by the standard Corey protocol using [Ph_3_C][B(C_6_F_5_)_4_] in methylene chloride in the presence of exactly one equivalent of FBN which gave the silylated nitrilium borate **6**[B(C_6_F_5_)_4_].2c Nitrilium ion **6** is characterized by the ^29^Si NMR chemical shift of *δ* ^29^Si=23.0, in the typical spectral range for silylated nitrilium ions (*δ* ^29^Si=6–40).^16^ The stronger deshielding of the *p*‐fluorine atom (*δ* ^19^F=−86.6) and the large ^1^ *J*(CF) coupling constant [^1^ *J*(CF)=272.8 Hz] indicate a higher contribution of the quinoid resonance structure for the silylated nitrilium ion **6** compared to the nitrilium borate ylide **4** and suggests a higher Lewis acidity for silylium ion **5**.

**Scheme 1:**
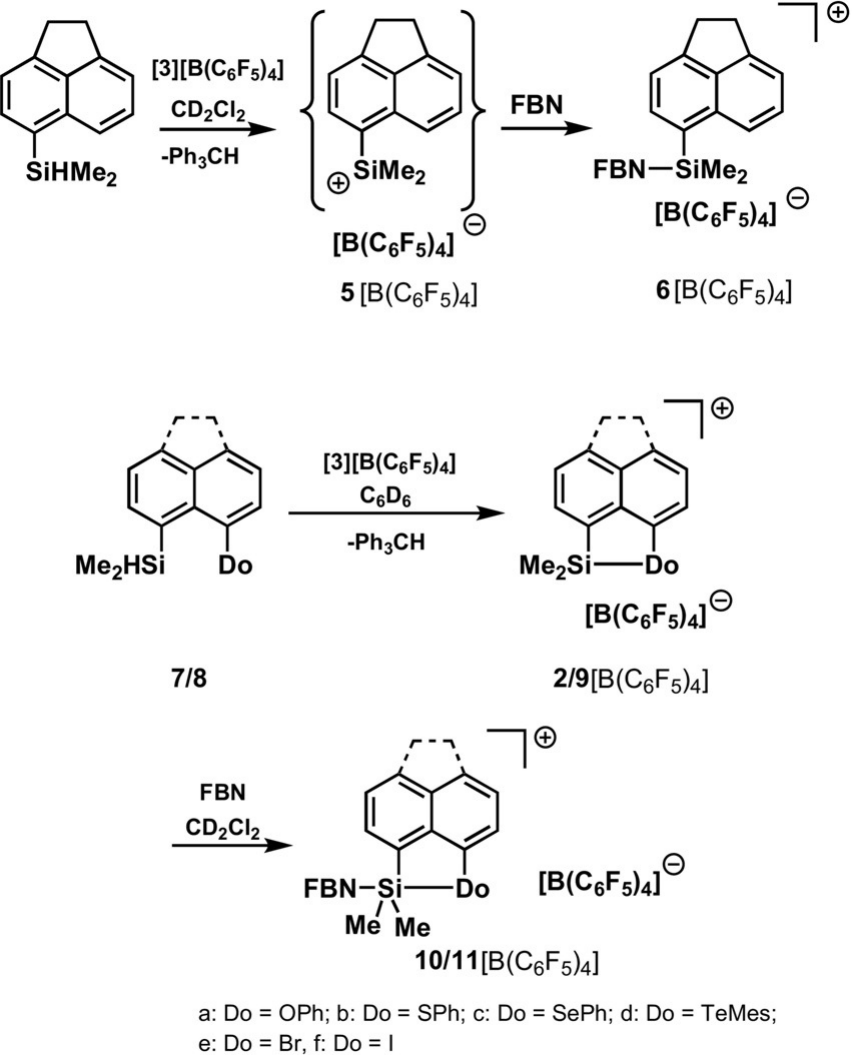
Formation of silyl cations **2**, **5** and **9** and subsequent transformation to nitrilium ions **6**, **10** and **11** (**2**, **5**, **7** and **10**: acenaphthene‐based compounds; **8**, **9**, **11**: naphthalene‐based compounds).

**Figure 4:**
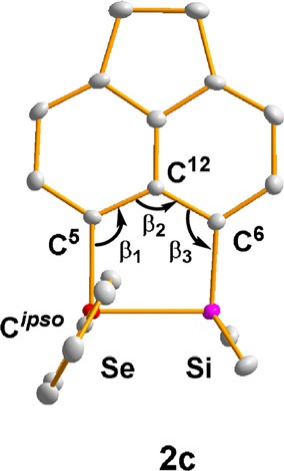
Molecular structure of cation **2 c** in the crystal of [**2 c**]_2_[B_12_Br_12_] *x* 8 [H_2_CCl_2_]. Pertinent bond lengths and angles: Se−Si 243.05(6) pm; Se−C^5^ 192.4(2) pm; Si−C^6^ 185.2(2) pm; *β*_1_ 115.37(16)°; *β*_2_ 125.7(2)°; *β*_3_ 114.85(16)°; C^5^‐Se‐C^ipso^ 101.19(9)°; C^6^‐C^1^‐Se‐Si −7.941(67)°; Σ*α*(SiC_3_) 346.06(3)°; Σ*α*(Se)=288.41(2)°; Σ*β*=355.9(4)°. (Thermal ellipsoids drawn at 50 % probability, hydrogen atoms are omitted, color code: grey carbon; violet silicon; red selenium).

Next, we tested the reactions of FBN with a series of silyl cations stabilized by interaction with chalcogenyl and halogen substituents based on the acenaphthene (**2**) and naphthalene (**9**) backbone. Recently, sulfur‐ and oxygen‐stabilized silyl cations have attracted special interest as possible Lewis acidic catalysts.^4^, ^13^ The selenyl‐stabilized cation **2 c** was chosen as a test case, considering that the cationic silicon atom and the selenium‐based donor group can be easily monitored by NMR spectroscopy. Silyl borate **2 c**[B(C_6_F_5_)_4_] was prepared according to Scheme 1 by using the standard Corey reaction^17^ and was fully characterized by multinuclear NMR spectroscopy. Its identity was verified by comparison with literature data.^13^ Finally, the molecular structure of cation **2 c** was unequivocally established by an XRD analysis of the salt [**2 c**]_2_[B_12_Br_12_]. The molecular structure of the acenaphthene‐based cation **2 c** closely resembles that of the previously reported naphthalene‐based cation **9 c** (Figure 4).^13^ The silicon atom in **2 c** is tetracoordinated with a significant trigonal flattening of the tetrahedral coordination of the silicon atom [Σ*α*(SiC_3_)=346.1°]. The coordination environment of the selenium atom is trigonal pyramidal [Σ*α*(Se)=288.4°]. As expected, the ethylidene bridge in cation **2 c** elongates the silicon selenium bond slightly [243.05(6) pm (**2 c**) vs. 240.6 pm (**9 c**)]. The attractive Lewis acid–base interaction between the two atoms in *peri*‐position of the acenaphthene moiety does not lead to significant strain in cation **2 c**.^18^ This is indicated by the sum of the bay angles Σβ of 355.9° that is close to the ideal value of 368° for the unsubstituted acenaphthene (see Figure 4).^19^ The silicon atom is placed 39 pm above the plane spanned by the ten carbon atoms of the naphthalene subunit, while the selenium atom is essentially placed in this plane.

**Figure 5:**
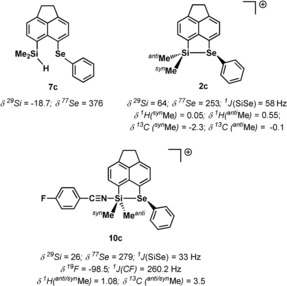
Selected NMR parameters of silylselenide **7 c** and cations **2 c** and **10 c**.

Nitrilium ion **10 c** was synthesized by addition of exactly an equimolar amount of FBN to a solution of **2 c**[B(C_6_F_5_)_4_] in methylene dichloride (Scheme 1). Its formation is indicated by a significant high‐field shift of the ^29^Si NMR resonance (Δ*δ* ^29^Si=−38) and by a deshielding of the selenium atom (Δ*δ* ^77^Se=26) compared to cation **2 c** (Figure 5). Interestingly, the ^77^Se NMR chemical shift of nitrilium ion **10 c** is still markedly smaller than reported for dicoordinated bisarylselenides such as selenide **7 c** and suggests for the selenium atom a trigonal pyramidal coordination environment. In agreement, the substantial ^1^ *J*(SiSe) coupling constant of 33 Hz points to the presence of a Si−Se bond.^20^ The coordination of the nitrile to the silicon atom is shown by the low field shift of the ^19^F NMR signal and an increase of the ^1^ *J*(CF) coupling constant [Δ*δ* ^19^F=4.9, Δ^1^ *J*(CF)=4.5 Hz]. Upon formation of the nitrilium ion **10 c** from selenonium ion **2 c** the diasterotopic methyl groups, ^*syn/anti*^Me, at the silicon atom become magnetically equivalent at room temperature in both ^1^H and ^13^C NMR spectra (Figure 5). This indicates a lower barrier for the inversion of the trigonal pyramidal configuration at the selenium atom.^13^ All attempts to obtain definite structural information for the pentacoordination of the silicon atom in nitrilium ion **10 c** by growing suitable single crystals for XRD analysis from salts of weakly coordinating anions, such as perfluorinated tetraarylborates or brominated dodecahedral borates, failed.^21^ Either slow decomposition occurred or crystals of the corresponding silyl cation **2 c** salts were obtained. For example, the salt [**2 c**]_2_[B_12_Br_12_] crystallized from a methylene chloride/hexane solution of nitrilium *closo*‐borate [**10 c**]_2_[B_12_Br_12_].

**Figure 6:**
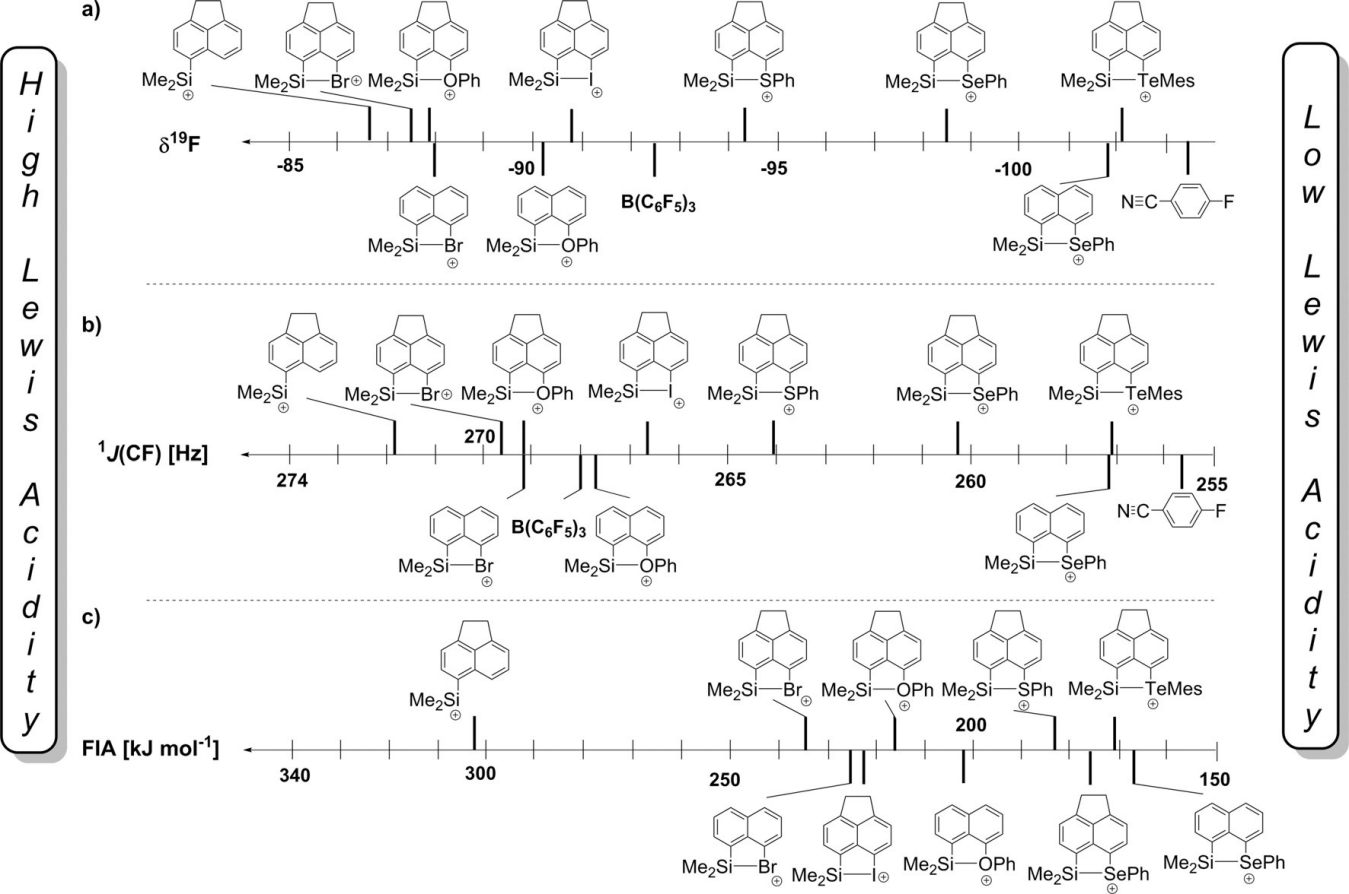
Different scales for the Lewis acidity for cations **2** and **9** and related compounds. a) Scale based on ^19^F NMR chemical shifts of nitrilium ions **10** and **11**. b) Scale based on ^1^ *J*(CF) coupling constants of nitrilium ions **10** and **11**. c) Scale based on fluoride ion affinities (FIA) calculated for cations **2** and **9** at SCIPM/M06‐2X/Def2‐TZVP for methylene chloride solution by using reaction (1) in Scheme 2.

**Figure 7:**
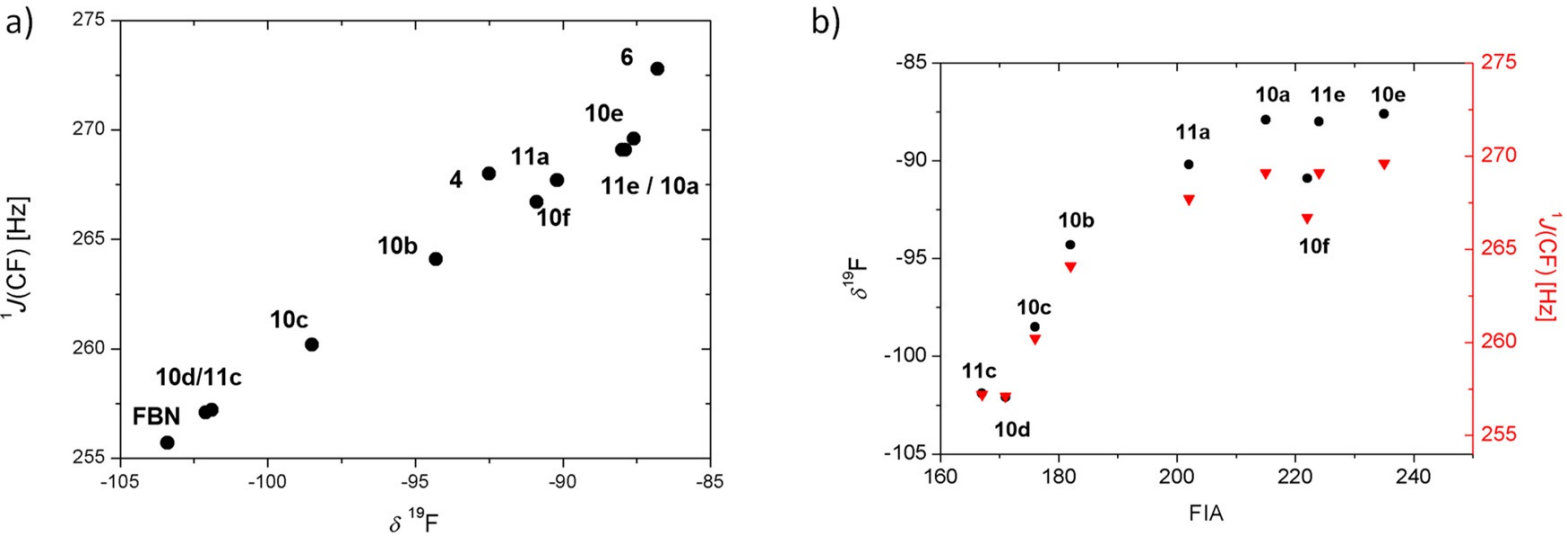
a) Plot of the ^1^ *J*(CF) coupling constants versus the ^19^F NMR chemical shifts of nitrilium ions **6**, **10**, **11**, and ylide **4**. A line fit using all eleven data points results in the following statistics: ^1^ *J*(CF)=(0.91±0.05) Hz × *δ* ^19^F + (350.27±4.62) Hz; *R* ^2^=0.97. b) Plot of *δ* ^19^F (black circles, left axis) and ^1^ *J*(CF) (red triangles, right axis) data for nitrilium ions **10** and **11** versus FIA calculated for the corresponding cations **2** and **9** by using reaction (1) in Scheme 2.

In summary, the NMR spectroscopic data for nitrilium ion **10 c** clearly indicate pentacoordination for the silicon atom and a significant bonding interaction between the silicon and selenium atoms, although the nitrile is coordinated to the silicon atom. The fact that nitrile coordination does not cancel the silicon–selenium interaction suggested to us that the spectroscopic data of nitrilium ion **10 c**, such as ^19^F NMR chemical shift and ^1^ *J*(CF) coupling constant, reflect the Lewis acidity of the stabilized silyl cation. The modification of these two parameters clearly show the attenuation of the acidity of the silyl Lewis acid **2 c** by the selenium donor compared to silyl cation **5**. To test if both NMR spectroscopic parameters are general measures of the acidity of intramolecularly stabilized Lewis acids, we analyzed the NMR spectroscopic data obtained for the series of nitrilium borates **10**[B(C_6_F_5_)_4_] and **11**[B(C_6_F_5_)_4_] (Scheme 1). All investigated nitrilium borates **10**[B(C_6_F_5_)_4_] and **11**[B(C_6_F_5_)_4_] were fully characterized by multinuclear NMR spectroscopy and the complete data are summarized in the Supporting Information. Results that are pertinent for the discussion are shown in Table 1. The ^19^F{^1^H} NMR spectra were collected at room temperature in CD_2_Cl_2_, and were calibrated against the signal of the *p*‐fluorine atoms of the [B(C_6_F_5_)_4_]^−^ anion (*δ* ^19^F=−163.44), which was referenced against fluorobenzene [*δ* ^19^F (C_6_H_5_F)=−113.78].^22^ In this series of intramolecularly stabilized silyl cations, the two NMR parameters varied from *δ* ^19^F=−102.1 and ^1^ *J*(CF)=257.1 Hz for the telluryl‐substituted nitrilium ion **10 d** to *δ* ^19^F=−86.6 and ^1^ *J*(CF)=272.8 Hz for the unsubstituted silyl cation **6** (Figure 6 a,b). The values for the tellurium compound **10 d** are close to those measured for the free FBN, which indicates for cation **2 d** the strongest interaction between the donor group and the silicon center. For the other extreme, the bromo‐substituted species **10 e**, the parameters are close to those for cation **5**. Hence, bromo‐substituted silyl cation **2 e** is the strongest Lewis acid in this series, whereas the tellurium compound **2 d** is the weakest. As expected, we noticed a clear linear correlation between both NMR parameters, which indicates that both values can be used as measure for the Lewis acidity of intramolecularly stabilized silyl cations (Figure 7). Consequently, both scales predict the same order of Lewis acidity for the tested silyl Lewis acids. We note, however, that the relative position of BCF varies somewhat on both scales (see Figure 6 a,b). In addition, we note that both scales indicate a larger dispersion of the data for weak Lewis acids and a smaller separation for stronger Lewis acids (Figure 6 a,b). Clearly, this confers to the sequence of strong Lewis acids a larger degree of uncertainty. Nevertheless, when acenaphthene‐based silyl Lewis acids **2** are compared to the corresponding naphthalene systems **9**, the Lewis acidity is always larger for the acenaphthene‐based cation. For example, for the donor OPh, cation **2 a** is more Lewis acidic than **9 a**. This is in line with the weaker Si–Do (Do=donor) interaction previously found for acenaphthene‐based cations **2** compared to the naphthalene systems **9** with the same donor.^13^

**Table 1:** Experimental NMR parameters of nitrilium ions **10**, **11** and related compounds (in CD_2_Cl_2_ at 305 K) and calculated bond energies (BDE) and fluoride ion affinity (FIA) of the corresponding silyl cation **2**, **5**, **9** and BCF (at M06‐2X/Def2‐TZVP, FIA at SCIPCM M06‐2X/Def2‐TZVP). Cpd=compound.

Cpd	Do	*δ* ^19^F	^1^ *J*(CF) [Hz]	^1^ *J*(SiDo) [Hz]	FIA [kJ mol^−1^]	BDE (Si−N) [kJ mol^−1^]
**10 a**	OPh	−87.9	269.1	–	215	96
**10 b**	SPh	−94.3	264.1	–	182	70
**10 c**	SePh	−98.5	260.2	33	176	65
**10 d**	TeMes	−102.1	257.1	171	171	51
**10 e**	Br	−87.6^[b]^	269.7	–	235	119
**10 f**	I	−90.9^[b]^	266.7	–	222	105
**11 a**	OPh	−90.2^[a]^	267.7^[a]}^	–	201	90
**11 c**	SePh	−101.9	257.2	56	167	62
**11 e**	Br	−88.0^[b]^	269.1	–	224	112
**4**	–	−92.5	268.0	–	–	–
**6**	–	−86.6	272.8	–	302	183
**FBN**	–	−103.4	255.7	–	–	–

[a] At 233 K in CD_2_Cl_2_vs. reference at 233 K *δ* ^19^F=−162.87, see Supporting Information. [b] Broad signals: [FWHH=299 Hz (**10 e**); 347 Hz (**10 f**); 109 Hz (**11 e**)].

**Figure 8:**
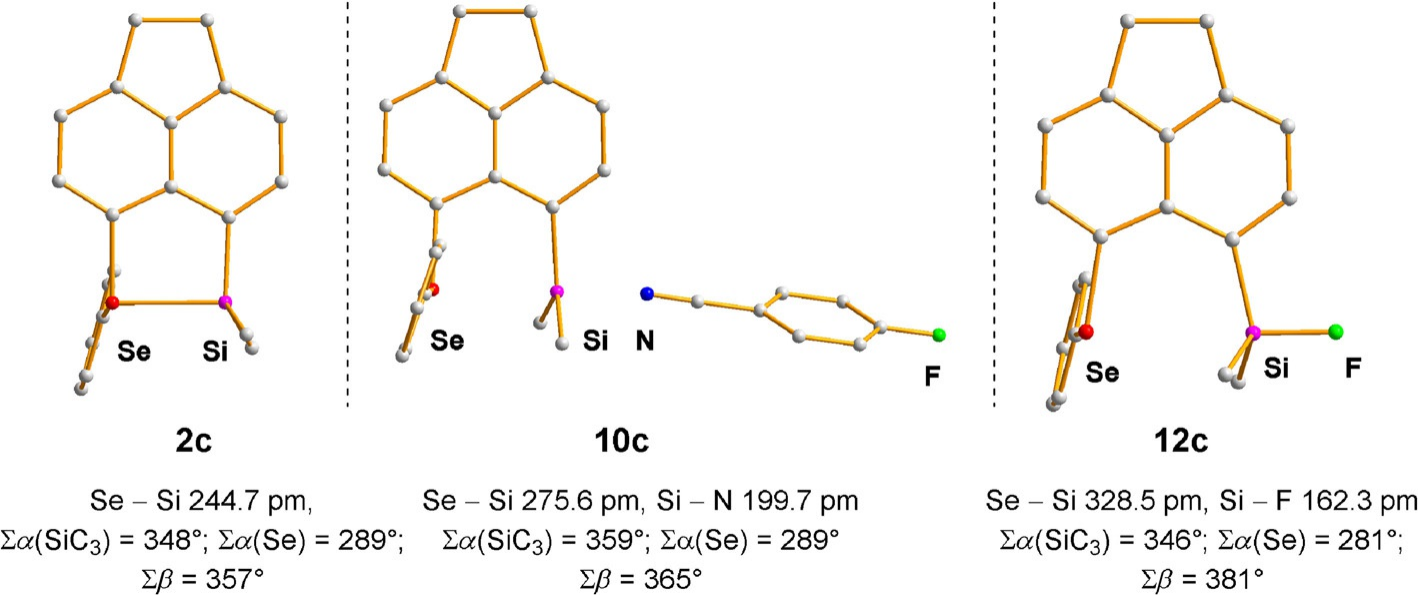
Calculated molecular structures of selenonium cation **2 c**, nitrilium ion **10 c** and silyl fluoride **12 c** (at M06‐2X/def2‐TZVP, all hydrogen atoms are omitted, color code: grey carbon; violet silicon; red selenium, blue nitrogen, green fluorine).

**Scheme 2:**
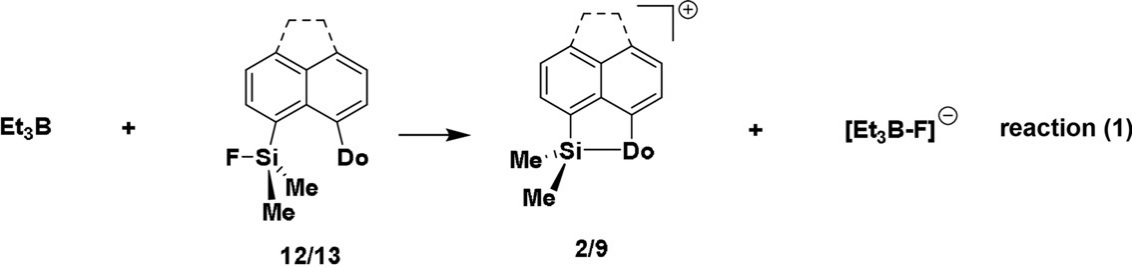
Reaction (1) used for the evaluation of the fluoride ion affinity.

Quantum mechanical calculations at the M06‐2X/def2‐TZVP level of theory were used to gain further insights. This computational model is justified by the close agreement between the theoretically predicted molecular structure of cation **2 c** and that determined experimentally by XRD. Optimized structural parameters that are pertinent to the discussion, such as bond lengths and bond angles in the bay region of cation **2 c**, differ by less than 1 % and provide a solid basis for the structural discussion. The calculated structural data for all investigated nitrilium ions are summarized in Table 2. As a typical example, Figure 8 compares the calculated molecular structure of cation **2 c** with that of nitrilium ion **10 c**. In nitrilium ion **10 c** the silicon atom adopts a trigonal bipyramidal coordination sphere with the selenyl group and the nitrile at the axial positions and an almost planar trigonal basis spanned by the silicon atom and its three carbon substituents [Σα(SiC_3_)=359°]. The Si–Se distance is 275.6 pm, elongated by 30.9 pm compared to selenonium ion **2 c**,^13^ and the coordination of the nitrile is indicated by the short Si−N distance of 199.7 pm. Both values are significantly smaller than the respective sum of the van der Waals radii [ΣvdW; 400 pm (SiSe) and 365 pm (SiN)].^23^ The sum of the bay angles (Σβ) taken as a measure for strain induced by the *peri* substitution indicates no significant hindrance in cation **2 c** and in nitrilium ion **10 c** [Σ*β*=357° (**2 c**) and 365° (**10 c**)].^18^ Very similar structural parameters are computed for all nitrilium ions **10** and **11** and are summarized in Table 2. A first idea about the Lewis acidity of the stabilized silyl cations is provided by the complexation energy between the cation and the coordinating nitrile, which is the bond dissociation energy of the Si−N(FBN) bond, BDE (Si−N) (Table 1). FBN is weakly bonded to the chalcogenyl‐substituted cations **2 a**–**d**, **9 a** [BDE (Si−N)=51–96 kJ mol^−1^] and only slightly stronger to the bromo‐ and iodo‐stabilized cations **2 e**, **f** and **9 e** [BDE (Si−N)=105–119 kJ mol^−1^]. Based on these calculated cation–nitrile interaction energies, the strongest Lewis acid among our test set is the bromo‐stabilized silyl cation **2 e** and the weakest is the telluryl‐stabilized **2 d**, in qualitative agreement with the scales based on ^19^F NMR chemical shifts and the ^1^ *J*(CF) coupling constants (Figure 6 a,b). A commonly accepted theoretical scale for Lewis acidity is based on calculated fluoride ion affinities.^7^, ^24^ We calculated the fluorine ion affinity (FIA) for cations **2** and **9** versus BEt_3_ at the M06‐2X/Def2‐TZVP level of theory with inclusion of solvent effects using the SCIPM model with methylene chloride as solvent according to reaction (1) (Scheme 2). The results are summarized in Table 1 and are graphically displayed in Figure 6 c. The FIAs for the stabilized silyl cations **2**, **9** are clearly separated from that predicted for the non‐stabilized cation **5** (303 kJ mol^−1^) and fall in the relative narrow range (FIA=171–224 kJ mol^−1^). The relative order of the Lewis acidity for cations **2** and **9** on the FIA scale is close to that given by the experimental *δ* ^19^F NMR and ^1^ *J*(CF) NMR parameters, although there is no linear correlation between the experimental scales and the FIA scale (Figure 7 b). It is worth noting that for the limited subset of data of chalcogenyl‐substituted cations all three scales provide the same sequence of increasing Lewis acidity as they do for the subset of halo‐substituted cations (Figure 6). The missing correlation between the computed FIA scale and the two experimental nitrilium‐based scales might be rationalized by a closer inspection of the computed structures of cations **2**/**9**, nitrilium ions **10**/**11** and the corresponding silylfluoride **12**/**13**. For the series of selenyl‐substituted compounds, the computed structures are shown in Figure 8. By taking the sum of the bay angles Σβ as an indicator for kind of interaction between the selenyl and the silyl group, it becomes evident that there is small attractive interaction in the stabilized silyl cation **2 c** (Σβ°=357°). The interaction becomes slightly repulsive in the nitrilium ion **10 c** with a pentacoordinated silicon atom (Σβ°=365°) and finally the repulsion between the *peri*‐substituents is significant in silyl fluoride **12 c** (Σβ°=381°).^25^ This comparison indicates that the attractive donor–acceptor interaction originally present in cation **2 c**, is preserved to a certain extent in nitrilium ion **10 c** but it is significantly pushed back and is even repulsive in silyl fluoride **12 c**. This suggests that the scales based on the NMR parameters of the nitrilium ions **10** and **11** are better suited to gauge the modifications of the Lewis acidity by the intramolecular donor than the theoretical FIA scale is.

**Table 2:** Calculated structural parameters of cations **2**, **9** and nitrilium ions **10**, **11** and silyl fluorides **12**, **13** that are relevant to the discussion (M06‐2X/Def2‐TZVP). (Do‐Si: atomic distance donor atom silicon; Σ*β*: sum of the bay angles; *d*(Si), *d*(Do): distances of the silicon atom and donor atom from the plane that is spanned by the ten carbon atoms of the naphthalene ring, Σ*α*(SiC_3_); Σ*α*(Do): sum of the bond angles around the silicon atom and the donor atom). Experimental XRD data are given for comparison in parentheses. Cpd=compound.

Cpd	Do	Do−Si [pm]	Σβ [°]	Si−N [pm]	*d*(Si) [pm]	*d*(Do) [pm]	Σ*α*(SiC_3_) [°]	Σα(Do) [°]
**2 a**	OPh	185.4	337.3	–	0.0	0.0	352.2	360.0
**2 b**	SPh	232.1	353.4	–	18.1	−8.0	349.0	299.4
**2 c**	SePh	244.7 (243.1)	357.2 (355.9)	–	15.6 (39.2)	−6.8 (−0.1)	347.9 (346.1)	288.9 (288.4)
**2 d**	TeMes	261.4	361.8	–	5.1	−4.1	345.4	290.1
**2 e**	Br	247.0	358.4	–	0	0	351.6	–
**2 f**	I	264.3	363.0	–	0	0	349.5	–
**9 a**	OPh	183.2 (183.8)	337.7 (337.9)	– (–)	0.6 (3.2)	0.9 (9.5)	351.7 (351.3)	359.5 (360)
**9 e**	Br	243.9	358.6	–	0	0	350.7	–
**10 a**	OPh	244.7	355.1	191.4	0.01	−0.01	354.8	360.0
**10 b**	SPh	263.6	361.8	199.9	27.4	−17.5	359.4	300.6
**10 c**	SePh	275.6	364.8	199.7	28.8	−22.2	358.9	289.3
**10 d**	TeMes	292.2	369.8	201.9	13.0	−11.3	359.7	295.5
**10 e**	Br	302.9	373.7	190.0	1.9	−1.6	353.9	–
**10 f**	I	328.7	377.0	190.0	45.6	−42.9	355.0	–
**11 a**	OPh	219.1	348.2	196.2	0.3	−0.1	357.7	360.0
**11 c**	SePh	267.6	362.4	201.8	38.3	−31.9	359.3	290.2
**11 e**	Br	291.4	369.3	191.0	38.8	−29.2	355.2	–
**12 a**	OPh	285.8	367.8	–	6.4	−7.9	342.4	349.4
**12 b**	SPh	318.2	378.0	–	24.8	−16.5	345.2	289.5
**12 c**	SePh	328.5	380.5	–	21.0	−17.0	345.6	280.8
**12 d**	TeMes	352.5	384.4	–	32.5	−42.8	345.4	301.6
**12 e**	Br	333.2	381.9	–	23.3	−12.5	331.2	–
**12 f**	I	351.3	384.2	–	42.3	−30.8	330.4	–
**13 a**	OPh	270.7	363.6	–	5.8	−6.1	344.4	355.6
**13 e**	Br	326.0	378.3	–	49.6	−31.6	329.9	–

Free triarylsilylium ions, such as *tris*‐pentamethylphenyl silylium, **1** (Pemp_3_Si^+^), are not stable in CH_2_Cl_2_ solutions,12b therefore we quantified their Lewis acidity with nitrile **1** in chlorobenzene solution. The borate **1**[B(C_6_F_5_)_4_] was synthesized by substituent‐exchange reaction and identified by NMR spectroscopy.^12^ An equimolar amount of FBN was added and the formation of the corresponding nitrilium ion **14** was indicated by the substantially high‐field shifted ^29^Si resonance [*δ* ^29^Si(**1**)=216.8; *δ* ^29^Si(**14**)=−2.2]. As expected, the experimental NMR parameters of nitrilium ion **14** [*δ* ^19^F=−87.3, ^1^ *J*(CF)=270 Hz] characterize silylium ion **1** as a very strong Lewis acid, stronger than bromonium ion **2 e** and somewhat weaker than silylium ion **5**. As an example for an intermolecular stabilized silyl cation, we tested the reactivity of triethylsilyltoluenium, [Et_3_Si(C_7_H_8_)]^+^, versus FBN.6b As expected from several previous examples, the nitrile replaces the toluene molecule completely as indicated by the typical ^29^Si NMR chemical shift of *δ* ^29^Si=41.2.2c, 16 Consequently, also the NMR parameters of [Et_3_Si(FBN)]^+^ indicate the Lewis acidity of the free silylium ion Et_3_Si^+^ [*δ* ^19^F=−87.9, ^1^ *J*(CF)=270 Hz]. According to our measured NMR parameters, the difference between the Lewis acidity of the silylium ions **1**, **5** and Et_3_Si^+^ are small and reflect the stabilizing electronic effects of the different substituents. In view of the vastly different steric requirement of the substituents in these cations, it suggests also that steric effects on nitrilium ion formation are only of minor importance. In this respect, the linear arrangement of the atoms in the nitrile donor is certainly beneficial.

**Scheme 3:**
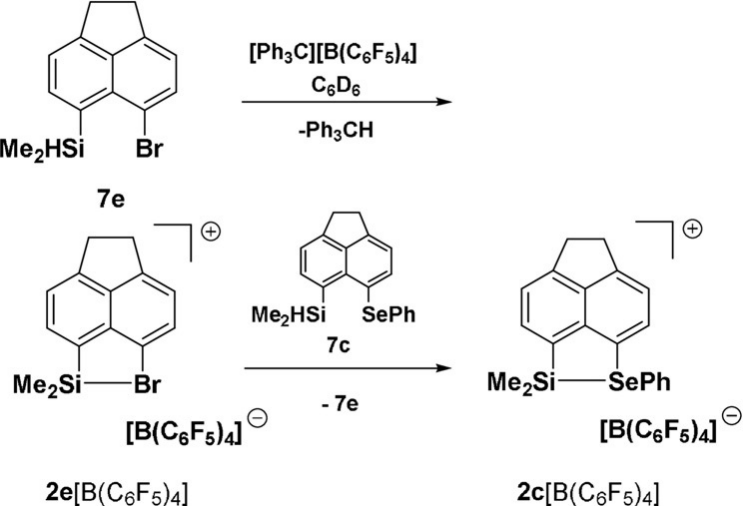
Hydride transfer to the bromo‐substituted silyl Lewis acid **2 e** with formation of the weaker selenyl‐substituted Lewis acid **2 c**.

The relative order of Lewis acidity was tested experimentally for the hydride transfer reaction of silane **7 c** with bromonium ion **2 e**. A biphasic solution of **2 e**[B(C_6_F_5_)_4_] was prepared by the standard Corey protocol from the silane **7 e** in [D_6_]benzene (Scheme 3). The clean formation of cation **2 e** was indicated by ^1^H NMR spectroscopy (see Supporting Information). Addition of one equivalent of selenyl silane **7 c** at room temperature gave the less Lewis acidic selenyl‐stabilized silyl cation **2 c** (*δ* ^29^Si=64, *δ* ^77^Se=254)^13^ and the bromoacenaphthyl silane **7 e** was recovered [*δ* ^29^Si=−15, *δ* ^1^H=5.46, 0.64 (SiHMe_2_)].

## Conclusion

**Figure 9:**
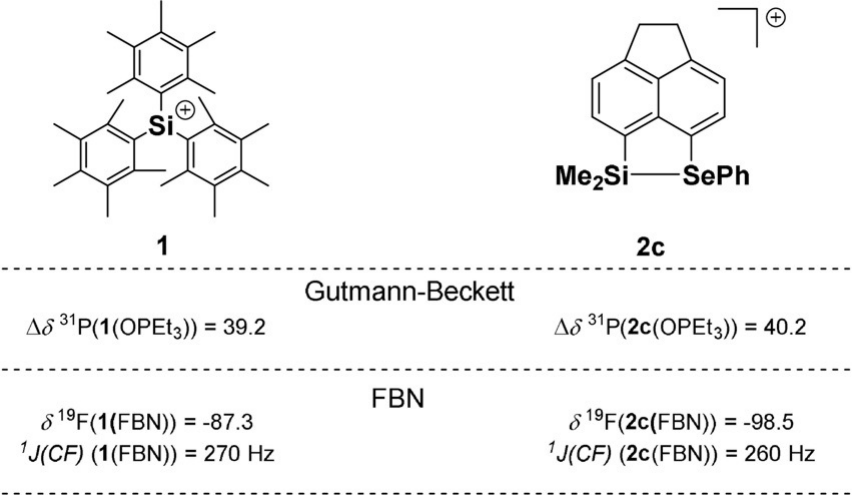
Comparison of the Gutmann–Beckett method with the FBN NMR probe for silylium ion **1** and silyl cation **2 c**.

The reactions of the weak donor *p*‐fluorobenzonitrile, FBN, with a family of internally stabilized silyl cations **2** and **9** were studied. The formed nitrilium ions **10** and **11** are stable in benzene solutions and in many cases, also in methylene chloride. They were fully characterized by NMR spectroscopy. Two spectroscopic parameters, the ^19^F NMR chemical shift of the *p*‐fluorine atom and the ^1^ *J*(CF) coupling constant between the fluorine atom and the *p*‐carbon atom were found to be sensitive to the different Lewis acidity of the investigated intramolecularly stabilized silyl cations. The NMR investigations that were supported by the results of density functional calculations indicate a trigonal bipyramidal coordination environment for the silicon atom in nitrilium ions **10** and **11**. Therefore, the coordination of the nitrile to the silicon center is not strong enough to cancel the interaction with the internal donor. As a consequence, the electronic situation of nitrilium ions **10** and **11** as disclosed in their NMR parameters reflect closely the original situation in the underlying silyl Lewis acids **2** and **9**. This suggests that FBN is a NMR probe well suited to distinguish and to gauge the Lewis acidity of silyl Lewis acids that are stabilized by relatively strong donors. This is shown by the clear discrimination between very similar silyl Lewis acids, such as iodine compound **2 f**, thiophenyl ether **2 b**, and phenylselenyl ether **2 c** (see Figure 6 a,b). The sequence found with the FBN probe is supported by the DFT calculations for the complexation energies with FBN, BDE (Si−N) (Table 1) and by competition experiments. The Lewis acidity based on the FBN scale parallels that of the theoretically derived FIA scale (Figure 6 c); there is, however, no linear correlation. The lack of proportionality between both scales can be rationalized by the stronger affinity of the fluoride ion used in the theoretical scale to silicon, which suppresses the small differences in the intramolecular donation by the different donor groups. Therefore, we suggest that the FBN NMR probe is well suited for gauging the actual Lewis acidity of a stabilized silyl Lewis acid. The probe is applicable to strong donors (weak Lewis acids), although there is clearly a limitation for very strong donors, which do not allow for an additional coordination of the nitrile. For weak donors (strong Lewis acids), their influence on the Lewis acidity of the donor‐stabilized acid is canceled as soon as they are replaced by the nitrile. The advantage of the FBN probe compared to established scales, such as the Gutmann–Beckett method, is demonstrated by the example quoted in the introduction, that is, the direct comparison between silylium ion **1** and the internally stabilized silyl cation **2 c**. Practically, the same Lewis acidity of both ions is suggested on the basis of the Gutmann–Beckett method, whereas the FBN probe differentiates substantially between both cations. This is a result that is expected from the vastly different reactivity of silylium ion **1** and intramolecularly stabilized silyl cation **2 c** (Figure 9).

The FBN NMR probe in our hands is a valuable extension of the toolbox of methods for quantifying the actual acidity of stabilized Lewis acids, which do find more and more applications in synthesis. It allows for the discrimination between silyl cations stabilized by donors of very similar donor ability and it is therefore useful for the design and fine tuning of silyl Lewis acids in particular and of Lewis acids in general. Currently, we are applying the FBN method to other intramolecularly stabilized cationic Lewis acids.

## Conflict of interest

The authors declare no conflict of interest.

## Supporting information

As a service to our authors and readers, this journal provides supporting information supplied by the authors. Such materials are peer reviewed and may be re‐organized for online delivery, but are not copy‐edited or typeset. Technical support issues arising from supporting information (other than missing files) should be addressed to the authors.

Supplementary
