# Supplementary material for: An Experimental Acidity Scale for Intramolecularly Stabilized Silyl Lewis Acids
Source: Chemistry. 2019 Oct 31;25(66):15123–30. doi: 10.1002/chem.201903241 (PMC6899571; doi:10.1002/chem.201903241)
Supplement: Supplementary file 1 — Supplementary [file CHEM-25-15123-s001.pdf]

CHEMISTRY
A European Journal

Supporting Information

An Experimental Acidity Scale for Intramolecularly Stabilized Silyl Lewis Acids

Sandra Künzler+, Saskia Rathjen+, Anastasia Merk+, Marc Schmidtmann, and Thomas Müller*[a]

chem_201903241_sm_miscellaneous_information.pdf

1. Experimental part 1
1.1. General Remarks 1
1.2. Syntheses of Silanes 3
1.3. Syntheses of Nitrilium Ions 9
1.4. 4-Fluorobenzonitrile + Tritylborate in CD_2Cl_2 53
1.5. Tris(pentafluorophenyl)borane 4-fluorobenzonitrile adduct **4** 55
1.6. Gutmann-Beckett method for silyl cation **2c** 59
1.7. Variable temperature NMR measurements of nitrilium ion **10c** with an excess 4-fluorobenzonitrile 61
1.8. Reaction of bromonium borate **2e**$[B(C_6F_5)_4]$ with silane **7c** 62
1.9. Data from X-ray diffraction analysis of compounds 66
1.9.1. Data from X-ray diffraction analysis of compound BCF adduct **4** 66
1.9.2. Data from X-ray diffraction analysis of compound [**2c**]$_2[B_{12}Br_{12}]$ 68
2. Computational Details 70
2.1. Computed Structures XYZ-files 72
3. References 102

1. Experimental part

1.1. General Remarks

All experiments were carried out under argon or nitrogen atmosphere using Schlenk techniques. The glass equipment was stored in an oven at 120°C and evacuated prior to use. The solvents *n*-pentane, *n*-hexane, benzene, tetrahydrofuran and diethyl ether were dried over sodium-potassium alloy and distilled under nitrogen atmosphere. Dichloromethane was dried over sodium hydride. The deuterated solvents were first dried over sodium-potassium alloy and then either condensed before use or stored over molecular sieve (4 Å). Dichloromethane-d_2 and trimethylsilane were stored over molecular sieve (4 Å). Commercially available solid materials were stored and weighted in a glove box or dried at high vacuum before use. *n*-Butyl lithium was used as a 1.6 M solution in *n*-hexane. N,N,N′,N′-Tetramethylethylenediamine (TMEDA) was dried over potassium hydroxide and freshly condensed before use. 1,8-dibromonaphthalene, 5,6-dibromoacenaphtene, 5-dimethylsilylacenaphthene, 6-phenoxy-5-dimethylsilylacenaphthene **7a**, 6-dimethylsilyl-5-thiophenylace-naphthene **7b**, 5-phenylselenyl-6-dimethylsilylacenaphthene **7c**, 6-dimethylsilyl-5-mesityltellurylacenaphthene **7d**, 8-phenoxy-1-dimethylsilylnaphthalene **8a**, 1-bromo-8-dimethylsilylnaphthalene **8e**, b*is*(pentamethylphenyl)methylsilane and trityl borate $[Ph_3C][B(C_6F_5)_4]$ were synthesized according to literature procedures.[S1-S7] 5,6-Diiodomoacenaphtene: The original procedure [S8] was slightly modified. The reaction mixture was stirred for 1 h at -10 °C – 0 °C after the addition of a solution of iodine in diethylether at -10 °C – 0 °C and was allowed to warm to room temperature overnight. 5,6-Diiodoacenaphthene was obtained as a colorless solid after workup and column chromatography using *n*-pentane (R_f = 0.41) as eluent.

Tris-(2,3,4,5,6-pentamethylphenyl)silylium borate, **1**$[B(C_6F_5)_4]$: The original procedure [S7] was slightly modified to allow easy handling of the silylium borate. The solvent was removed and the residue was dried in vacuum before the schlenk tube was transferred into the glove box. Then, the residue was pulverized in a mortar until a yellow powder of *tris*(2,3,4,5,6-pentamethylphenyl)silylium borate **1**$[B(C_6F_5)_4]$ was obtained and stored under nitrogen. Silyl arenium borate $[Et_3Si(C_7D_8)][B(C_6F_5)_4]$ was prepared according to literature procedure using toluene-d_8 as solvent.[S9]

Thin-layer chromatography was performed using commercial available aluminum foil (Fluka) coated with silica gel 60 and fluorescent indicator F254. For the column chromatography silica gel of the mesh size 60 from Merck was used.

NMR spectra were recorded on Bruker Avance 500, Bruker Avance III 500 spectrometer. ^{1}H NMR spectra were referenced to the residual solvent resonance as internal standard (benzene-d_6: δ^{1}H(C_6D_5H) = 7.20, toluene-d_8: δ^{1}H($C_6D_5CD_2H$) = 2.08, chlorobenzene-d_5: δ^{1}H(C_6D_4HCl) = 7.14, dichloromethane-d_2: δ^{1}H($CDHCl_2$) = 5.32) and ^{13}C NMR spectra by using the central line of the solvent signal (benzene-d_6: δ^{13}C(C_6D_6) = 128.0, toluene-d_8: δ^{13}C($C_6D_5CD_3$) = 20.4, chlorobenzene-d_5: δ^{13}C(C_6D_5Cl) = 134.2, dichloromethane-d_2: δ^{1}H(CD_2Cl_2) = 54.0). ^{29}Si NMR spectra were referenced to an external standard (δ ^{29}Si(Me_2SiHCl) = 11.1 versus tetramethylsilane (TMS)), ^{11}B NMR spectra against $BF_3{\cdot}OEt_2$ (δ^{11}B($BF_3{\cdot}OEt_2$) = 0.0) and ^{77}Se NMR spectra against external Me_2Se (δ^{77}Se(Me_2Se) = 0.0). The ^{31}P NMR spectra were calibrated using an external standard (δ^{31}P(($H_3CO)_3P$) = 141.0 versus 85% H_3PO_4(aq)).The ^{29}Si NMR inverse gated spectra were recorded with a relaxation delay D1 = 10 s. The ^{29}Si INEPT spectra were recorded with delays D3 = 0.0084 s and D4 = 0.0313 s, if not given otherwise.

Reference sample for the ^{19}F NMR chemical shift

The NMR sample was prepared using fluorobenzene (302 µmol, 28 µL), trityl borate (302 µmol, 278 mg) and 4-fluorobenzonitrile (302 µmol, 37 mg) in 0.55 mL dichloromethane-d_2.

Table S1 – ^{19}F NMR chemical shifts of the *p*-fluorine of $[B(C_6F_5)_4]^-$ and free 4-fluorobenzonitrile (FBN) referenced against fluorobenzene $\delta^{19}F$ = -113.78 at different temperatures.

T [K]	$\delta^{19}F$ $[B(C_6F_5)_4]^-$	$\delta^{19}F$ FBN
305	-163.44	-103.43
293	-163.37	-
283	-163.29	-103.38
273	-163.20	-
263	-163.12	-103.33
253	-163.04	-
243	-162.96	-103.30
233	-162.87	-103.29
223	-162.79	-103.30
213	-162.73	-
203	-162.63	-103.30
193	-162.57	-103.31

1.2. Syntheses of Silanes

***5-bromo-6-dimethylsilylacenaphtene* 7e**

5,6-Dibromoacenaphthene (2.00 g, 6.41 mmol), was suspended in 50 mL THF and cooled to -80 °C. Then *n*-butyl lithium (1.6 M in *n*-hexane, 4.0 mL, 6.41 mmol) was added dropwise and the reaction mixture was stirred for 1.5 hours at the same temperature. After that chlorodimethylsilane (0.72 mL, 6.41 mmol) was added and the mixture was stirred for an additional hour at -80 °C. Then the reaction mixture was allowed to warm to room temperature overnight. After adding NH_4Cl solution (10 mL) to the reaction mixture and extraction with *n*-pentane (3 x 10 mL), the organic phase was dried over $MgSO_4$ and the solvent was removed under low pressure. Silane **7e**

was obtained as a colorless solid after column chromatography using *n*-pentane (R_f = 0.48) as eluent. Yield: 1.72 g (5.93 mmol, 93 %, **Fp**: 35-37 °C)

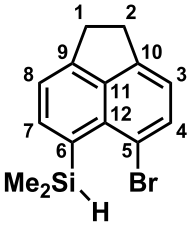

7e

^{1}H NMR (500.13 MHz, 298.0 K, C_6D_6): δ = 0.68 (d, 6H, $^3J_{H,H}$ = 3.6 Hz, $Si(CH_3)_2$), 2.76-2.80 (m, 2H, CH_2), 2.86-2.91 (m, 2H, CH_2), 5.56 (sept, 1H, $^3J_{H,H}$ = 3.6 Hz, $^1J_{H,Si}$ = 196.9 Hz, Si-H), 6.72 (d, 1H, $^3J_{H,H}$ = 7.3 Hz, H-3), 7.04 (d, 1H, $^3J_{H,H}$ = 7.0 Hz, H-8), 7.69 (d, 1H, $^3J_{H,H}$ = 7.3 Hz, H-4), 7.98 (d, 1H, $^3J_{H,H}$ = 7.0 Hz, H-7). **^{13}C{^{1}H} NMR** (125.77 MHz, 298.1 K, C_6D_6): δ = 0.7 ($Si(CH_3)_2$), 29.6 (CH_2), 30.2 (CH_2), 118.1 (C^q, C-5), 119.9 (CH, C-8), 120.5 (CH, C-3), 131.2 (C^q, C-6), 133.5 (CH, C-4), 135.9 (C^q, C-12), 139.9 (CH, C-7), 141.4 (C^q, C-11), 146.8 (C^q, C-10), 149.2 (C^q, C-9). **^{29}Si{^{1}H} NMR** (99.31 MHz, 305.0 K, C_6D_6): δ = -14.5. **^{29}Si{^{1}H} INEPT NMR** (99.31 MHz, 305.0 K, C_6D_6, D_3 = 0.0084 s, D_4 = 0.0313 s): δ = -14.4. **^{29}Si INEPT NMR** (99.31 MHz, 305.0 K, C_6D_6, D_3 = D_4 = 0.0013 s): δ = -14.3 (dm, $^1J_{H,Si}$ = 197.0 Hz). **IR** (ATR, neat): $\tilde{\nu}$ = 2170 (SiH) cm^{-1}. **HR/MS** (ESI, pos.): [$^{12}C_{14}{}^1H_{15}{}^{79}Br^{28}Si$], calculated 290.013; measured: 313.0032 [$^{12}C_{14}{}^1H_{15}{}^{79}Br\ {}^{28}Si^{23}Na$]. **EA**: $C_{14}H_{15}BrSi$, calculated: C 57.56, H 5.92; measured: C 57.73, H 5.19.

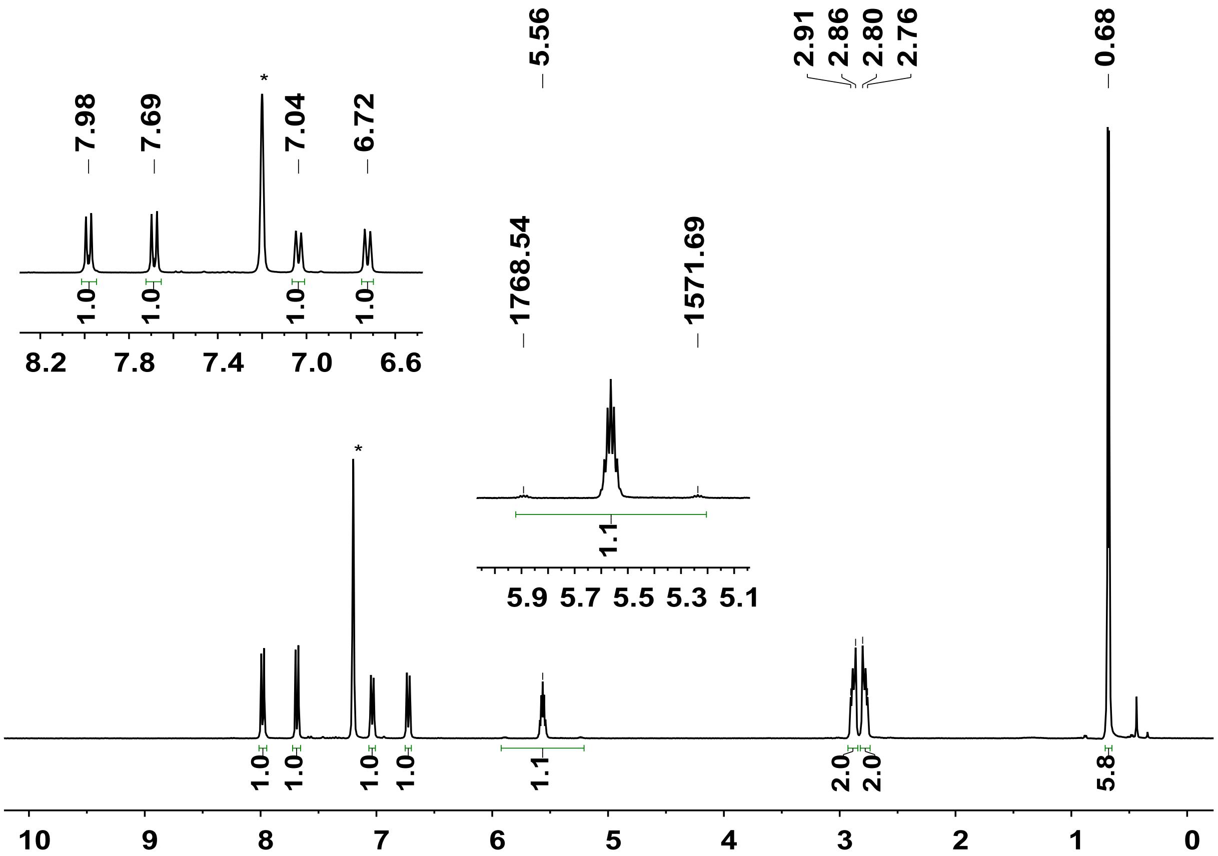

Figure S1 – ^{1}H NMR spectrum (500.13 MHz, 298.0 K, C_6D_6, *C_6D_5H) of silane 7e.

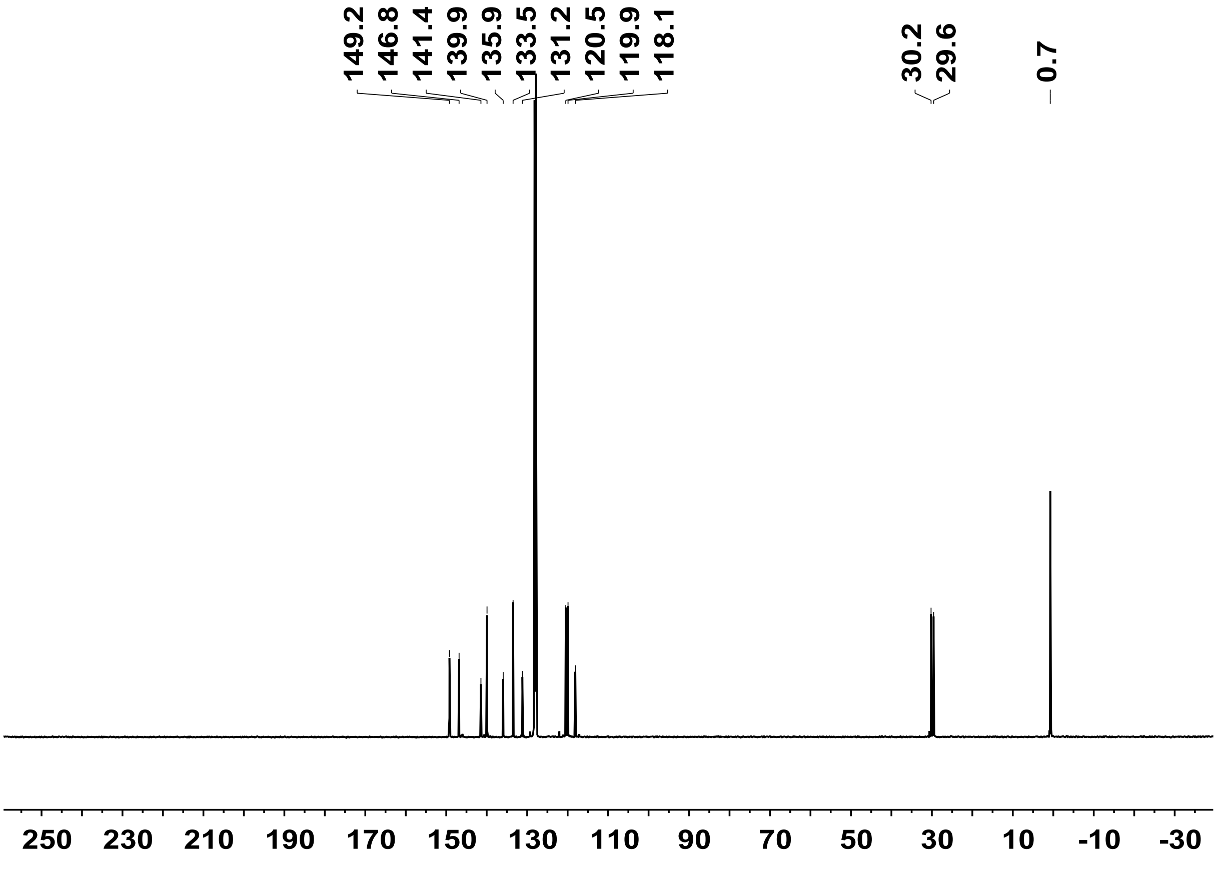

Figure S2 – $^{13}C\{^{1}H\}$ NMR spectrum (125.77 MHz, 298.1 K, C_6D_6, *C_6D_6) of silane 7e.

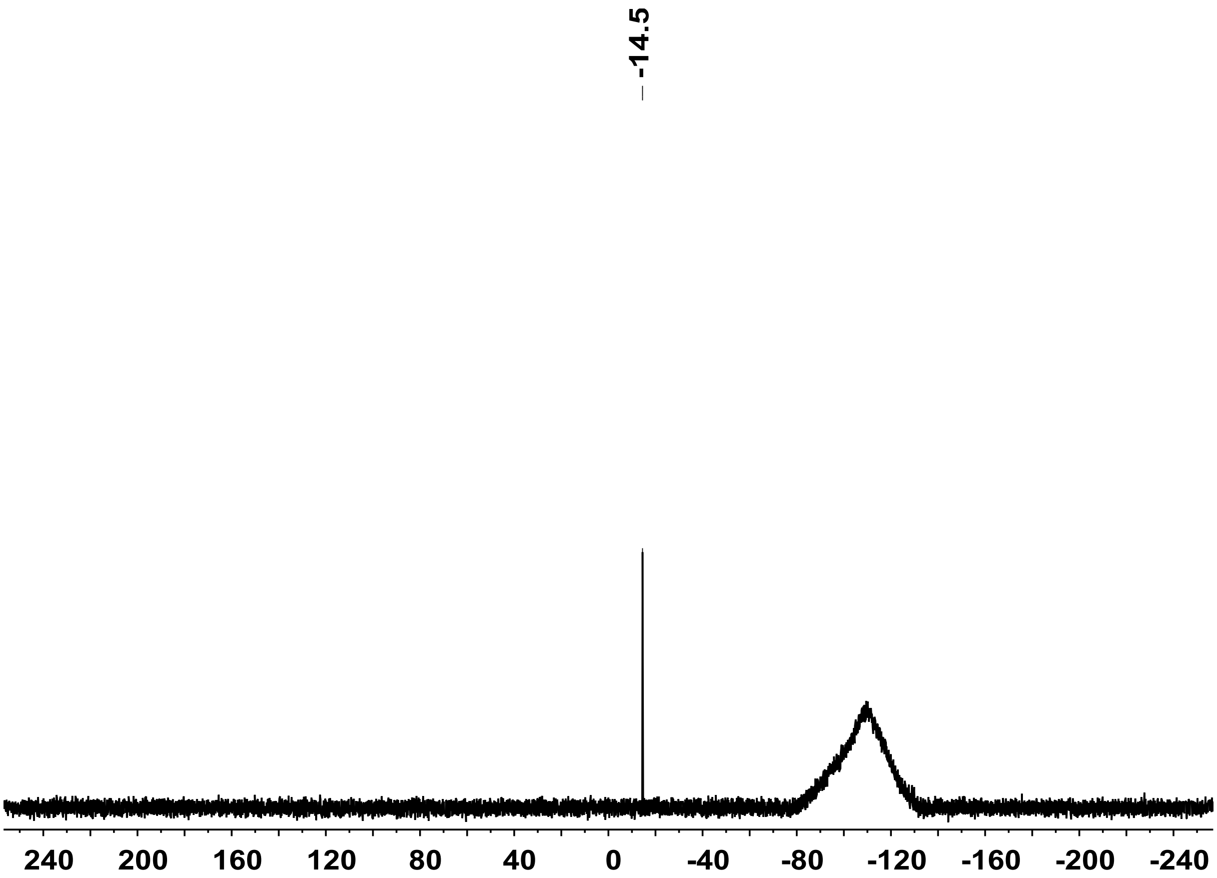

Figure S3 – $^{29}Si\{^1H\}$ NMR spectrum (99.31 MHz, 305.0 K, C_6D_6) of silane 7e.

***5-iodo-6-dimethylsilylacenaphtene* 7f**

5,6-Diiodoacenaphthene (0.93 g, 2.29 mmol), was suspended in 30 mL diethylethter and cooled to -80 °C. Then *n*-butyl lithium (1.6 M in *n*-hexane, 1.4 mL, 2.29 mmol) was added dropwise and the reaction mixture was stirred for 1 hour at the same temperature. After that 15 mL diethylethter was added and the reaction mixture was stirred for 1.5 hours at -50 °C. Chlorodimethylsilane (0.26 mL, 2.29 mmol) was added and the mixture was stirred for an additional hour at -50 °C. Then the reaction mixture was allowed to warm to r.t. overnight. Silane **7f** decomposes by performing an aqueous workup and needs to be stored under inert conditions. The solvent was removed under low pressure, the residue was dried in vacuum and suspended in *n*-pentane. The solution was transferred into another Schlenk flask, the solid was washed two more times and the obtained solutions were also added to the Schlenk flask. After that the solvent was removed again under low pressure and the residue was dried in vacuum. Silane **7f** was obtained as the main product in a reaction mixture with 5-iodoacenaphthene and a small amount of 5-dimethylsilylacenaphthene.

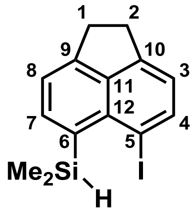

7f

^{1}H NMR (499.87 MHz, 305.1 K, C_6D_6): δ = 0.70 (d, 6H, $^3J_{H,H}$ = 3.6 Hz, $Si(CH_3)_2$), 2.75-2.86 (m, 4H, 2 x CH_2), 5.96 (sept, 1H, $^3J_{H,H}$ = 3.6 Hz, $^1J_{H,Si}$ = 199.0 Hz, Si-H), 6.56 (d, 1H, $^3J_{H,H}$ = 7.3 Hz, H-3), 6.98 (d, 1H, $^3J_{H,H}$ = 7.0 Hz, H-8), 7.88 (d, 1H, $^3J_{H,H}$ = 7.0 Hz, H-7), 8.03(d, 1H, $^3J_{H,H}$ = 7.3 Hz, H-4). **^{13}C{^{1}H} NMR** (125.77 MHz, 299.0 K, C_6D_6): δ = 1.4 ($Si(CH_3)_2$), 29.6 (CH_2), 29.9 (CH_2), 91.0 (C^q, C-5), 119.7 (CH, C-8), 121.3 (CH, C-3), 132.7 (C^q, C-6), 139.2 (C^q, C-12), 139.9 (CH, C-7), 141.2 (C^q, C-11), 142.1 (CH, C-4), 147.8 (C^q, C-10), 149.5 (C^q, C-9). **^{29}Si{^{1}H} NMR** (99.36 MHz, 298.4 K, C_6D_6): δ = -19.1.

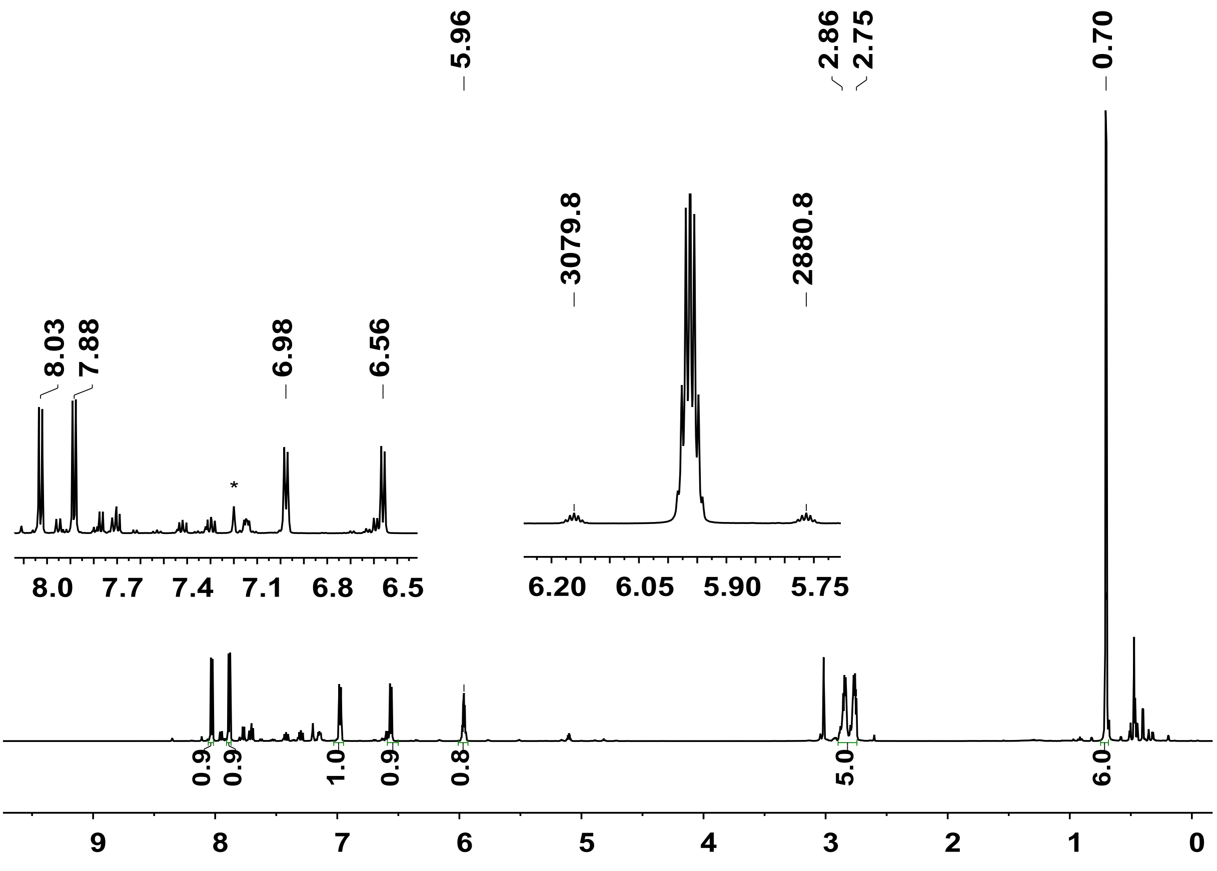

Figure S4 – ^{1}H NMR spectrum (499.87 MHz, 305.1 K, C_6D_6, *C_6D_5H) of the reaction mixture.

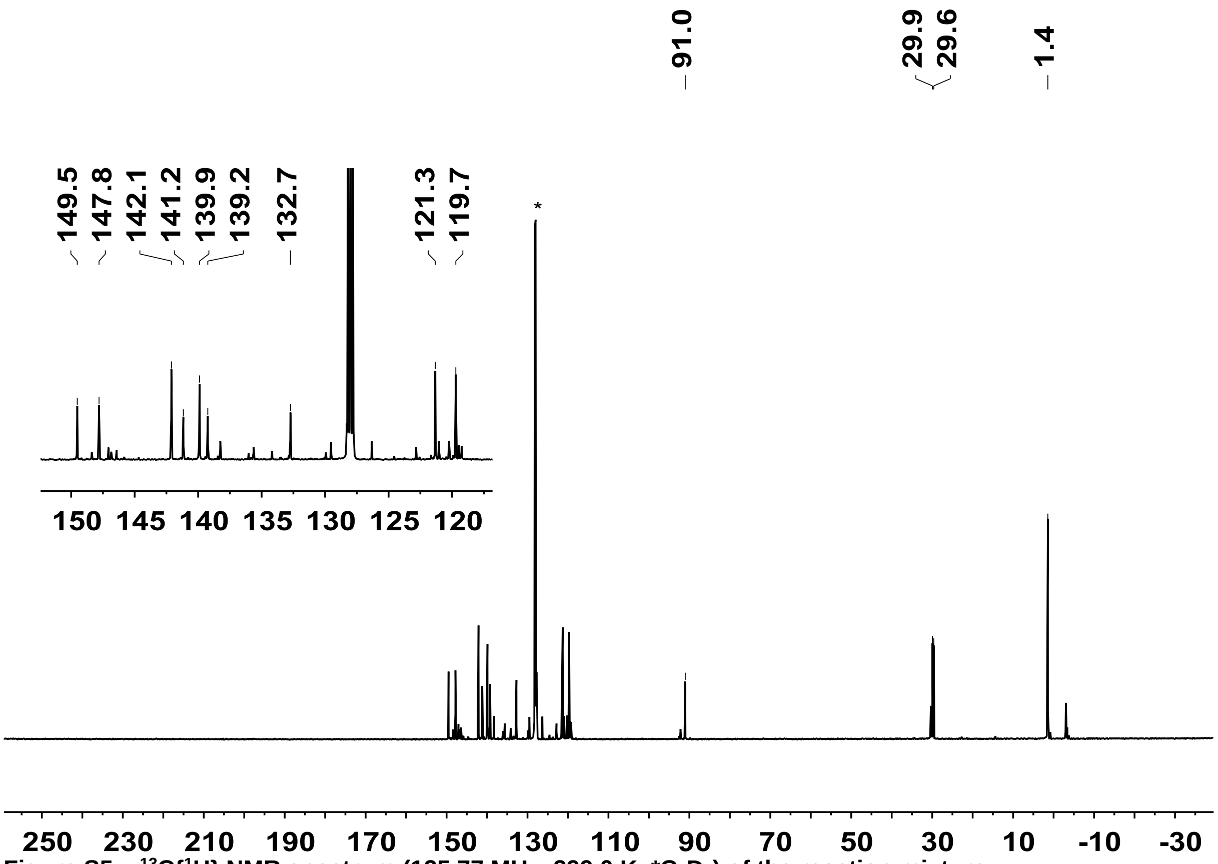

Figure S5 – $^{13}C\{^{1}H\}$ NMR spectrum (125.77 MHz, 299.0 K, *C_6D_6) of the reaction mixture.

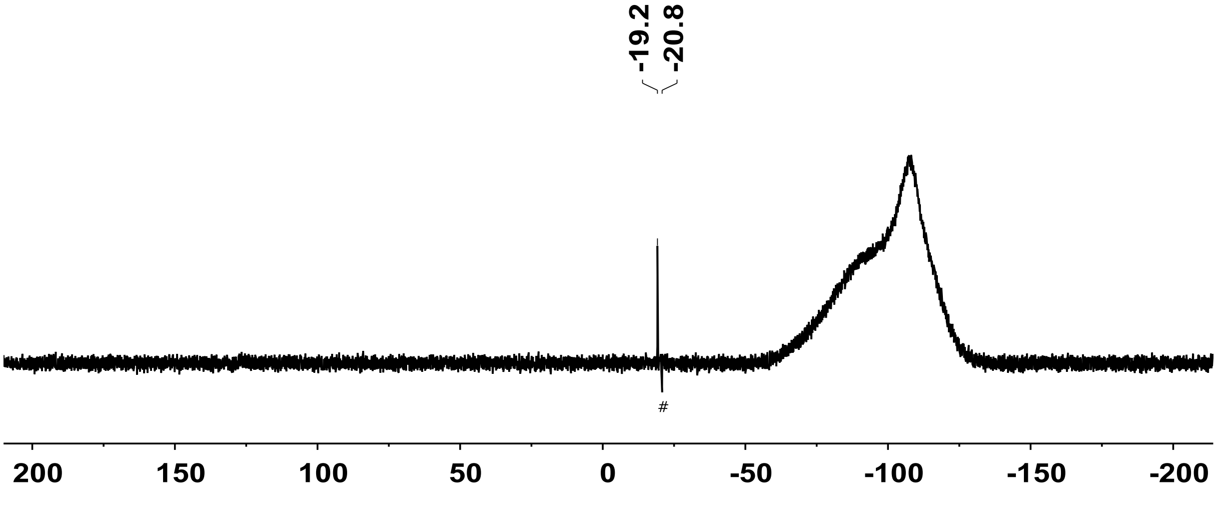

Figure S6 – $^{29}Si\{^{1}H\}$ NMR spectrum (99.36 MHz, 298.4 K, C_6D_6, #5-dimethylsilylacenaphthene) of the reaction mixture.

1.3. Syntheses of Nitrilium Ions

General Procedure A

A Schlenk tube was charged with the corresponding silane and trityl borate $[Ph_3C][B(C_6F_5)_4]$ and the solids were dissolved in benzene. The reaction mixture was stirred at r.t. for 20 min. Then, the phases were separated, the upper, nonpolar phase was removed and the polar phase was washed twice with benzene. After removing the solvent under reduced pressure, 4-fluorobenzonitrile was dissolved in 0.6 mL deuterated methylene chloride or deuterated benzene and added to the residue. Afterwards, the reaction mixture was stirred for further 15 min and subsequently transferred to a NMR tube for analysis.

General procedure B

Trityl borate $[Ph_3C][B(C_6F_5)_4]$ and 4-fluorobenzonitrile were dissolved in dichloromethane-d_2. This solution was added to a solution of the corresponding silane in dichloromethane-d_2. The reaction mixture was stirred for 5 min and subsequently transferred to a NMR tube for analysis.

General Procedure C

In a Schlenk tube the corresponding silane and trityl borate $[Ph_3C][B(C_6F_5)_4]$ were dissolved in benzene-d_6 and stirred for 30 min. After the biphasic reaction mixture was analyzed by NMR spectroscopy, the nonpolar phase was removed. The polar phase was first washed with a mixture of benzene and pentane and then with pentane. After removing the solvent under reduced pressure, the residue was dissolved in dichloromethane-d_2. To this solution 4-fluorobenzonitrile was added and the reaction mixture was stirred for 10 min and after that analyzed by NMR spectroscopy.

Phenoxyl substituted derivatives

Nitrilium borate **10a**$[B(C_6F_5)_4]$ was synthesized according to general procedure **A** using 1.0 equiv. 6-phenoxy-5-dimethylsilylacenaphthene (379.46 µmol, 116 mg), 1.0 equiv. trityl borate (379.46 µmol, 349 mg) and 1.0 equiv. 4-fluorobenzonitrile (379.46 µmol, 46 mg) (NMR spectroscopy in benzene-d_6) or general procedure **B** using 1.0 equiv. 5-diemethylsilyl-6-phenoxyacenaphthen (132.36 µmol, 40 mg), 1.0 equiv. tritylborate (132.36 µmol, 122 mg) and 1.0 equiv. 4-fluorobenzonitrile (132.36 µmol, 16 mg). Nitrilium ion **10a** decomposes in dichloromethane-d_2 within one day.

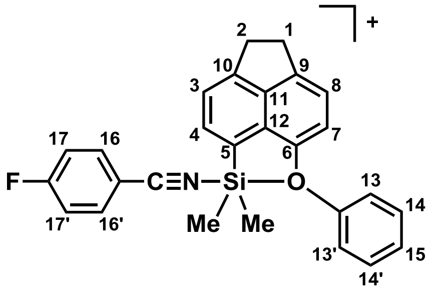

10a $[B(C_6F_5)_4]^-$

^{1}H NMR (499.87 MHz, 305.1 K, C_6D_6): δ = 0.74 (s, 6 H, $Si(CH_3)_2$), 3.03-3.05 (m, 2 H, CH_2, H-1), 3.16-3.18 (m, 2H, CH_2, H-2), 6.58-6.64 (m, 3 H, H-17, H-17', H-7), 6.89-6.91 (m, 2 H, H-13, H-13'), 6.92-6.96 (m, 3 H, H-16, H-16', H-8), 7.00-7.05 (m, 1 H, H-15), 7.14-7.19 (m, 2 H, H-14, H-14'), 7.31 (d, $^3J_{H,H}$ = 7.2 Hz, 1 H, H-3), 7.72 (d, $^3J_{H,H}$ = 7.1 Hz, 1 H, H-4). **^{13}C{^{1}H} NMR** (125.71 MHz, 305.0 K, C_6D_6): δ = 0.8 ($Si(CH_3)_2$), 29.7 (CH_2), 31.3 (CH_2), 98.4 (<u>C</u>-CN), 113.0 (C-7), 115.5 (C-5), 118.3 (d, $^2J_{C,F}$ = 23.5 Hz, C-17, C-17'), 120.5, 120.6 (C-3, C-8), 121.0 (C-13, C-13´), 122.0 (CN), 123.9-126.0 (C, $[B(C_6F_5)_4]^-$), 126.3 (C-15), 126.9 (C-12), 131.0 (C-14, C-14´), 136.4 (C-4), 137.0 (dm, $^1J_{C,F}$ = 233.2 Hz, CF, $[B(C_6F_5)_4]^-$), 137.7 (d, $^3J_{C,F}$ = 11.2 Hz, C-16, C-16'), 138.9 (dm, $^1J_{C,F}$ = 232.6 Hz, CF, $[B(C_6F_5)_4]^-$), 140.8 (C-11), 142.0 (C-9), 149.0 (d, $^1J_{C,F}$ = 239.0 Hz, CF, $[B(C_6F_5)_4]^-$), 151.2 (C-6), 152.9 (C-10), 154.2 (C, C-Ph), 168.7 (d, $^1J_{C,F}$ = 268.3 Hz, CF). **^{19}F{^{1}H} NMR** (470.30 MHz, 305.0 K, C_6D_6): -167.5-(-167.3) (m, 8 F, *m*-F, $[B(C_6F_5)_4]^-$), -163.44 (t, $^3J_{F,F}$ = 20.5 Hz, 4 F, *p*-F, $[B(C_6F_5)_4]^-$), -133.0-(-132.8) (m, 8 F, *o*-F, $[B(C_6F_5)_4]^-$), -89.8 (s, 1 F, CF). **^{29}Si{^{1}H} NMR** (99.31 MHz, 305.0 K, C_6D_6): δ = 15.4. **^{11}B{^{1}H} NMR** (160.38 MHz, 305.1 K, C_6D_6): δ = -16.1.

^{1}H NMR (499.87 MHz, 305.1 K, CD_2Cl_2): δ = 1.08 (s, 6 H, $Si(CH_3)_2$), 3.44-3.46 (m, 2 H, CH_2, H-1), 3.54-3.56 (m, 2H, CH_2, H-2), 6.84 (d, $^{3}J_{H,H}$ = 7.7 Hz, 1 H, H-7), 7.18-7.20, 7.24-7.29, 7.31-7.37 (3 m, 19 H, H-8, CH-Ph, Ph_3CH), 7.42-7.46 (m, 2H, H-17, H-17´), 7.49-7.53 (m, 3 H, H-3, CH-Ph), 7.94 (d, $^{3}J_{H,H}$ = 7.0 Hz, 1 H, H-4), 8.00-8.03 (m, 2 H, H-16). **^{19}F{^{1}H} NMR** (470.28 MHz, 305.1 K, CD_2Cl_2): δ = -167.4-(-167.2) (m, 8 F, *m*-F, $[B(C_6F_5)_4]^-$), -163.44 (t, $^{3}J_{F,F}$ = 20.5 Hz, 4 F, *p*-F, $[B(C_6F_5)_4]^-$), -133.0-(-132.9) (m, 8 F, *o*-F, $[B(C_6F_5)_4]^-$), -87.9 (s, 1 F, ^{1}J(CF) = 269.1 Hz). **^{29}Si{^{1}H} NMR** (99.31 MHz, 305.0 K, CD_2Cl_2): δ = 16.3.

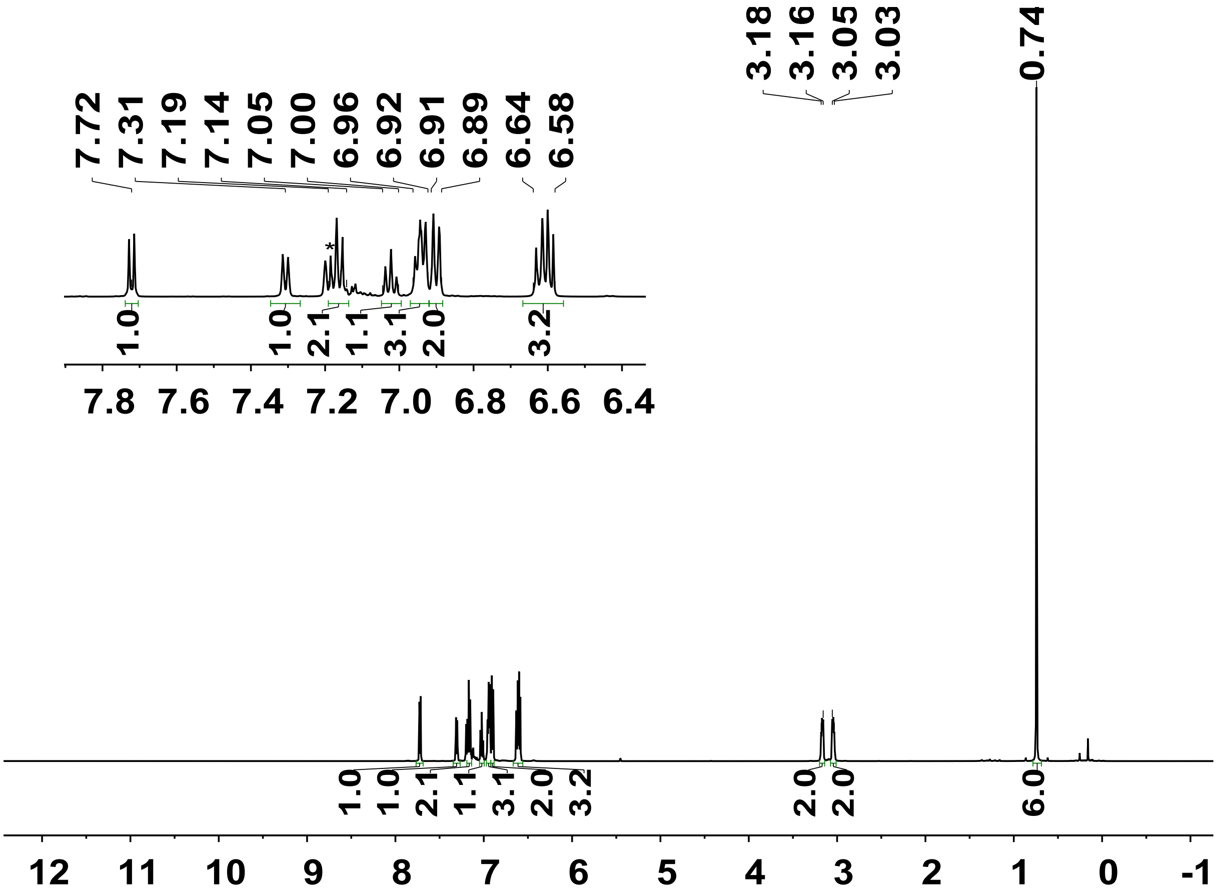

Figure S7 – ^{1}H NMR spectrum (499.87 MHz, 305.1 K, C_6D_6, lb: 0.3 Hz, *C_6D_5H) of 10a[$B(C_6F_5)_4$].

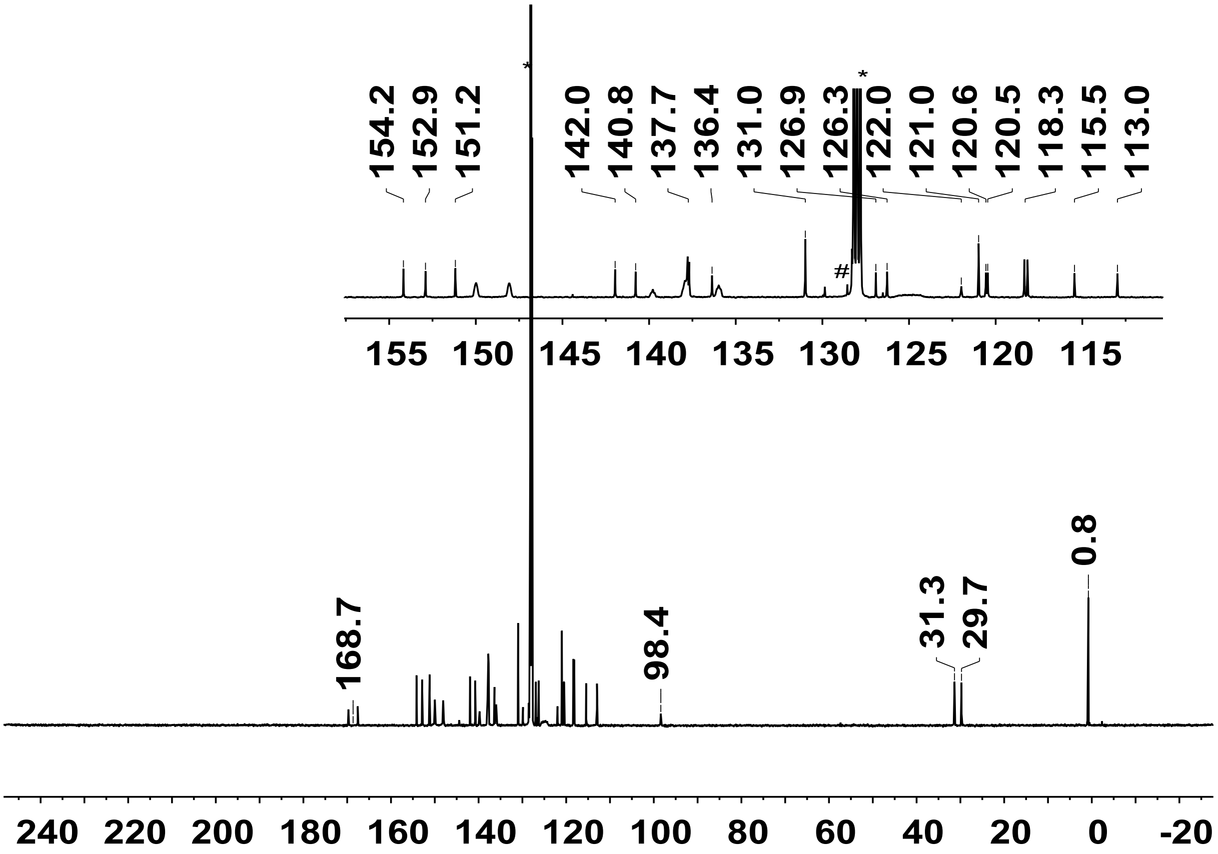

Figure S8 – $^{13}C\{^{1}H\}$ NMR spectrum (125.71 MHz, 305.0 K, C_6D_6, *C_6D_6, #Ph_3CH) of 10a[B(C_6F_5)$_4$].

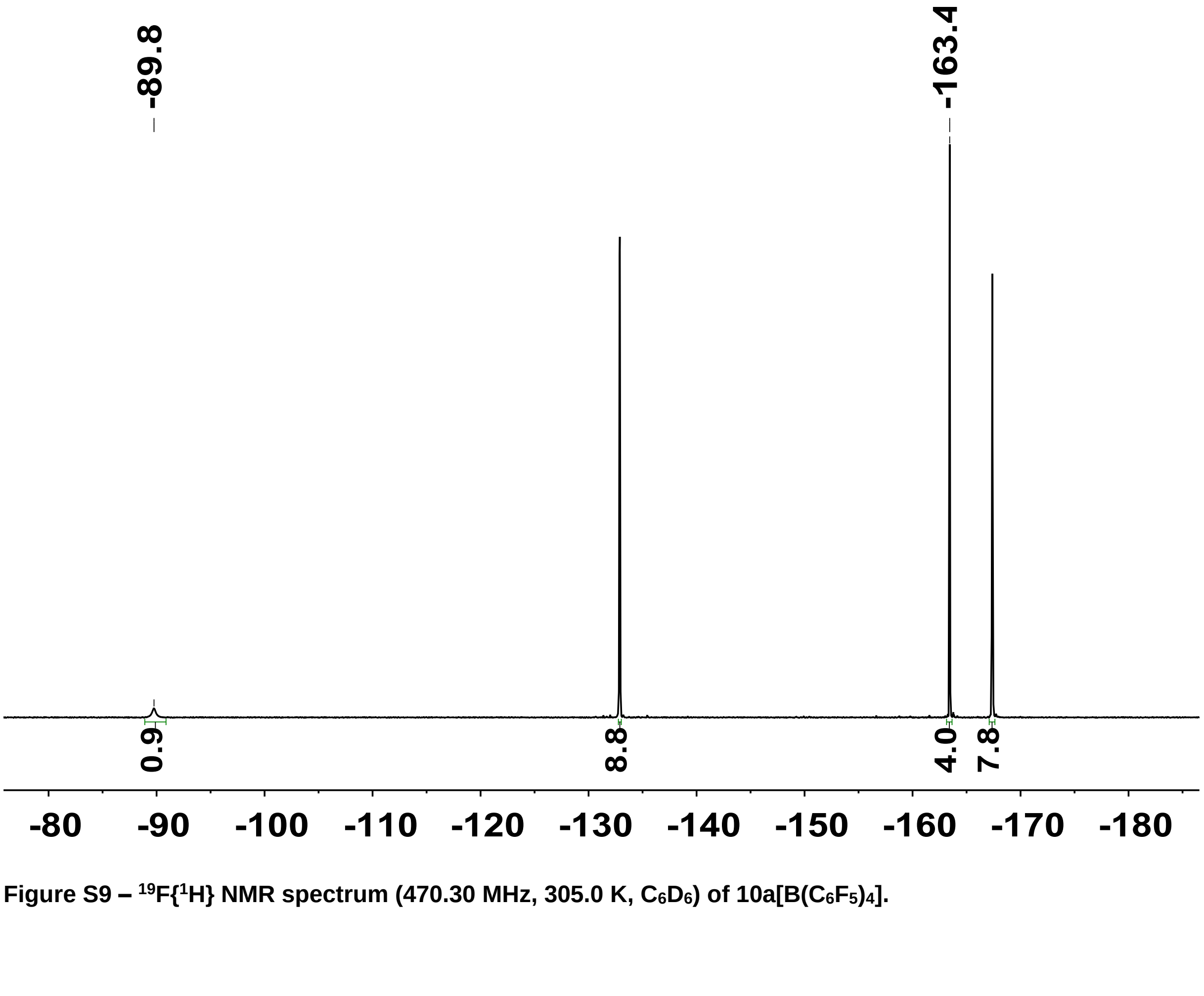

Figure S9 – $^{19}F\{^{1}H\}$ NMR spectrum (470.30 MHz, 305.0 K, C_6D_6) of 10a[B(C_6F_5)$_4$].

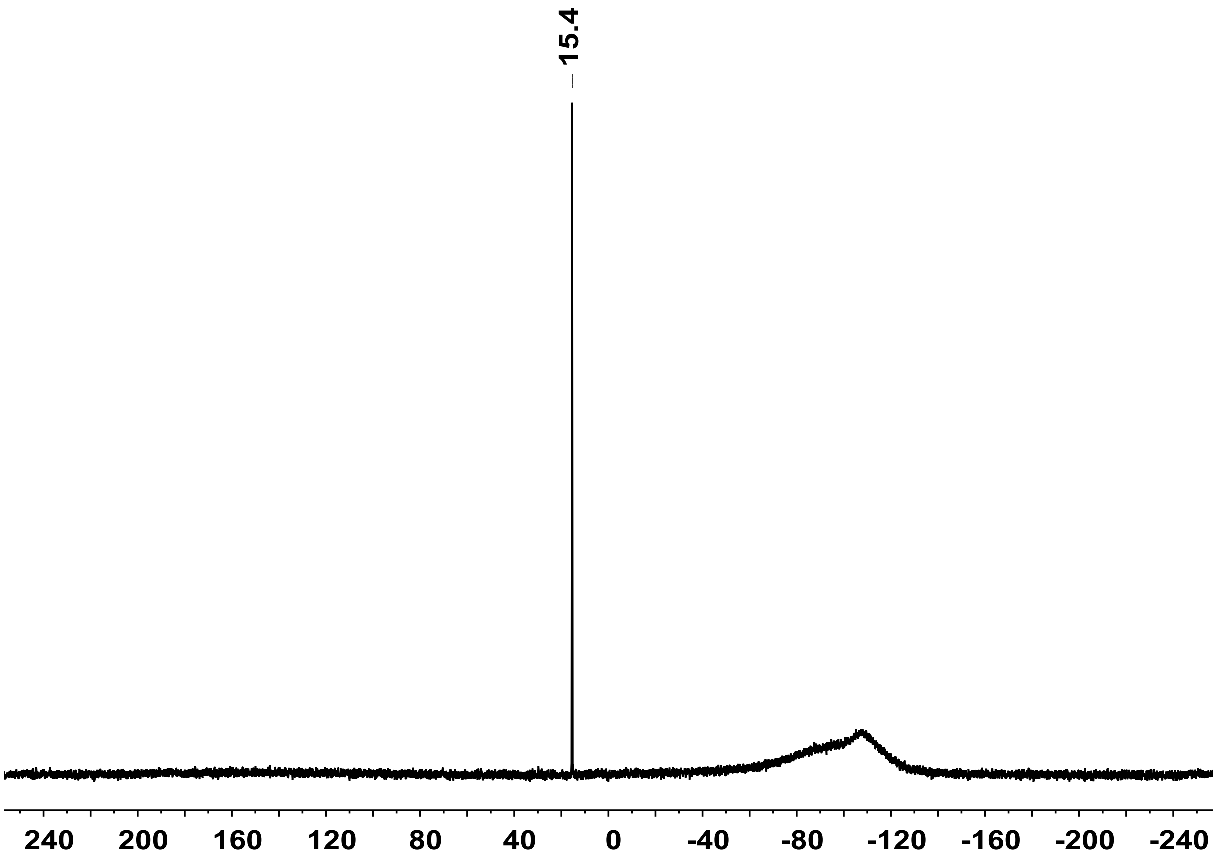

Figure S10 – $^{29}Si\{^{1}H\}$ NMR spectrum (99.31 MHz, 305.0 K, C_6D_6) of 10a[$B(C_6F_5)_4$].

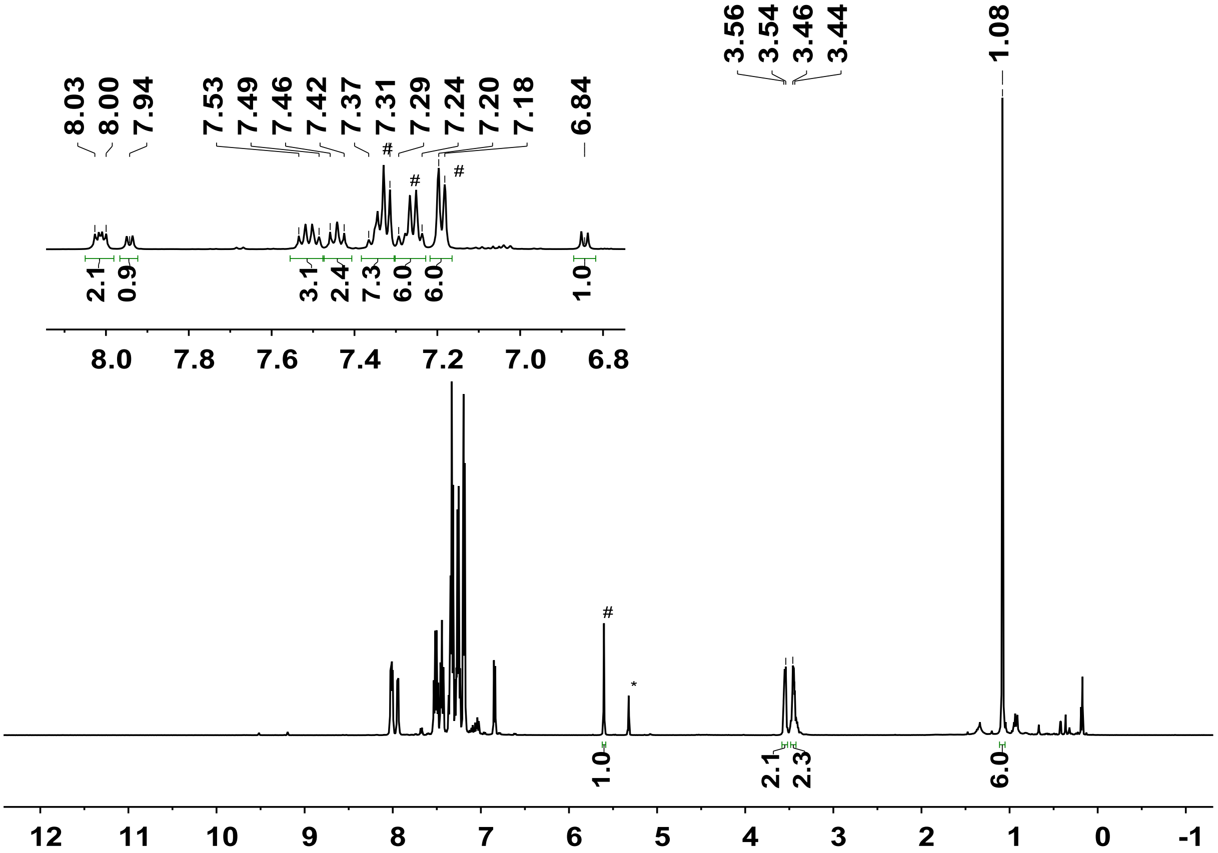

Figure S11 – ^{1}H NMR spectrum (499.87 MHz, 305.1 K, CD_2Cl_2, lb: 0.3 Hz, *$CDHCl_2$, $^{\#}Ph_3CH$) of 10a[$B(C_6F_5)_4$].

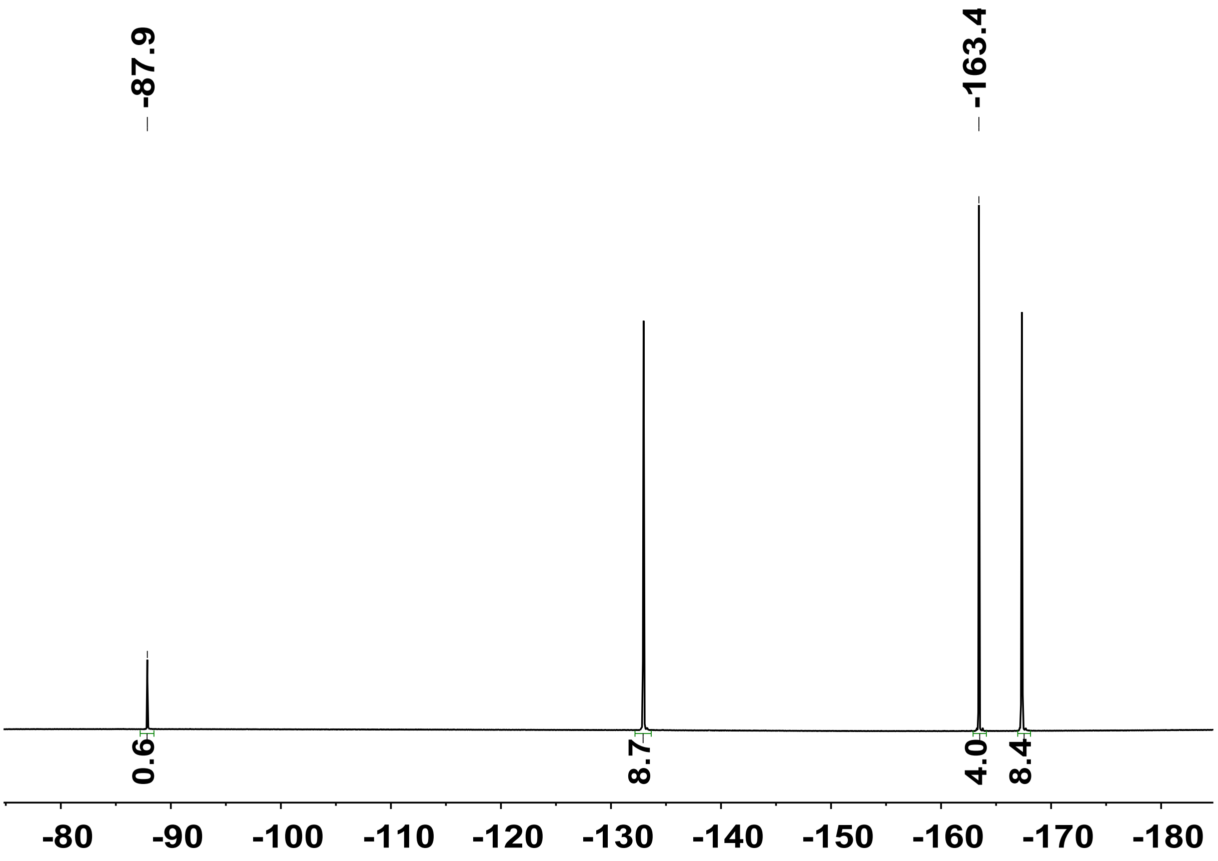

Figure S12 – ^{19}F{^{1}H} NMR spectrum (470.28 MHz, 305.1 K, CD_2Cl_2) of 10a[B(C_6F_5)$_4$].

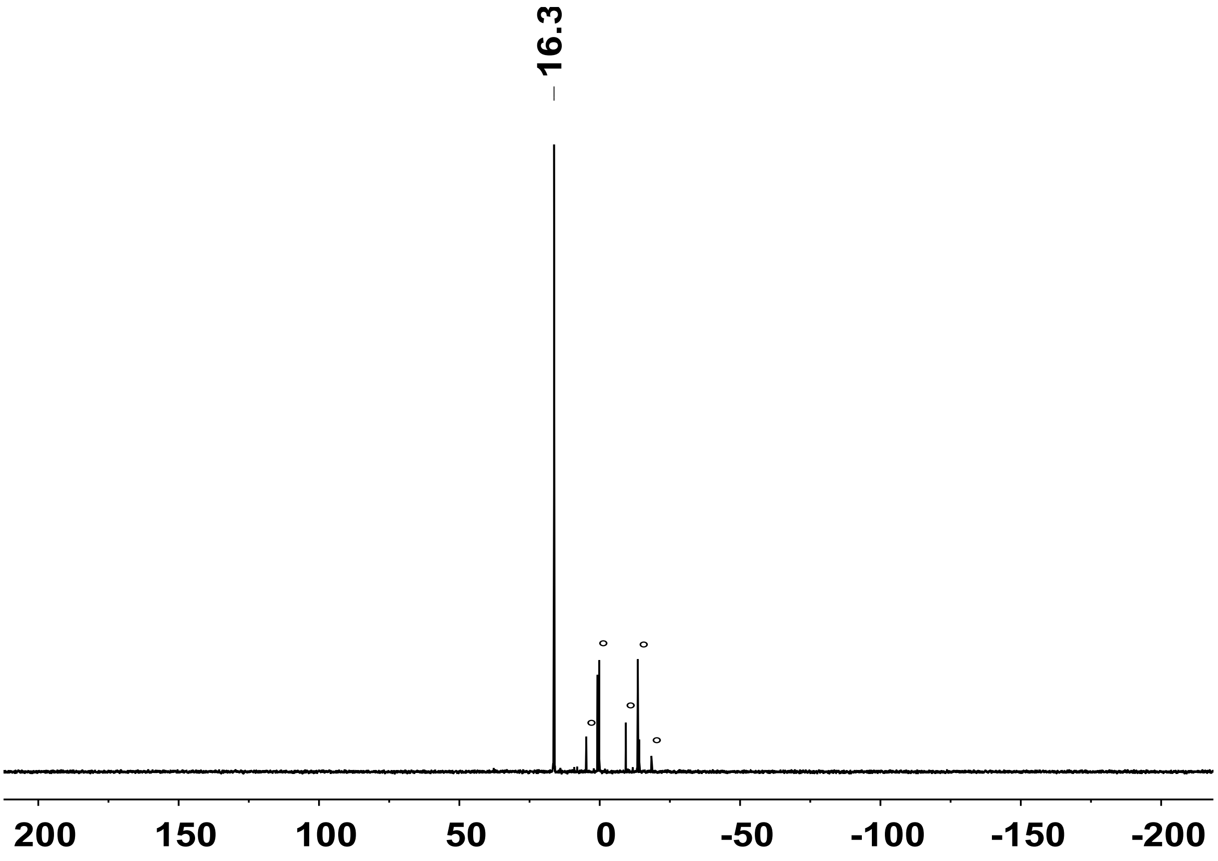

Figure S13 – ^{29}Si{^{1}H} INEPT NMR spectrum (99.31 MHz, 305.0 K, CD_2Cl_2, D3 = 0.0084, D4 = 0.0313, ° decomposition) of 10a[B(C_6F_5)$_4$].

Nitrilium borate **11a**[B(C_6F_5)$_4$] was synthesized according to general procedure **A** using 1.0 equiv. 8-phenoxy-1-dimethylsilylnaphthalene (251.41 µmol, 70 mg), 1.0 equiv. trityl borate (251.41 µmol, 230 mg) and 1.0 equiv. 4-fluorobenzonitrile (251.41 µmol, 30 mg).

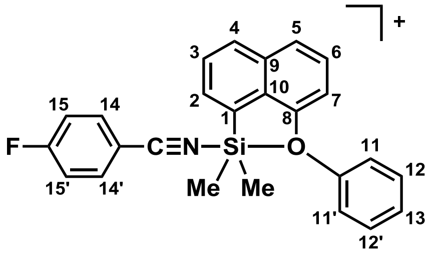

11a [B(C_6F_5)$_4$]$^-$

^{1}H NMR (499.87 MHz, 305.0 K, CD_2Cl_2) δ = 1.07 (s, 6 H, Si(CH_3)$_2$), 6.70 (d, $^3J_{H,H}$ = 7.8 Hz, 1 H, H-7), 7.35-7.37 (m, 2 H, H-11, H-11'), 7.39-7.43 (m, 3 H, H-15, H-15'), 7.47 (t, $^3J_{H,H}$ = 8.0 Hz, 1 H, H-6), 7.52 (t, $^3J_{H,H}$ = 7.5 Hz, 1 H, H-13), 7.65 (t, $^3J_{H,H}$ = 7.9 Hz, 2 H, H-12, H-12´), 7.70-7.76 (m, 2 H, H-4, H-5), 8.04-8.06 (m, 3 H, H-14, H-14'), 8.12 (d, $^3J_{H,H}$ = 7.0 Hz, 1 H, H-3), 8.18 (d, $^3J_{H,H}$ = 8.3 Hz, 1 H, H-2). **^{13}C{^{1}H} NMR** (125.71 MHz, 305.0 K, CD_2Cl_2) δ = 2.6 (Si(CH_3)$_2$), 103.1 (C-CN), 109.5 (C-7), 118.8 (d, $^2J_{C,F}$ = 23.3 Hz, C-15, C-15'), 120.9 (CN), 121.9 (C-1), 123.2 (C-11, C-11'), 123.7 – 125.7 (brm, [B(C_6F_5)$_4$], C), 124.3 (C-4/C-5), 127.4, 127.6, 128.4 (C-6, C-4/C-5, C-13), 128.9 (C-9/C-10), 131.8 (C-12, C-12'), 132.7 (C-2), 134.8 (C-3), 134.9 (C-9/C-10), 136.9 (d, $^1J_{C,F}$ = 243.5 Hz, [B(C_6F_5)$_4$], CF), 137.6 (d, $^3J_{C,F}$ = 10.2 Hz, C-14, C-14'), 138.9 (d, $^1J_{C,F}$ = 244.1 Hz, [B(C_6F_5)$_4$], CF), 148.8 (d, $^1J_{C,F}$ = 241.0 Hz, [B(C_6F_5)$_4$], CF), 152.3 (C-Ph), 154.4 (C-8), 168.3 (d, $^1J_{C,F}$ = 264.4 Hz, CF). **^{29}Si{^{1}H} NMR** (99.31 MHz, 305.0 K, CD_2Cl_2) δ = 1.6. **^{19}F{^{1}H} NMR** (470.30 MHz, 233.1 K, CD_2Cl_2) δ = -166.9-(-166.7) (m, 8 F, [B(C_6F_5)$_4$]), -162.87 (t, $^3J_{F,F}$ = 21.0 Hz, 4 F, [B(C_6F_5)$_4$]), -133.6-(-133.3) (m, 9 F, [B(C_6F_5)$_4$]), -102.6 (s, 0.5 F), -90.2 (s, 1 F, ^1J(CF) = 267.7 Hz). **^{11}B{^{1}H} NMR** (160.38 MHz, 305.1 K, CD_2Cl_2) δ = -16.6.

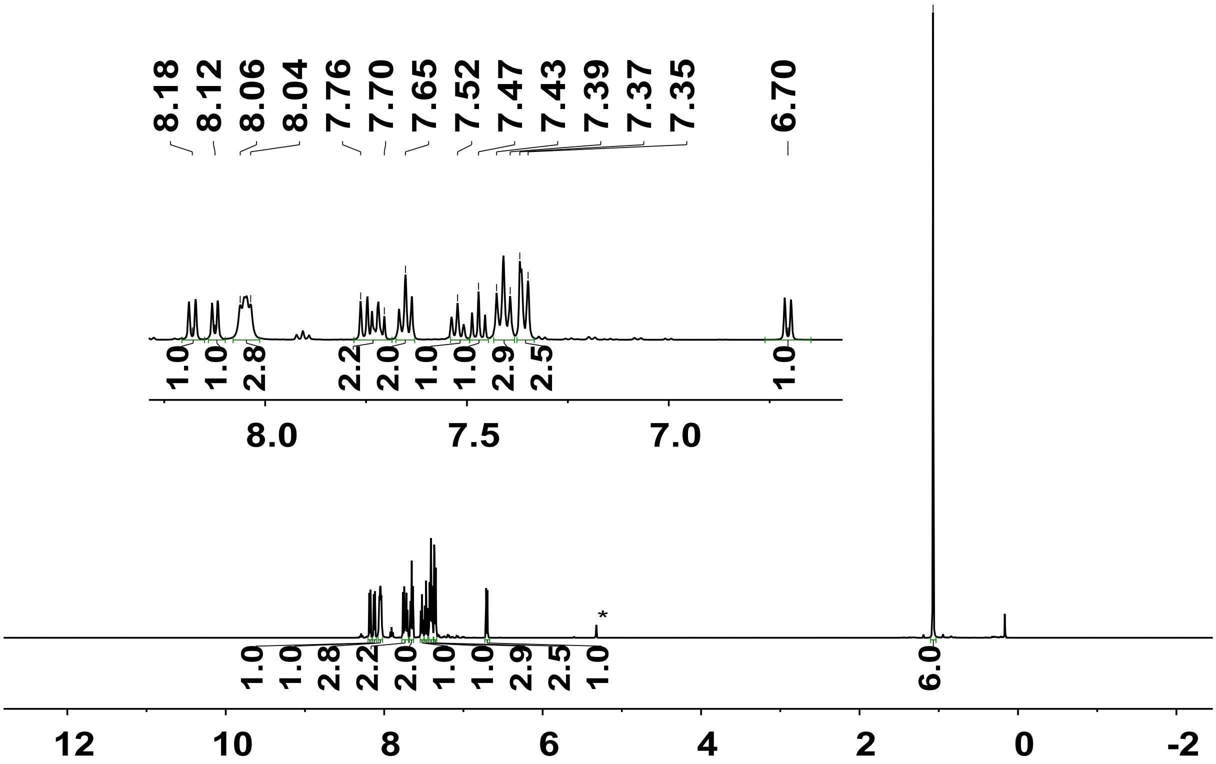

Figure S14 – ^{1}H NMR spectrum (499.87 MHz, 305.0 K, CD_2Cl_2, *$CDHCl_2$) of 11a[B(C_6F_5)$_4$].

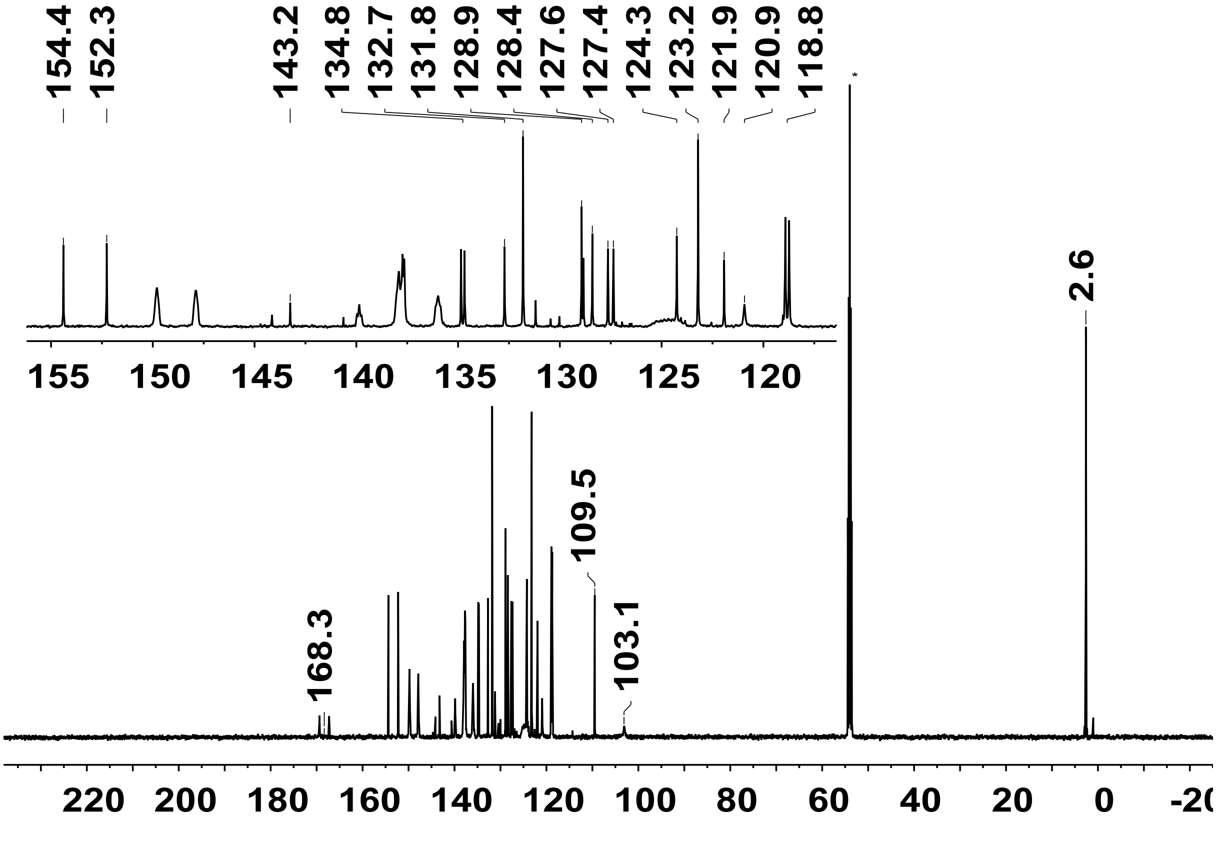

Figure S15 – ^{13}C{^{1}H} NMR spectrum (125.71 MHz, 305.0 K, *CD_2Cl_2) of 11a[B(C_6F_5)$_4$].

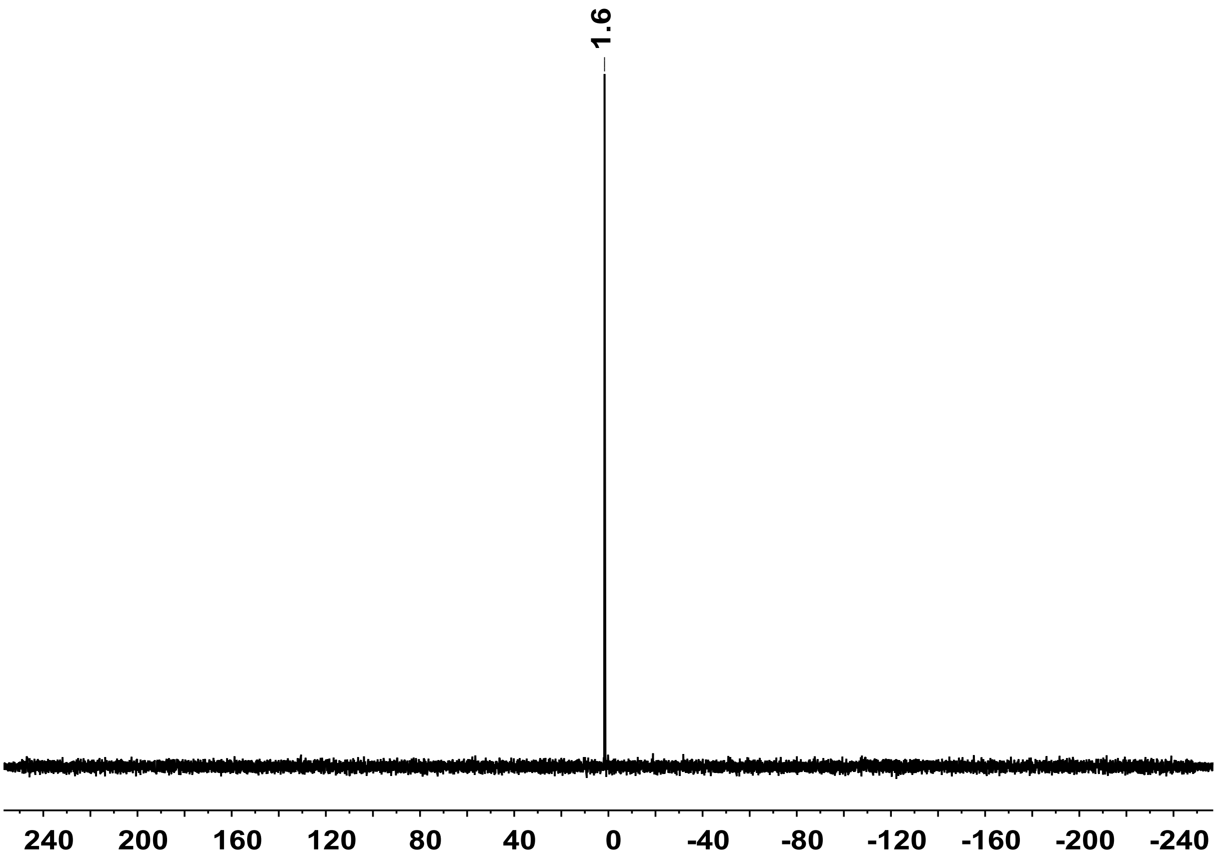

Figure S16 – $^{29}Si\{^1H\}$ NMR spectrum (99.31 MHz, 305.0 K, CD_2Cl_2) of 11a[B(C_6F_5)$_4$].

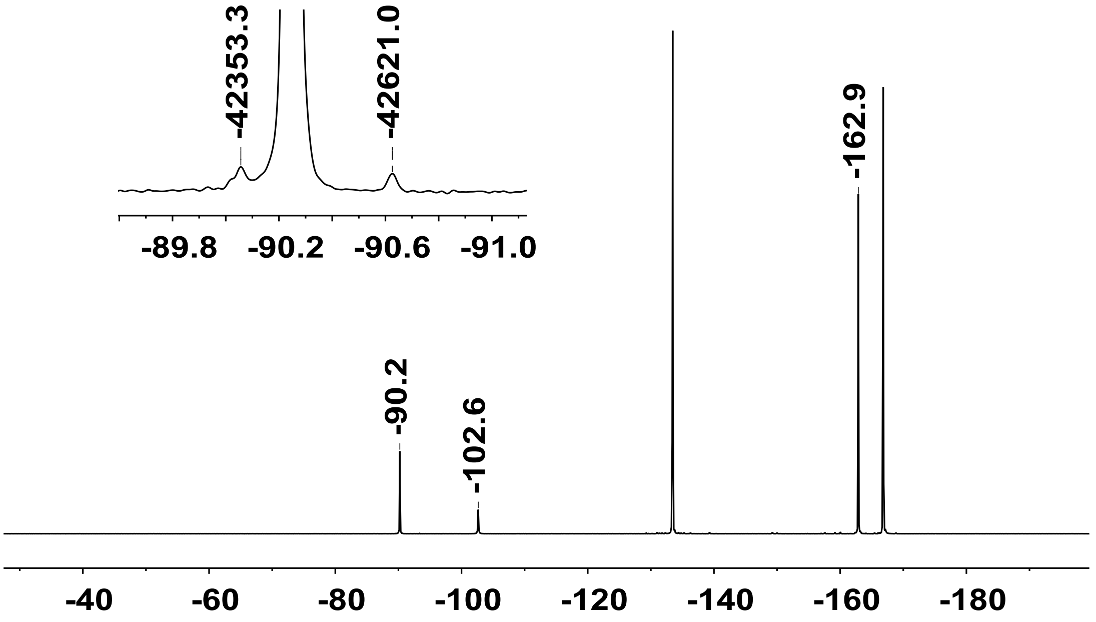

Figure S17 – $^{19}F\{^1H\}$ NMR spectrum (470.30 MHz, 233.1 K, CD_2Cl_2) of 11a[B(C_6F_5)$_4$].

Thiophenyl substituted derivative

The nitrilium borate **10b**[B(C_6F_5)$_4$] was synthesized according to general procedure **A** using 1.0 equiv. 6-dimethylsilyl-5-thiophenylacenaphthene (75 mg, 233.99 µmol), 1.0 equiv. trityl borate (215 mg, 233.99 µmol) and 1.0 equiv. 4-fluorobenzonitrile (28 mg, 233.99 µmol).

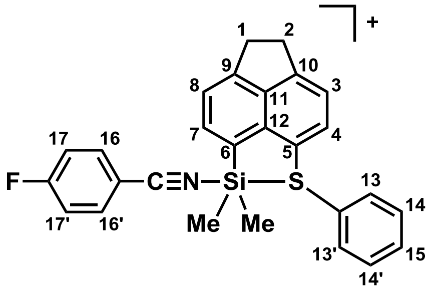

10b [B(C_6F_5)$_4$]$^-$

^{1}H NMR (499.87 MHz, 298.7 K, CD_2Cl_2): δ = 0.95 (s, 6 H, Si(CH_3)$_2$), 3.58-3.64 (m, 4 H, 2 × CH_2), 7.04-7.07 (m, 2 H, H-13, H13'), 7.38-7.43 (m, 4 H, H-14, H-14', H-17, H-17'), 7.44-7.48 (m, 1 H, H-15), 7.60-7.64 (m, 2 H, H-3, H-8), 7.95 (d, $^3J_{H,H}$ = 7.4 Hz, 1 H, H-4), 8.00-8.02 (m, 2 H, H-16, H-16'), 8.14 (d, $^3J_{H,H}$ = 7.1 Hz, 1 H, H-7). **^{13}C{^{1}H} NMR** (125.71 MHz, 298.7 K, CD_2Cl_2): δ = 2.7 (Si(CH_3)$_2$), 31.5 (CH_2), 31.7 (CH_2), 103.0 (<u>C</u>-CN), 118.8 (d, $^2J_{C,F}$ = 23.4 Hz, C-17, C-17'), 120.0, 120.1 (C, CN), 122.1 (C-6), 122.6, 122.9 (C-3, C-8), 128.8 (C-13, C-13´), 130.6 (C, C-Ph), 131.2 (C-14, C-14'), 135.8 (C-4), 136.9 (dm, $^1J_{C,F}$ = 243.0 Hz, CF, [B(C_6F_5)$_4$]$^-$), 137.6 (d, $^3J_{C,F}$ = 10.4 Hz, C-16, C-16'), 138.8 (C-7), 138.9 (dm, $^1J_{C,F}$ = 245.1 Hz, CF, [B(C_6F_5)$_4$]$^-$), 139.6 (C), 140.4 (C), 148.8 (dm, $^1J_{C,F}$ = 242.0 Hz, CF, [B(C_6F_5)$_4$]$^-$), 153.2 (C), 153.6 (C), 168.3 (d, $^1J_{C,F}$ = 264.0 Hz, CF). **^{19}F{^{1}H} NMR** (470.28 MHz, 298.7 K, CD_2Cl_2): δ = -167.4-(-167.2) (m, 8 F, *m*-F, [B(C_6F_5)$_4$]$^-$), -163.44 (t, $^3J_{F,F}$ = 20.5 Hz, 4 F, *p*-F, [B(C_6F_5)$_4$]$^-$), -132.9-(-132.7) (m, 8 F, *o*-F, [B(C_6F_5)$_4$]$^-$), -94.3 (s, 1 F, CF). **^{29}Si{^{1}H} NMR** (99.31 MHz, 298.7 K, CD_2Cl_2): δ = 29.5. **^{11}B{^{1}H} NMR** (160.38 MHz, 305.0 K, CD_2Cl_2): δ = -16.6.

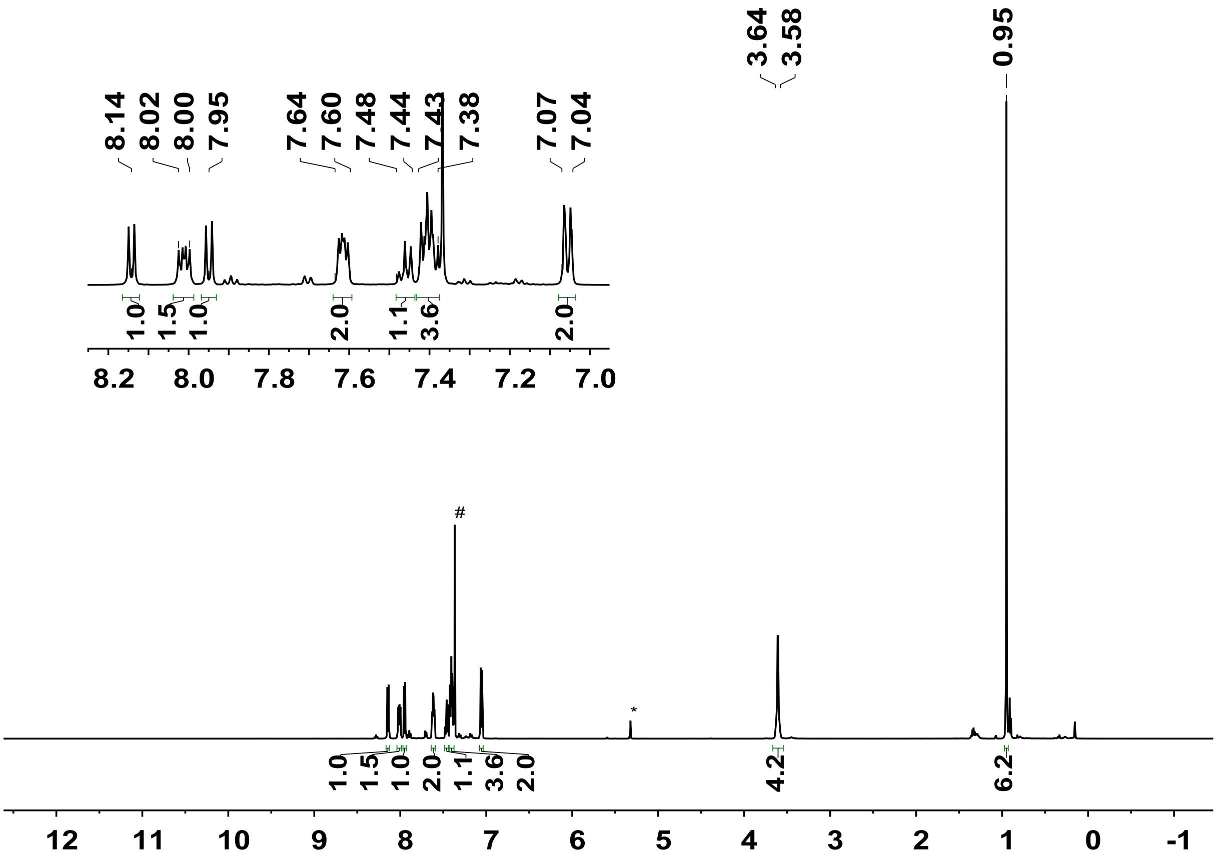

Figure S18 – ^{1}H NMR spectrum (499.87 MHz, 305.0 K, CD_2Cl_2, *$CDHCl_2$, $^{\#}C_6H_6$) of 10b[B(C_6F_5)$_4$].

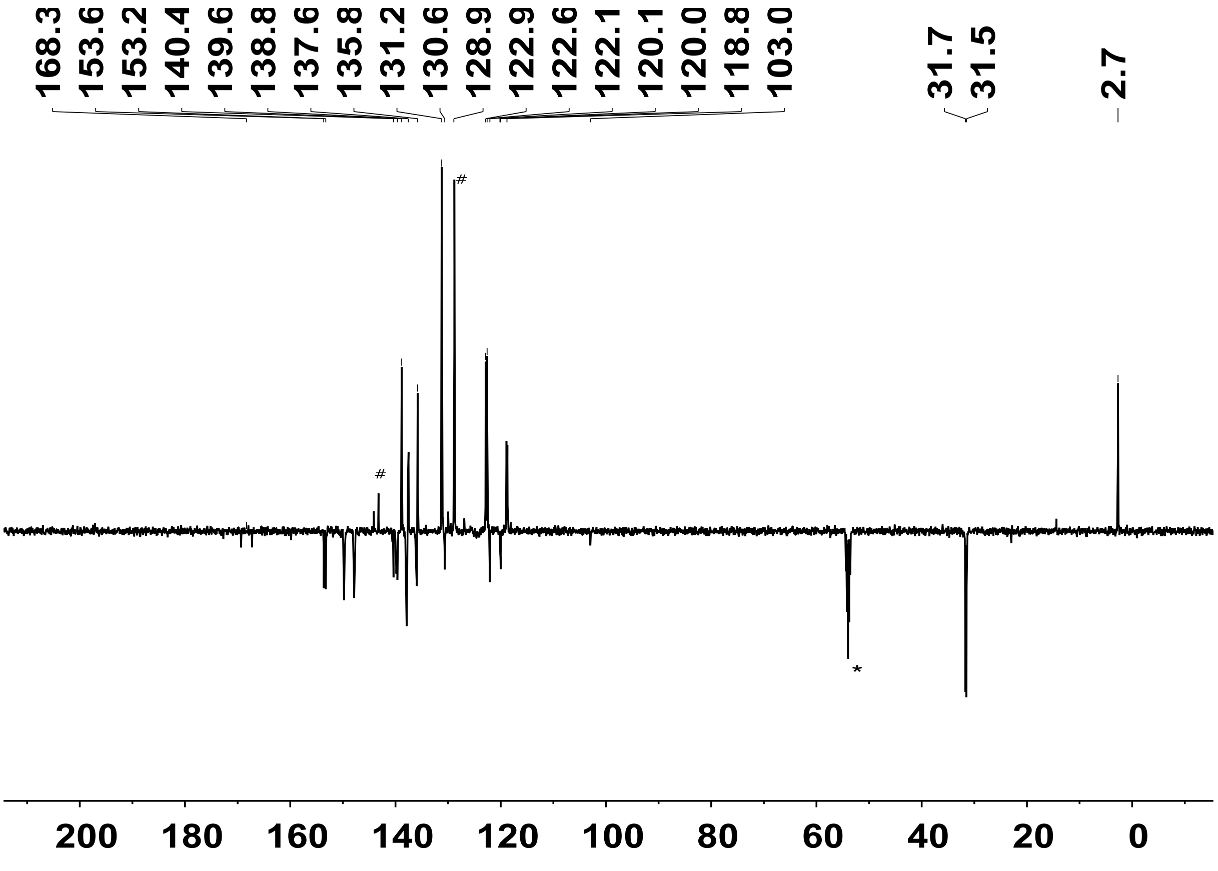

Figure S19 – ^{13}C{^{1}H} APT NMR spectrum (125.71 MHz, 305.0 K, *CD_2Cl_2, # C_6H_6, impurities) of 10b[B(C_6F_5)$_4$].

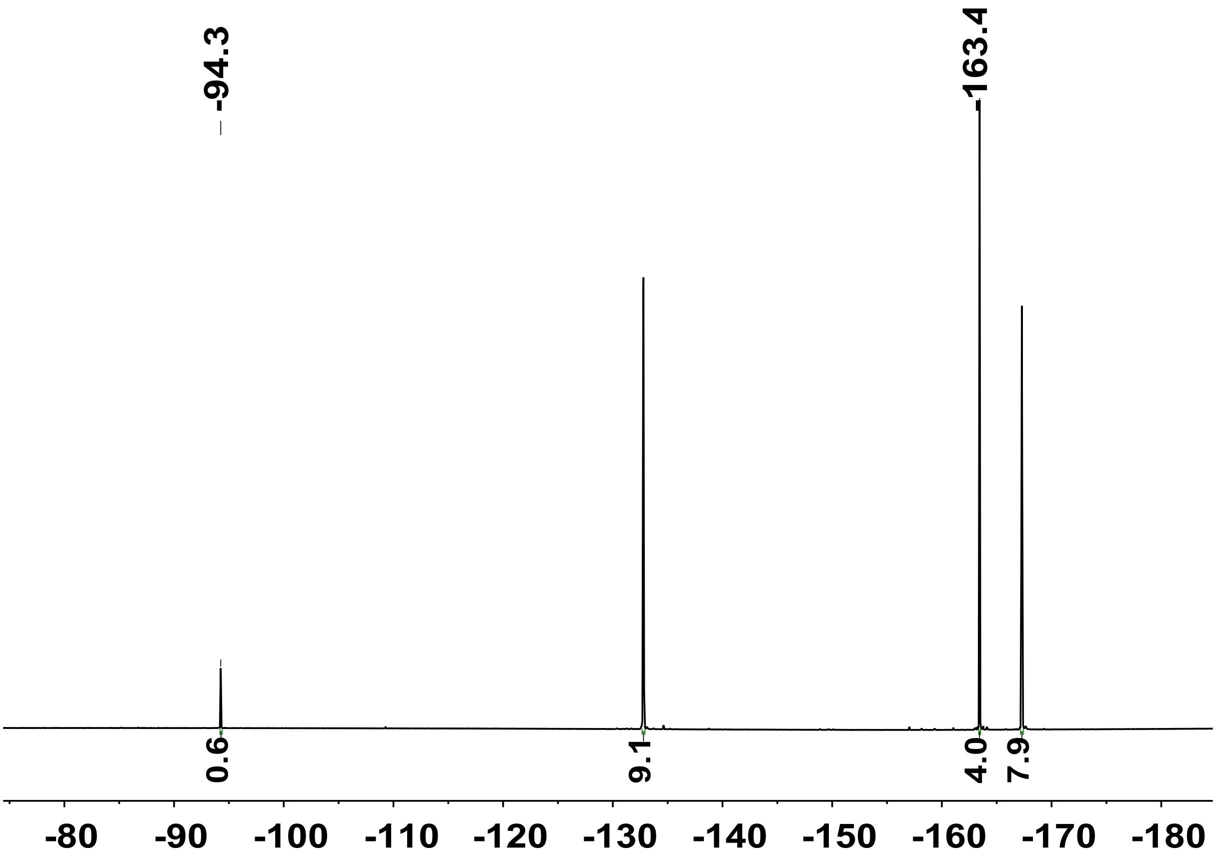

Figure S20 – $^{19}F\{^{1}H\}$ NMR spectrum (470.30 MHz, 305.0 K, CD_2Cl_2) of 10b[B(C_6F_5)$_4$].

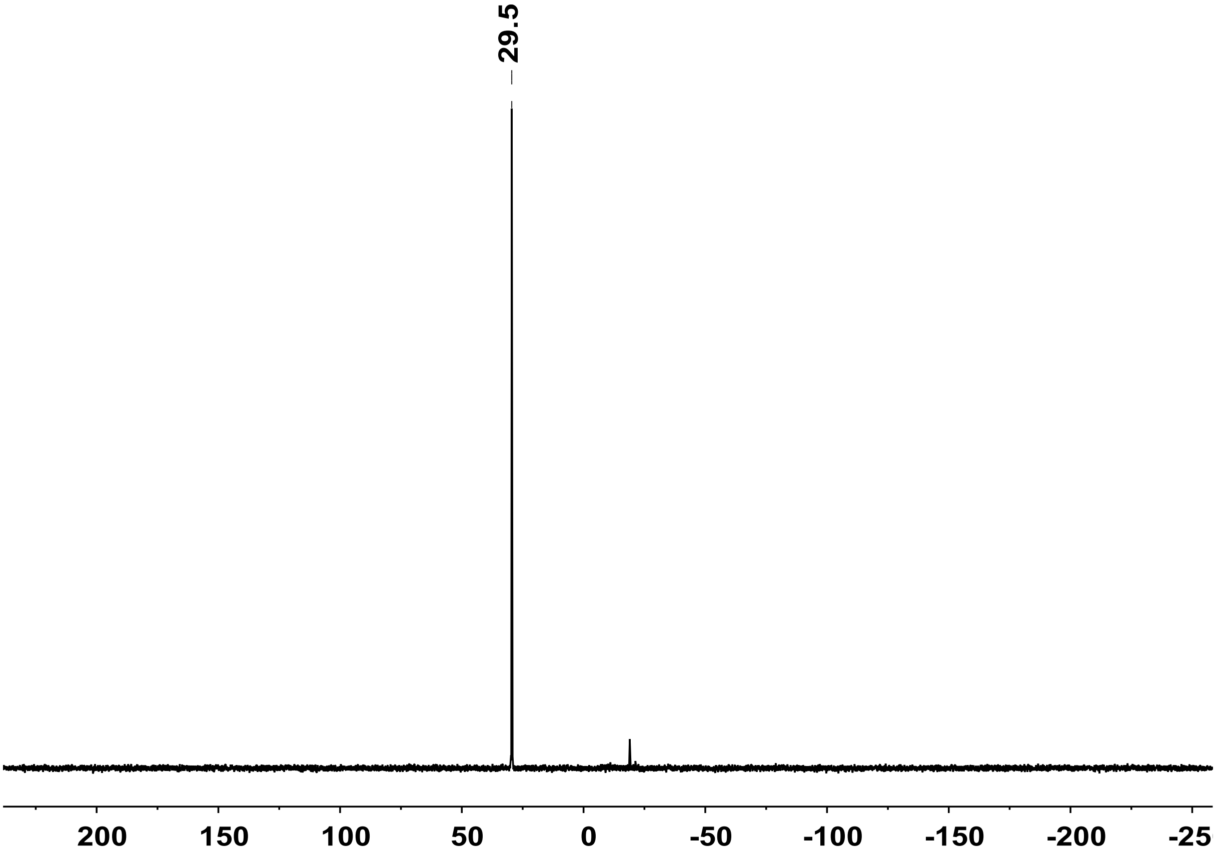

Figure S21 – $^{29}Si\{^{1}H\}$ INEPT NMR spectrum (99.31 MHz, 305.0 K, CD_2Cl_2) of 10b[B(C_6F_5)$_4$].

Selenylphenyl substituted derivative

The nitrilium ion **10c**[B(C_6F_5)$_4$] was synthesized according to general procedure **C** using 1.0 equiv. 5-phenylselenyl-6-dimethylsilylacenaphthene **7c** (147 mg, 400 µmol), 1.0 equiv. trityl borate (369 mg, 400 µmol) and 1.0 equiv. 4-fluorobenzonitrile (49 mg, 400 µmol).

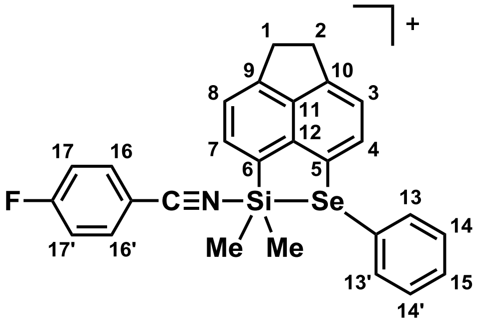

10c [B(C_6F_5)$_4$]$^-$

^{1}H NMR (499.87 MHz, 305.1 K, CD_2Cl_2): δ = 1.08 (s, 6 H, Si(CH_3)$_2$), 3.59-3.65 (m, 4 H, 2 × CH_2), 7.07 (d, $^3J_{H,H}$ = 7.7 Hz, 2 H, H-13, H13'), 7.29-7.35 (m, 3 H, H-17, H-17'), 7.35-7.38 (m, 2 H, H-14, H-14'), 7.44 (t, $^3J_{H,H}$ = 7.3 Hz, 1 H, H-15), 7.59-7.64 (m, 2 H, H-3, H-8), 7.86-7.92 (m, 3 H, H-16, H-16'), 8.05 (d, $^3J_{H,H}$ = 7.3 Hz, 1 H, H-4), 8.21 (d, $^3J_{H,H}$ = 7.1 Hz, 1 H, H-7). **^{13}C{^{1}H} NMR** (125.71 MHz, 305.0 K, CD_2Cl_2): δ = 3.5 ($^1J_{C,Si}$ = 64.6 Hz, $^2J_{C,Se}$ = 15.0 Hz, Si(CH_3)$_2$), 31.4 (CH_2), 31.5 (CH_2), 105.8 (d, $^4J_{C,F}$ = 3.5 Hz, C-CN), 118.1 (d, $^2J_{C,F}$ = 23.1 Hz, C-17, C-17'), 119.6 (CN), 120.6 ($^1J_{C,Se}$ = 84.7 Hz, C-5), 122.4, 122.9 (C-3, C-8), 123.9-125.8 (brs, C, [B(C_6F_5)$_4$]$^-$), 124.9 (C-6), 129.1 (C, C-Ph), 129.5 (C-13, C-13'), 130.7 (C-15), 131.4 (C-14, C-14'), 136.6 (d, $^3J_{C,F}$ = 10.0 Hz, C-16, C-16'), 137.1 (dm, $^1J_{C,F}$ = 241.9 Hz, CF, [B(C_6F_5)$_4$]$^-$), 137.2 (C-4), 139.0 (dm, $^1J_{C,F}$ = 245.8 Hz, CF, [B(C_6F_5)$_4$]$^-$), 139.3 (C-7), 140.5, 141.0 (C-11, C-12), 149.0 (d, $^1J_{C,F}$ = 240.9 Hz, CF, [B(C_6F_5)$_4$]$^-$), 153.3, 153.4 (C-9, C-10), 167.2 (d, $^1J_{C,F}$ = 260.2 Hz, CF). **^{19}F{^{1}H} NMR** (470.28 MHz, 305.0 K, CD_2Cl_2): δ = -167.4-167.2 (m, 8 F, *m*-F, [B(C_6F_5)$_4$]$^-$), -163.44 (t, $^3J_{F,F}$ = 20.5 Hz, 4 F, *p*-F, [B(C_6F_5)$_4$]$^-$), -132.9-(-132.7) (m, 8 F, *o*-F, [B(C_6F_5)$_4$]$^-$), -98.5 (s, 1 F, CF). **^{29}Si{^{1}H} NMR** (99.31 MHz, 305.0 K, CD_2Cl_2): δ = 25.5 ($^1J_{Si,Se}$ = 33 Hz). **^{77}Se{^{1}H} NMR** (95.36 MHz, 305.0 K, CD_2Cl_2): δ = 279.3. **^{11}B{^{1}H} NMR** (160.38 MHz, 305.0 K, CD_2Cl_2): δ = -16.4.

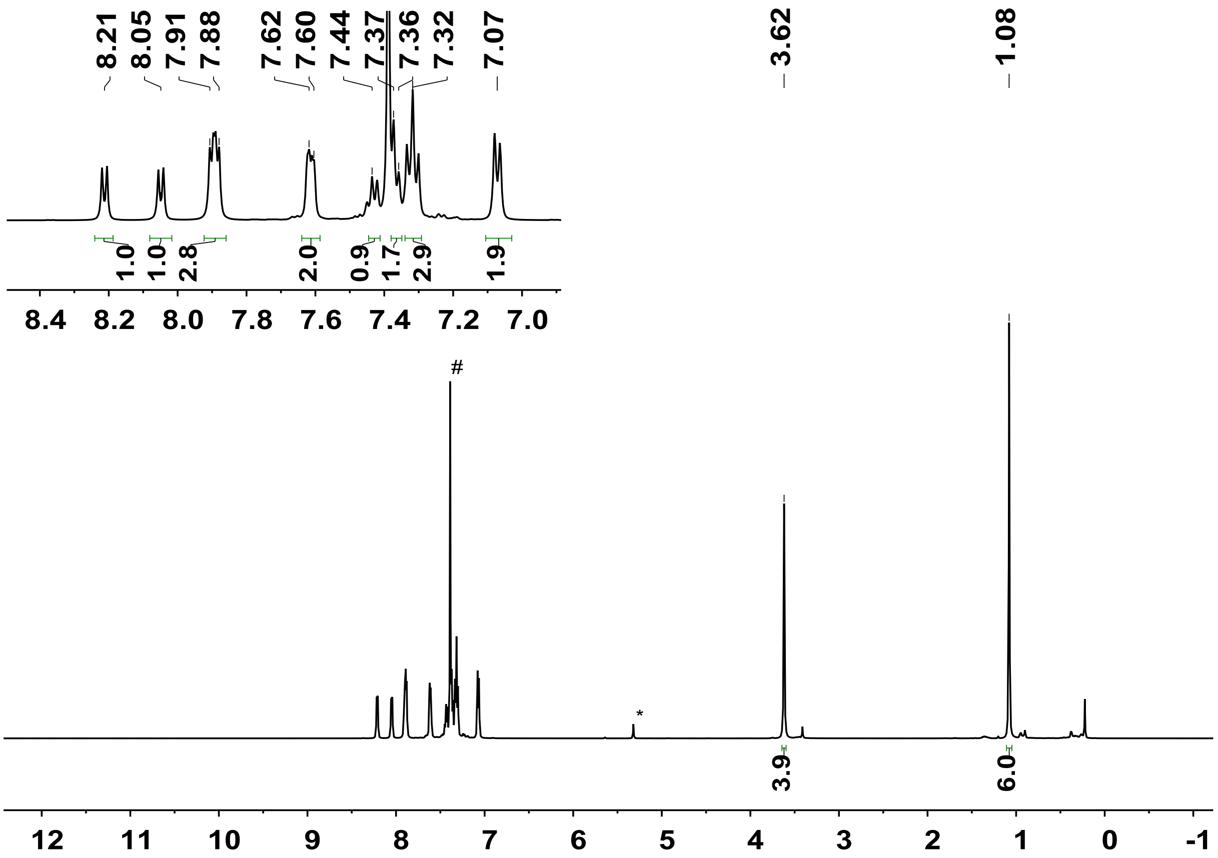

Figure S22 – ^{1}H NMR spectrum (499.87 MHz, 305.1 K, CD_2Cl_2, *$CDHCl_2$, #C_6H_6) of 10c[B(C_6F_5)$_4$].

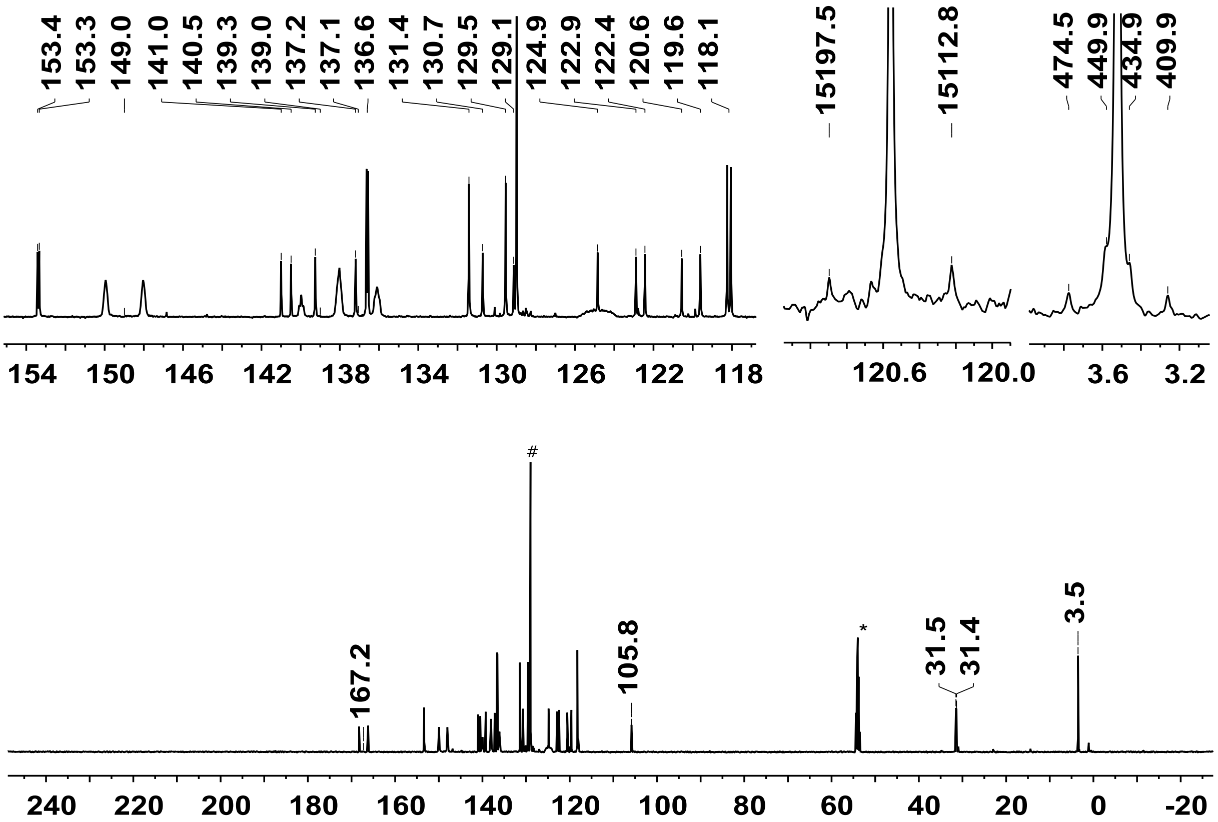

Figure S23 – ^{13}C{^{1}H} NMR spectrum (125.71 MHz, 305.0 K, *CD_2Cl_2, #C_6H_6) of 10c[B(C_6F_5)$_4$].

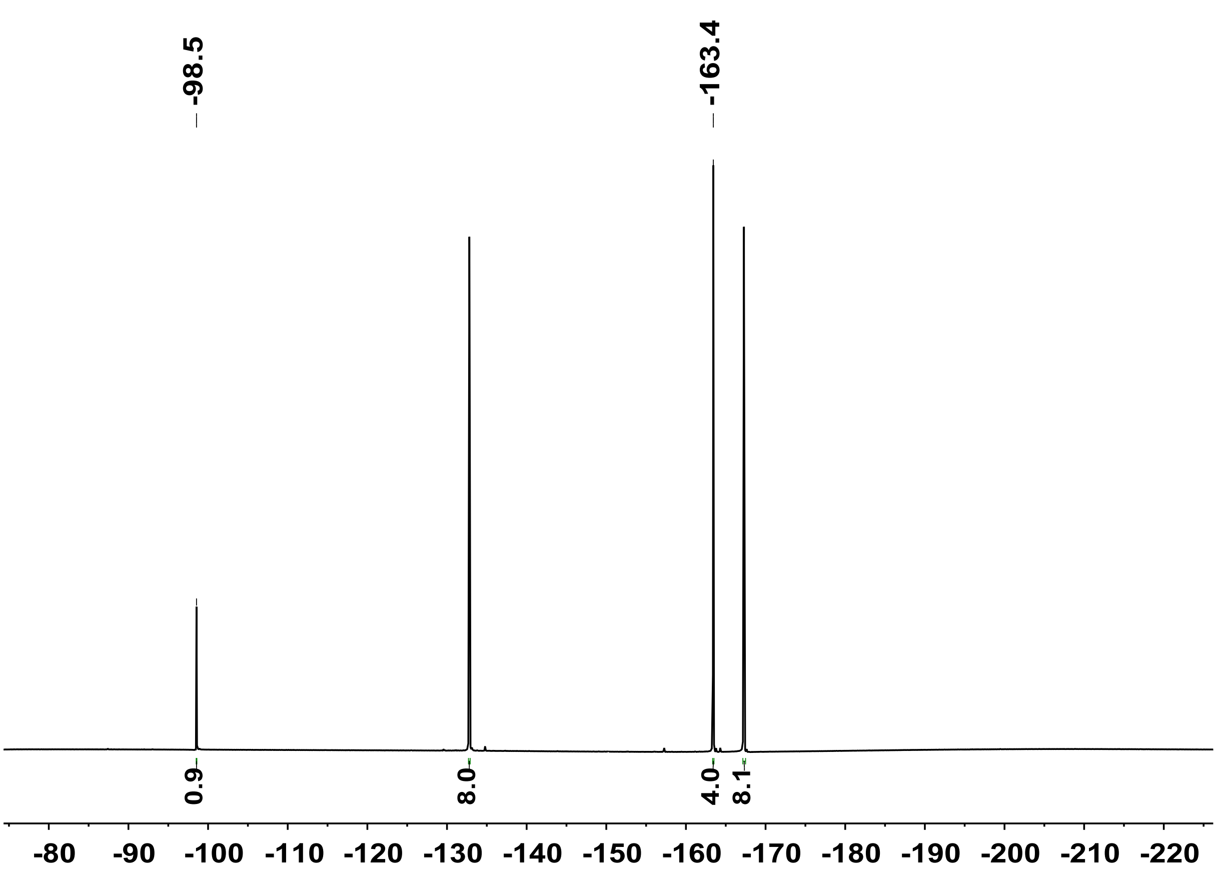

Figure S24 – $^{19}F\{^1H\}$ NMR spectrum (470.28 MHz, 305.0 K, CD_2Cl_2) of 10c[B(C_6F_5)$_4$].

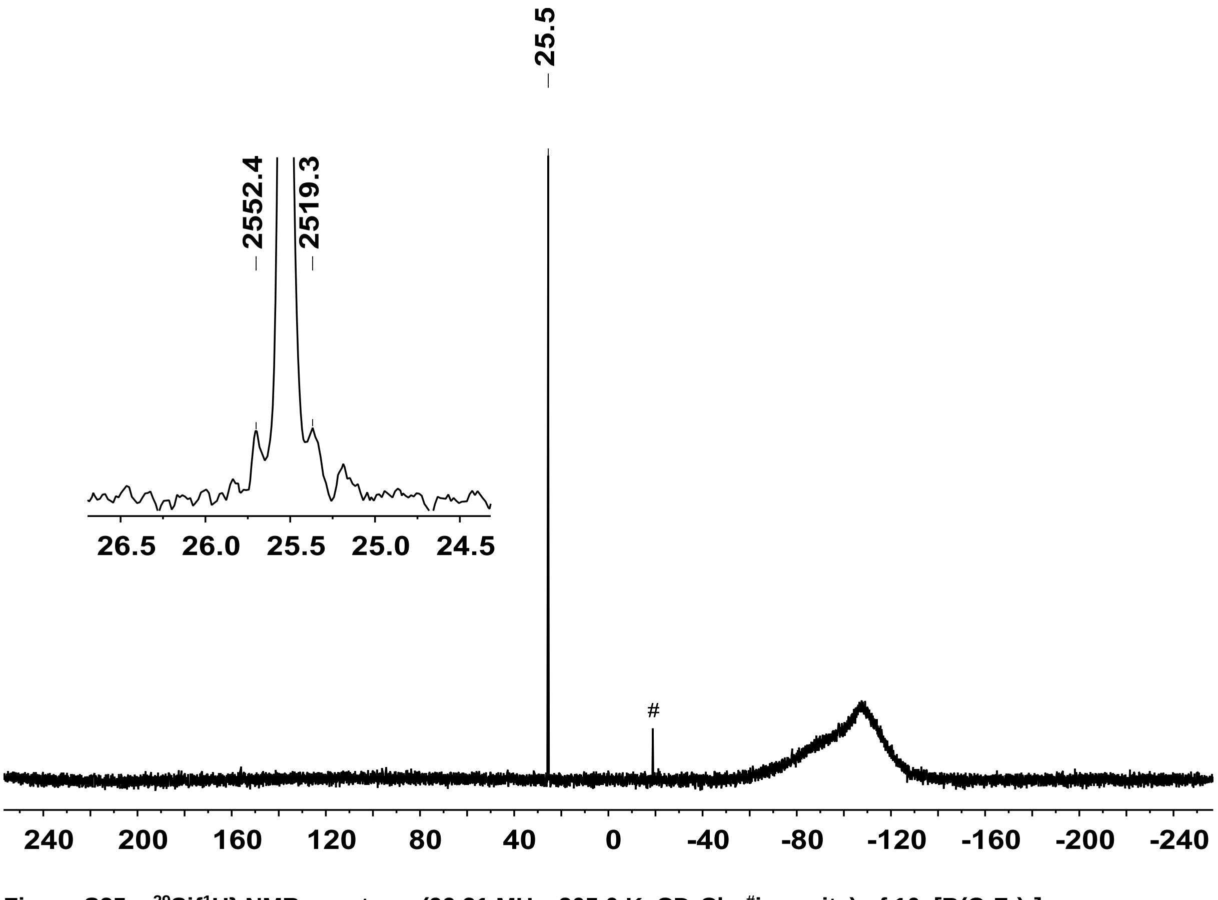

Figure S25 – $^{29}Si\{^1H\}$ NMR spectrum (99.31 MHz, 305.0 K, CD_2Cl_2, #impurity) of 10c[B(C_6F_5)$_4$].

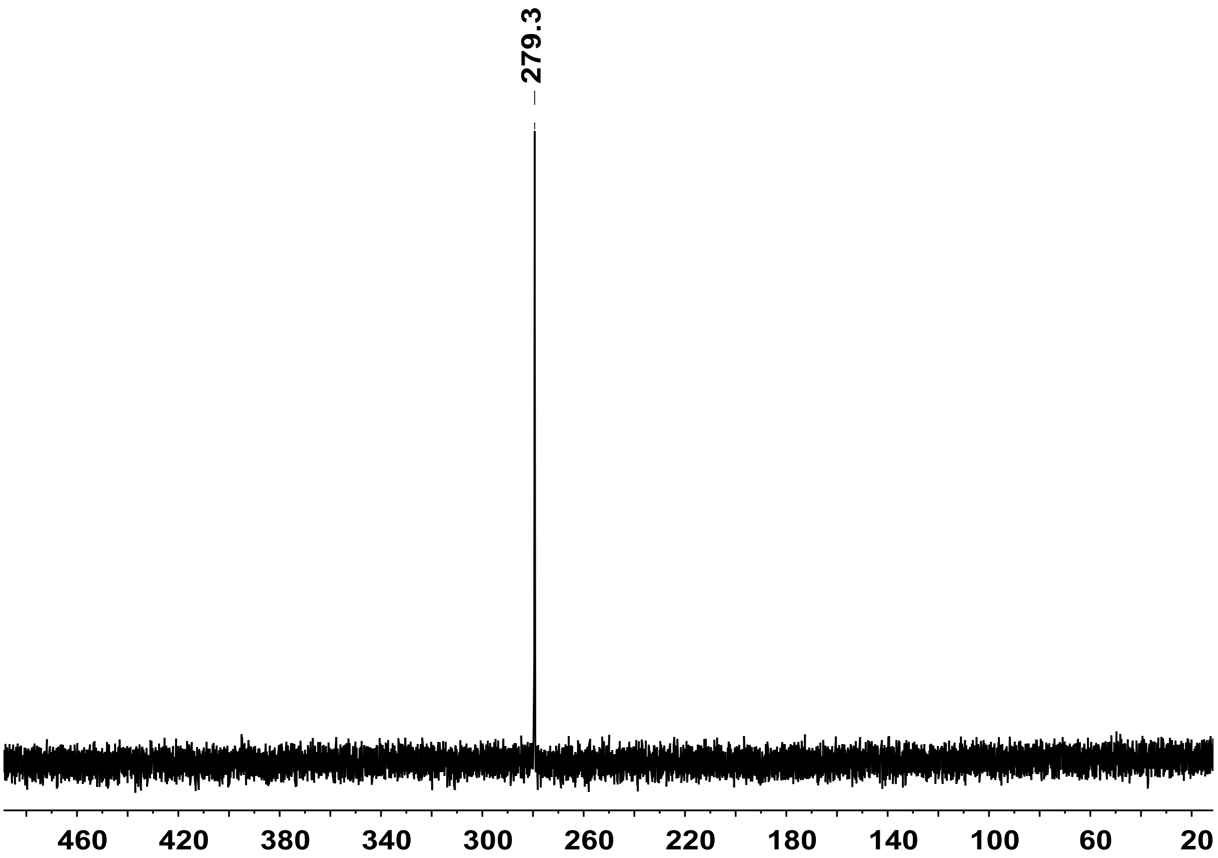

Figure S26 – ^{77}Se{^{1}H} NMR spectrum (95.36 MHz, 305.0 K, CD_2Cl_2) of 10c[$B(C_6F_5)_4$].

One Schlenk tube was charged with 1.0 equiv. 1-phenylselenyl-8-dimethylsilylnaphthalene **8c** (120 mg, 351 µmol). In another Schlenk tube 1.0 equiv. trityl borate [Ph_3C][$B(C_6F_5)_4$] (324 mg, 351 µmol) was dissolved in benzene. This solution was added to silane **8c**. The resulting mixture was stirred for one hour. After that the nonpolar phase was removed. The polar phase was washed with a mixture of benzene and pentane and then with pentane. After removing the solvent under reduced pressure, the residue was dissolved in dichloromethane-d_2. 0.9 equiv. 4-fluorobenzonitrile (38 mg, 316 µmol) were added. The reaction mixture was stirred for 15 min and after that analyzed by NMR spectroscopy.

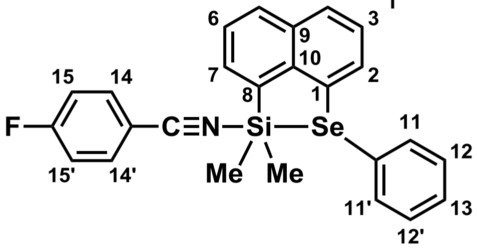

11c [$B(C_6F_5)_4$]$^-$

^{1}H NMR (499.87 MHz, 305.0 K, CD_2Cl_2): δ = 0.98 (s, 6 H, $Si(CH_3)_2$), 7.08-7.11 (m, 2 H, H-11, H-11’), 7.22-7.27 (m, 2 H, H-15, H-15’), 7.43-7.47 (m, 2 H, H-12, H-12’), 7.53-7.57 (m, H-13), 7.74-7.78 (m, 2 H, H-14, H-14’), 7.82-7.89 (m, 2 H, H-3, H-6), 8.14 (dd, $^{3}J_{H,H}$ = 7.4 Hz, $^{4}J_{H,H}$ = 0.8 Hz, 1 H, H-7), 8.17 (dd, $^{3}J_{H,H}$ = 6.9 Hz, $^{4}J_{H,H}$ = 0.8 Hz, 1 H, H-2), 8.29 (d, $^{3}J_{H,H}$ = 7.8 Hz, 1 H, H-5), 8.37 (d, $^{3}J_{H,H}$ = 7.8 Hz, 1 H, H-4). **^{13}C{^{1}H} NMR** (125.71 MHz, 305.0 K, CD_2Cl_2): δ = 0.3 ($Si(CH_3)_2$), 108.1 (d, $^{4}J_{C,F}$ = 2.1 Hz, <u>C</u>-CN), 117.6 (d, $^{2}J_{C,F}$ = 23.0 Hz, C-15, C-15’), 119.0 (CN), 124.0-125.5 (brs, C, $[B(C_6F_5)_4]^-$), 125.4 (C, C-Ph), 126.9 (C-9), 129.3 (C-3, C-6), 129.9 (C-11, C-11’), 131.8 (C-1/C-8), 132.2 (C-12, C-12’), 132.5 (C-13), 133.2 (C-5), 134.4 (C-4), 134.7 (C-2), 135.5 (C-1/C-8), 135.7 (d, $^{3}J_{C,F}$ = 9.5 Hz, C-14, C-14’), 136.9 (C-7), 137.1 (dm, $^{1}J_{C,F}$ = 242.1 Hz, CF, $[B(C_6F_5)_4]^-$), 139.0 (dm, $^{1}J_{C,F}$ = 243.9 Hz, CF, $[B(C_6F_5)_4]^-$), 140.2 (C-10), 166.3 (d, $^{1}J_{C,F}$ = 257.2 Hz, CF). **^{19}F{^{1}H} NMR** (470.29 MHz, 305.1 K, CD_2Cl_2): δ = -167.4-(-167.2) (m, 8 F, *m*-F, $[B(C_6F_5)_4]^-$), -163.44 (t, $^{3}J_{F,F}$ = 20.3 Hz, 4 F, *p*-F, $[B(C_6F_5)_4]^-$), -132.9-(-132.7) (m, 8 F, *o*-F, $[B(C_6F_5)_4]^-$), -101.9 (s, 1 F, CF). **^{29}Si{^{1}H} NMR** (99.31 MHz, 305.0 K, CD_2Cl_2): δ = 49.2 ($^{1}J_{Si,Se}$ = 56 Hz). **^{77}Se{^{1}H} NMR** (95.36 MHz, 305.0 K, CD_2Cl_2): δ = 254.8 ($^{1}J_{Se,Si}$ = 56 Hz). **^{11}B{^{1}H} NMR** (160.38 MHz, 305.1 K, CD_2Cl_2): δ = -16.5.

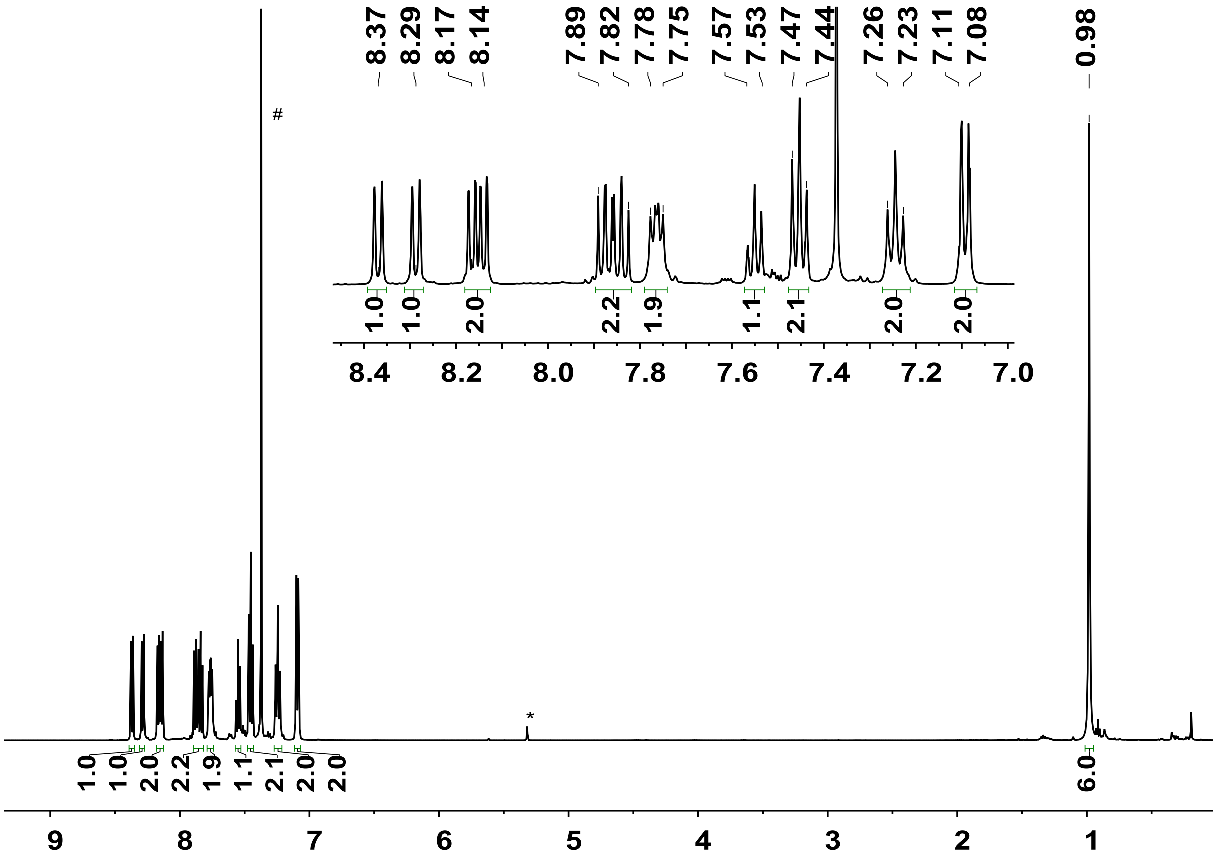

Figure S27 – ^{1}H NMR spectrum (499.87 MHz, 305.0 K, CD_2Cl_2, *$CDHCl_2$, $^{\#}C_6H_6$) of 11c$[B(C_6F_5)_4]$.

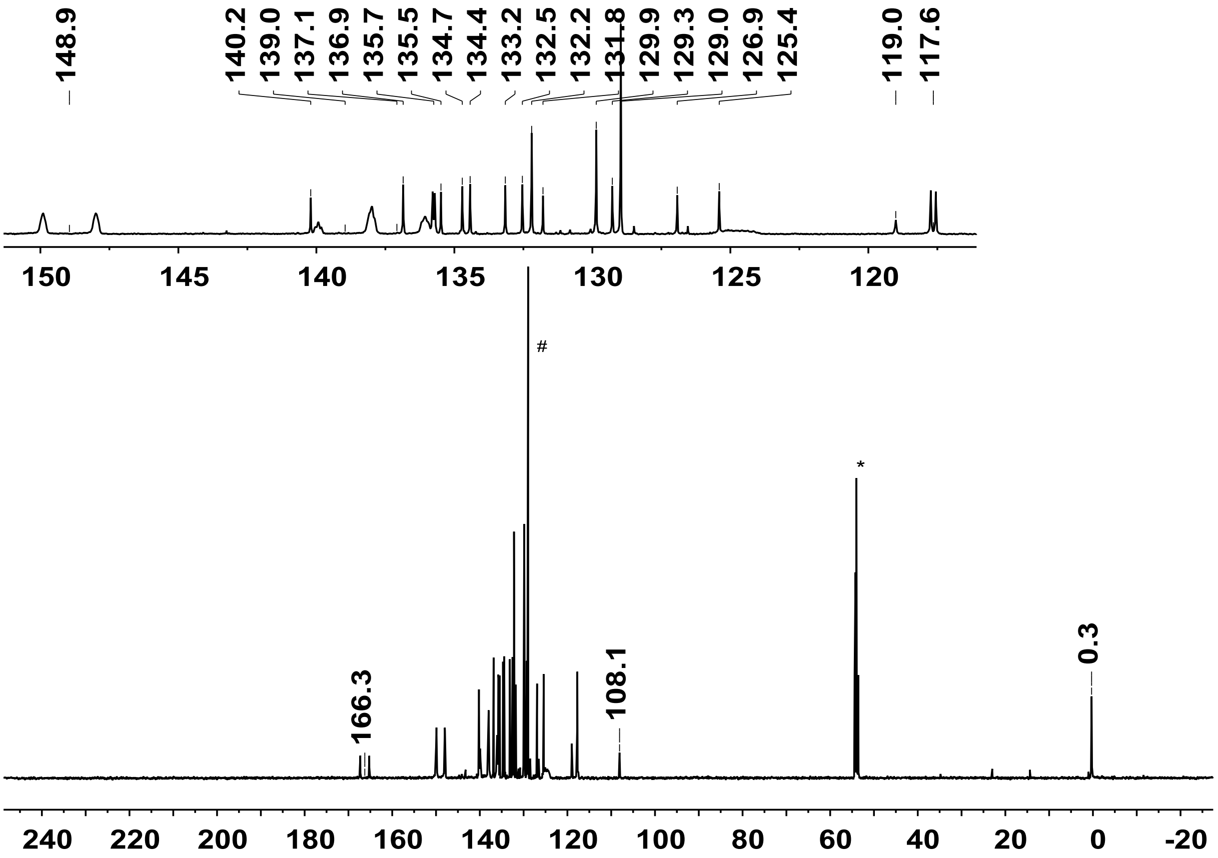

Figure S28 – $^{13}C\{^1H\}$ NMR spectrum (125.71 MHz, 305.0 K, *CD_2Cl_2, #C_6H_6) of 11c[$B(C_6F_5)_4$].

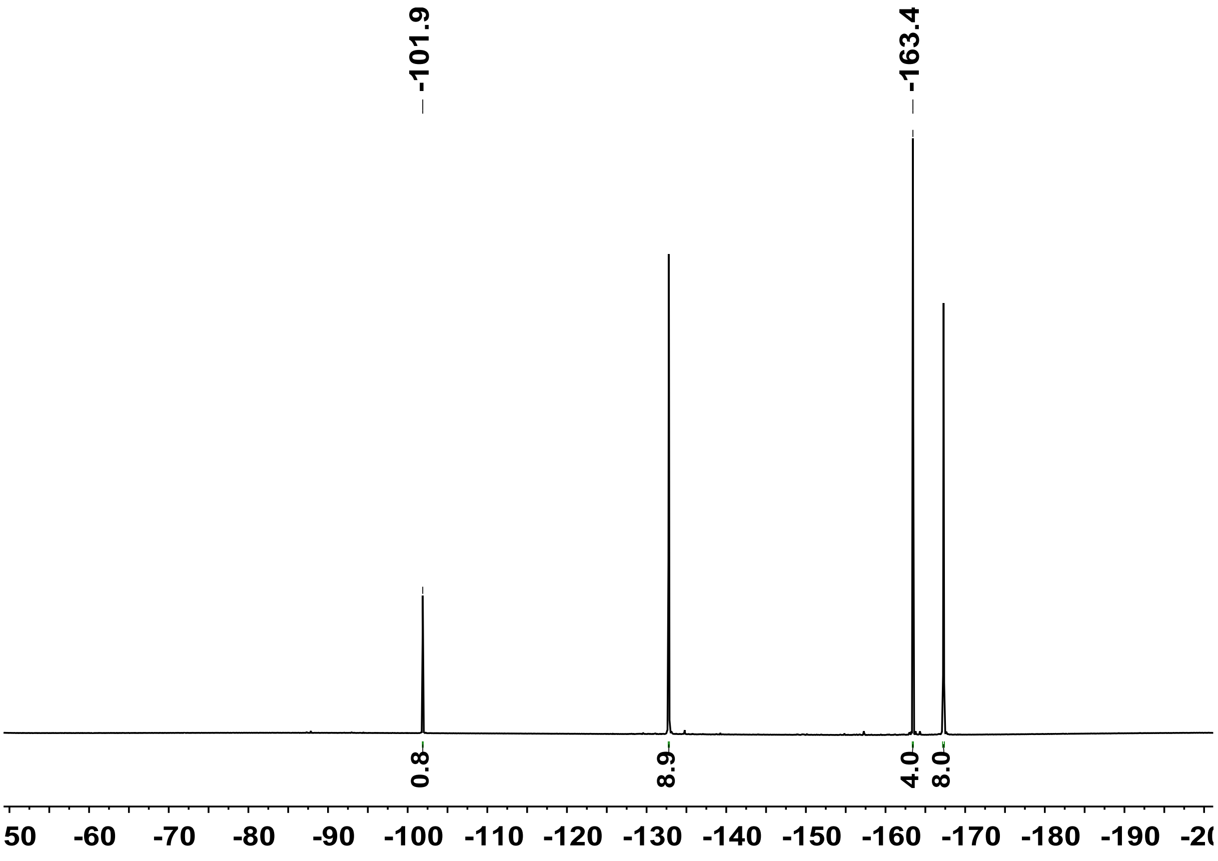

Figure S29 – $^{19}F\{^1H\}$ NMR spectrum (470.29 MHz, 305.1 K, CD_2Cl_2) of 11c[$B(C_6F_5)_4$].

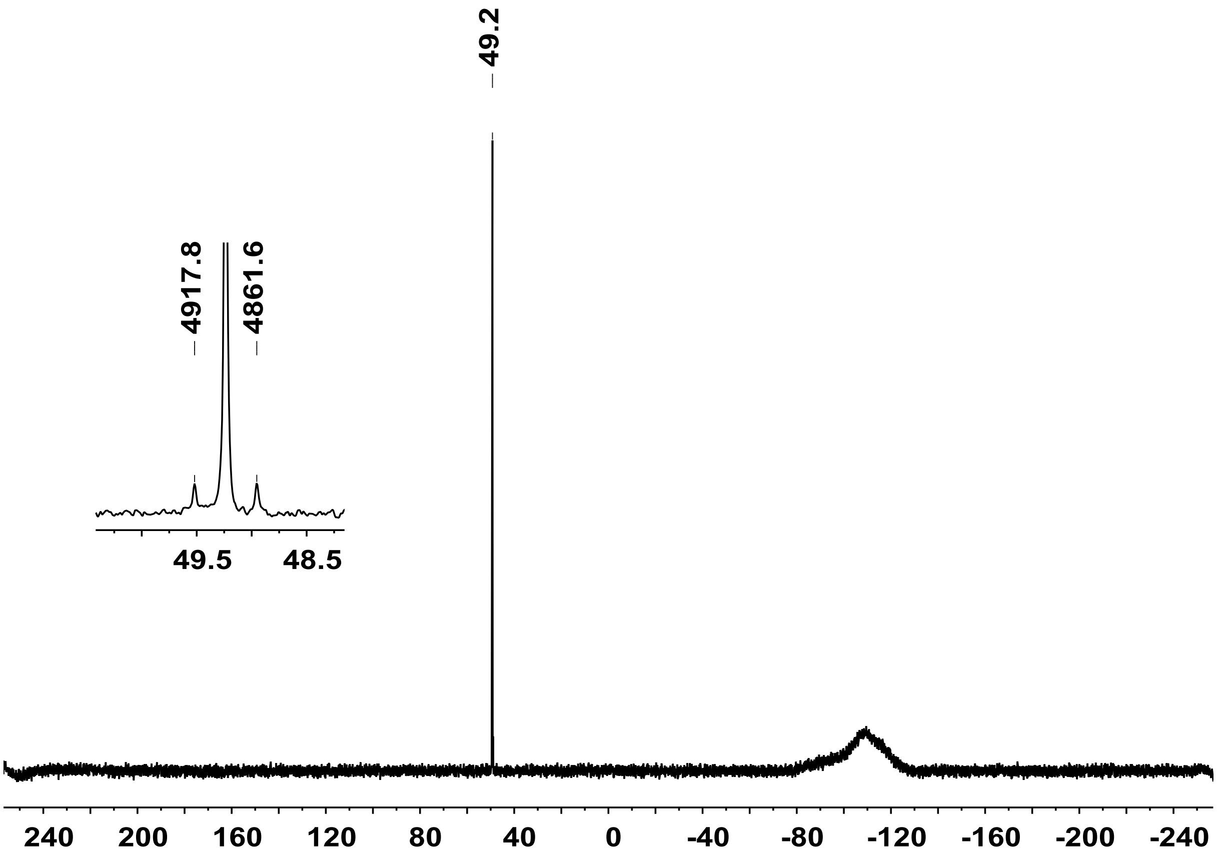

Figure S30 – $^{29}Si\{^1H\}$ NMR spectrum (99.31 MHz, 305.0 K, CD_2Cl_2) of 11c[B(C_6F_5)$_4$].

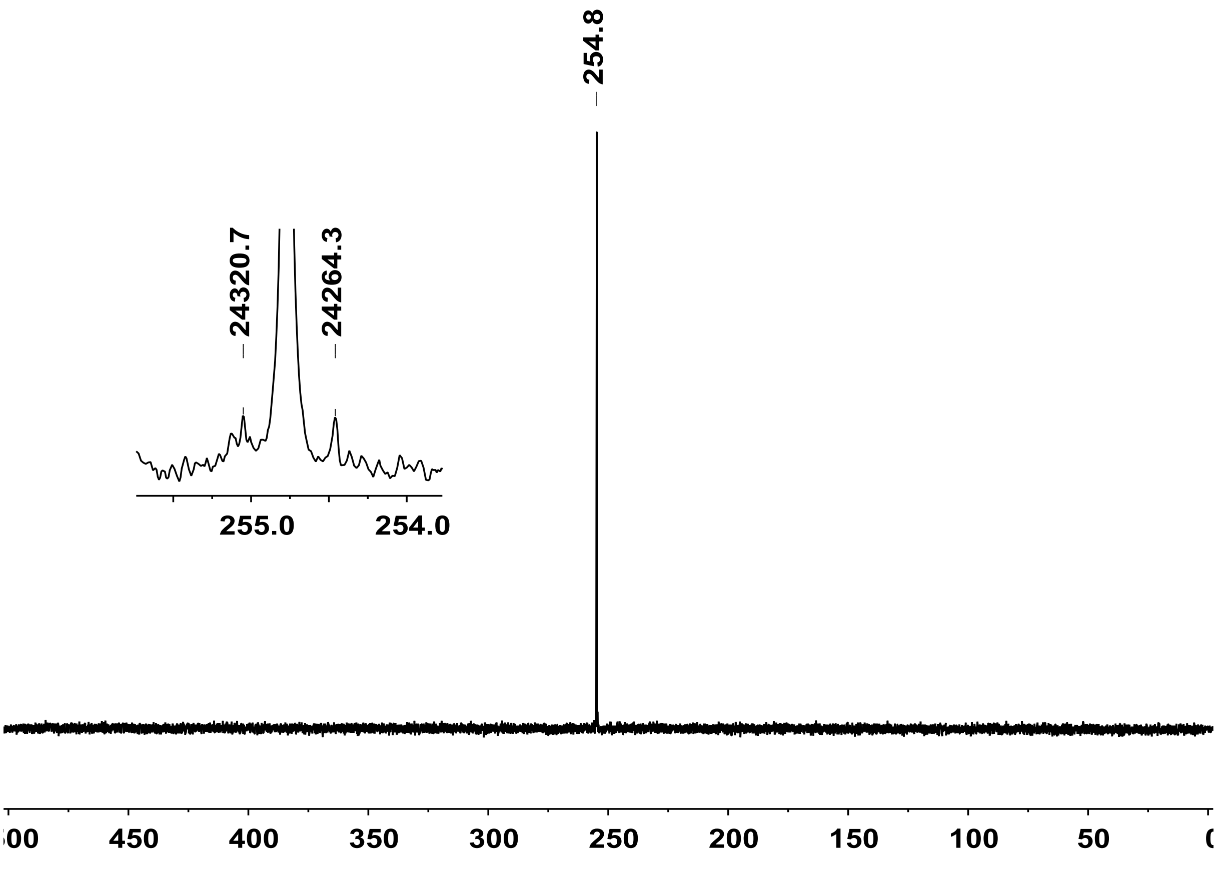

Figure S31 – $^{77}Se\{^1H\}$ NMR spectrum (95.36 MHz, 305.0 K, CD_2Cl_2) of 10c[B(C_6F_5)$_4$].

Tellurylmesityl substituted derivative

The nitrilium ion **10d**[B(C_6F_5)$_4$] was synthesized according to general procedure **C** using 1.0 equiv. 6-dimethylsilyl-5-mesityltellurylacenaphthene **7d** (147 mg, 400 µmol), 1.0 equiv. trityl borate (369 mg, 400 µmol) and 1.0 equiv. 4-fluorobenzonitrile (49 mg, 400 µmol).

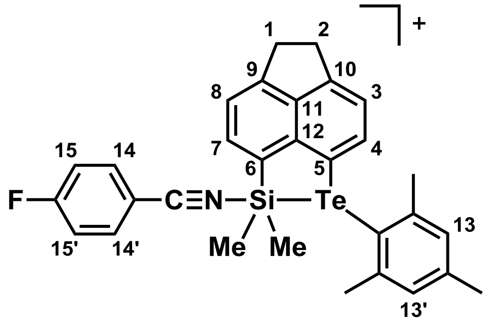

10d [B(C_6F_5)$_4$]$^-$

^{1}H NMR (499.87 MHz, 305.1 K, CD_2Cl_2): δ = 1.11 (s, 6 H, Si(CH_3)$_2$), 2.19 (s, 6 H, *o*-CH_3-Mes), 2.33 (s, 3 H, *p*-CH_3-Mes), 3.59-3.63 (m, 4 H, 2 × CH_2), 7.04 (s, 2 H, H-13, H-13‘), 7.20-7.25 (m, 3 H, H-15, H-15‘), 7.58 (d, $^3J_{H,H}$ = 7.2 Hz, 1 H, H-3), 7.63 (d, $^3J_{H,H}$ = 6.9 Hz, 1 H, H-8), 7.70-7.73 (m, 3 H, H-14, H-14‘), 8.05 (d, $^3J_{H,H}$ = 6.9 Hz, 1 H, H-7), 8.12 (d, $^3J_{H,H}$ = 7.2 Hz, 1 H, H-4). **^{13}C{^{1}H} NMR** (125.71 MHz, 305.0 K, CD_2Cl_2): δ = 1.8 ($^1J_{C,Si}$ = 53.7 Hz, $^2J_{C,Te}$ = 24.2 Hz, Si(CH_3)$_2$), 20.9 (CH_3, *p*-Mes), 25.5 (CH_3, *o*-Mes), 31.3 (CH_2-2), 31.4 (CH_2-1), 108.3 (d, $^4J_{C,F}$ = 2.9 Hz, C-CN), 114.0 (C-5), 115.0 (C, *ipso*-Mes), 117.6 (d, $^2J_{C,F}$ = 22.9 Hz, C-15, C-15’), 119.1 (CN), 122.6 (C-8), 123.0 (C-3), 124.0-125.6 (brs, C, [B(C_6F_5)$_4$]$^-$), 132.2 (C-13, C-13‘), 132.2 (C-6), 135.7 (d, $^3J_{C,F}$ = 9.6 Hz, C-14, C-14’), 137.0 (dm, $^1J_{C,F}$ = 242.0 Hz, CF, [B(C_6F_5)$_4$]$^-$), 137.8 (C-7), 139.0 (dm, $^1J_{C,F}$ = 244.2 Hz, CF, [B(C_6F_5)$_4$]$^-$), 139.4 (C-4), 141.3 (C-11), 142.9 (C-12), 143.8 (C, *p*-Mes), 144.2 (C, *o*-Mes), 149.0 (d, $^1J_{C,F}$ = 240.7 Hz, CF, [B(C_6F_5)$_4$]$^-$), 153.2 (C-9), 154.2 (C-10), 166.2 (d, $^1J_{C,F}$ = 257.1 Hz, CF). **^{19}F{^{1}H} NMR** (470.28 MHz, 305.0 K, CD_2Cl_2): δ = -167.4-(-167.2) (m, 8 F, *m*-F, [B(C_6F_5)$_4$]$^-$), -163.44 (t, $^3J_{F,F}$ = 20.4 Hz, 4 F, *o*-F, [B(C_6F_5)$_4$]$^-$), -132.8-(-132.6) (m, 8 F, *o*-F, [B(C_6F_5)$_4$]$^-$), -102.1 (s, 1 F, CF). **^{29}Si{^{1}H} NMR** (99.31 MHz, 305.0 K, CD_2Cl_2): δ = 48.6 ($^1J_{Si,Te}$ = 171 Hz). **^{29}Si{^{1}H} INEPT NMR** (99.31 MHz, 305.0 K, d_3 = 0.0084, d_4 = 0.0313, CD_2Cl_2): δ = 48.6 ($^1J_{Si,C}$ = 68.2 Hz, $^1J_{Si,C}$ = 53.3 Hz, $^1J_{Si,Te}$ = 171 Hz). **^{125}Te{^{1}H} NMR** (157.75 MHz, 305.0 K, CD_2Cl_2): δ = 219.7 ($^1J_{Te,Si}$ = 171 Hz). **^{11}B{^{1}H} NMR** (160.38 MHz, 305.1 K, CD_2Cl_2): δ = -16.4.

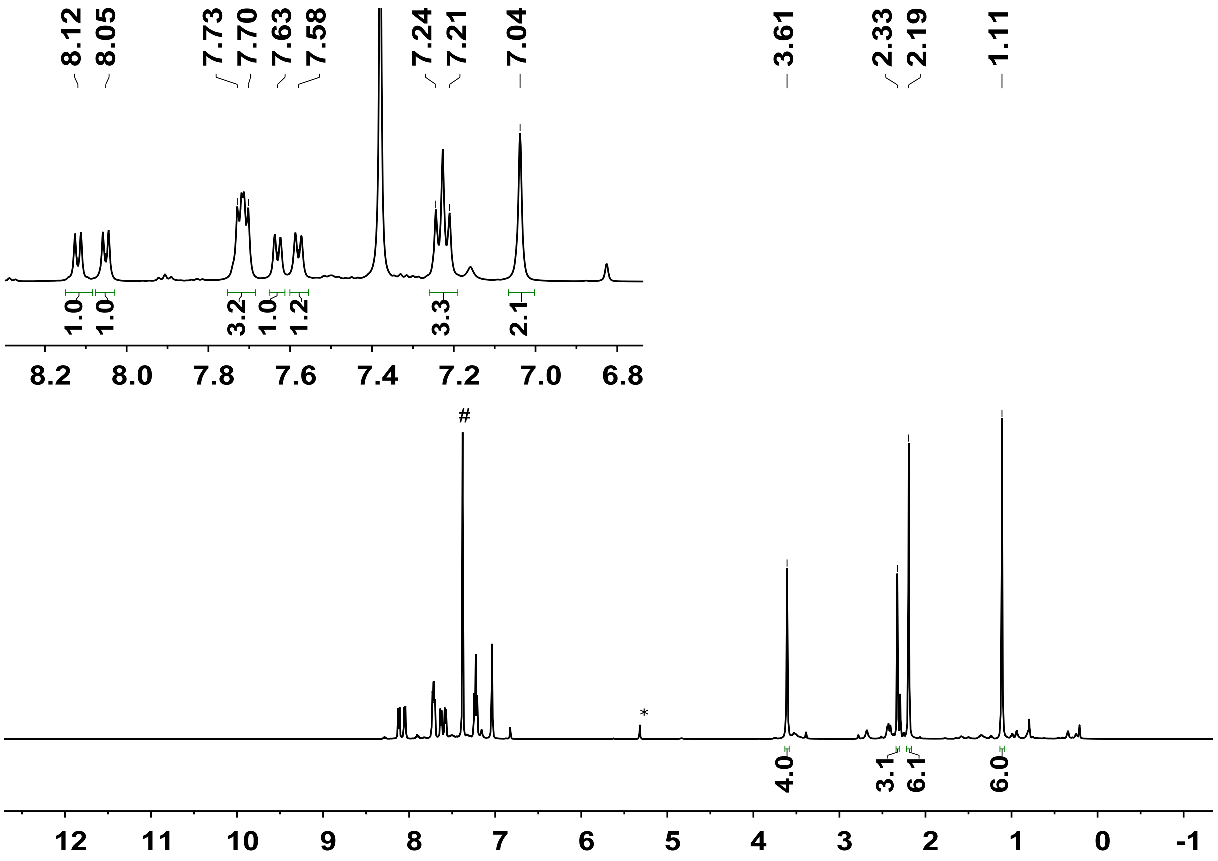

Figure S32 – ^{1}H NMR spectrum (499.87 MHz, 305.1 K, CD_2Cl_2, *$CDHCl_2$, #C_6H_6) of 10d[B(C_6F_5)$_4$].

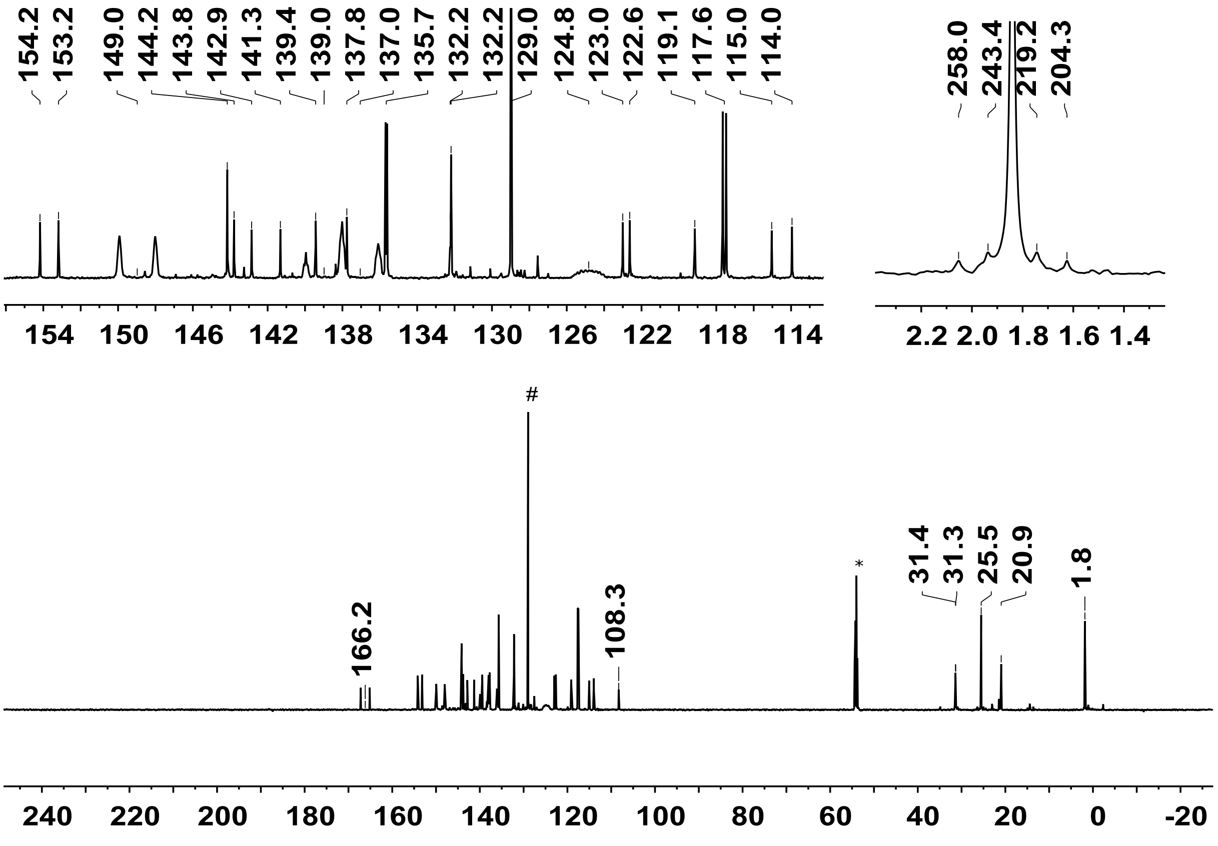

Figure S33 – $^{13}C\{^{1}H\}$ NMR spectrum (125.71 MHz, 305.0 K, *CD_2Cl_2, #C_6H_6) of 10d[B(C_6F_5)$_4$].

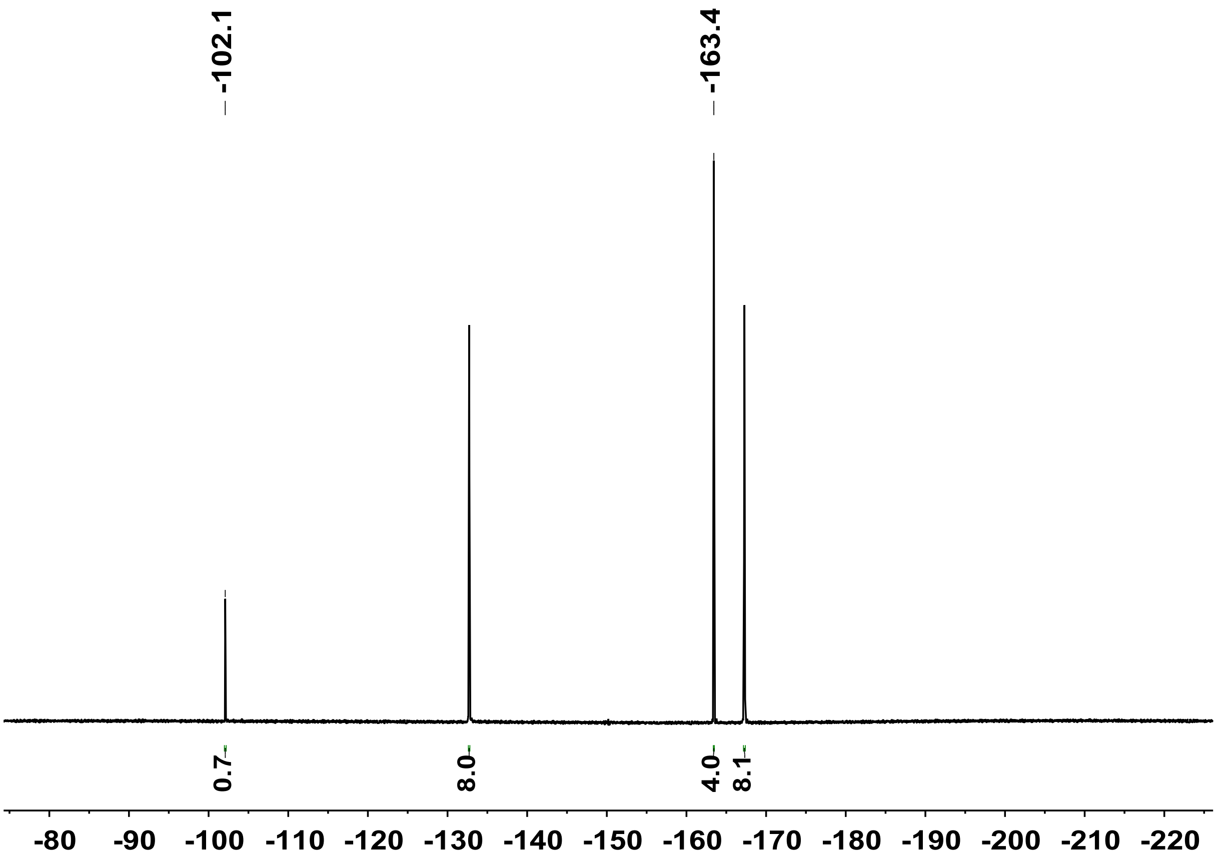

Figure S34 – $^{19}F\{^1H\}$ NMR spectrum (470.28 MHz, 305.0 K, CD_2Cl_2) of 10d[B(C_6F_5)$_4$].

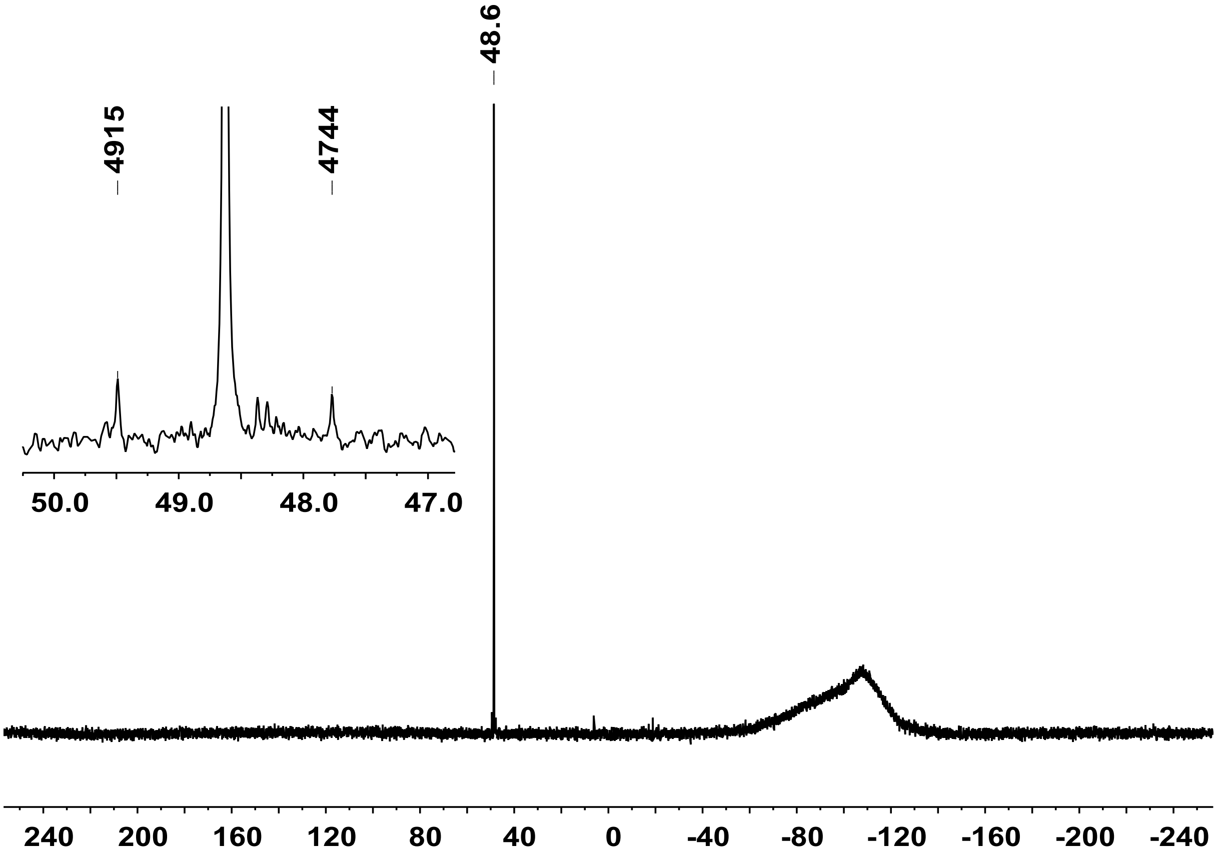

Figure S35 – $^{29}Si\{^1H\}$ NMR spectrum (99.31 MHz, 305.0 K, CD_2Cl_2) of 10d[B(C_6F_5)$_4$].

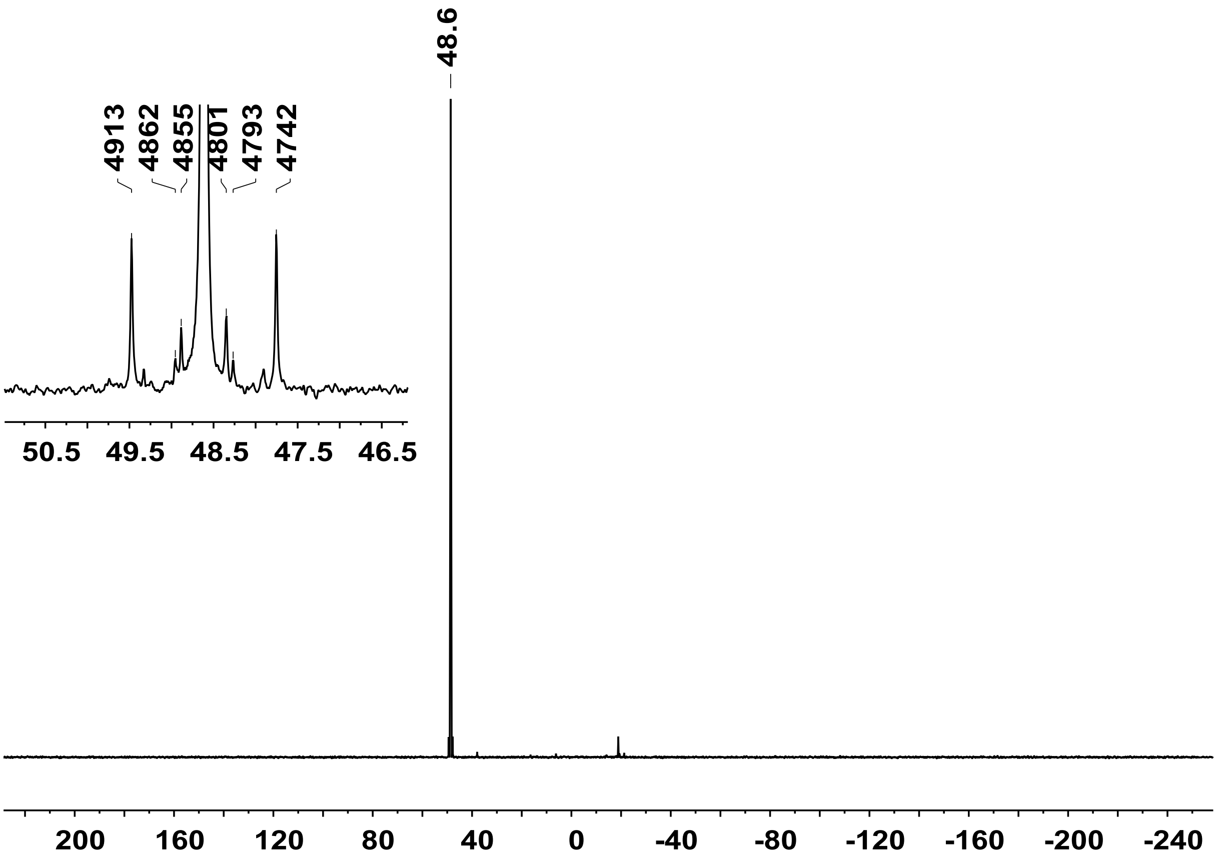

Figure S36 – $^{29}Si\{^1H\}$ INEPT NMR spectrum (99.31 MHz, 305.0 K, d_3 = 0.0084, d_4 = 0.0313, CD_2Cl_2) of 10d[B(C_6F_5)$_4$].

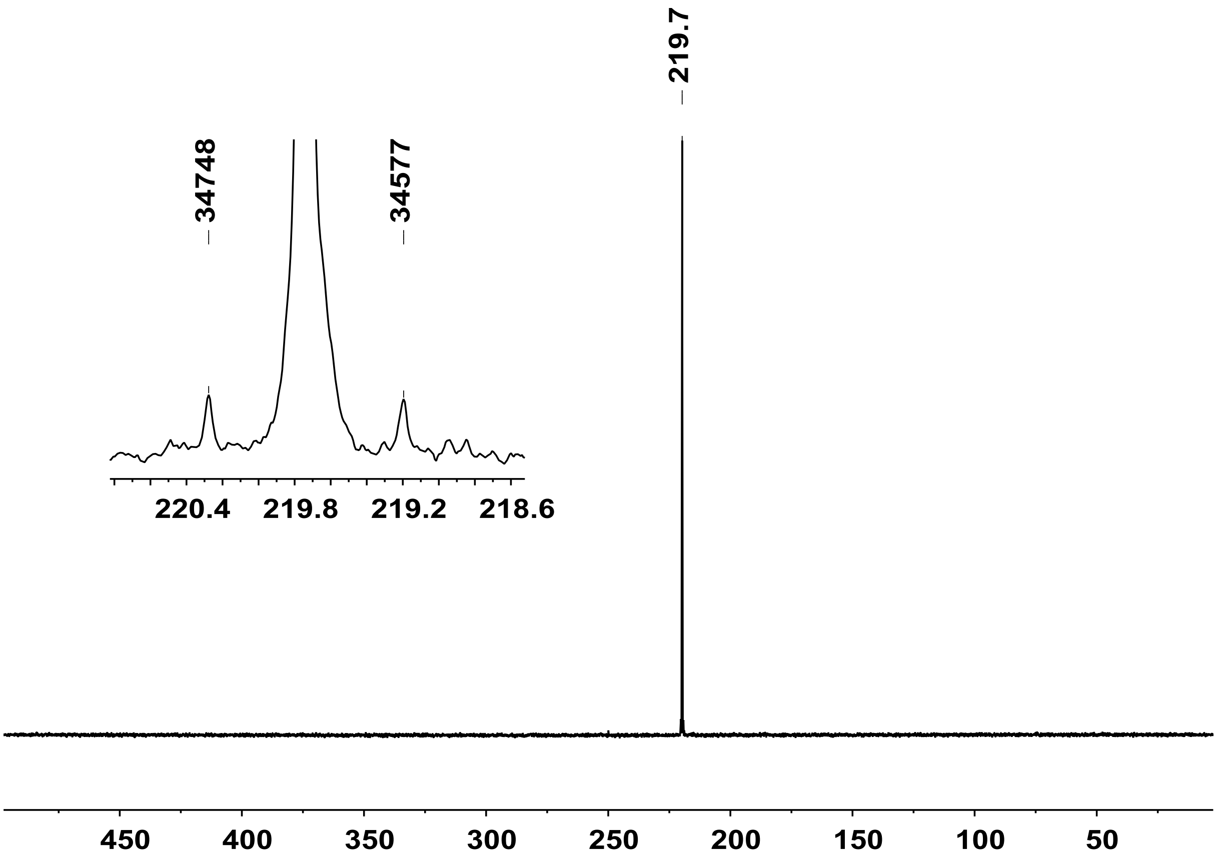

Figure S37 – $^{125}Te\{^1H\}$ NMR spectrum (157.75 MHz, 305.0 K, CD_2Cl_2) of 10d[B(C_6F_5)$_4$].

Halogene substituted derivatives

Bromine

In toluene-d$_8$: A solution of trityl borate (1.0 equiv., 434 µmol, 400 mg) in toluene-d$_8$ was added to a solution of 5-bromo-6-dimethylsilylacenaphthene (1.1 equiv., 477 µmol, 138 mg) in toluene-d$_8$ at -10 °C and stirred for 30 min at the same temperature. Then, the biphasic reaction mixture was allowed to warm to room temperature and the upper, nonpolar phase was removed. The polar phase was washed with 0.3 mL toluene-d$_8$. After the polar phase was analyzed by NMR spectroscopy 4-fluorobenzonitrile (1.0 equiv., 434 µmol, 53 mg) was added. Afterwards, the reaction mixture was stirred for further 10 min and nitrilium borate **10e**[B(C_6F_5)$_4$] was subsequently analyzed by NMR spectroscopy.

In dichloromethane-d$_2$:

A solution of trityl borate (1.0 equiv., 200 mg, 217 µmol) in toluene was added to a solution of 5-bromo-6-dimethylsilylacenaphthene **7e** (1.1 equiv., 70 mg, 239 µmol) in toluene at -10 °C and stirred for 30 min at the same temperature. The reaction mixture was allowed to warm to r.t. and the upper, nonpolar phase was removed. Then 4-fluorobenzonitrile (1.0 equiv., 27 mg, 217 µmol) was added to the reaction mixture and stirred for 10 min. The polar phase was washed with benzene first and then up to two times with *n*-pentane. After removing the solvent under reduced pressure, the residue was dissolved in dichloromethane-d$_2$ and analyzed by NMR spectroscopy.

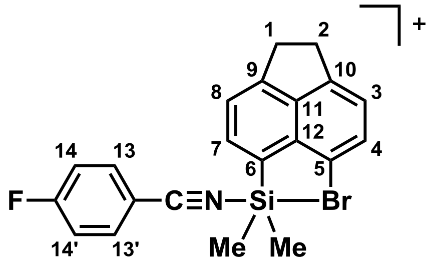

10e [B(C_6F_5)$_4$]$^-$

^{1}H NMR (500.13 MHz, 301.0 K, C_7D_8): δ = 0.95 (s, 6H, Si(CH_3)$_2$), 2.89-2.92 (m, 2H, CH_2), 3.02-3.04 (m, 2H, CH_2), 6.63-6.66 (m, 3H, H-14, H-14'), 6.86 (d, 1H, $^3J_{H,H}$ = 7.6 Hz, H-3), 7.14 (d, 1H, $^3J_{H,H}$ = 7.2 Hz, H-8), 7.17-7.20 (m, 3H, H-13, H-13'), 7.47 (d, 1H, $^3J_{H,H}$ = 7.4 Hz, H-4), 7.80 (d, 1H, $^3J_{H,H}$ = 7.2 Hz, H-7). **^{1}H NMR** (499.87 MHz, 305.0 K,

CD_2Cl_2): δ = 1.30 (s, 6H, $Si(CH_3)_2$), 3.41-3.52 (m, 4H, 2x CH_2), 7.36-7.39 (m, 1H, H-3, overlapping with C_6H_6), 7.42 (d, 1H, $^3J_{H,H}$ = 7.2 Hz, H-8), 7.47 (t, 3H, $^3J_{H,H}$ = 8.3 Hz, H-14, H-14'), 7.87 (d, 1H, $^3J_{H,H}$ = 7.5 Hz, H-4), 8.07 (d, 1H, $^3J_{H,H}$ = 7.3 Hz, H-7), 8.10-8.20 (m, 3H, H-13, H-13'). **^{13}C{^{1}H} NMR** (125.77 MHz, 300.8 K, C_7D_8): δ = 4.5 ($Si(CH_3)_2$), 29.8 (CH_2), 30.7 (CH_2), 100.5 (s, <u>C</u>-CN), 115.6 (C, C-5), 118.2 (d, $^2J_{C,F}$ = 23.6 Hz, CH, C-14, C-14'), 119.8 (C, C-6), 120.3 (CH, C-8), 122.4 (CH, C-3, overlapping with <u>C</u>N), 124.1-125.8 (brs, C, $[B(C_6F_5)_4]^-$),133.7 (CH, C-4), 135.1 (C, C-12), 137.0 (dm, $^1J_{C,F}$ = 242.9 Hz, CF, $[B(C_6F_5)_4]^-$), 137.4 (d, $^3J_{C,F}$ = 10.7 Hz, CH, C-13, C-13'), 139.0 (dm, $^1J_{C,F}$ = 239.7 Hz, CF, $[B(C_6F_5)_4]^-$), 139.6 (CH, C-7), 141.7 (C, C-11), 148.8 (C, C-10), 149.2 (dm, $^1J_{C,F}$ = 242.9 Hz, CF, $[B(C_6F_5)_4]^-$), 154.5 (C, C-9), 168.4 (d, $^1J_{C,F}$ = 269.8 Hz, CF, F-Ph). **^{13}C{^{1}H} NMR** (125.71 MHz, 305.0 K, CD_2Cl_2): δ = 5.6 ($Si(CH_3)_2$), 30.4 (C-2), 31.3 (C-1), 99.4 (s, <u>C</u>-CN), 116.0 (C, C-5), 119.7 (d, $^2J_{C,F}$ = 23.6 Hz, CH, C-14, C-14'), 120.2 (C, C-6), 120.7 (CH, C-8), 122.7 (CH, C-3, overlapping with <u>C</u>N), 123.7-125.5 (brs, C, $[B(C_6F_5)_4]^-$),134.0 (CH, C-4), 135.4 (C, C-12), 136.9 (dm, $^1J_{C,F}$ = 242.5 Hz, CF, $[B(C_6F_5)_4]^-$), 138.9 (dm, $^1J_{C,F}$ = 244.0 Hz, CF, $[B(C_6F_5)_4]^-$), 139.1 (d, $^3J_{C,F}$ = 11.2 Hz, CH, C-13, C-13'), 140.1 (CH, C-7), 142.0 (C, C-11), 148.8 (dm, $^1J_{C,F}$ = 241.6 Hz, CF, $[B(C_6F_5)_4]^-$), 149.2 (C, C-10), 154.8 (C, C-9), 169.9 (d, $^1J_{C,F}$ = 269.7 Hz, CF, F-Ph). **^{19}F{^{1}H} NMR** (470.30 MHz, 305.1K, C_7D_8): δ = -167.4-(-167.3) (m, 8 F, *m*-F, $[B(C_6F_5)_4]^-$), -163.4 (t, $^3J_{F,F}$ = 20.6 Hz, 4 F, *p*-F, $[B(C_6F_5)_4]^-$), -132.8-(-132.5) (m, 8 F, *o*-F, $[B(C_6F_5)_4]^-$), -92.0 (s, 1 F, CF, F-Ph). **^{19}F{^{1}H} NMR** (470.30 MHz, 305.1 K, CD_2Cl_2): δ = -167.5-(-167.2) (m, 8 F, *m*-F, $[B(C_6F_5)_4]^-$), -163.4 (t, $^3J_{F,F}$ = 20.3 Hz, 4 F, *p*-F, $[B(C_6F_5)_4]^-$), -133.2-(-132.5) (m, 8 F, *o*-F, $[B(C_6F_5)_4]^-$), -87.6 (brs, 1 F, CF, F-Ph). **^{29}Si(^{1}H) INEPT NMR** (99.31 MHz, 305.0 K, C_7D_8, d_3 = 0.0084, d_4 = 0.0313): δ = 20.1 ($^1J_{Si,C}$ = 85.3 Hz, $^1J_{Si,C}$ = 65.9 Hz). **^{29}Si{^{1}H} NMR** (99.36 MHz, 300.7 K, C_7D_8): δ = 20.0. **^{29}Si{^{1}H} NMR** (99.31 MHz, 305.0 K, CD_2Cl_2): δ = 20.8. **^{11}B{^{1}H} NMR** (160.38 MHz, 300.8 K, C_7D_8): δ = -16.8. **^{11}B{^{1}H} NMR** (160.38 MHz, 305.0 K, CD_2Cl_2): δ = -16.5.

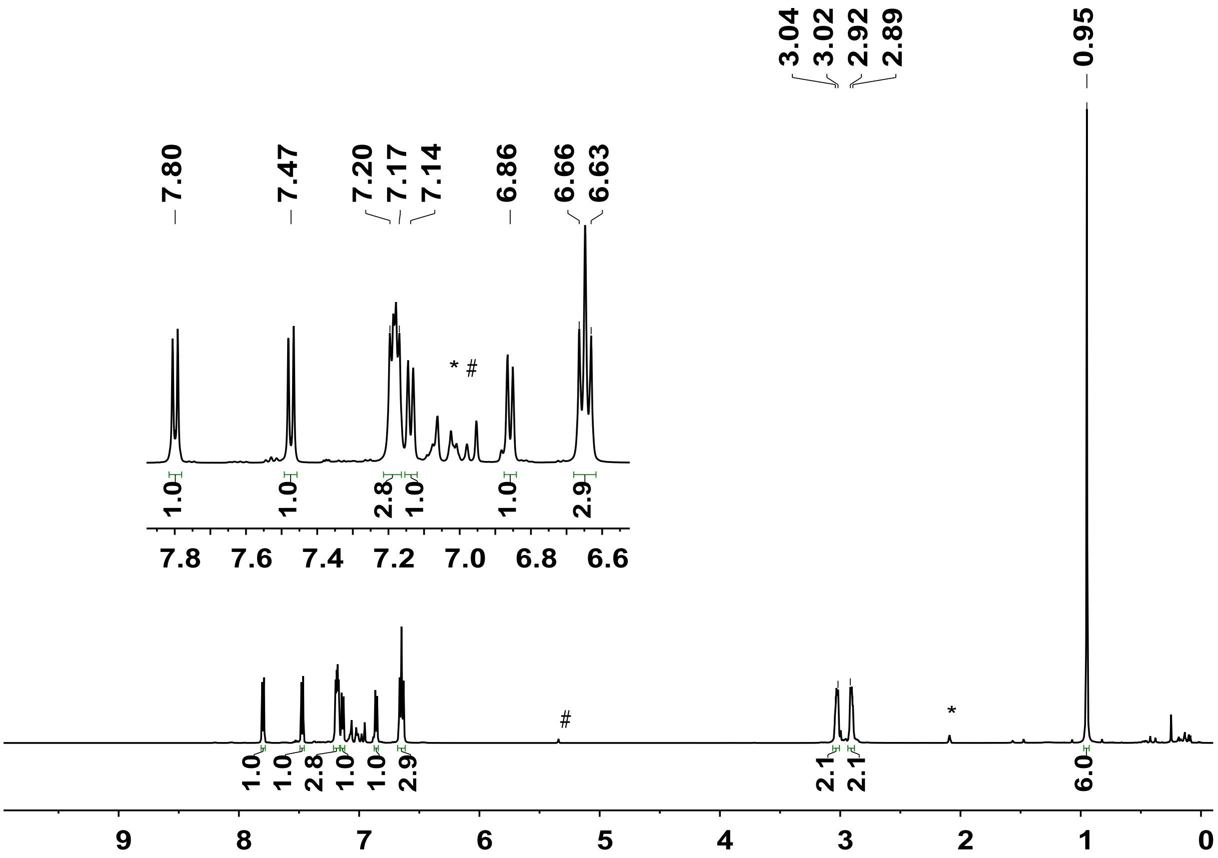

Figure S38 – ^{1}H NMR spectrum (500.13 MHz, 301.0 K, C_7D_8, * C_7D_7H, # triphenylmethane) of 10e[$B(C_6F_5)_4$].

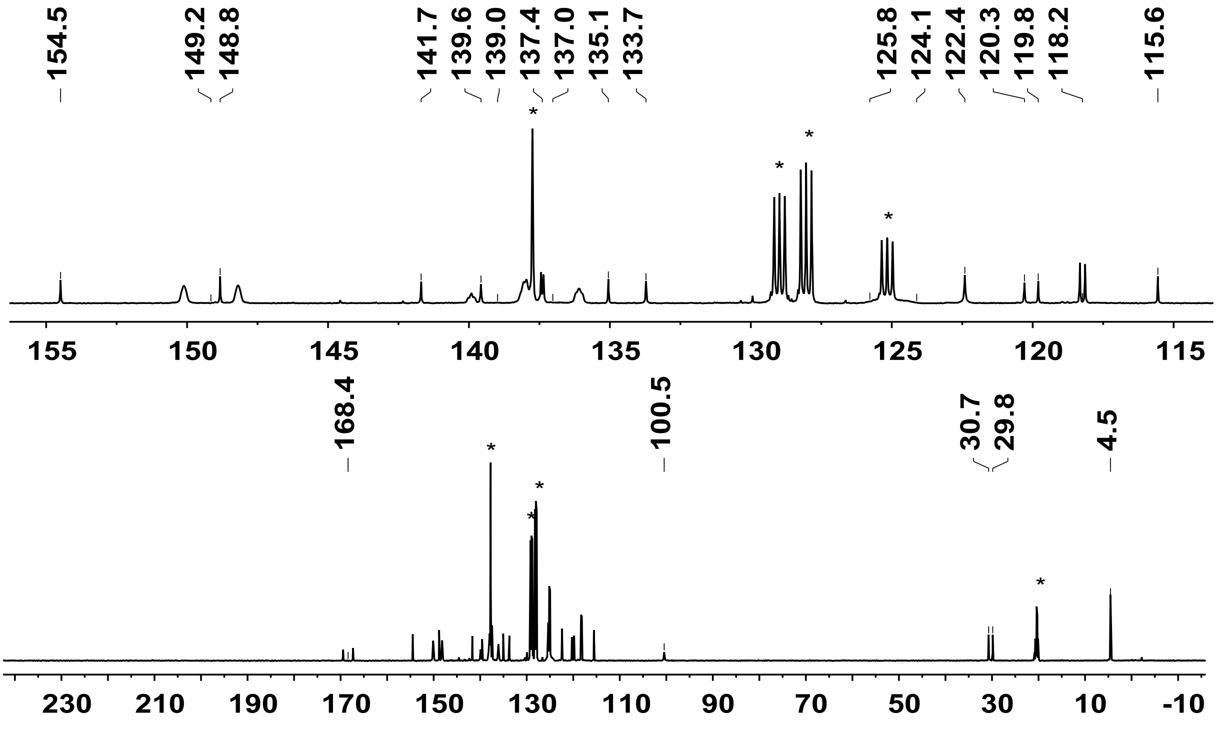

Figure S39 – $^{13}C\{^{1}H\}$ NMR spectrum (125.77 MHz, 300.8 K, * C_7D_8) of 10e[$B(C_6F_5)_4$].

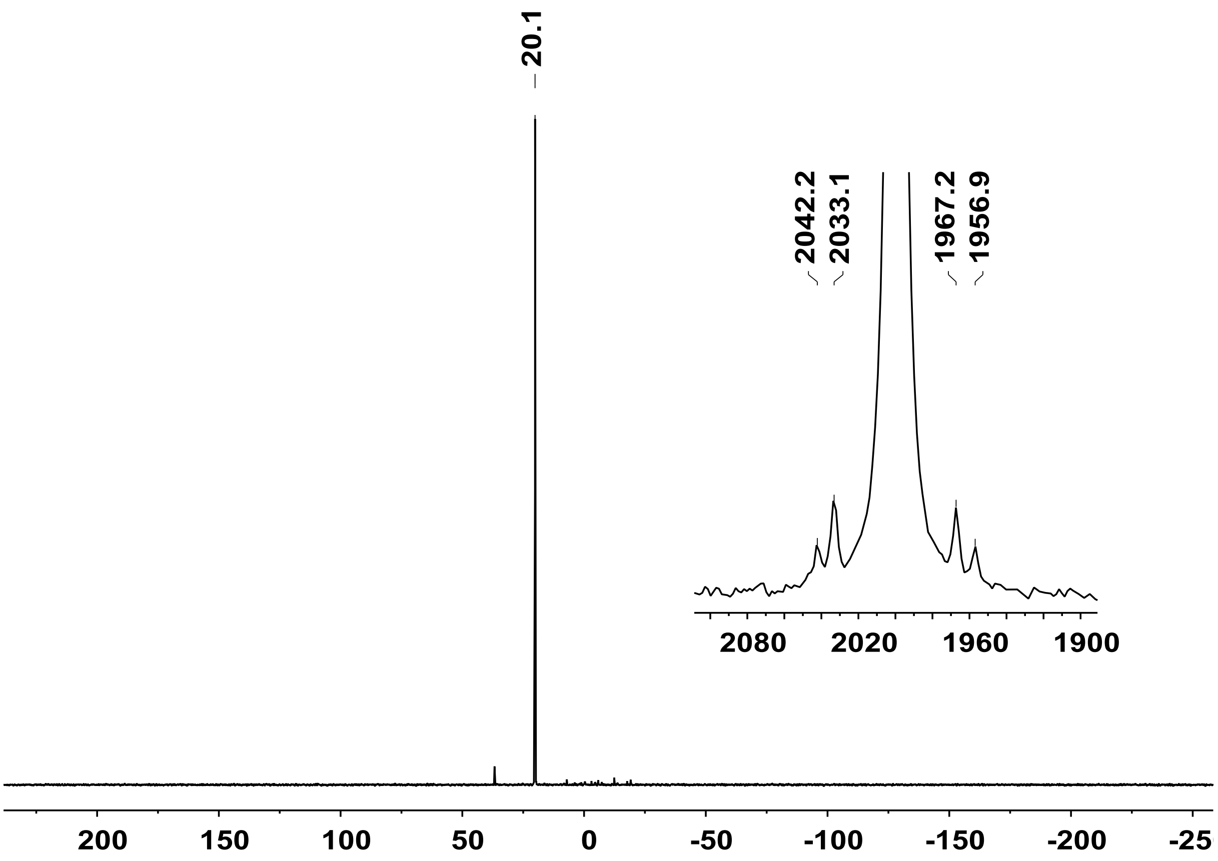

Figure S40 – $^{29}Si\{^1H\}$ INEPT NMR spectrum (99.31 MHz, 305.0 K, C_7D_8, d_3 = 0.0084, d_4 = 0.0313) of 10e[B(C_6F_5)$_4$].

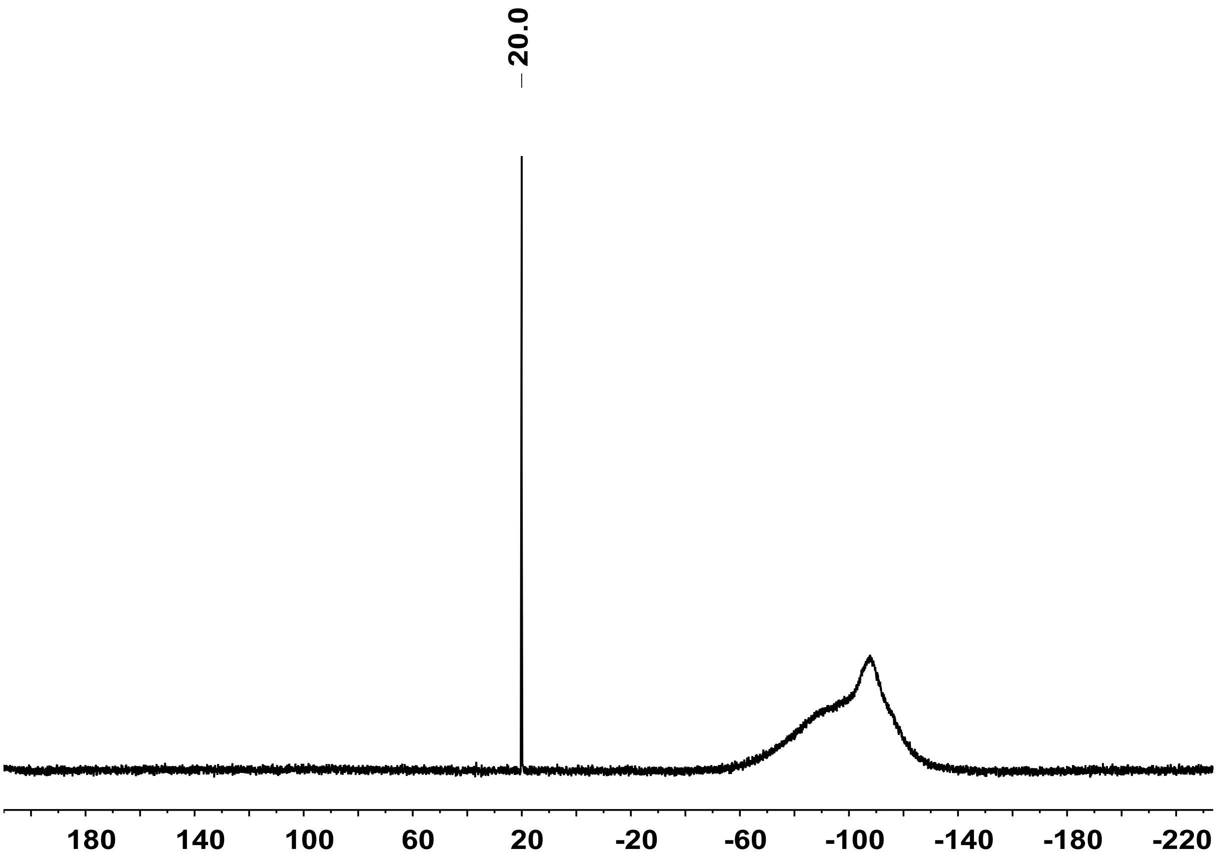

Figure S41 – $^{29}Si\{^1H\}$ NMR spectrum (99.36 MHz, 300.7 K, C_7D_8) of 10e[B(C_6F_5)$_4$].

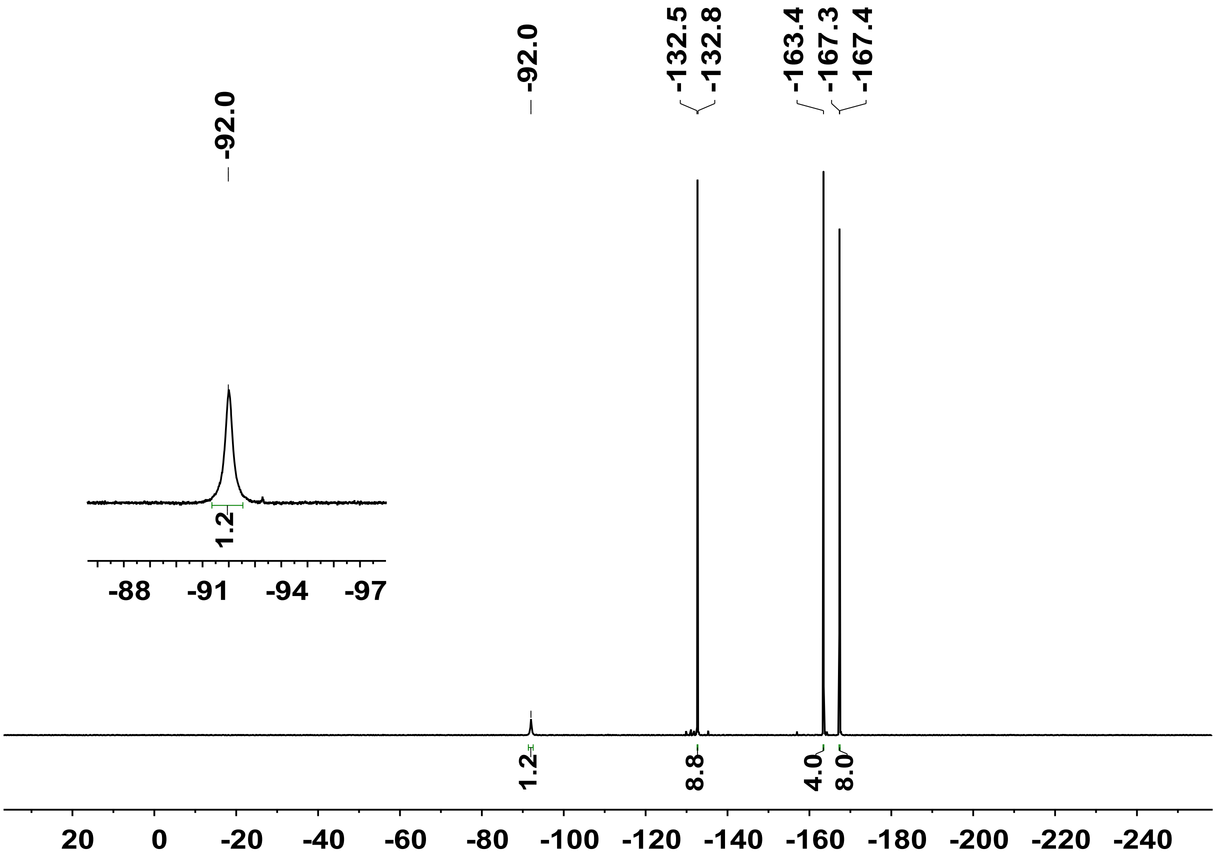

Figure S42 – $^{19}F\{^{1}H\}$ NMR spectrum (470.30 MHz, 305.1 K, C_7D_8) of 10e[B(C_6F_5)$_4$].

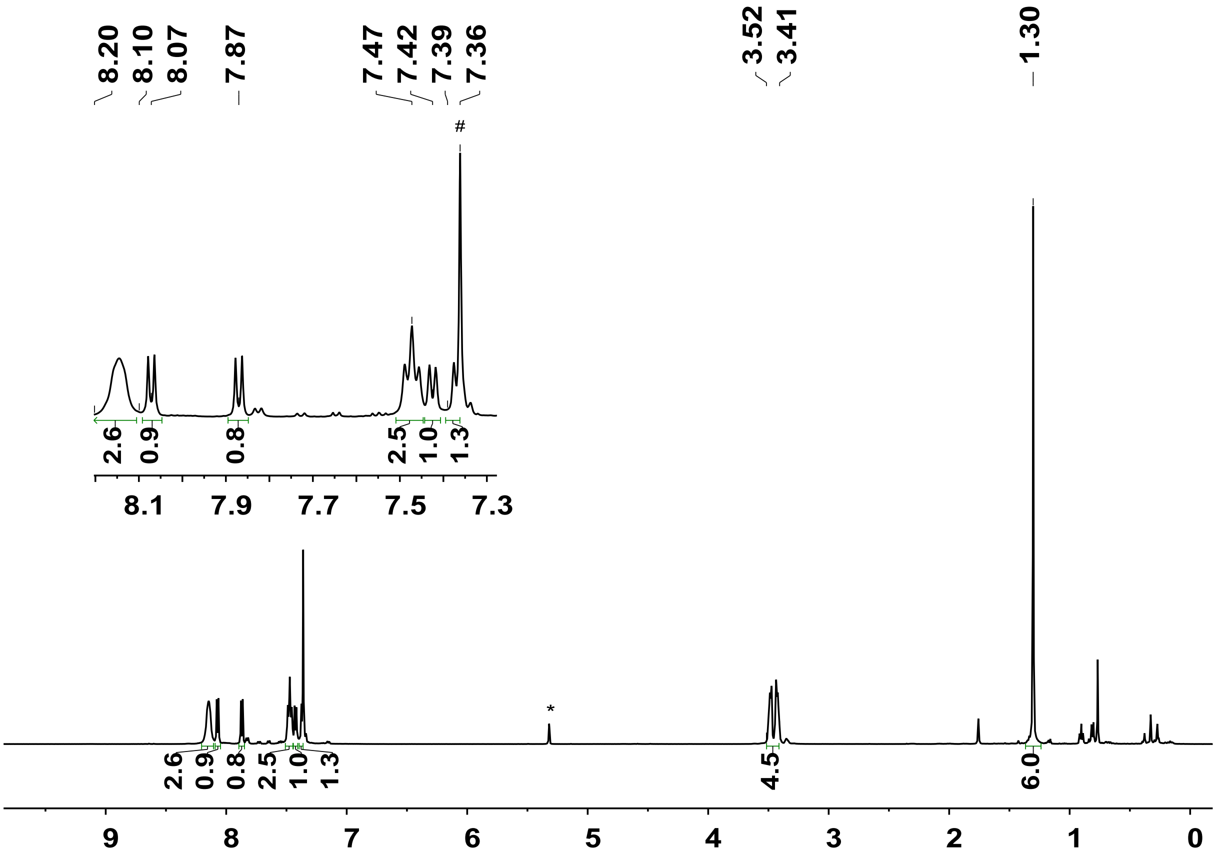

Figure S43 – ^{1}H NMR (499.87 MHz, 305.0 K, *CD_2Cl_2, # C_6H_6) of 10e[B(C_6F_5)$_4$].

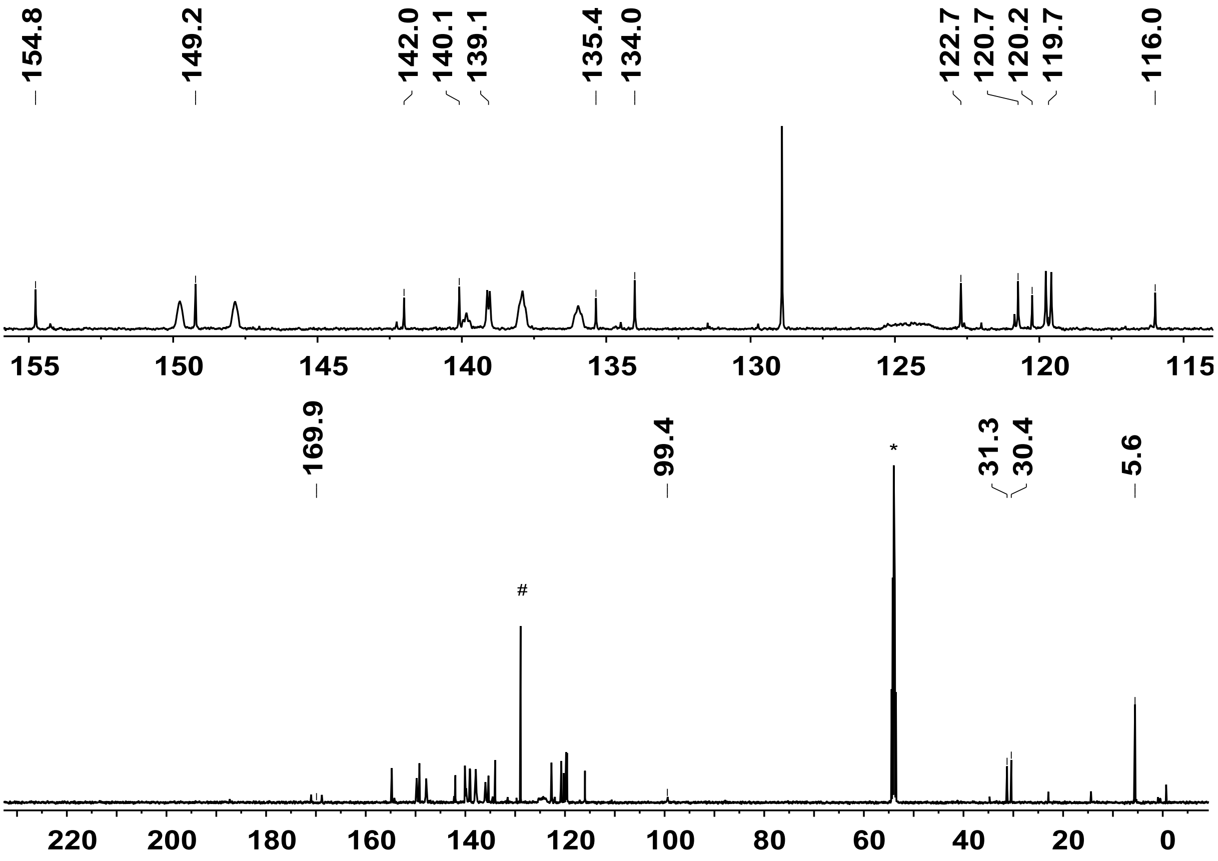

Figure S44 – $^{13}C\{^1H\}$ NMR spectrum (125.71 MHz, 305.0 K, *CD_2Cl_2, # C_6H_6) of 10e[B(C_6F_5)$_4$].

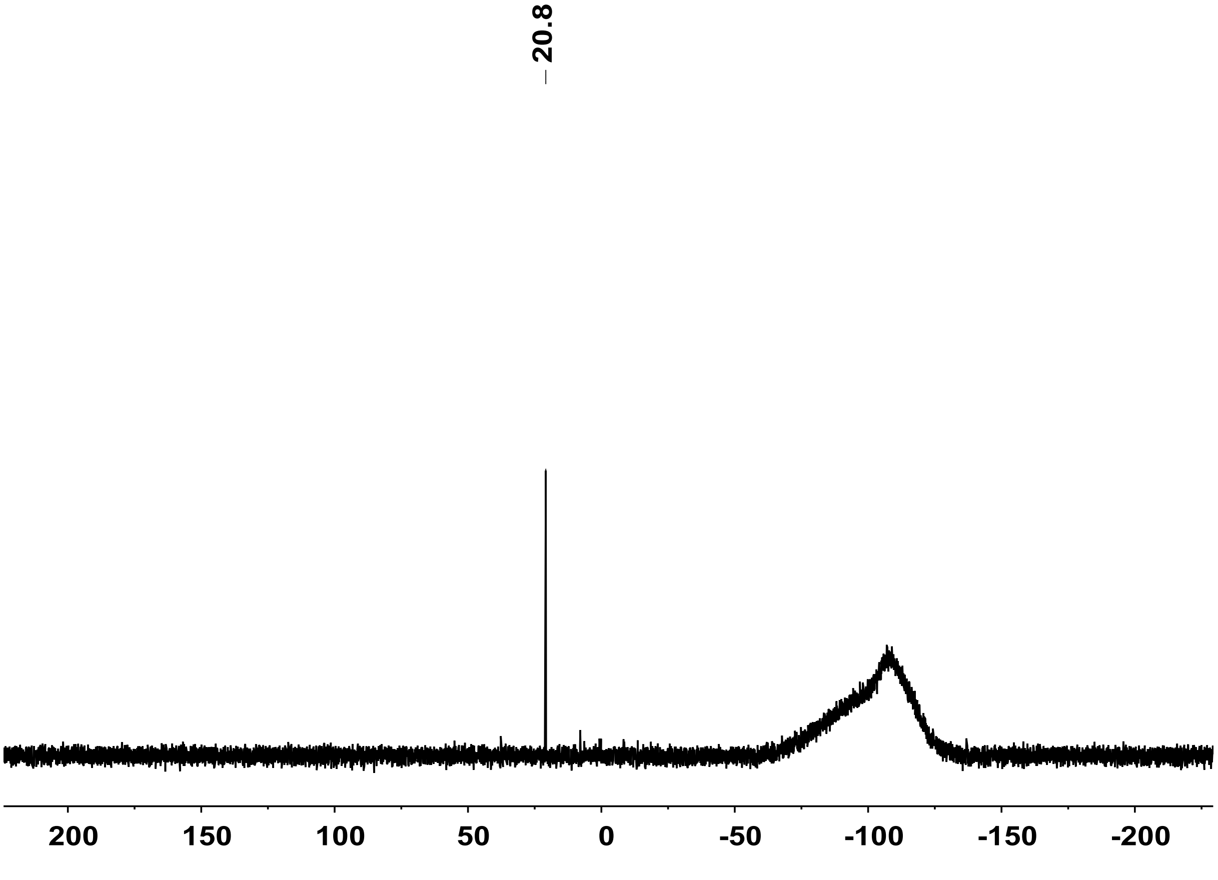

Figure S45 – $^{29}Si\{^1H\}$ NMR spectrum (99.31 MHz, 305.0 K, CD_2Cl_2) of 10e[B(C_6F_5)$_4$].

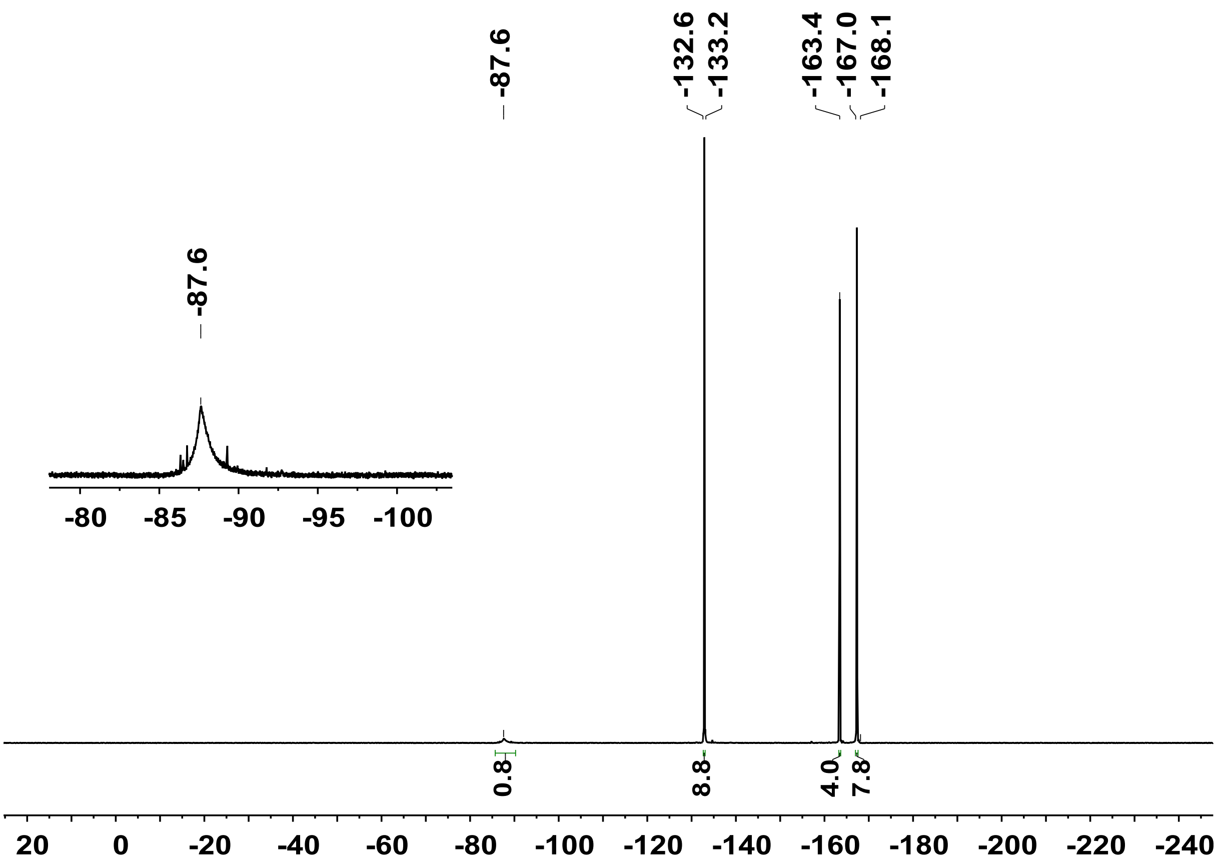

Figure S46 – $^{19}F\{^1H\}$ **NMR spectrum (470.30 MHz, 305.1 K, CD_2Cl_2) of 10e[B(C_6F_5)$_4$].**

A solution of trityl borate (1.0 equiv., 370 mg, 401 µmol) in benzene-d_6 was added to a solution of 1-bromo-8-dimethylsilylnaphthalene (1.2 equiv., 128 mg, 481 µmol) in benzene-d_6 and stirred for 30 min at room temperature. Then 4-fluorobenzonitrile (1.0 equiv., 49 mg, 401 µmol) was added and stirred for additional 10 min. Afterwards the upper, nonpolar phase was removed and after removing the solvent under reduced pressure, the residue was dissolved in dichloromethane-d_2 and nitrilium borate **11e**[B(C_6F_5)$_4$] was analyzed by NMR spectroscopy.

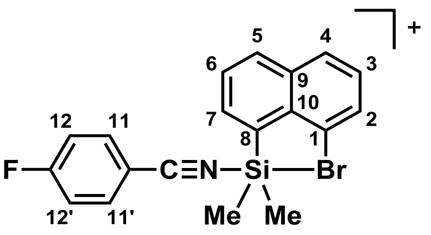

11e **[B(C_6F_5)$_4$]$^-$**

^{1}H NMR (499.87 MHz, 305.1 K, CD_2Cl_2): δ = 1.39 (s, 6H, $Si(CH_3)_2$), 7.48-7.51 (m, 2H, H-12, H-12'), 7.55 (t, 1H, $^3J_{H,H}$ = 7.9 Hz, H-3), 7.65 (t, 1H, $^3J_{H,H}$ = 7.6 Hz, H-6), 7.98 (d, 1H, $^3J_{H,H}$ = 7.7 Hz, H-2), 8.05 (d, 1H, $^3J_{H,H}$ = 8.0 Hz, H-4), 8.16 (d, 1H, $^3J_{H,H}$ = 8.2 Hz, H-5), 8.18-8.21 (m, 2H, H-11, H-11'), 8.27 (d, 1H, $^3J_{H,H}$ = 7.2 Hz, H-7). **^{13}C{^{1}H} NMR** (125.71 MHz, 305.0 K, CD_2Cl_2): δ = 6.5 ($^1J_{C,Si}$ = 68.2 Hz, $Si(CH_3)_2$), 99.2 (s, C-CN), 119.7 (d, $^2J_{C,F}$ = 23.7 Hz, CH, C-12, C-12'), 121.9 (C, C-1), 123.7 (CN), 124.0-125.6 (brs, C, $[B(C_6F_5)_4]^-$), 126.6 (CH, C-6), 127.0 (C, C-8), 128.2 (CH, C-3), 130.9 (CH, C-4), 132.2 (CH, C-2), 134.9 (CH, C-5), 136.6 (C, C-9 or C-10), 136.9 (C, C-9 or C-10), 137.0 (dm, $^1J_{C,F}$ = 242.7 Hz, CF, $[B(C_6F_5)_4]^-$), 138.1 (CH, C-7), 139.0 (dm, $^1J_{C,F}$ = 240.0 Hz, CF, $[B(C_6F_5)_4]^-$), 139.1 (d, $^3J_{C,F}$ = 11.2 Hz, CH, C-11, C-11'), 148.9 (dm, $^1J_{C,F}$ = 240.6 Hz, CF, $[B(C_6F_5)_4]^-$), 170.0 (d, $^1J_{C,F}$ = 269.1 Hz, CF, F-Ph). **^{19}F{^{1}H} NMR** (470.30 MHz, 305.1 K, CD_2Cl_2): δ = -167.4-(-167.3) (m, 8 F, *m*-F, $[B(C_6F_5)_4]^-$), -163.4 (t, $^3J_{F,F}$ = 20.4 Hz, 4 F, *p*-F, $[B(C_6F_5)_4]^-$), -133.6-(-132.7) (m, 8 F, *o*-F, $[B(C_6F_5)_4]^-$), -88.0 (brs, 1 F, CF, F-Ph). **^{29}Si(^{1}H) INEPT NMR** (99.31 MHz, 305.0 K, CD_2Cl_2, d_3 = 0.0084, d_4 = 0.0313): δ = 14.8 ($^1J_{Si,C}$ = 85.5 Hz, $^1J_{Si,C}$ = 68.3 Hz). **^{29}Si{^{1}H} NMR** (99.31 MHz, 305.0 K, CD_2Cl_2): δ = 14.8. **^{11}B{^{1}H} NMR** (160.38 MHz, 305.0 K, CD_2Cl_2): δ = -16.5.

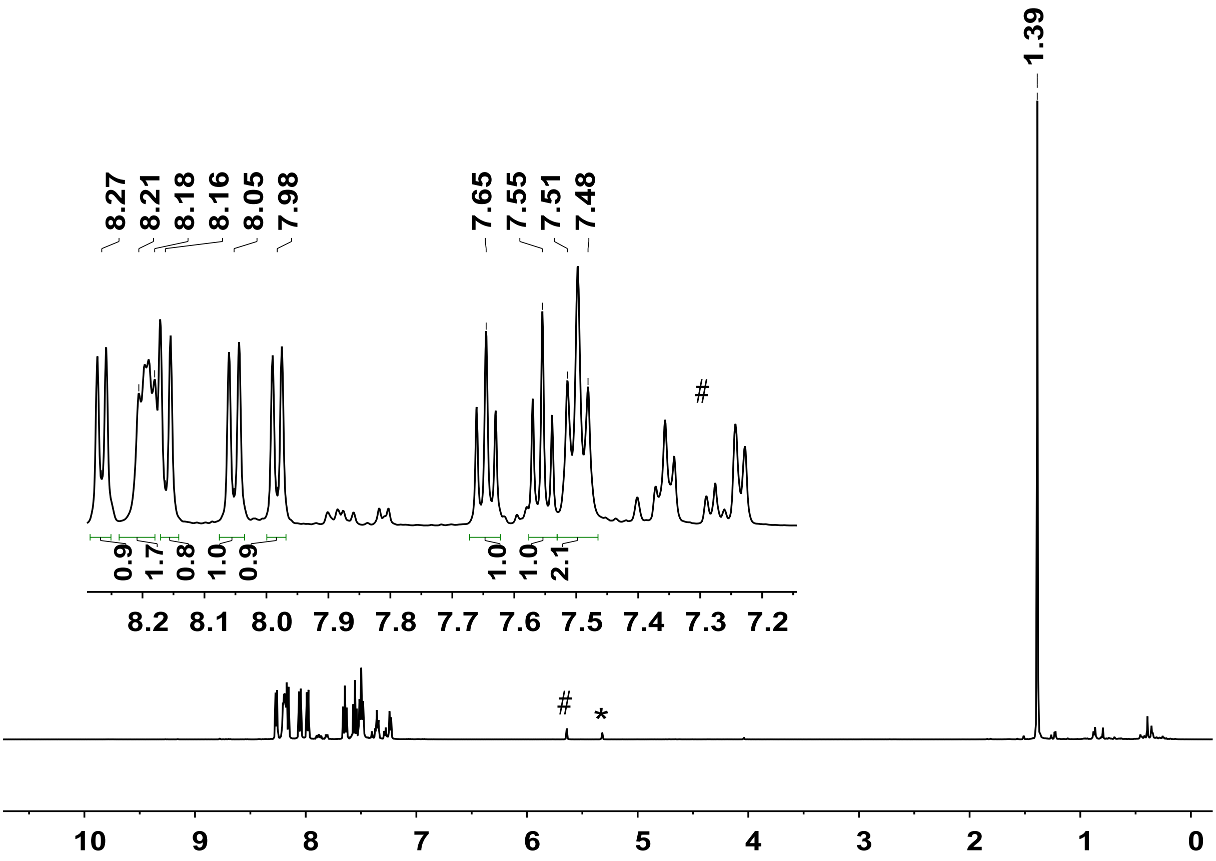

Figure S47 – ^{1}H NMR (499.87 MHz, 305.1 K, *CD_2Cl_2, # Ph_3CH) of 11e[$B(C_6F_5)_4$].

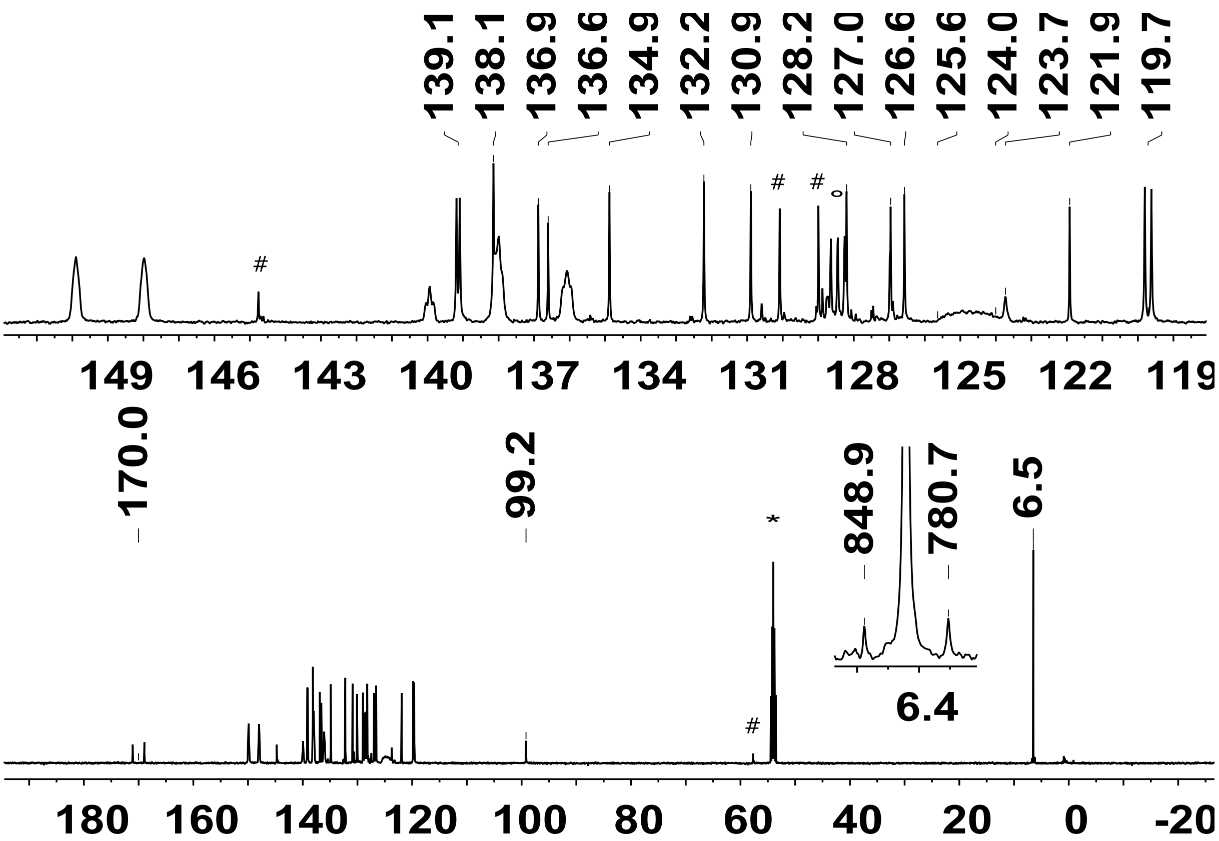

Figure S48 – $^{13}C\{^1H\}$ NMR spectrum (125.71 MHz, 305.0 K, *CD_2Cl_2, # Ph_3CH, ° C_6D_6) of 11e[B(C_6F_5)$_4$].

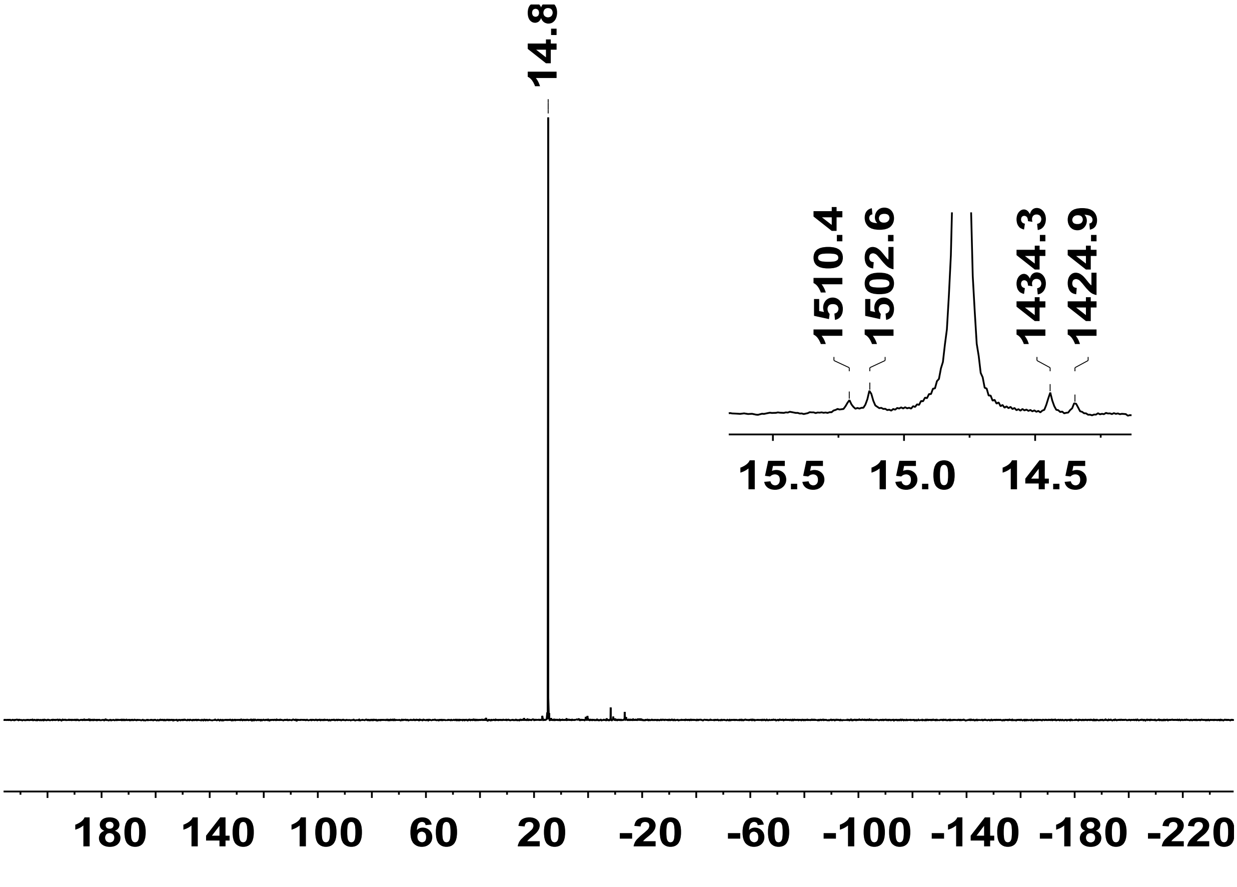

Figure S49 – $^{29}Si\{^1H\}$ INEPT NMR spectrum (99.31 MHz, 305.0 K, CD_2Cl_2, d_3 = 0.0084, d_4 = 0.0313) of 11e[B(C_6F_5)$_4$].

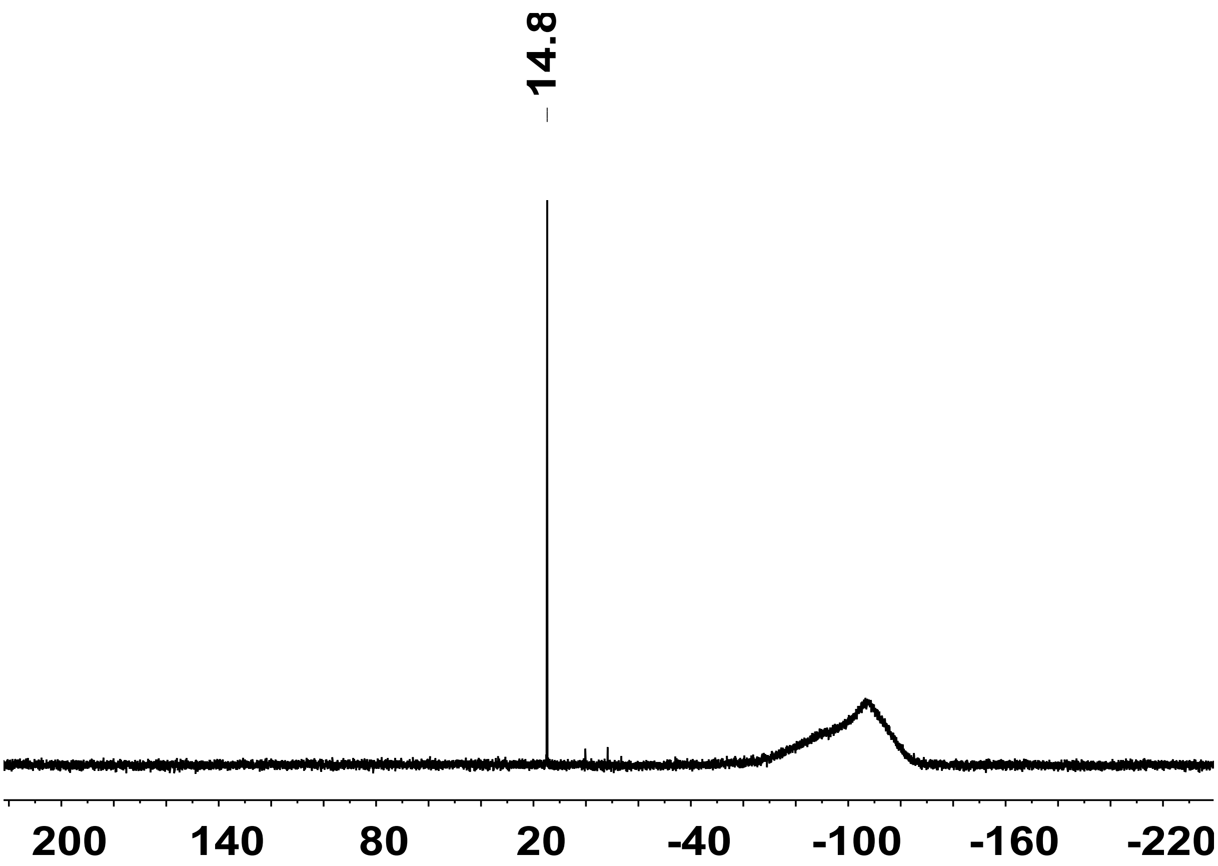

Figure S50 – $^{29}Si\{^1H\}$ NMR spectrum (99.31 MHz, 305.0 K, CD_2Cl_2) of 11e[B(C_6F_5)$_4$].

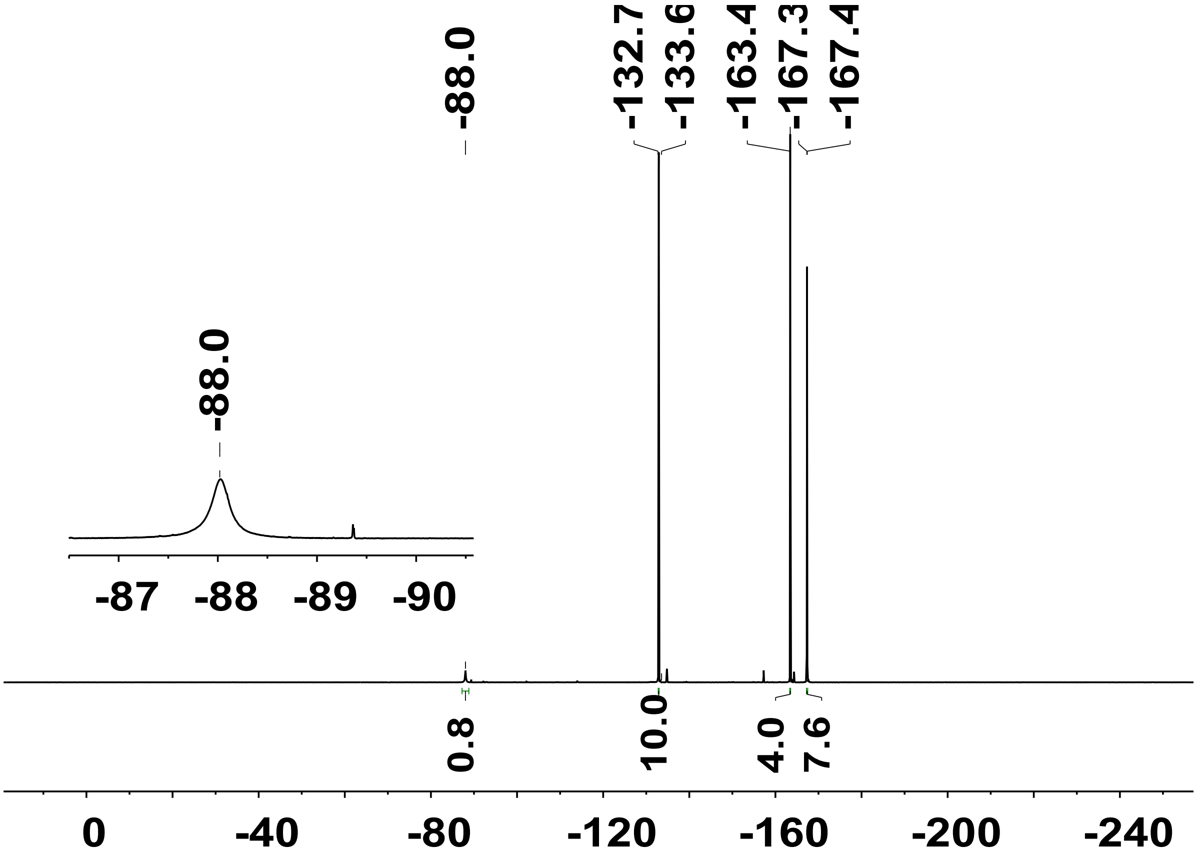

Figure S51 – $^{19}F\{^1H\}$ NMR spectrum (470.30 MHz, 305.1 K, CD_2Cl_2) of 11e[B(C_6F_5)$_4$].

Iodine

A solution of trityl borate (1.0 equiv., 370 mg, 401 µmol) in benzene was added to a solution of a reaction mixture containing 5-iodo-6-dimethylsilylacenaphthene **7f** as main product (248 mg) in benzene and stirred for 30 min at room temperature. Here the reaction mixture containing 5-iodo-6-dimethylsilylacenaphthene **7f** was used in excess compared to the amount of the trityl borate. The upper, nonpolar phase was removed and the polar phase was washed with benzene two times and then with *n*-pentane to remove organic impurities and excess of silane **7f**. Afterwards the solvent was removed under reduced pressure, the residue was dissolved in benzene-d_6 and analyzed by NMR spectroscopy. Then 4-fluorobenzonitrile (1.0 equiv., 49 mg, 401 µmol) was added to the reaction mixture and stirred for 15 min. The polar phase was washed up to two times with *n*-pentane. After removing the solvent under reduced pressure, the residue was dissolved in dichloromethane-d_2 and nitrilium borate **10f**$[B(C_6F_5)_4]$ was analyzed by NMR spectroscopy.

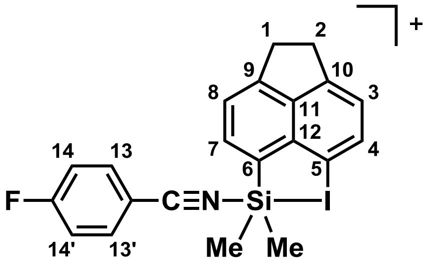

10f $[B(C_6F_5)_4]^-$

^{1}H NMR (499.87 MHz, 305.0 K, CD_2Cl_2): δ = 1.47 (s, 6H, $Si(CH_3)_2$), 3.39-3.55 (m, 4H, 2 x CH_2, H-1, H-2), 7.26 (d, 1H, $^3J_{H,H}$ = 6.8 Hz, H-3), 7.39-7.44 (m, 3H, H-8, H-14, H-14’), 8.04-8.11 (m, 2H, H-13, H-13’), 8.13 (d, 1H, $^3J_{H,H}$ = 7.1 Hz, H-7), 8.16 (d, 1H, $^3J_{H,H}$ = 7.1 Hz, H-4). **^{13}C{^{1}H} NMR** (125.71 MHz, 305.0 K, CD_2Cl_2): δ = 7.9 (Si($\underline{C}H_3)_2$), 30.4 (CH_2), 30.9 (CH_2), 90.4 (C, C-5), 101.0 (s, $\underline{C}$-CN), 119.3 (d, $^2J_{C,F}$ = 23.5 Hz, CH, C-14, C-14’), 120.6 (CH, C-8), 122.7 ($\underline{C}$N), 123.2 (C, C-6), 123.5 (CH, C-3), 123.8-125.6 (brs, C, $[B(C_6F_5)_4]^-$), 137.0 (dm, $^1J_{C,F}$ = 240.8 Hz, CF, $[B(C_6F_5)_4]^-$), 138.4 (d, $^3J_{C,F}$ = 10.9 Hz, CH, C-13, C-13’), 138.9 (dm, $^1J_{C,F}$ = 244.3 Hz, CF, $[B(C_6F_5)_4]^-$), 139.5 (C, C-12), 140.6 (CH, C-7), 142.0 (CH, C-4), 142.1 (C, C-11), 148.9 (dm, $^1J_{C,F}$ = 240.5 Hz, CF, $[B(C_6F_5)_4]^-$), 150.2 (C, C-10), 155.1 (C, C-9), 169.2 (d, $^1J_{C,F}$ = 266.7 Hz, CF, F-Ph).

$^{19}F\{^1H\}$ NMR (470.30 MHz, 305.1 K, CD_2Cl_2): δ = -167.4-(-167.3) (m, 8F, *m*-F, $[B(C_6F_5)_4]$), -163.4 (t, $^3J_{F,F}$ = 20.4 Hz, 4F, *p*-F, $[B(C_6F_5)_4]^-$), -133.3-(-132.6) (m, 8F, *o*-F, $[B(C_6F_5)_4]^-$), -90.9 (brs, 1F, CF, F-Ph). **$^{29}Si\{^1H\}$ NMR** (99.31 MHz, 305.0 K, CD_2Cl_2): δ = 20.0. **$^{11}B\{^1H\}$ NMR** (160.38 MHz, 305.0 K, CD_2Cl_2): δ = -16.5.

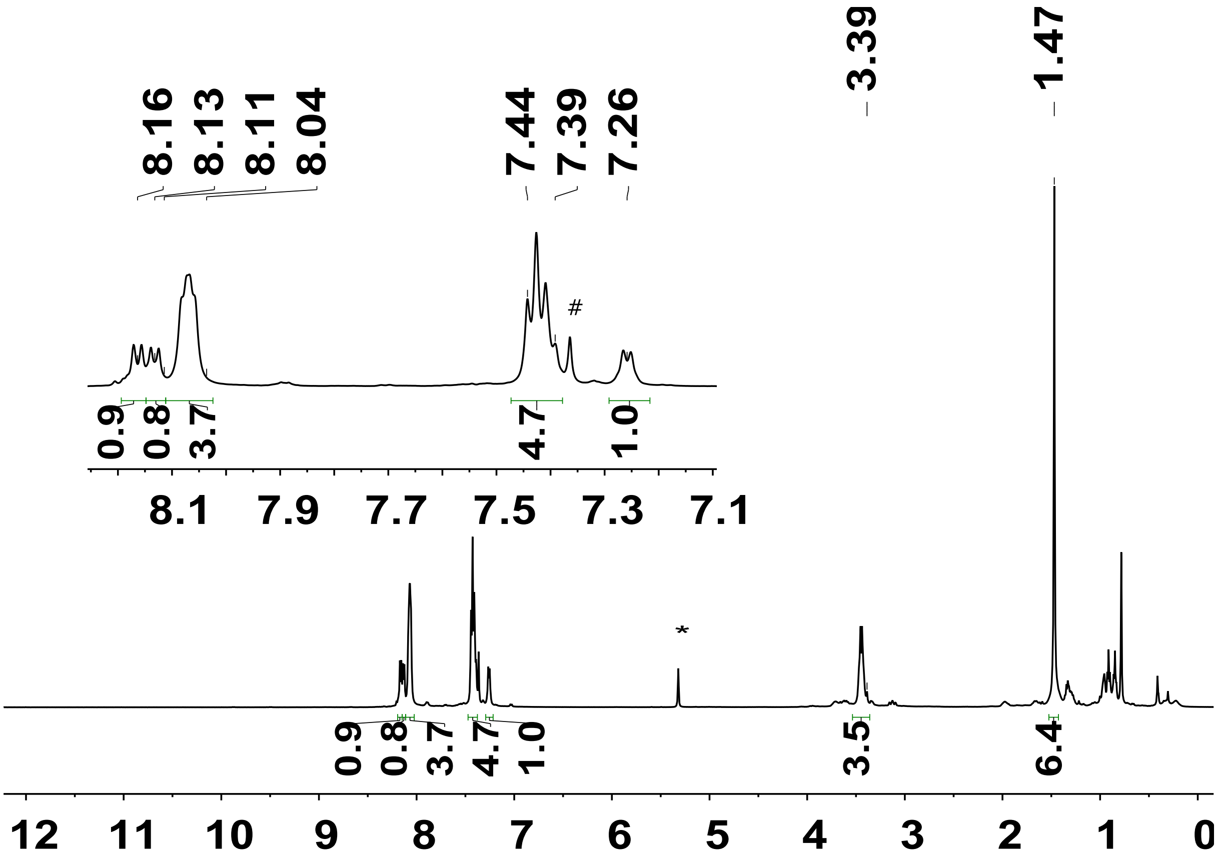

Figure S52 – ^1H NMR (499.87 MHz, 305.0 K, *CD_2Cl_2, # C_6D_6) of 10f$[B(C_6F_5)_4]$.

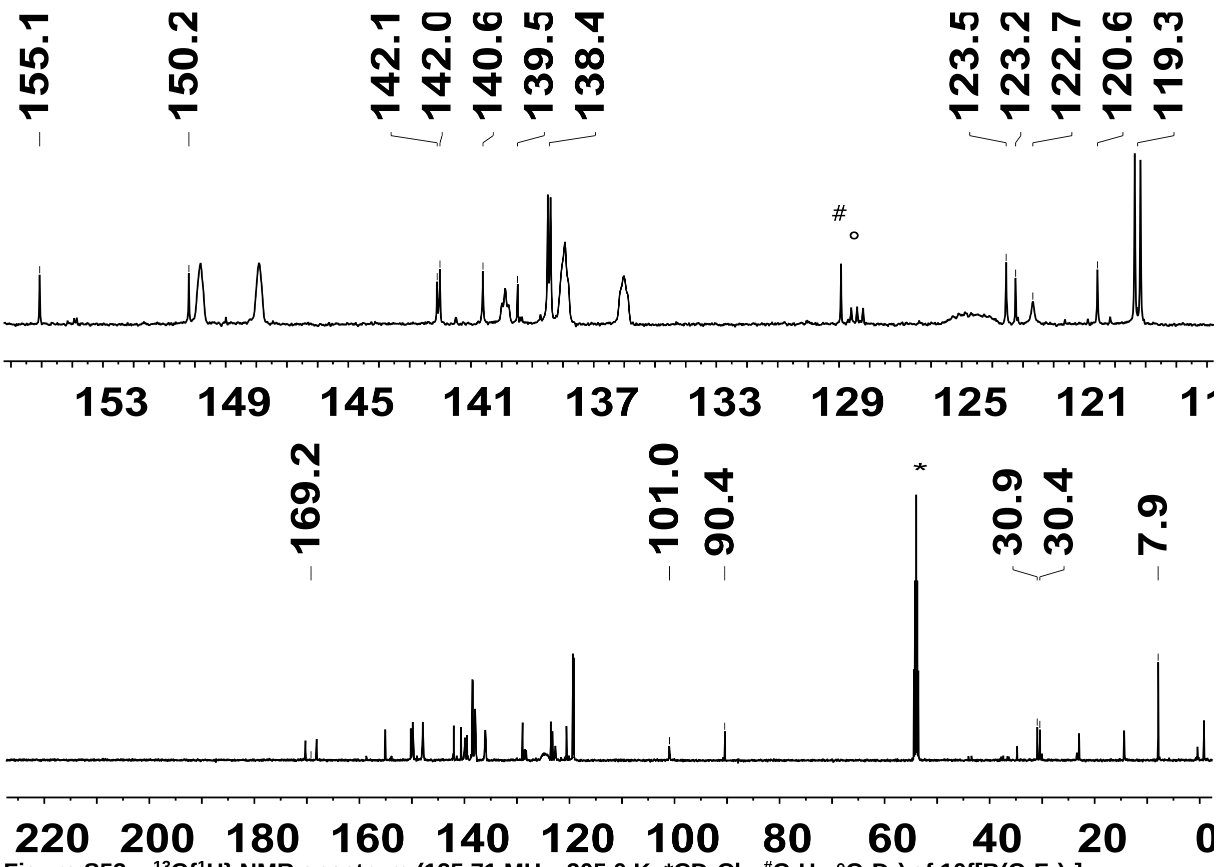

Figure S53 – $^{13}C\{^1H\}$ NMR spectrum (125.71 MHz, 305.0 K, *CD_2Cl_2, #C_6H_6, °C_6D_6) of 10f[B(C_6F_5)$_4$].

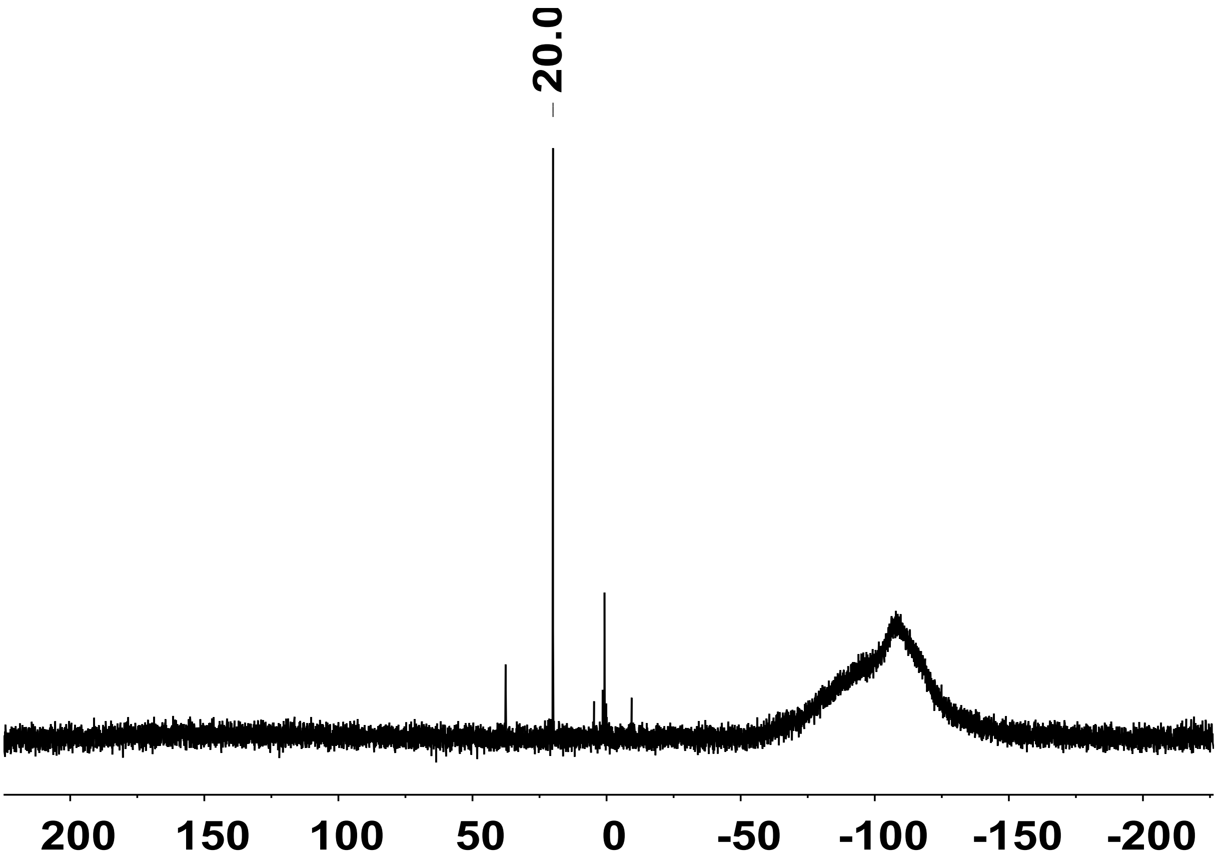

Figure S54 – $^{29}Si\{^1H\}$ NMR spectrum (99.31 MHz, 305.0 K, CD_2Cl_2) of 10f[B(C_6F_5)$_4$].

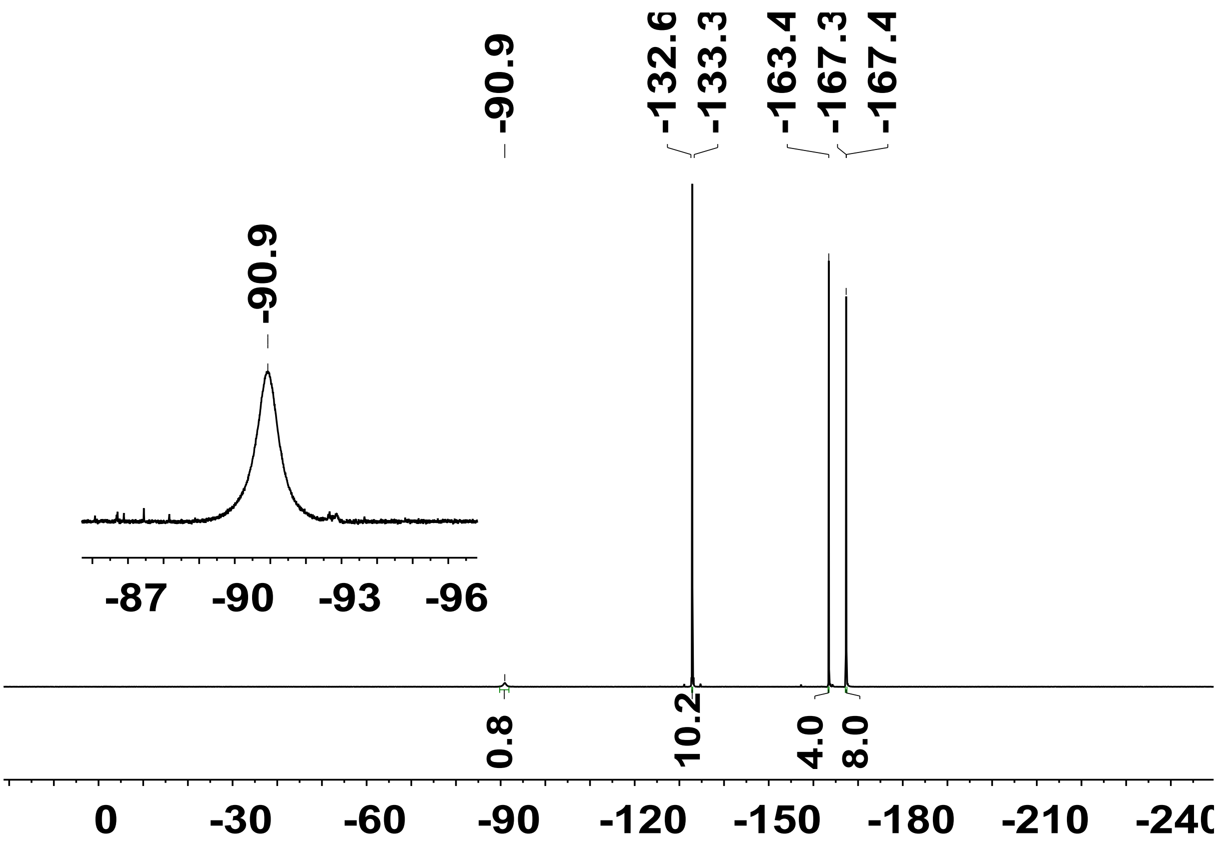

Figure S55 – $^{19}F\{^1H\}$ NMR spectrum (470.30 MHz, 305.1 K, CD_2Cl_2) of 10f[$B(C_6F_5)_4$].

Without donor

Nitrilium borate **6**[B(C_6F_5)$_4$] was synthesized according to general procedure **B** using 1.0 equiv. 5-dimethylsilylacenaphthene (250.00 µmol, 53 mg), 1.0 equiv. trityl borate (250.00 µmol, 230 mg) and 1.0 equiv. 4-fluorobenzonitrile (250.00 µmol, 30 mg). Nitrilium ion **6** is not stable and decomposes within one day.

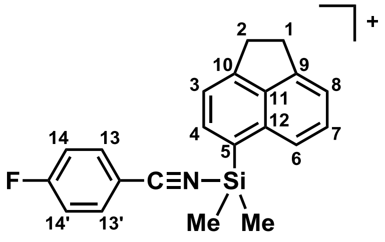

6 **[B(C_6F_5)$_4$]$^-$**

^{1}H NMR (499.87 MHz, 305.1 K, CD_2Cl_2): δ = 1.26 (s, 6 H, Si(CH_3)$_2$), 3.53 (brs, 4 H, 2 × CH_2), 7.40-7.44 (m, 2 H, H-14, H-14‘), 7.50 (d, $^3J_{H,H}$ = 6.9 Hz, 1 H, H-3), 7.54 (d, $^3J_{H,H}$ = 7.0 Hz, 1 H, H-8), 7.71-7.74 (m, 1 H, H-7), 7.84 (d, $^3J_{H,H}$ = 8.3 Hz, 1 H, H-6), 7.93 (d, $^3J_{H,H}$ = 6.9 Hz, 1 H, H-4), 8.01-8.06 (m, 2 H, H-13, H-13‘). **^{13}C{^{1}H} NMR** (125.71 MHz, 305.0 K, CD_2Cl_2): δ = -0.9 (Si(CH_3)$_2$), 30.9 (CH_2), 97.8 (<u>C</u>-CN), 118.9 (C, C-5), 119.8, 120.1 (CH, C-3, C-14, C-14’, overlapping with CN), 121.0 (CH, C-6), 121.6 (CH, C-8), 132.8-125.7 (brs, C, [B(C_6F_5)$_4$]$^-$), 130.8 (CH, C-7), 134.7 (C, C-12), 137.0 (dm, $^1J_{C,F}$ = 242.2 Hz, CF, [B(C_6F_5)$_4$]$^-$), 138.8 (CH, C-4), 139.0 (dm, $^1J_{C,F}$ = 233.2 Hz, CF, [B(C_6F_5)$_4$]$^-$), 139.9 (d, $^3J_{C,F}$ = 10.3 Hz, C-13, C-13’), 146.8 (C, C-11), 148.9 (dm, $^1J_{C,F}$ = 241.0 Hz, CF, [B(C_6F_5)$_4$]$^-$), 149.4 (C, C-9), 154.9 (C, C-10), 170.4 (d, $^1J_{C,F}$ = 277.5 Hz, CF). **^{19}F{^{1}H} NMR** (470.28 MHz, 305.0 K, CD_2Cl_2): δ = -167.5-(-167.2) (m, 8 F, *m*-F, [B(C_6F_5)$_4$]$^-$), -163.44 (t, $^3J_{F,F}$ = 20.4 Hz, 4 F, *o*-F, [B(C_6F_5)$_4$]$^-$), -133.0-(-132.8) (m, 8 F, *o*-F, [B(C_6F_5)$_4$]$^-$), -86.6 (s, 1 F, CF). **^{29}Si{^{1}H} NMR** (99.31 MHz, 305.0 K, CD_2Cl_2): δ = 23.0. **^{11}B{^{1}H} NMR** (160.38 MHz, 305.1 K, CD_2Cl_2): δ = -16.4.

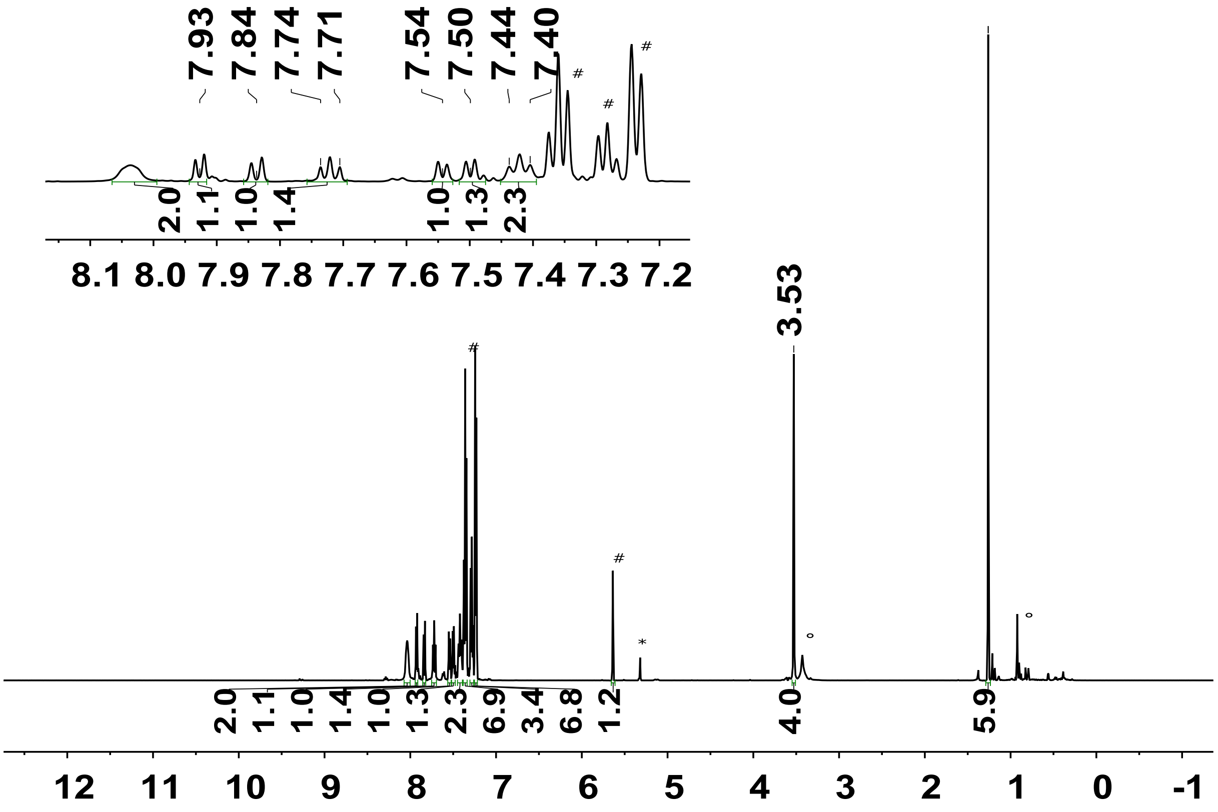

Figure S56 – ^{1}H NMR spectrum (499.87 MHz, 305.0 K, CD_2Cl_2, lb: -2.3 Hz, gb: 2.6, * $CDHCl_2$, # Ph_3CH, ° impurities/decomposition) of 6[B(C_6F_5)$_4$].

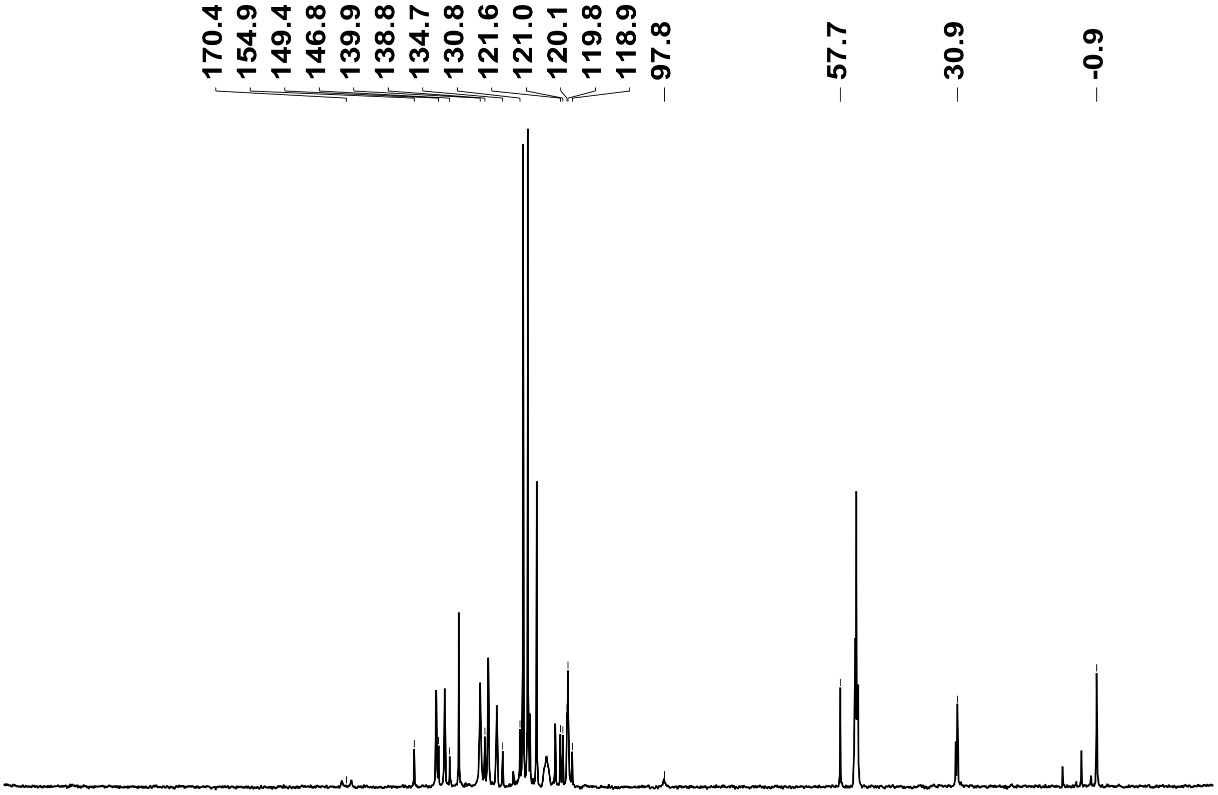

Figure S57 – $^{13}C\{^{1}H\}$ NMR spectrum (125.71 MHz, 305.0 K, CD_2Cl_2) of 6[B(C_6F_5)$_4$].

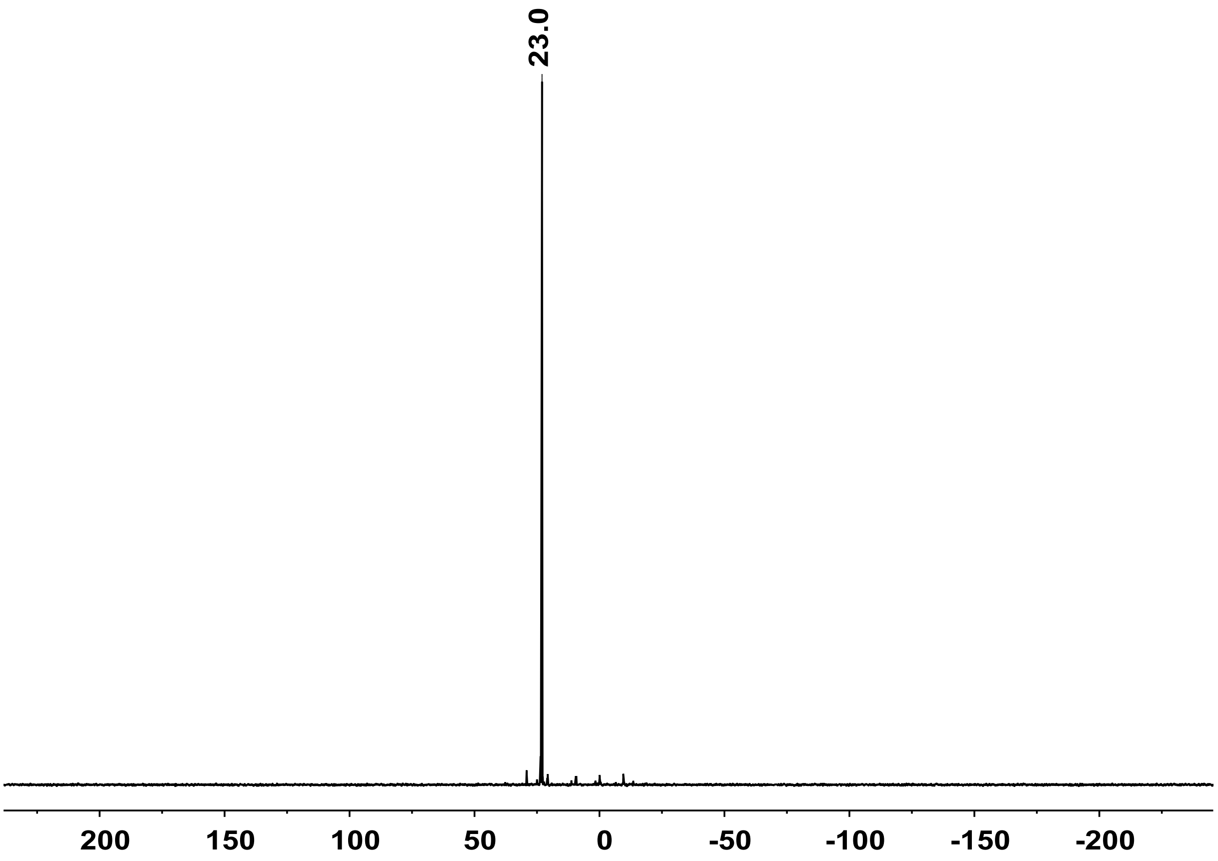

Figure S58 – $^{29}Si\{^1H\}$ INEPT NMR spectrum (99.31 MHz, 305.0 K, D3 = 0.0084, D4 = 0.0313, CD_2Cl_2) of 6[B(C_6F_5)$_4$].

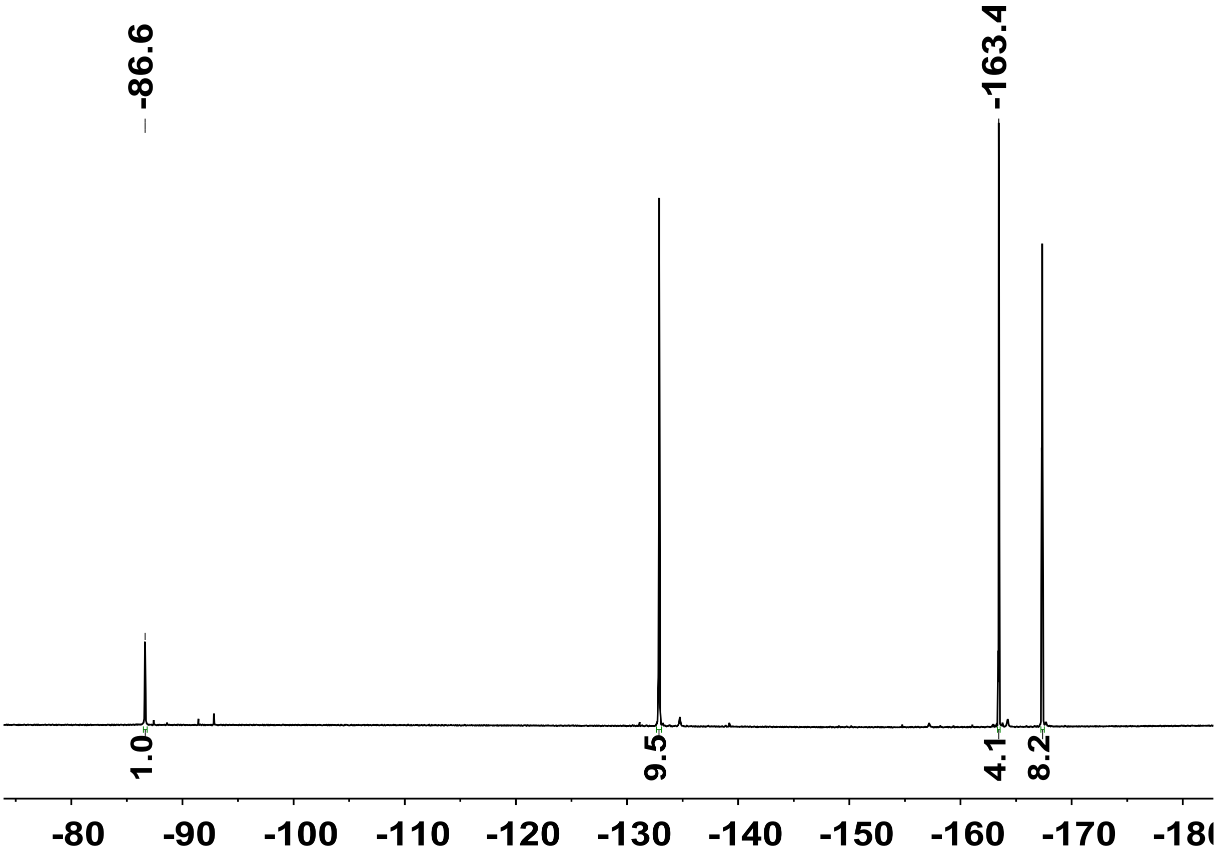

Figure S59 – $^{19}F\{^1H\}$ NMR spectrum (470.30 MHz, 305.0 K, CD_2Cl_2) of 6[B(C_6F_5)$_4$].

4-Fluorobenzonitrile + silylium borate 1[B(C_6F_5)$_4$]

4-Fluorobenzonitrile (1.0 equiv., 10.5 mg, 87 µmol) was added to a solution of silylium borate **1**[B(C_6F_5)$_4$] (1.0 equiv., 100 mg, 87 µmol) in chlorobenzene-d$_5$, stirred for 15 min at room temperature and analyzed by NMR spectroscopy.

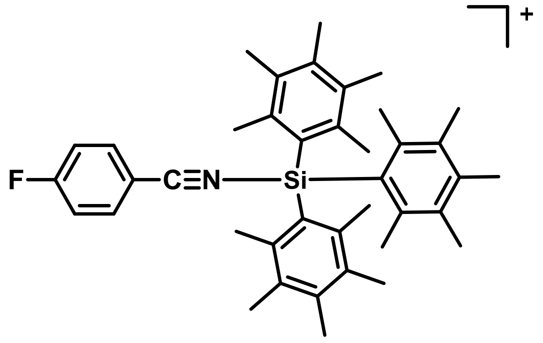

14 [B(C_6F_5)$_4$]$^-$

^{19}F{^{1}H} NMR (470.30 MHz, 305.0 K, C_6D_5Cl): δ = -167.4-(-167.3) (m, 8F, *m*-F, [B(C_6F_5)$_4$]), -163.4 (t, $^3J_{F,F}$ = 20.5 Hz, 4F, *p*-F, [B(C_6F_5)$_4$]$^-$), -132.9-(-132.8) (m, 8F, *o*-F, [B(C_6F_5)$_4$]$^-$), -87.3 (s, 1F, $^1J_{F,C}$ = 269.7 Hz, CF, F-Ph). **^{29}Si{^{1}H} NMR** (99.31 MHz, 305.0 K, C_6D_5Cl): δ = -2.2.

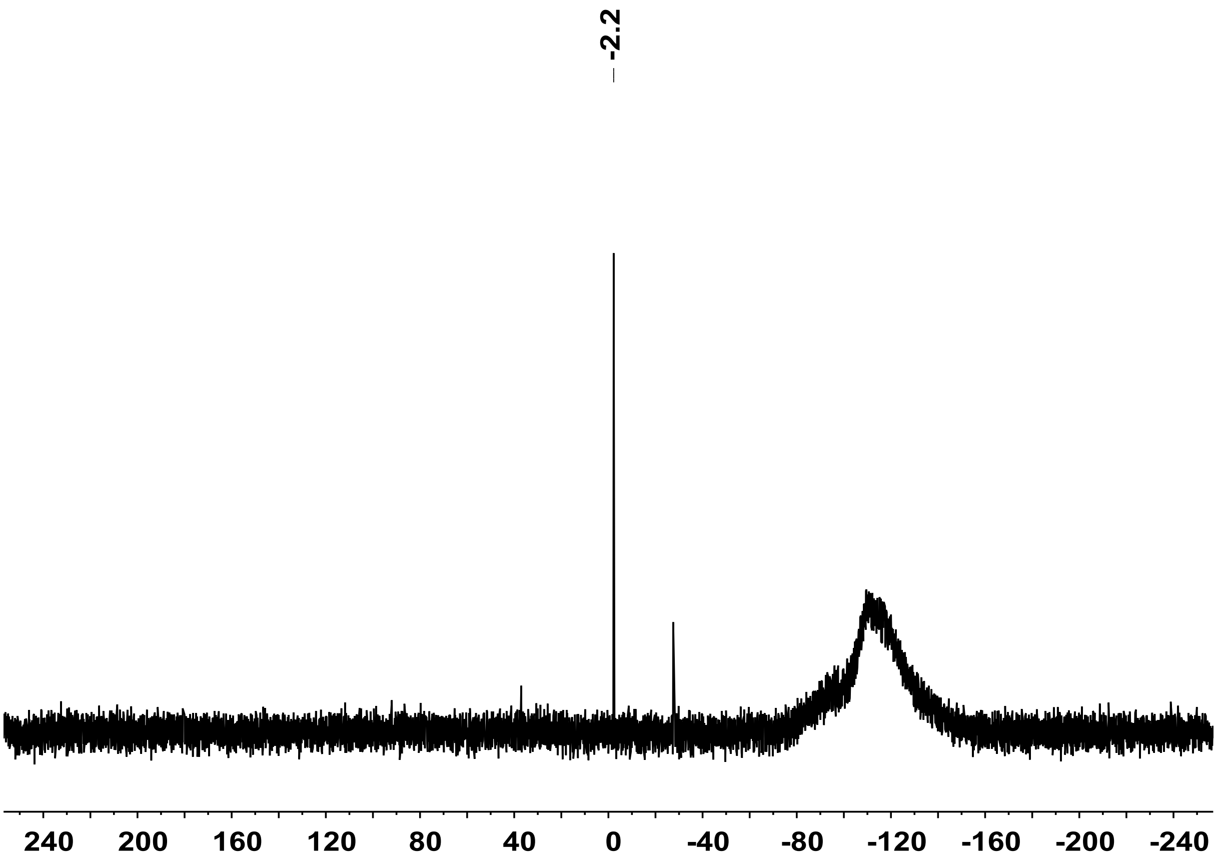

Figure S60 – ^{29}Si{^{1}H} NMR spectrum (99.31 MHz, 305.0 K, C_6D_5Cl) of 14[B(C_6F_5)$_4$].

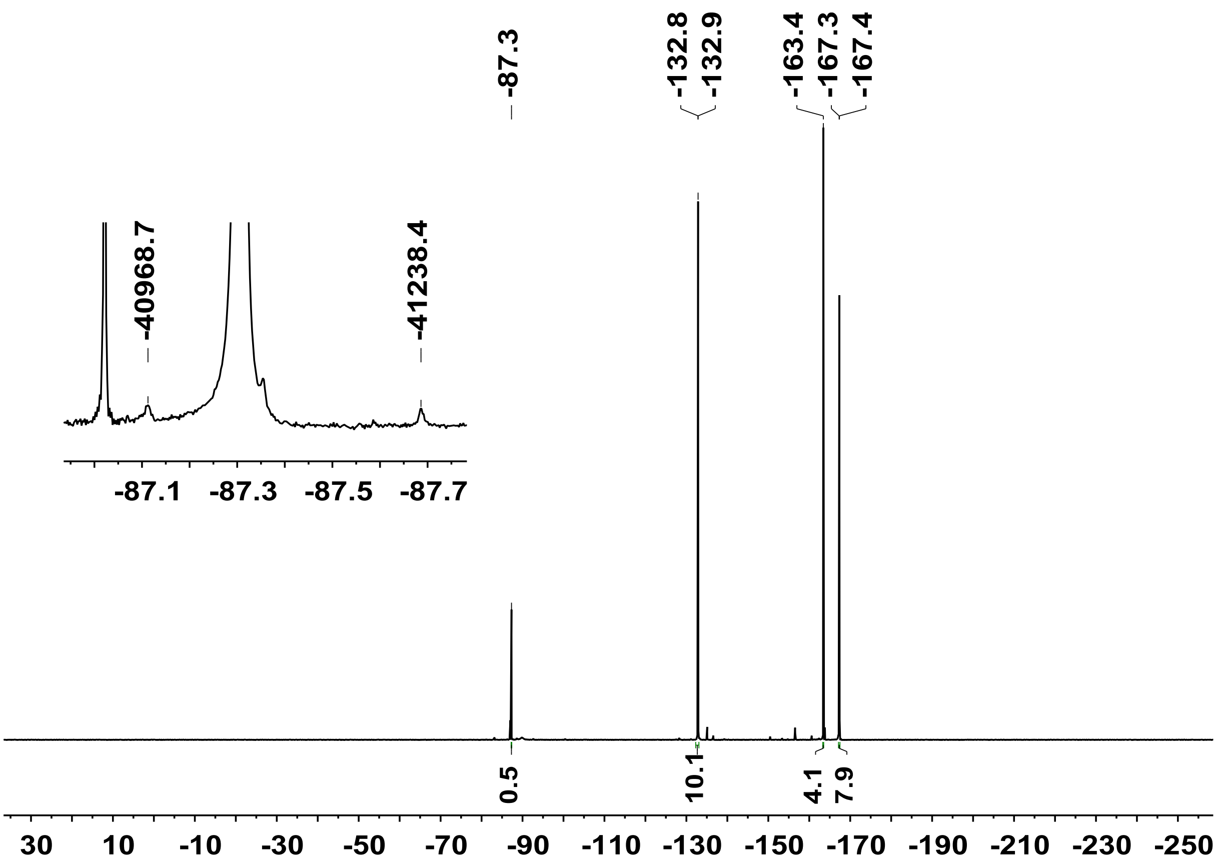

Figure S61 – $^{19}F\{^{1}H\}$ NMR spectrum (470.30 MHz, 305.0 K, C_6D_5Cl) of 14[$B(C_6F_5)_4$].

4-Fluorobenzonitrile + [$Et_3Si(C_7D_8)$][$B(C_6F_5)_4$]

Triethysilane (1.0 equiv., 0.07 mL, 421 µmol) was added at r.t. to a well stirred two-phase solution of trityl borate [Ph_3C][$B(C_6F_5)_4$] (1.0 equiv., 370 mg, 401 µmol) in toluene-d_8 and stirred for 15 min. After the biphasic reaction mixture was analyzed by NMR spectroscopy, the nonpolar phase was removed and 4-Fluorobenzonitrile (1.0 equiv., 48 mg, 401 µmol) was added. The reaction mixture was stirred for 15 min at room temperature. After removing the solvent under reduced pressure, the residue was dissolved in dichloromethane-d_2 and analyzed by NMR spectroscopy.

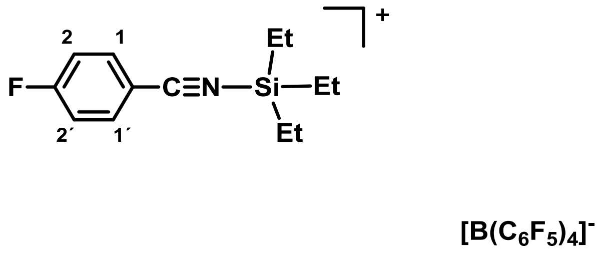

^{1}H NMR (499.87 MHz, 305.0 K, CD_2Cl_2): δ = 1.14-1.23 (m, 12H, Si(<u>CH</u>$_2$<u>CH</u>$_3$)$_3$), 7.43-7.46 (m, 2H, H-2, H-2'), 8.08-8.10 (m, 2H, H-1, H-1'). **^{13}C{^{1}H} NMR** (125.71 MHz, 305.0 K, CD_2Cl_2): δ = 4.4 (3 x CH_2), 6.2 (3 x CH_3), 99.0 (s, <u>C</u>-CN), 119.7 (d, $^{2}J_{C,F}$ = 23.7 Hz, CH, C-2, C-2'), 123.7-125.8 (brs, C, $[B(C_6F_5)_4]^-$), 124.2 (<u>C</u>N), 137.0 (dm, $^{1}J_{C,F}$ = 243.0 Hz, CF, $[B(C_6F_5)_4]^-$), 138.9 (dm, $^{1}J_{C,F}$ = 243.5 Hz, CF, $[B(C_6F_5)_4]^-$), 139.2 (d, $^{3}J_{C,F}$ = 11.0 Hz, CH, C-1, C-1'), 148.9 (dm, $^{1}J_{C,F}$ = 241.5 Hz, CF, $[B(C_6F_5)_4]^-$), 169.9 (d, $^{1}J_{C,F}$ = 269.9 Hz, CF, F-Ph). **^{19}F{^{1}H} NMR** (470.29 MHz, 305.1 K, CD_2Cl_2): δ = -167.8-(-167.4) (m, 8F, *m*-F, $[B(C_6F_5)_4]$), -163.4 (t, $^{3}J_{F,F}$ = 20.3 Hz, 4F, *p*-F, $[B(C_6F_5)_4]^-$), -133.2-(-132.8) (m, 8F, *o*-F, $[B(C_6F_5)_4]^-$), -87.9 (brs, 1F, CF, F-Ph). **^{29}Si{^{1}H} NMR** (99.31 MHz, 305.0 K, CD_2Cl_2): δ = 41.2. **^{11}B{^{1}H} NMR** (160.38 MHz, 305.0 K, CD_2Cl_2): δ = -16.6.

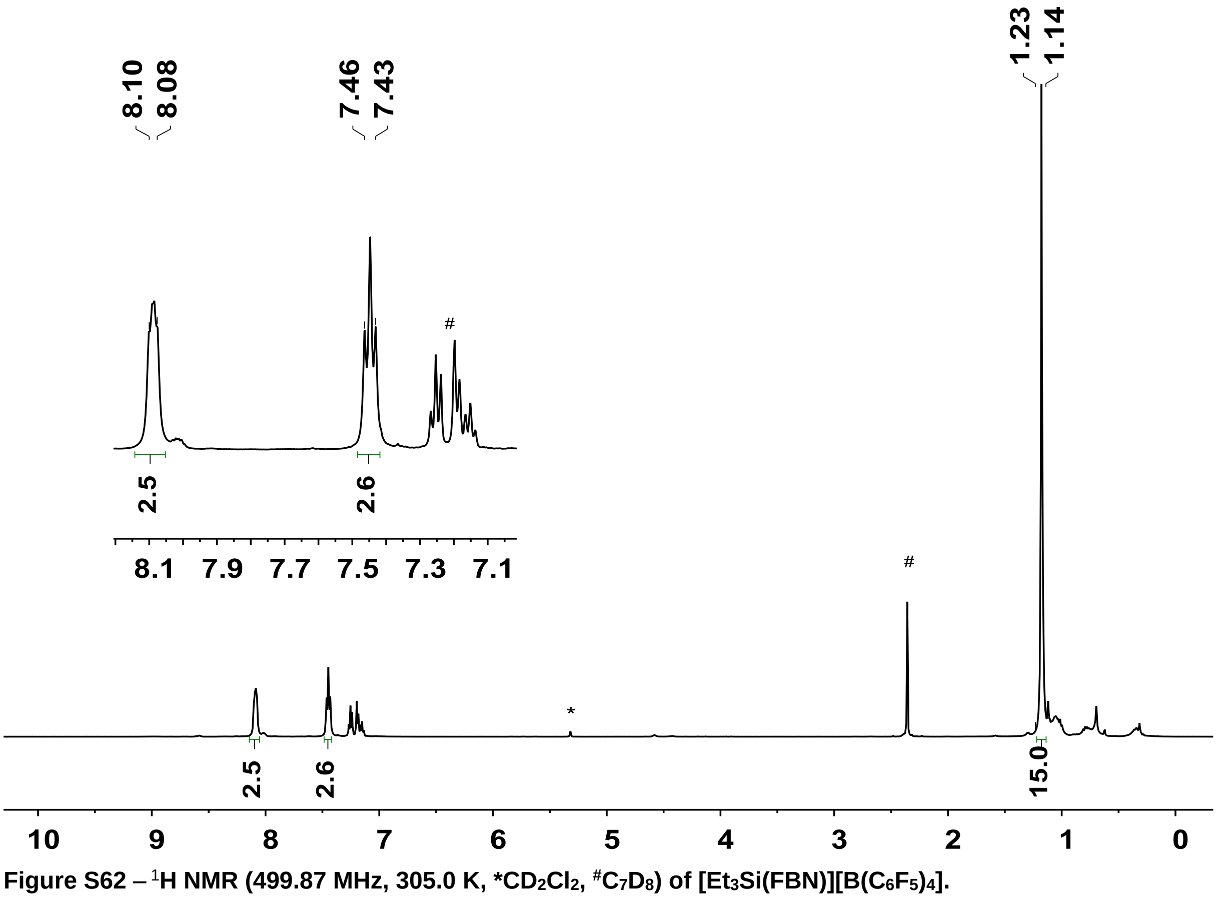

Figure S62 – **^{1}H NMR (499.87 MHz, 305.0 K, *CD_2Cl_2, #C_7D_8) of $[Et_3Si(FBN)][B(C_6F_5)_4]$.**

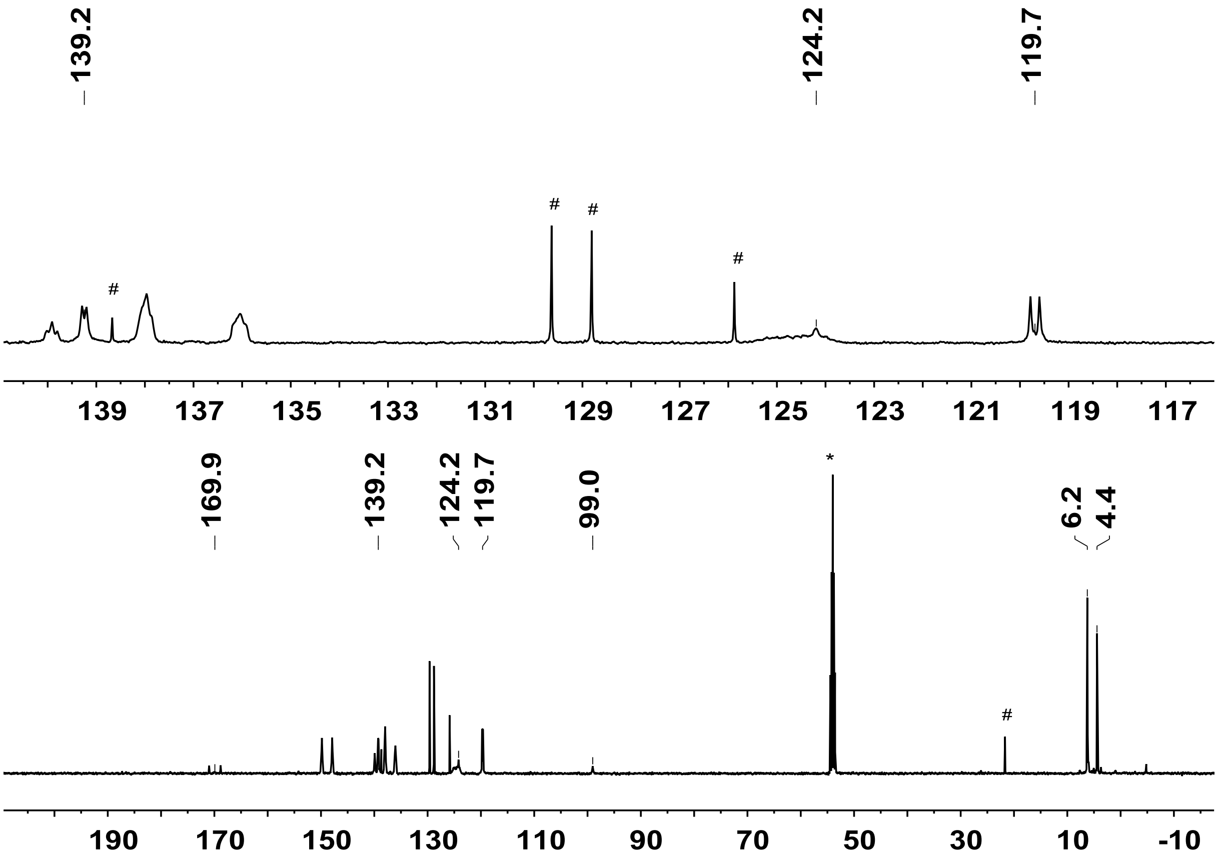

Figure S63 – $^{13}C\{^1H\}$ NMR spectrum (125.71 MHz, 305.0 K, *CD_2Cl_2, #C_6H_6) of [Et_3Si(FBN)][$B(C_6F_5)_4$].

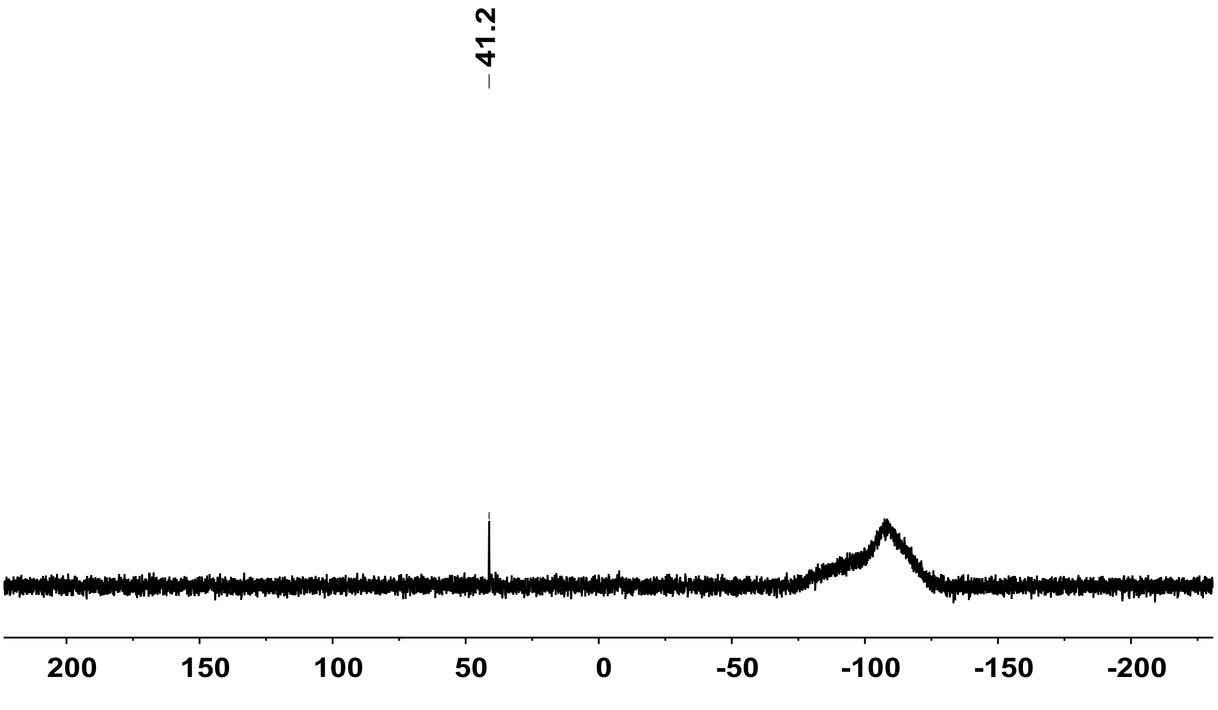

Figure S64 – $^{29}Si\{^1H\}$ NMR spectrum (99.31 MHz, 305.0 K, CD_2Cl_2) of [Et_3Si(FBN)][$B(C_6F_5)_4$].

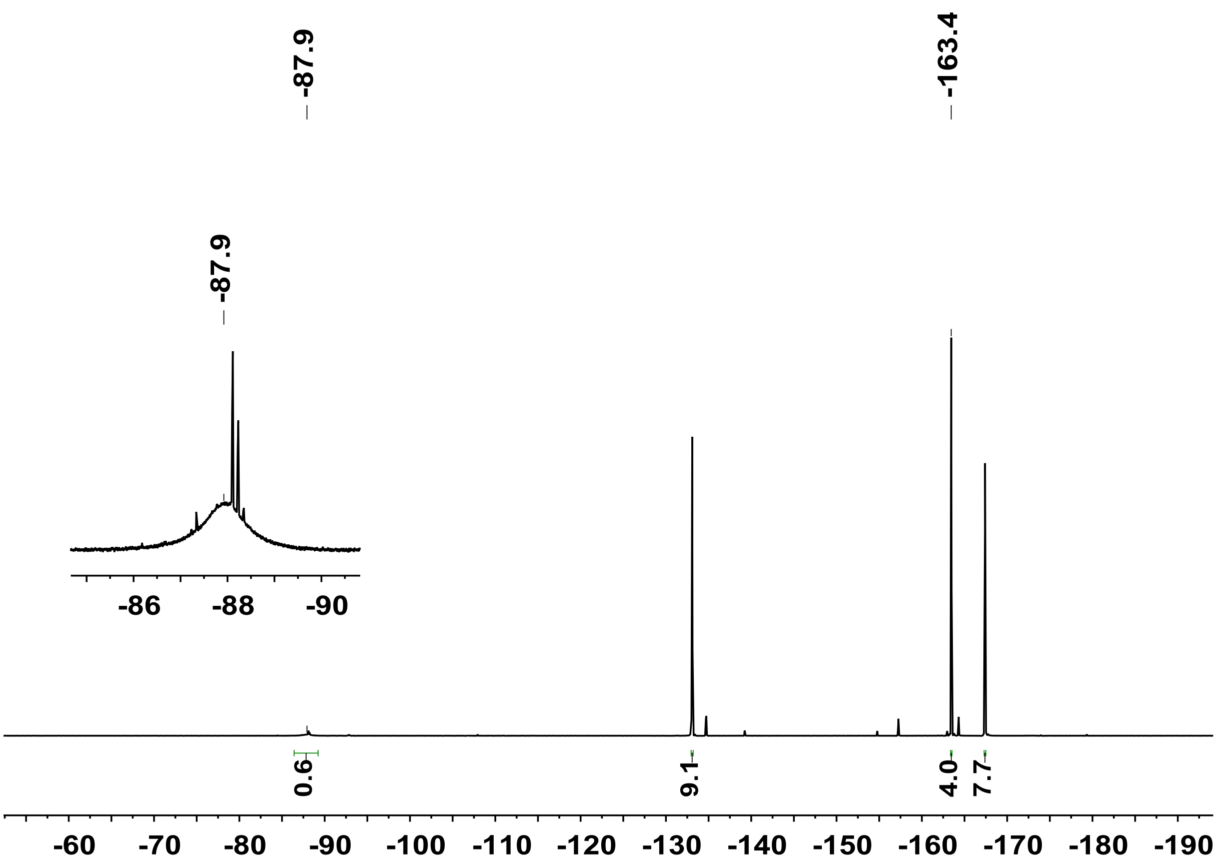

Figure S65 – $^{19}F\{^1H\}$ NMR spectrum (470.29 MHz, 305.1 K, CD_2Cl_2) of $[Et_3Si(FBN)][B(C_6F_5)_4]$.

1.4. 4-Fluorobenzonitrile + Tritylborate in CD_2Cl_2

The NMR sample was prepared using trityl borate (250.00 µmol, 230 mg) and 4-fluorobenzonitrile (250.00 µmol, 30 µL) in 0.7 mL dichloromethane-d2.

^{1}H NMR (499.87 MHz, 305.0 K, CD_2Cl_2): δ = 7.19-7.25 (m, 2 H, CN-Ar, *m*-CH), 7.69-7.72 (m, 8 H, $[CPh_3]^+$, *o*-CH, CN-Ar, *o*-CH), 7.89 (t, $^3J_{H,H}$ = 7.8 Hz, 6 H, $[CPh_3]^+$, *m*-CH), 8.28 (t, $^3J_{H,H}$ = 7.5 Hz, 3 H, $[CPh_3]^+$, *p*-CH). **$^{13}C\{^1H\}$ NMR** (125.71 MHz, 305.0 K, CD_2Cl_2): δ = 109.3 (C-CN), 117.4 (d, $^2J_{C,F}$ = 22.8 Hz, CN-Ar, *m*-CH), 118.7 (CN), 123.7-125.7 (brs, C, $[B(C_6F_5)_4]^-$), 131.2 ($[CPh_3]^+$, *m*-CH), 135.3 (d, $^3J_{C,F}$ = 9.4 Hz, CN-Ar, *o*-CH), 136.9 (dm, $^1J_{C,F}$ = 243.4 Hz, CF, $[B(C_6F_5)_4]^-$), 138.9 (dm, $^1J_{C,F}$ = 244.0 Hz, CF, $[B(C_6F_5)_4]^-$), 140.6 ($[CPh_3]^+$, *ipso*-C), 143.2 ($[CPh_3]^+$, *o*-CH), 144.1 ($[CPh_3]^+$, *p*-CH), 148.8 (d, $^1J_{C,F}$ = 239.7 Hz, CF, $[B(C_6F_5)_4]^-$), 165.7 (d, $^1J_{C,F}$ = 255.7 Hz, CF), 211.6 ([CPh3]$^+$). **$^{19}F\{^1H\}$ NMR** (470.28 MHz, 305.0 K, CD_2Cl_2): δ = -167.4-(-167.2) (m, 8 F, *m*-F, $[B(C_6F_5)_4]^-$), -163.44 (t, $^3J_{F,F}$ = 20.4 Hz, 4 F, *o*-F, $[B(C_6F_5)_4]^-$), -132.9-(-132.7) (m, 8 F, *o*-F, $[B(C_6F_5)_4]^-$), -103.4 (s, 1 F, CF). **$^{11}B\{^1H\}$ NMR** (160.38 MHz, 305.1 K, CD_2Cl_2): δ = -16.6.

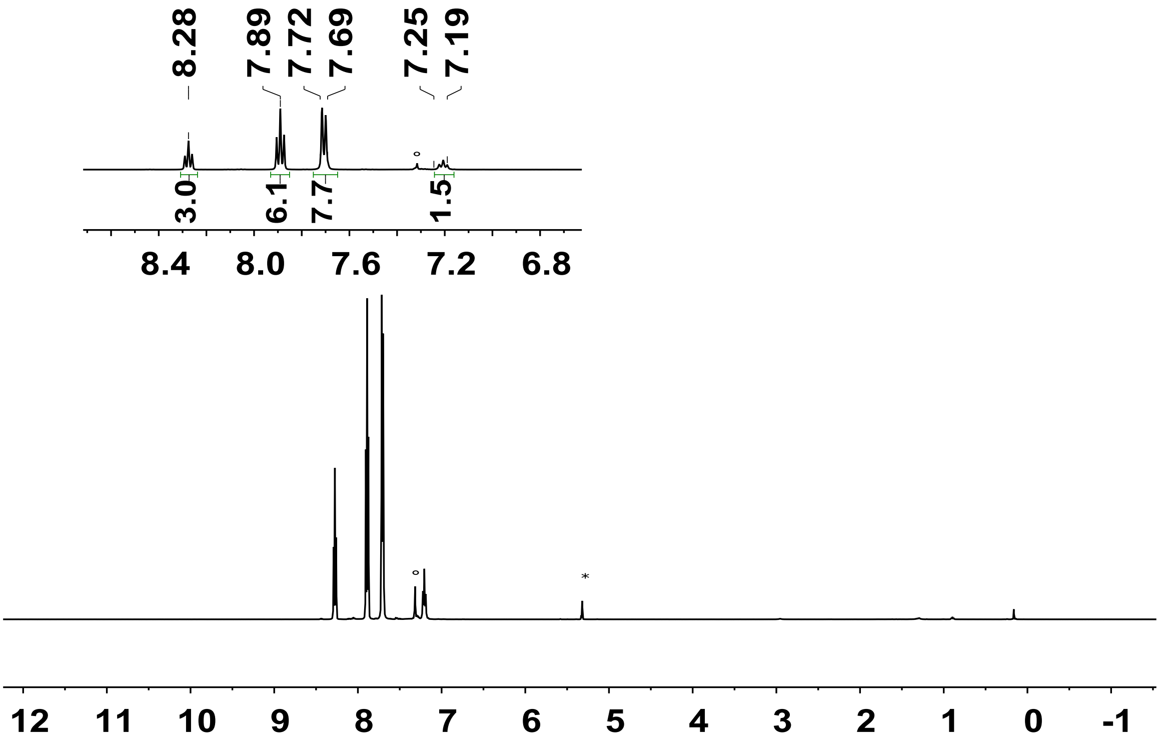

Figure S66 – ^{1}H NMR spectrum (499.87 MHz, 305.0 K, CD_2Cl_2, * $CDHCl_2$, ° impurities) of 4-Fluorobenzonitrile + Tritylborate.

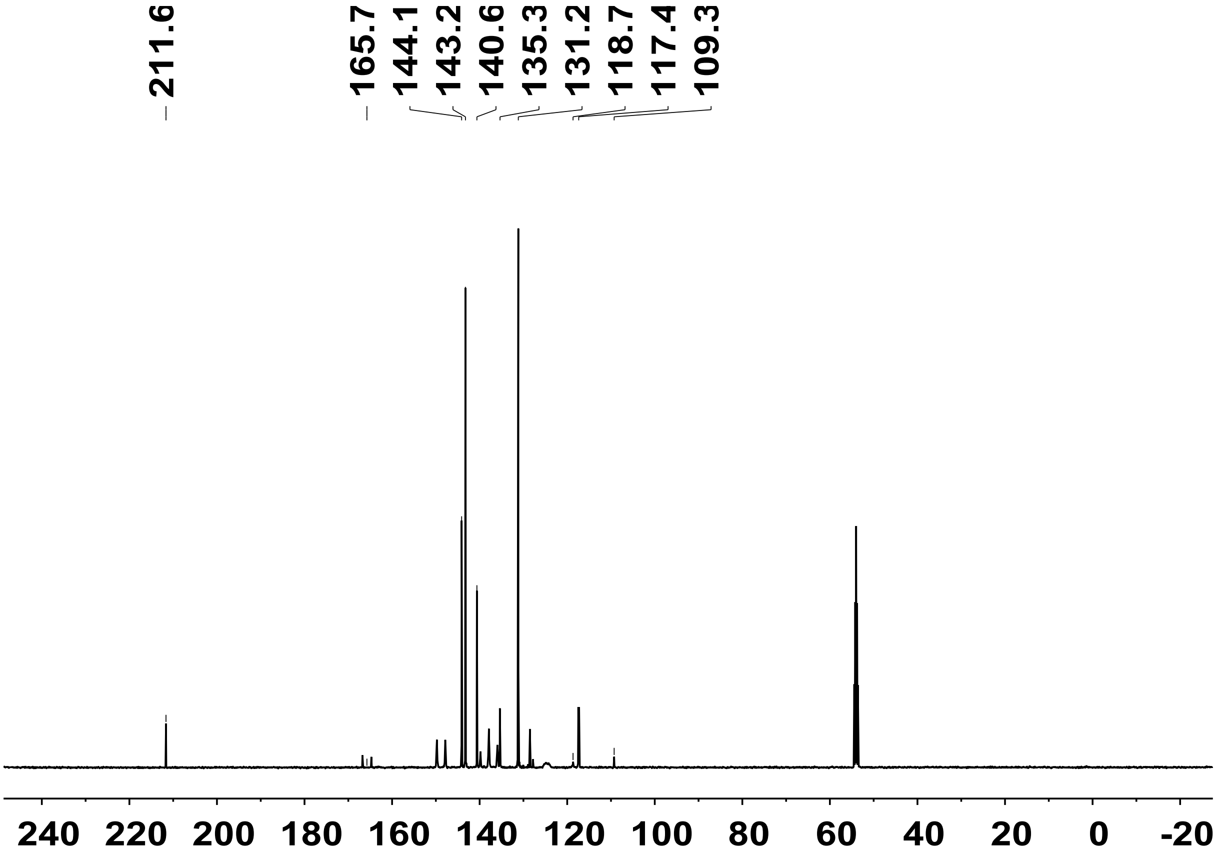

Figure S67 – $^{13}C\{^{1}H\}$ NMR spectrum (125.71 MHz, 305.0 K, CD_2Cl_2) of 4-Fluorobenzonitrile + Tritylborate.

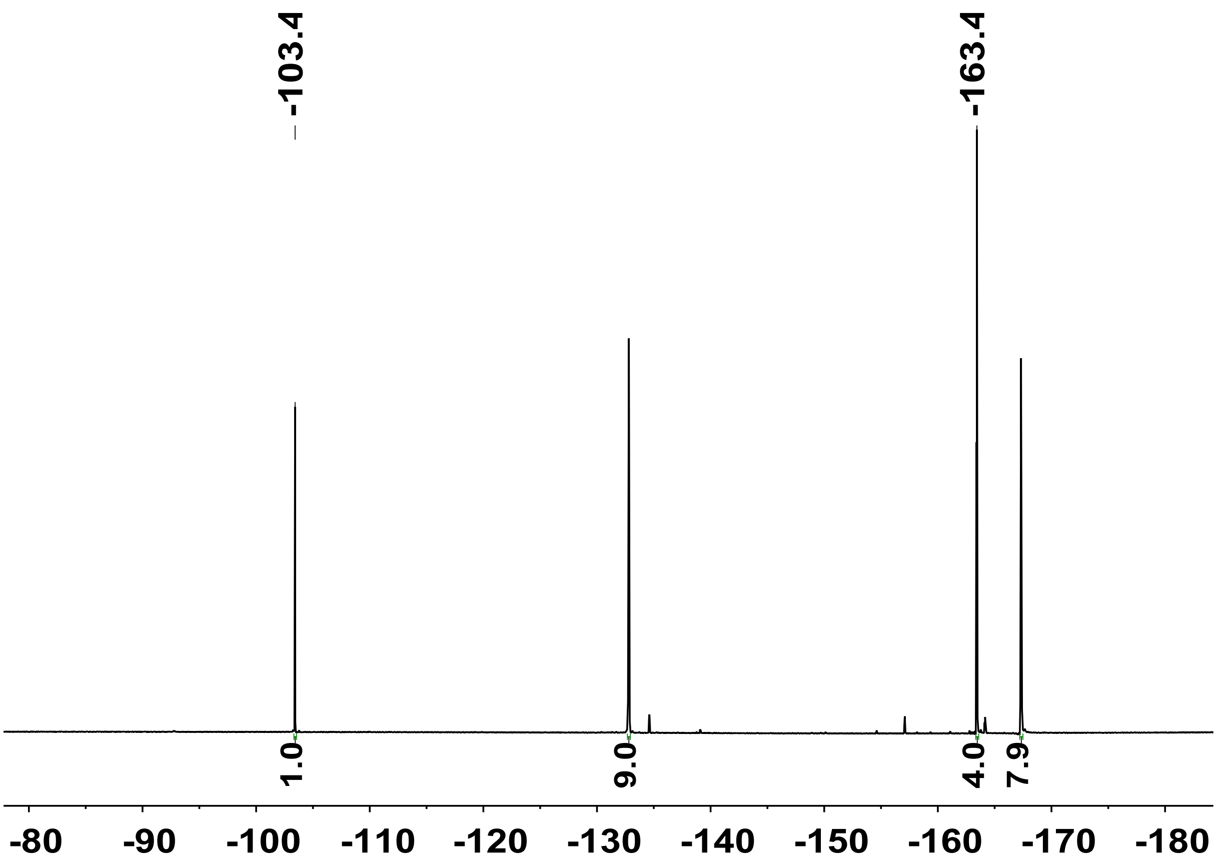

Figure S68 – $^{19}F\{^1H\}$ NMR spectrum (470.30 MHz, 305.0 K, CD_2Cl_2) of 4-Fluorobenzonitrile + Tritylborate.

1.5. Tris(pentafluorophenyl)borane 4-fluorobenzonitrile adduct 4

A Schlenk tube was charged with 94 mg (183.60µmol) tris(pentafluorophenyl)borane, 22 mg (183.60 µmol) 4-fluorobenzonitrile and 52 mg (56.38 µmol) trityl borate. The solids were dissolved in 0.7 mL CD_2Cl_2 and the mixture was stirred for 20 min at r.t.. Then the mixture was transferred to a NMR tube for analysis. Trityl borate was added as internal reference for the ^{19}F NMR spectroscopy.

^1H NMR (499.87 MHz, 305.0 K, CD_2Cl_2): δ = 7.35-7.43 (brm, 2 H, CN-Ar, *m*-CH), 7.69 (d, $^3J_{H,H}$ = 7.5 Hz, $[CPh_3]^+$, *o*-CH), 7.89 (t, $^3J_{H,H}$ = 7.8 Hz, $[CPh_3]^+$, *m*-CH), 7.94-7.05 (brm, 2 H, CN-Ar, *o*-CH), 8.28 (t, $^3J_{H,H}$ = 7.5 Hz, $[CPh_3]^+$, *p*-CH). **$^{13}C\{^1H\}$ NMR** (125.71 MHz, 305.0 K, CD_2Cl_2): δ = 102.0 (NC-Ar), 114.5 (NC-Ar), 115.7 (NC-Ar), 119.0 (d, $^2J_{C,F}$ = 13.5 Hz, NC-Ar), 123.4-125.5 (brs, C, $[B(C_6F_5)_4]^-$), 131.2 ($[CPh_3]^+$, CH), 136.8 (dm, $^1J_{C,F}$ = 241.4 Hz, CF, $[B(C_6F_5)_4]^-$), 138.0 (dm, $^1J_{C,F}$ = 249.0 Hz, C_6F_5, *m*-CF), 138.8 (dm, $^1J_{C,F}$ = 247.6 Hz, CF, $[B(C_6F_5)_4]^-$), 140.6 ($[CPh_3]^+$, CH), 141.1 (dm, $^1J_{C,F}$ = 251.0 Hz, C_6F_5, *p*-CF), 143.3 ($[CPh_3]^+$, CH), 144.2 ($[CPh_3]^+$, CH), 148.8 (d,

$^{1}J_{C,F}$ = 240.8 Hz, CF, $[B(C_6F_5)_4]^-$, C_6F_5, *o*-CF), 168.7 (d, $^{1}J_{C,F}$ = 269.9 Hz, NC-Ar, CF), 211.5 ([<u>C</u>Ph$_3$]$^+$). **^{11}B{^{1}H} NMR** (160.38 MHz, 305.0 K, CD_2Cl_2): δ = -10.0 (N-B), -16.6 ($[B(C_6F5)_4]^-$]. **^{19}F{^{1}H} NMR** (470.28 MHz, 193.0 K, CD_2Cl_2): δ = -167.4-(-167.2) (m, *m*-F, $[B(C_6F_5)_4]^-$), -164.0-(-163.9) (m, 6 F, *m*-C_6F_5), -163.44 (t, $^{3}J_{F,F}$ = 20.3 Hz , *o*-F, $[B(C_6F_5)_4]^-$), -156.8 (t, $^{3}J_{F,F}$ = 20.2 Hz, 3 F, *p*-C_6F_5), -134.5-(-134.3) (m, 6 H, *o*-C_6F_5), -132.9-(-132.5) (m, *o*-F, $[B(C_6F_5)_4]^-$), -92.5 (s, 1 F, CF).

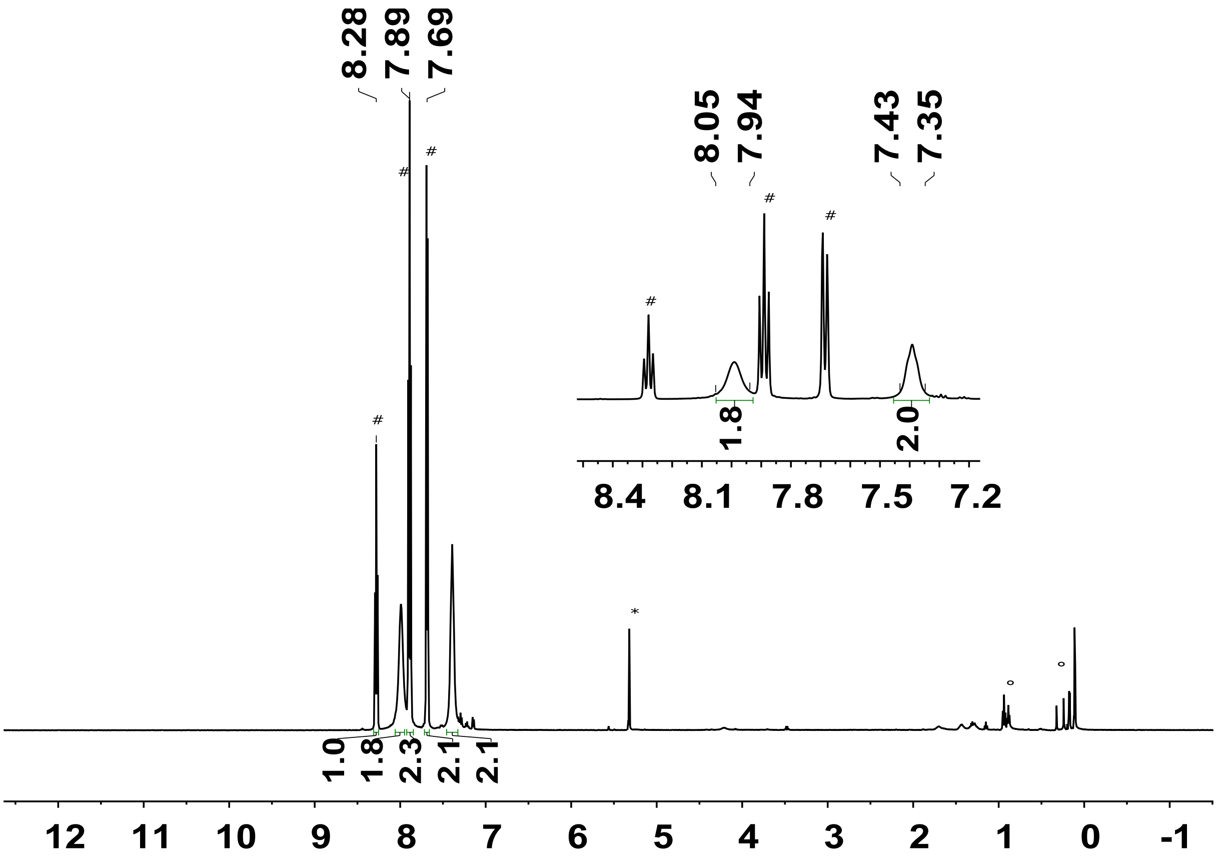

Figure S69 – ^{1}H NMR spectrum (499.87 MHz, 305.0 K, CD_2Cl_2, * $CDHCl_2$, # trityl borate, ° impurities) of 4[$B(C_6F_5)_4$].

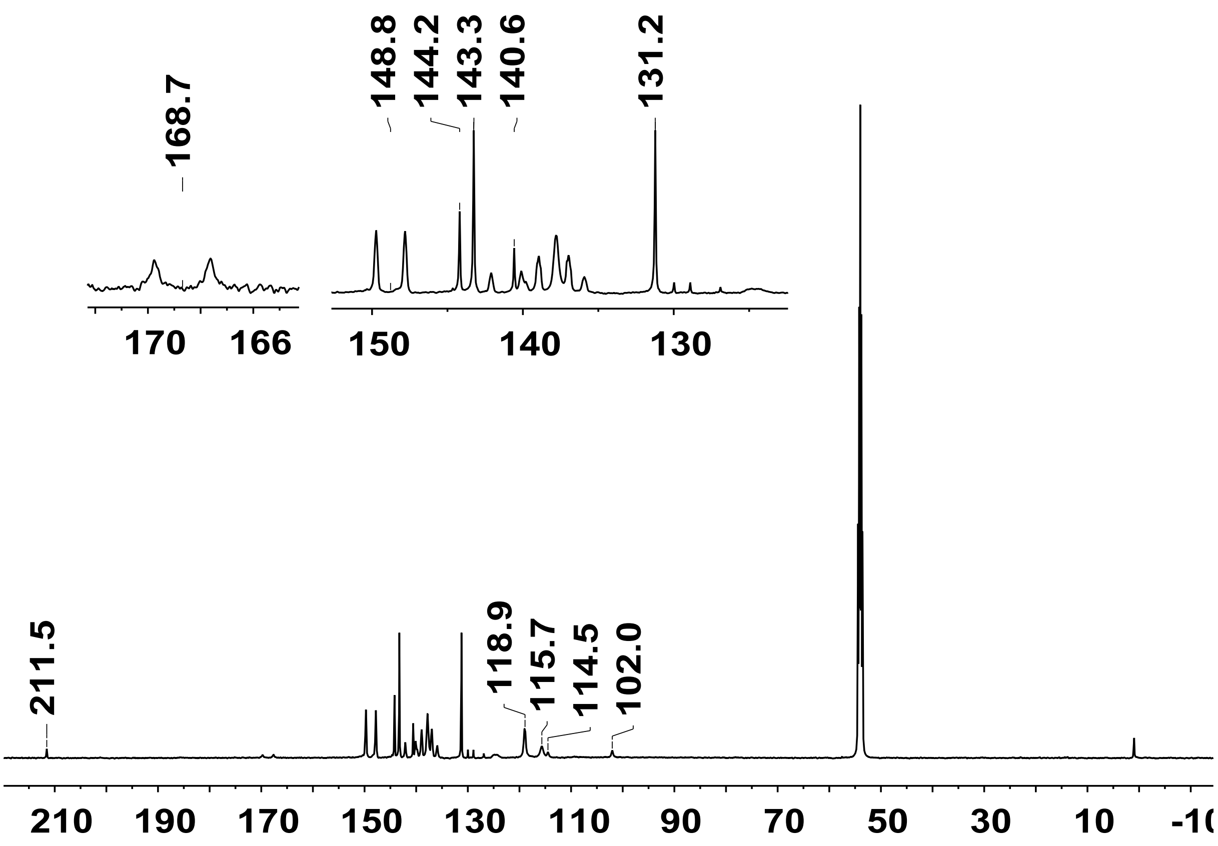

Figure S70 – $^{13}C\{^1H\}$ NMR spectrum (125.85 MHz, 300.0 K, CD_2Cl_2) of 4[B(C_6F_5)$_4$].

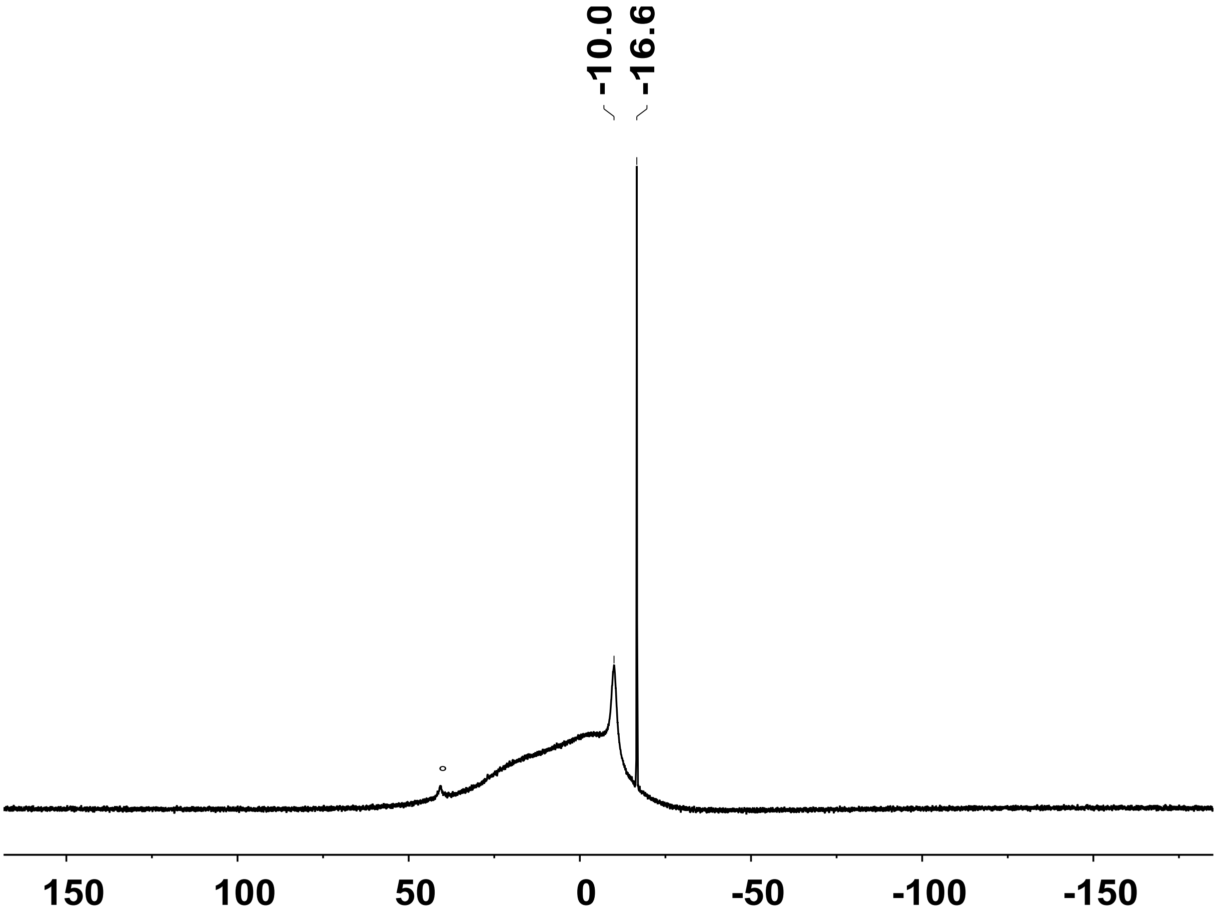

Figure S71 – $^{11}B\{^1H\}$ NMR spectrum (160.38 MHz, 305.0 K, CD_2Cl_2, ° impurity) of 4[B(C_6F_5)$_4$].

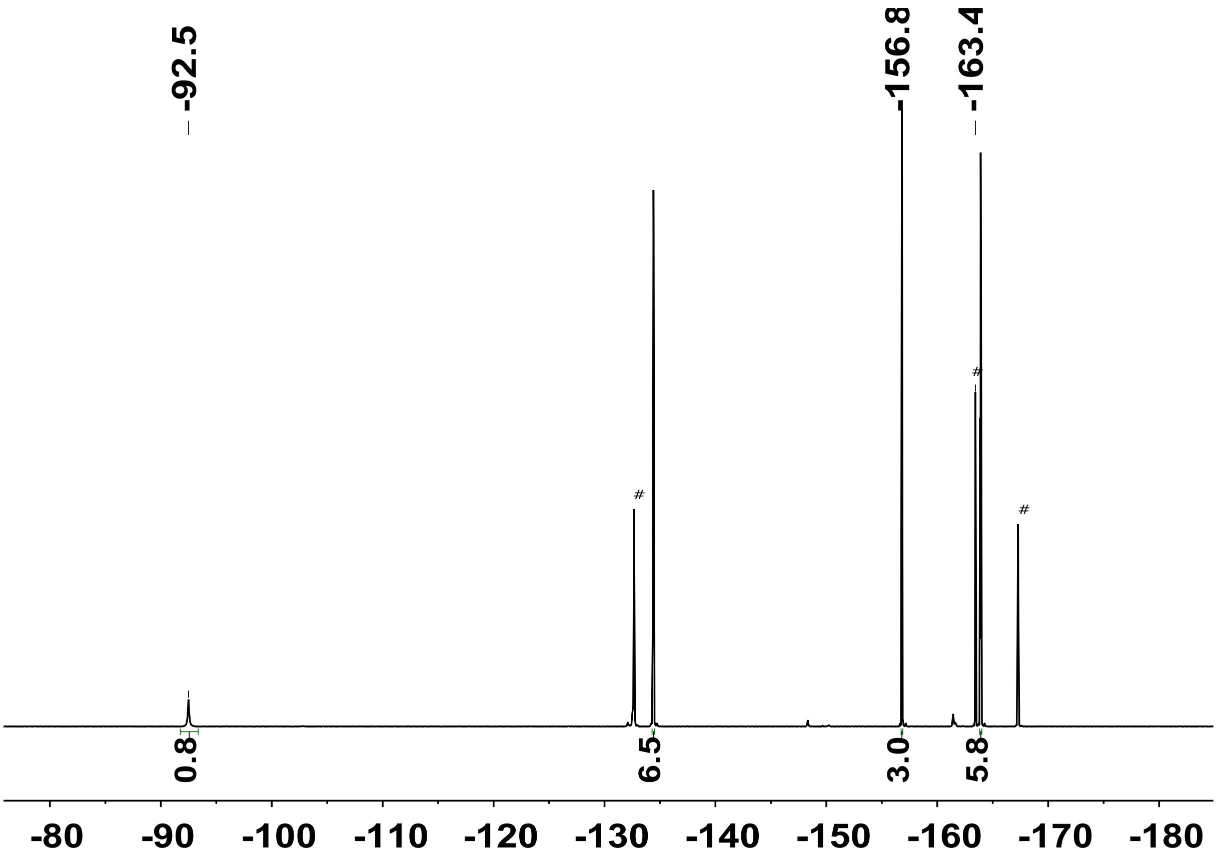

Figure S72 – ^{19}F{^{1}H} NMR spectrum (470:29 MHz, 305.0 K, D1 = 10 s CD_2Cl_2, # trityl borate) of 4[B(C_6F_5)$_4$].

1.6. Gutmann-Beckett method for silyl cation 2c

A Schlenk tube was charged with 155 mg (0.421 mmol) selenyl silane **7c**. In another Schlenk tube 380 mg (0.401 mmol) trityl borate $[Ph_3C][B(C_6F_5)_4]$ was dissolved in deuterated benzene. This solution was added to the silane and stirred for 30 min. The biphasic reaction mixture was transferred into an NMR tube and analyzed by NMR spectroscopy. The solution of **2c** was then added to a Schlenk tube, charged with 54 mg (0.401 mmol) triethylphosphane oxide. After stirring for one hour, the mixture was transferred into an NMR tube for analysis.

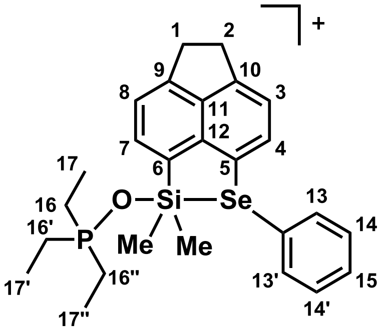

2c($OPEt_3$) $[B(C_6F_5)_4]^-$

^{29}Si{1H} NMR (99.31 MHz, 305.0 K, C_6D_6): δ = 9.4 ($^1J_{Si,P}$ = 15.2 Hz). **^{33}P{1H} NMR** (202.35 MHz, 305.1 K, C_6D_6): δ = 86.0. **^{77}Se{1H} NMR** (95.36 MHz, 305.0 K, C_6D_6): δ = 348.0.

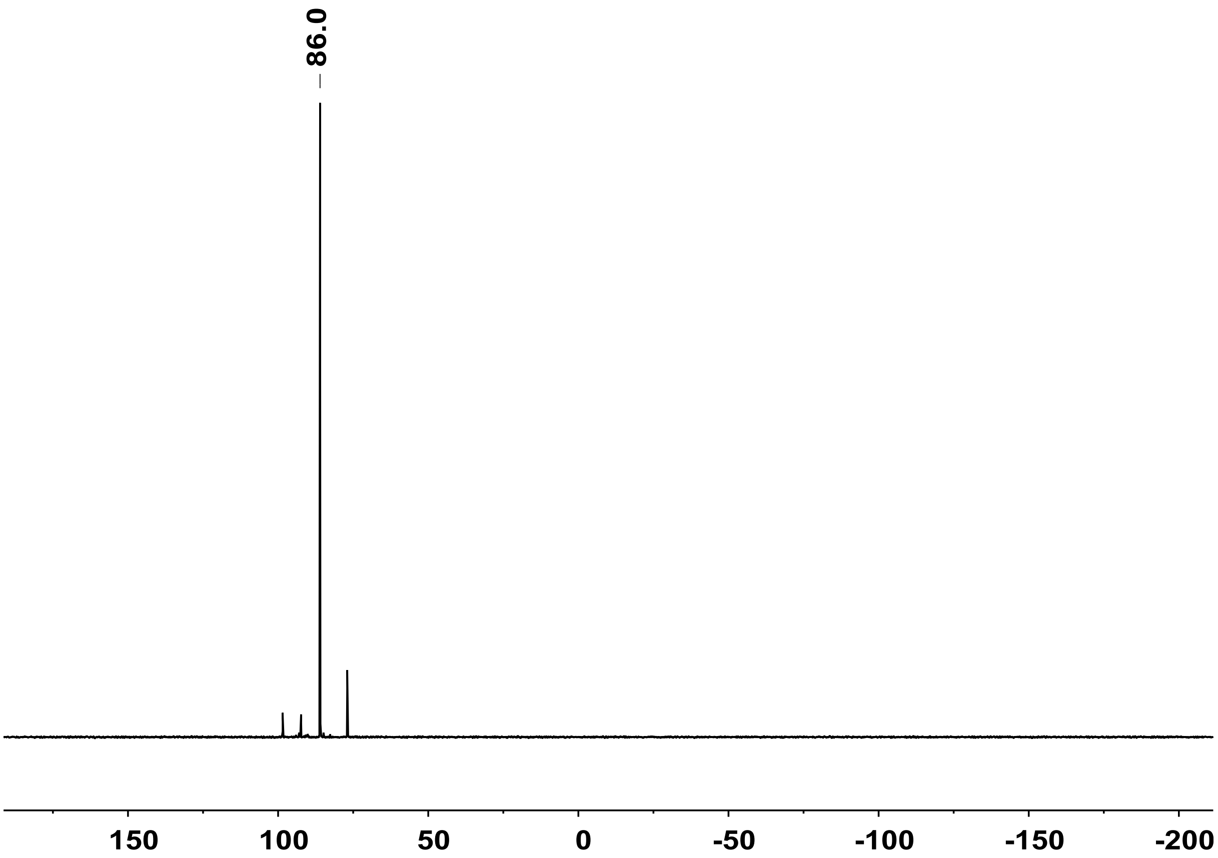

Figure S73 – $^{31}P\{^1H\}$ NMR spectrum (202.35 MHz, 305.1 K, C_6D_6) of $2c(OPEt_3)[B(C_6F_5)_4]$.

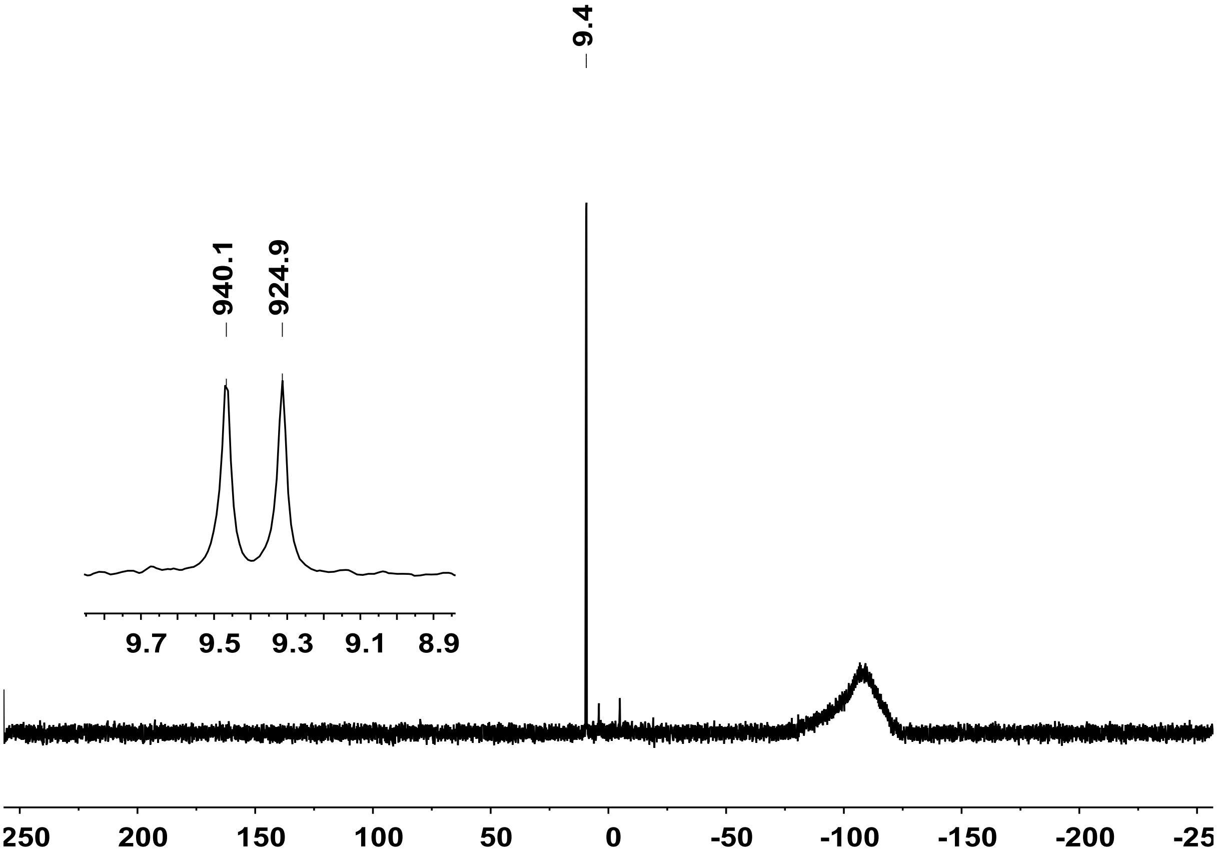

Figure S74 – $^{29}Si\{^1H\}$ NMR spectrum (99.31 MHz, 305.0 K, C_6D_6) of $2c(OPEt_3)[B(C_6F_5)_4]$.

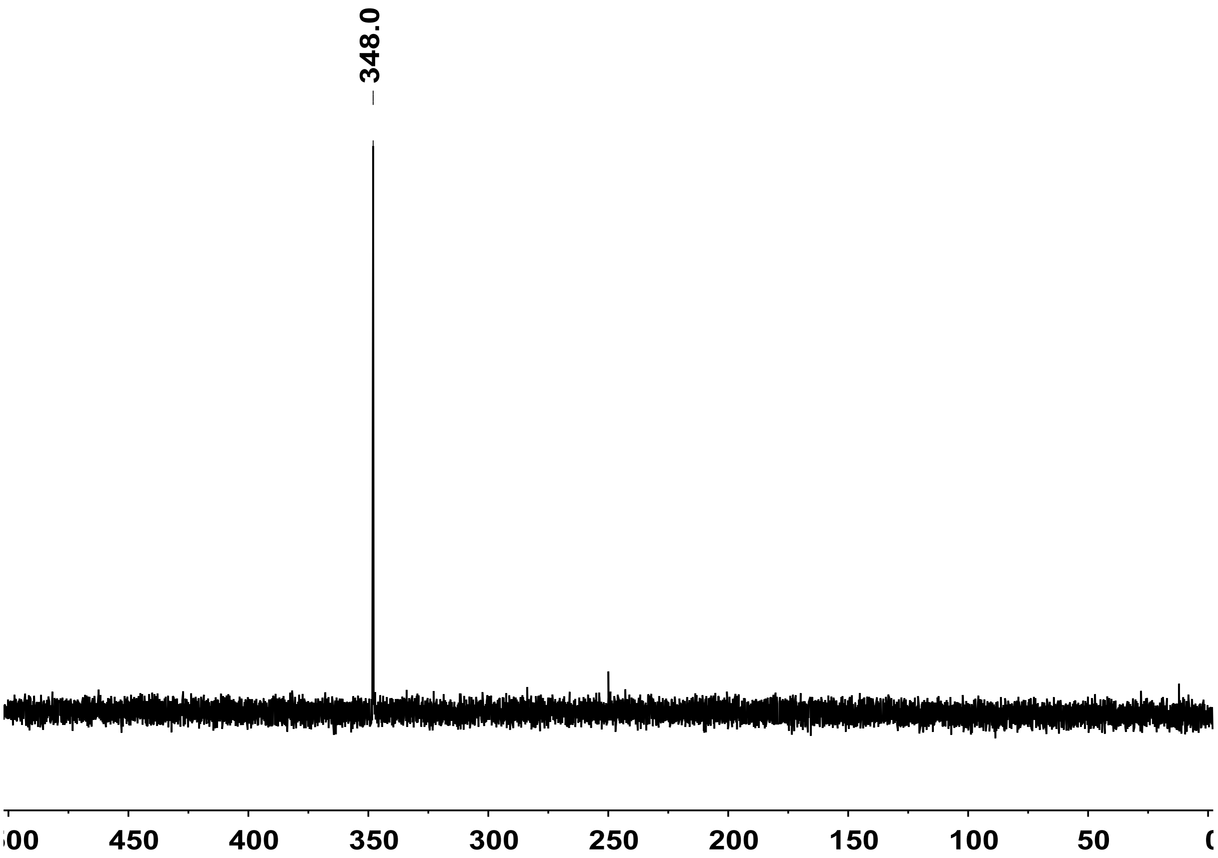

Figure S75 – ^{77}**Se{**^{1}**H} NMR spectrum (95.36 MHz, 305.0 K,** C_6D_6**) of 2c(**$OPEt_3$**)[**$B(C_6F_5)_4$**].**

1.7. Variable temperature NMR measurements of nitrilium ion 10c with an excess 4-fluorobenzonitrile

To the NMR solution of **10c**[$B(C_6F_5)_4$] (stoichiometric) was added another equivalent 4-fluorobenzonitrile (49 mg, 400 µmol). The resulting solution was then analyzed by NMR spectroscopy at various temperatures. The signal of the *p*-fluorine atoms of $[B(C_6F_5)_4]^-$ was referenced against the corresponding values in Table S1. At high temperatures the ^{19}F NMR signal of the time averaged minimum was detected, while at lower temperature it splits into the signal of the complex and free FBN.

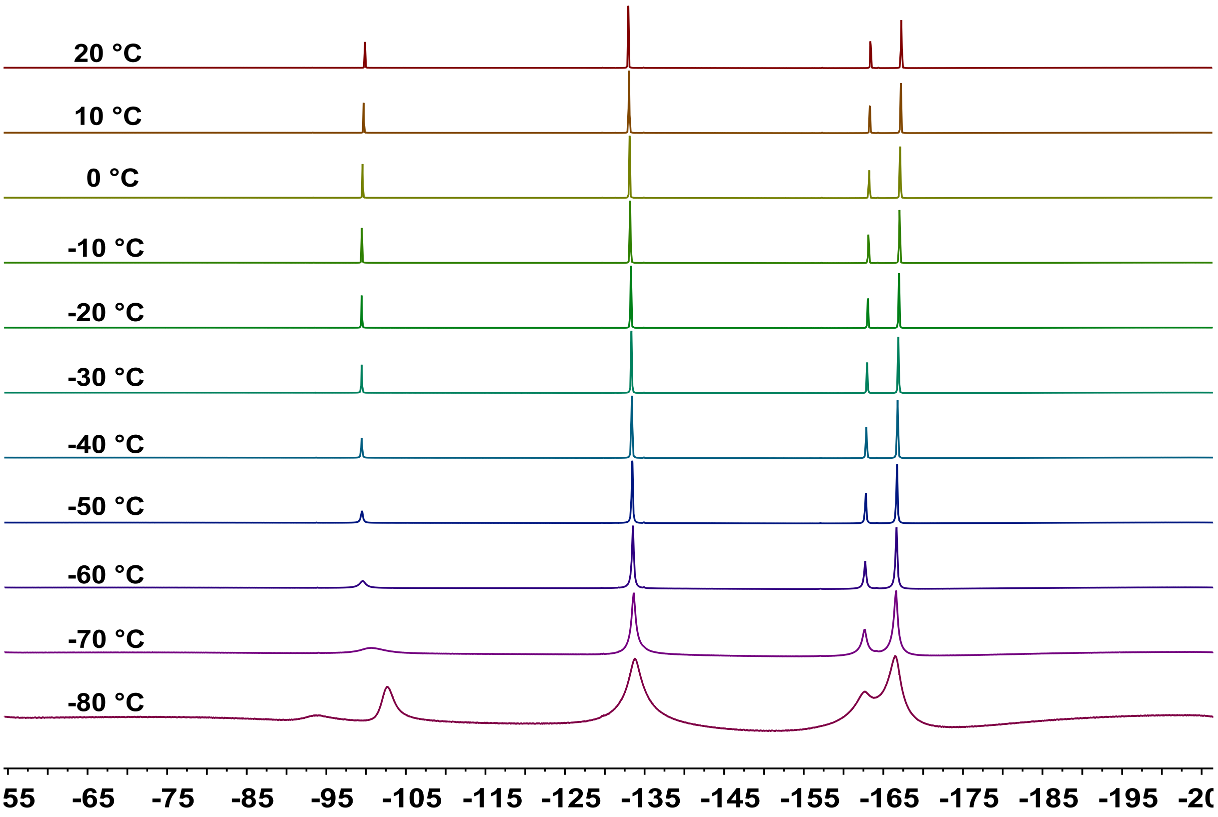

Figure S76 – VT ^{1}H NMR spectra (470.29 MHz, CD_2Cl_2) of 10c[$B(C_6F_5)_4$] with an excess 4-fluorobenzonitrile.

1.8. Reaction of bromonium borate 2e[$B(C_6F_5)_4$] with silane 7c

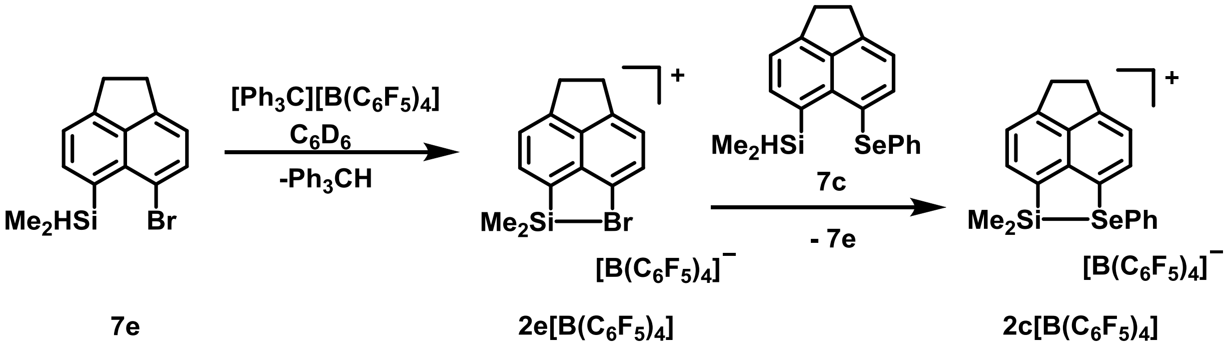

A Schlenk tube was charged with silane **7e** (1.1 equiv., 128 mg, 441 µmol) and trityl borate [Ph_3C][$B(C_6F_5)_4$] (1.0 equiv., 370 mg, 401 µmol). The solids were dissolved in benzene-d_6 and the solution of the silane was slowly added to the solution of trityl borate while cooling at 5 °C. Then the reaction mixture was stirred at r.t. for 30 min. The phases were separated, the upper, nonpolar phase was removed and the polar phase was transferred into an NMR tube and analyzed by NMR spectroscopy.

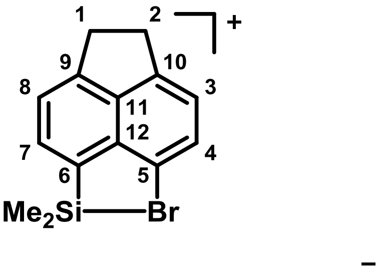

2e

^{1}H NMR (500.13 MHz, 297.7 K, C_6D_6): δ = 0.61 (s, 6H, Si(<u>CH</u>$_3$)$_2$), 2.93-3.01 (m, 4H, 2 x CH_2), 6.89 (d, 1H, $^3J_{H,H}$ = 7.4 Hz), 6.98 (d, 1H, $^3J_{H,H}$ = 7.4 Hz), 7.10-7.14 (m, 1H, H-8, overlapping with Ph_3CH), 7.25 (d, 1H, $^3J_{H,H}$ = 6.9 Hz, H-7).

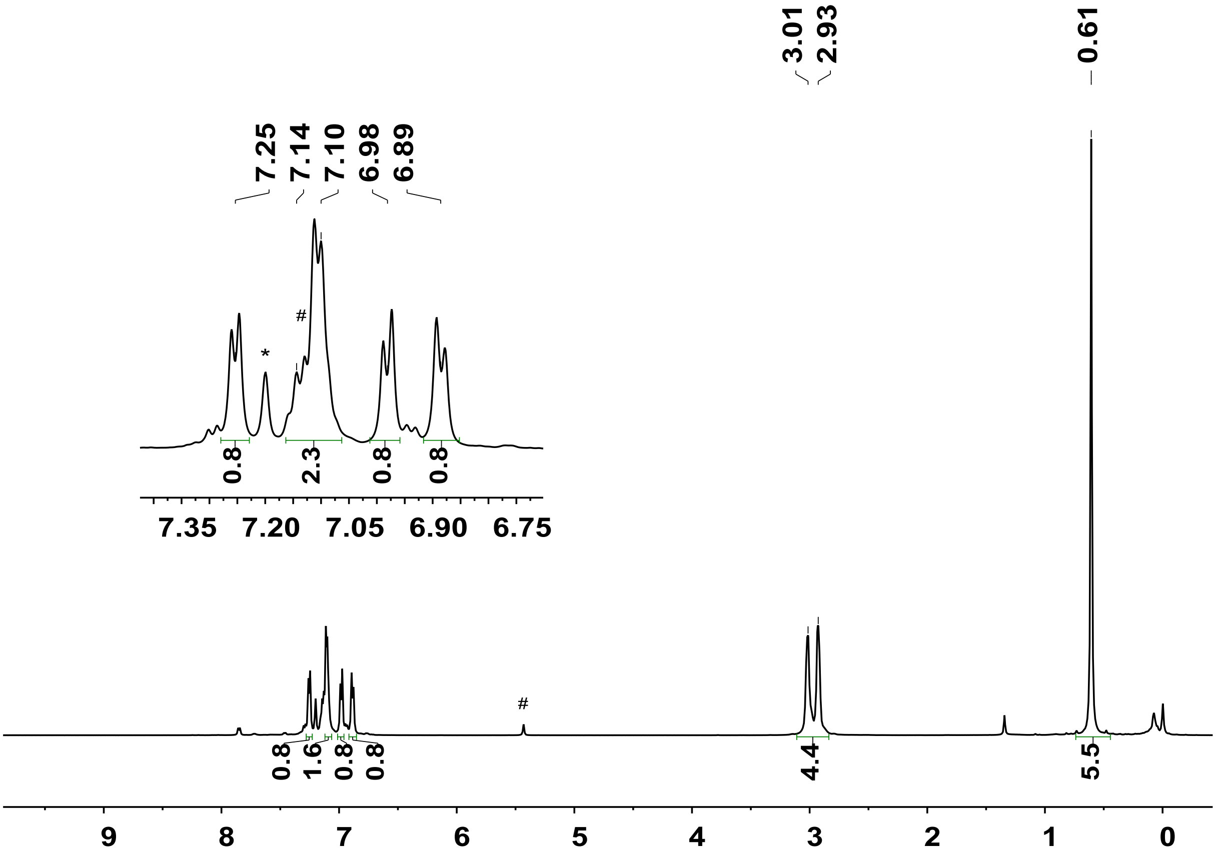

Figure S77 – ^{1}H NMR (500.13 MHz, 297.7 K, *C_6D_6, $^\#Ph_3CH$) of 2e[B(C_6F_5)$_4$].

The solution of silyl borate **2e**[B(C_6F_5)$_4$] was then added to a Schlenk tube, charged with silane **7c** (1.0 equiv., 148 mg, 401 µmol). After stirring for 30 min, the mixture was transferred into an NMR tube for analysis. The obtained NMR spectroscopic data indicated the formation of silyl borate **2c**[B(C_6F_5)$_4$] [S6] and silane **7e**.

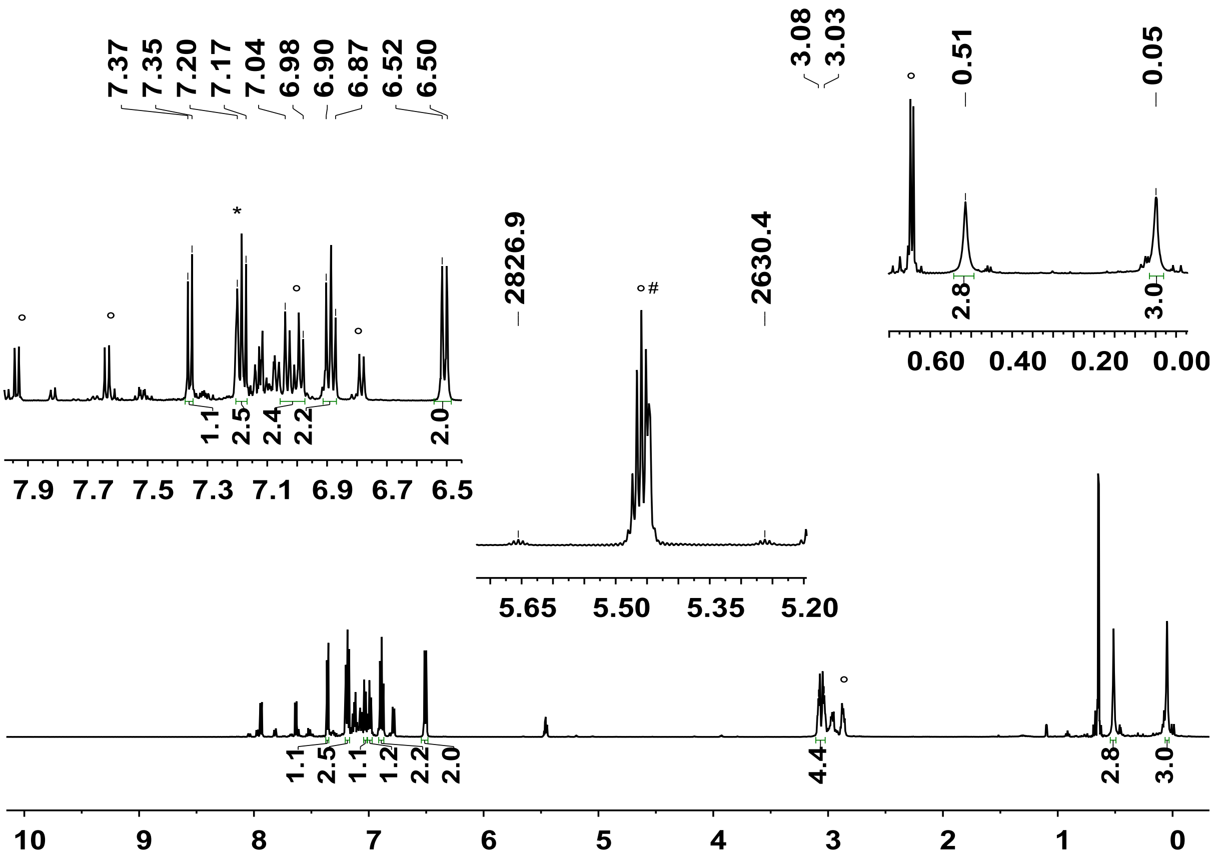

Figure S78 – ^{1}H NMR (499.87 MHz, 305.1 K, *C_6D_6, #Ph_3CH, °5-bromo-6-dimethylsilylacenaphthen 7e) of the reaction mixture containing silyl borate 2c[$B(C_6F_5)_4$].

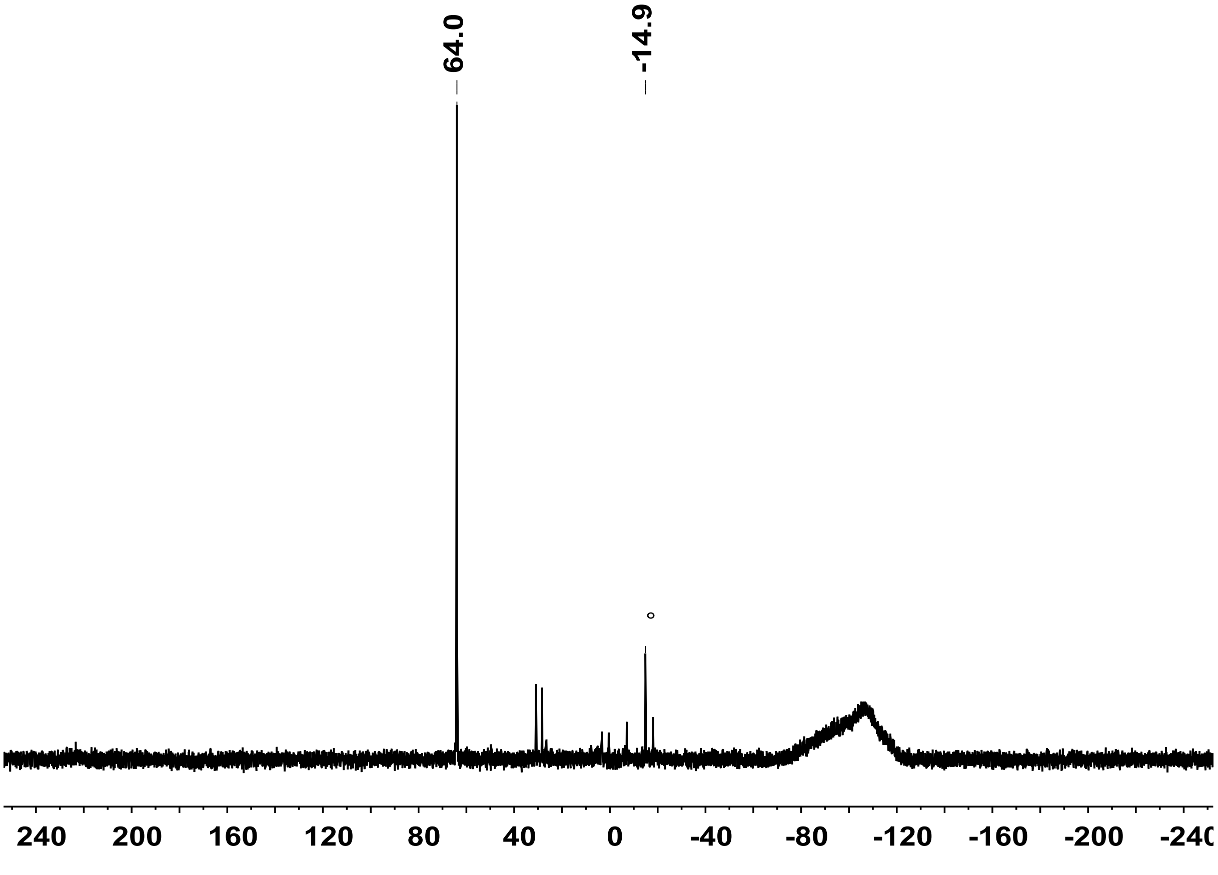

Figure S79 – $^{29}Si\{^{1}H\}$ NMR spectrum (99.31 MHz, 305.0 K, C_6D_6, °5-bromo-6-dimethylsilylacenaphthen 7e) of the reaction mixture containing silyl borate 2c[$B(C_6F_5)_4$].

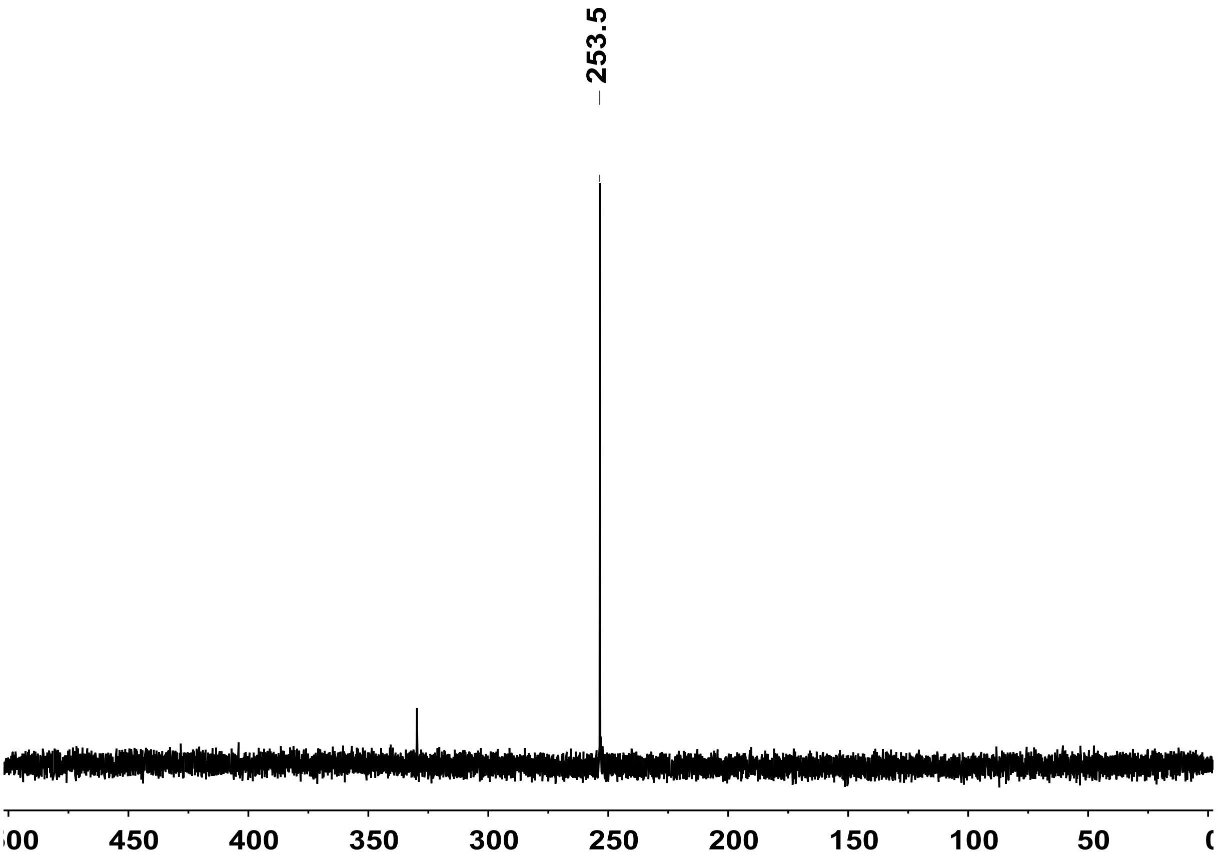

Figure S80 – $^{77}Se\{^{1}H\}$ NMR spectrum (95.36 MHz, 305.0 K, C_6D_6) of the reaction mixture containing silyl borate 2c[B(C_6F_5)$_4$].

1.9. Data from X-ray diffraction analysis of compounds

Single crystal X-ray data were measured on a *Bruker AXS Apex-II CCD* diffractometer with graphite-monochromated Mo-Kα radiation (λ = 71.073 pm, Kappa 4 circle goniometer, *Bruker Apex II* detector). Absorption corrections based on symmetry-related measurements (multi-scan) were performed with the program SADABS.[10] The structures were solved with the program SHELXS and refined with SHELXL.[S11] Pertinent data are summarized in Tables S2 - 3. CCDC-1935621 and CCDC-1935639, contain the supplementary crystallographic data for this paper. These data can be obtained free of charge from The Cambridge Crystallographic Data Centre. The Director, CCDC, 12 Union Road, Cambridge CB2 1EZ, UK [Fax: (internat.) +44-1223/336-033; E-mail: deposit@ccdc.cam.ac.uk].

1.9.1. Data from X-ray diffraction analysis of compound BCF adduct 4

Colorless needles of compound **4** that were suitable for X-ray diffraction analysis were obtained by adding *n*-hexane to a solution of nitrilium borate **10e**[B(C_6F_5)$_4$] in CD_2Cl_2 at room temperature.

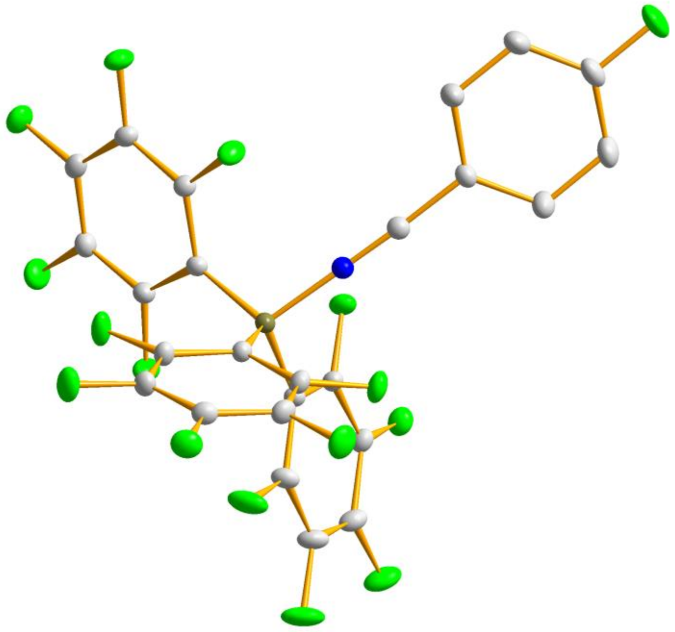

Figure S81 – Molecular structure of complex 4 in the crystal. Pertinent bond lengths and angles: B–N 158.77(11) pm; C–N 114.56(10) pm; C–C^{ipso} 142.47(10) pm; B–N–C 179.732(77)°; ∑α(CBC) = 340.0°. (Thermal ellipsoids drawn at 50%, color code: grey carbon; green fluorine; blue nitrogen, brown boron).

Table S2 – Crystal data and structure refinement for compound 4 (CCDC-1935621).

Identification code	skd14	
Empirical formula	C25 H4 B F16 N	
Formula weight	633.10	
Temperature	100(2) K	
Wavelength	0.71073 Å	
Crystal system	Monoclinic	
Space group	$P2_1/c$	
Unit cell dimensions	a = 12.6503(4) Å	a = 90°.
	b = 12.0109(4) Å	b = 94.2726(13)°.
	c = 14.7028(4) Å	g = 90°.
Volume	2227.76(12) $Å^3$	
Z	4	
Density (calculated)	1.888 Mg/m^3	
Absorption coefficient	0.205 mm^{-1}	
F(000)	1240	
Crystal size	0.460 x 0.180 x 0.160 mm^3	
Theta range for data collection	1.614 to 36.317°	
Index ranges	-21<=h<=21, -20<=k<=20, -24<=l<=24	
Reflections collected	118890	
Independent reflections	10787 (R(int) = 0.0282)	
Observed reflections (I > 2(I))	8916	
Completeness to theta = 36.317°	100.0 %	
Absorption correction	Semi-empirical from equivalents	
Max. and min. transmission	1.0000 and 0.9700	
Refinement method	Full-matrix least-squares on F^2	
Data / restraints / parameters	10787 / 0 / 388	
Goodness-of-fit on F^2	1.097	
Final R indices (I>2sigma(I))	R1 = 0.0347, wR2 = 0.0945	
R indices (all data)	R1 = 0.0446, wR2 = 0.1013	
Extinction coefficient	n/a	
Largest diff. peak and hole	0.609 and -0.226 e.$Å^{-3}$	

1.9.2. Data from X-ray diffraction analysis of compound $[2c]_2[B_{12}Br_{12}]$

The crystals of silyl cation **2c** were obtained from a mixture of silane **7c**, *p*-fluorobenzonitrile and half of an equivalent trityl *closo*-borate $[Ph_3C]_2[B_{12}Br_{12}]$ in dichloro methane (c = 0.12 mol·L^{-1}). The resulting mixture was then slowly covered with hexane and/or toluene. After a while green crystals grew at the phase boundary.

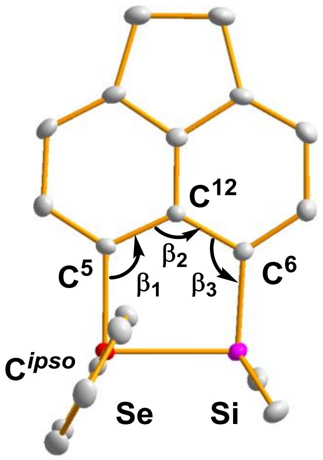

Figure S82 – Molecular structure of cation 2c in the crystal of $[2c]_2[B_{12}Br_{12}]$ x 8 $[H_2CCl_2]$. Pertinent bond lengths and angles: Se–Si 243.05(6) pm; Se–C^5 192.4(2) pm; Si–C^6 185.2(2) pm; β_1 115.37(16)°; β_2 125.7(2)°; β_3 114.85(16)°; C^5–Se–C^{ipso} 101.19(9)°; C^6–C^1–Se–Si -7.941(67)°; $\Sigma\alpha(SiC_3)$ 346.06(3)°; $\Sigma\alpha$(Se) = 288.41(2)°; $\Sigma\beta$ = 355.9(4)°. (Thermal ellipsoids drawn at 50%, hydrogen atoms omitted).

Table S3 – Crystal data and structure refinement for compound $[2c]_2[B_{12}Br_{12}]$ (CCDC-1935639).

Identification code	sara21	
Empirical formula	C48 H54 B12 Br12 Cl16 Se2 Si2	
Formula weight	2500.85	
Temperature	100(2) K	
Wavelength	0.71073 Å	
Crystal system	Orthorhombic	
Space group	Pbca	
Unit cell dimensions	a = 17.2140(9) Å	α = 90°.
	b = 17.6120(9) Å	β = 90°.
	c = 26.9095(14) Å	γ = 90°.
Volume	8158.2(7) $Å^3$	
Z	4	
Density (calculated)	2.036 Mg/m^3	
Absorption coefficient	7.372 mm^{-1}	
F(000)	4760	
Crystal size	0.280 x 0.240 x 0.050 mm^3	
Theta range for data collection	1.513 to 32.031°	
Index ranges	-25<=h<=25, -26<=k<=26, -40<=l<=39	
Reflections collected	206929	
Independent reflections	14214 (R(int) = 0.0714)	
Observed reflections	(I > 2(I)) 10981	
Completeness to theta = 32.031°	100.0 %	
Absorption correction	Numerical	
Max. and min. transmission	0.7293 and 0.1754	
Refinement method	Full-matrix least-squares on F^2	
Data / restraints / parameters	14214 / 0 / 453	
Goodness-of-fit on F^2	0.995	
Final R indices (I>2sigma(I))	R1 = 0.0291, wR2 = 0.0512	
R indices (all data)	R1 = 0.0520, wR2 = 0.0564	
Extinction coefficient	n/a	
Largest diff. peak and hole	1.395 and -1.180 e.$Å^{-3}$	

2. Computational Details

All quantum chemical calculations were carried out using the Gaussian09 package.[S12] The molecular structure optimizations were performed using the M06-2X functional [S13] along with the def2-tzvp basis set for the elements F, I, Br, Te, Se, S, O, Si, C, B, H and using the corresponding pseudopotential for Te and I.[S14] Every stationary point was identified by a subsequent frequency calculation either as minimum (Number of imaginary frequencies (NIMAG): 0). We calculated the fluorine ion affinity (FIA) for cations **2** and **9** versus BEt_3 at the M06-2X/def2-TZVP level of theory with inclusion of solvent effects using the SCIPM model with methylene chloride. The SCF energies (E(SCF)) and the absolute computed Gibbs free energies at T = 298.15 K and p = 0.101 MPa (1 atm) in the gas phase (G298) are given in Tables S4 and S5 for all optimized molecular structures. The optimized molecular structures of compounds **2, 9-13**, FBN, BEt_3, $[FBEt_3]^-$, BCF, $[FBCF]^-$ are given as cartesian coordinates in the structure file (Computed_Molecular_structures.xyz).

Table S4 – Calculated absolute energies (E(SCF)) and free enthalpies at 298 K (G(298)), at M06-2X/Def-2TZVP.

**Compound**	**E(SCF) [a.u.]**	**ZPVE [kJmol^{-1}]**	**G(298) [a.u.]**
Ace -OPh nitrilium **10a**	-1561.81304	1125	-1561.44779
Ace -OPh cation **2a**	-1138.04862	878	-1137.76254
Ace -OPh fluoride **12a**	-1238.20091	885	-1237.91494
Naph-OPh nitrilium **11a**	-1484.39743	1035	-1484.06303
Naph-OPh cation **9a**	-1060.63519	787	-1060.38124
Naph-OPh fluoride **13a**	-1160.78454	793	-1160.53113
Ace-SPh nitrilium **10b**	-1884.77898	1118	-1884.41449
Ace-SPh cation **2b**	-1461.02458	870	-1460.74233
Ace- SPh fluoride **12b**	-1561.16313	878	-1560.87980
Ace-SePh nitrilium **10c**	-3888.16376	1115	-3887.80097
Ace-SePh cation **2c**	-3464.41101	867	-3464.13112
Ace-SePh fluoride **12c**	-3564.54765	874	-3564.26783
Naph-SePh nitrilium **11c**	-3810.74308	1023	-3810.41519
Naph-SePh cation **9c**	-3386.99168	777	-3386.74370
Naph-SePh fluoride **13c**	-3487.12705	783	-3486.87826
Ace-TeMes nitrilium **10d**	-1872.44664	1331	-1872.00878
Ace-TeMes cation **2d**	-1448.69925	1084	-1448.34329
Ace-TeMes fluoride **12d**	-1548.83205	1090	-1548.47630

Ace-Br nitrilium **10e**	-3829.13620	875	-3828.86200
Ace-Br cation **2e**	-3405.36322	626	-3405.16796
Ace-Br fluoride **12e**	-3505.52743	635	-3505.33072
Ace-I nitrilium **10f**	-1552.56799	874	-1552.29293
Ace-I cation **2f**	-1128.80006	625	-1128.60597
Ace-I fluoride **12f**	-1228.95914	634	-1228.76344
Nap-Br nitrilium **11e**	-3751.71364	783	-3751.47150
Nap-Br cation **9e**	-3327.94326	533	-3327.78120
Nap-Br fluoride **13e**	-3428.10633	542	-3427.94224
Ace-H nitrilium **6**	-1255.54149	900	-1255.25328
Ace- H cation **5**	-831.74406	652	-831.53745
Ace- H fluoride	-931.93853	659	-931.72989
4-Fluorbenzonitril	-423.72783	245	-423.66734
BEt3	-262.48406	527	-262.31828
FBEt3	-362.43997	530	-362.27401
BCF adduct **4**	-2632.23189	662	-2632.05191
BCF flouride	-2308.49985	418	-2308.40367
BCF	-2208.47342	416	-2208.37364

Table S5 – Calculated absolute energies (E(SCF)) and free enthalpies at 298 K (G(298)), at M06-2X/Def2-TZVP in DCM, *single point.

**Compound**	**E(SCF) [a.u.]**	**ZPVE [kJmol^{-1}]**	**G(298) [a.u.]**
Ace -OPh nitrilium **10a**	-1561.86583	1125	-1561.50064
Ace -OPh cation **2a**	-1138.10159	877	-1137.81614
Ace -OPh cation* **2a**	-1138.10148*		
Ace -OPh fluoride* **12a**	-1238.20704*		
Naph-OPh nitrilium **11a**	-1484.45004	1034	-1484.11609
Naph-OPh cation* **9a**	-1060.69028*		
Naph-OPh fluoride* **13a**	-1160.79066*		
Ace-SPh nitrilium **10b**	-1884.82958	1119	-1884.46472
Ace-SPh cation **2b**	-1461.07688	870	-1460.79387
Ace-SPh cation* **2b**	-1461.07677*		
Ace- SPh fluoride* **12b**	-1561.16960*		
Ace-SePh nitrilium **10c**	-3888.21448	1116	-3887.85199
Ace-SePh cation **2c**	-3464.46358	867	-3464.18276
Ace-SePh cation* **2c**	-3464.46350*		
Ace- SePh fluoride* **12c**	-3564.55429*		
Naph-SePh nitrilium **11c**	-3810.79472	1024	-3810.46509

Naph-SePh cation **9c**	-3387.04627	777	-3386.79757
Naph-SePh cation* **9c**	-3387.04608		
Naph-SePh fluoride* **13c**	-3487.13339		
Ace-TeMes nitrilium **10d**	-1872.49669	1331	-1872.05979
Ace-TeMes cation **2d**	-1448.74997	1084	-1448.39282
Ace-TeMes cation* **2d**	-1448.74951*		
Ace-TeMes fluoride* **12d**	-1548.83841*		
Ace-Br nitrilium **10e**	-3829.19102	876	-3828.91326
Ace-Br cation **2e**	-3405.42051	626	-3405.22577
Ace-Br cation*	-3405.42042*		
Ace-Br fluoride *****	-3505.53338*		
Ace-I nitrilium **10f**	-1552.62238	874	-1552.34662
Ace-I cation **2f**	-1128.85687	624	-1128.66403
Ace-I cation*	-1128.85683*		
Ace-I fluoride *****	-1228.96507*		
Nap-Br nitrilium **11e**	-3751.76922	783	-3751.52572
Nap-Br cation **9e**	-3328.00313	534	-3327.84101
Nap-Br cation*	-3328.00303*		
Nap-Br fluoride*	-3428.11190*		
Ace-H nitrilium **6**	-1255.60066	901	-1255.31104
Ace-H cation* **5**	-831.80471*		
Ace-H fluoride*	-931.94356*		
4-Fluorbenzonitril	-423.73492	241	-423.67447
BEt_3	-262.48460	526	-262.31938
BEt_3*	-262.48460*		
$FBEt_3$	-362.50853	530	-362.34316
$FBEt_3$*	-362.50824*		
BCF	-2208.47945	413	-2208.38237
BCF*	-2208.47918*		
BCF fluoride	-2308.54999	417	-2308.45354
BCF fluoride*	-2308.54966*		

2.1. Computed Structures XYZ-files

```
43
ace-H-SiMe2 nitrilium 6 opt
 C      -5.63837300   -0.45750100    0.00321100
 C      -4.98555300    0.74425500   -0.01038300
 C      -3.57863900    0.75060300   -0.01046500
```

C	-2.78950400	-0.40745900	0.00277200
C	-4.87018900	-1.64573600	0.01669400
H	-6.71896400	-0.51899100	0.00381500
C	-3.05508300	2.05648000	-0.02564400
C	-1.36908900	-0.21770300	0.00033000
H	-5.38712800	-2.59674600	0.02739100
C	-0.87468100	1.07390600	-0.01533800
C	-1.69747800	2.22426100	-0.02837200
H	0.19704700	1.23766000	-0.01839300
H	-1.24352700	3.20670200	-0.04026600
C	-4.19692000	3.04413000	-0.03660300
H	-4.14800700	3.70211400	0.83172000
H	-4.14809300	3.68262000	-0.91934800
C	-5.48465500	2.17048100	-0.02670100
H	-6.10355000	2.37749300	0.84671600
H	-6.10342400	2.35748400	-0.90470800
C	-0.28012300	-2.68864500	1.58626100
H	-1.25819300	-3.15111000	1.73128700
H	0.46727900	-3.48258500	1.55961500
H	-0.09225700	-2.04717400	2.44785500
C	-0.27285900	-2.72292500	-1.52675500
H	0.47411900	-3.51631100	-1.47941900
H	-1.25040300	-3.18850500	-1.66543800
H	-0.08158500	-2.10057400	-2.40152000
Si	-0.28547500	-1.70603700	0.01876700
N	1.42887700	-0.99379900	0.01309800
C	2.50332400	-0.59295400	0.00970200
C	3.82556600	-0.09576800	0.00457800
C	4.50151000	0.03738700	-1.21589400
C	4.43147200	0.25141100	1.21966900
C	5.79135900	0.52034900	-1.21921800
H	4.01570000	-0.23608100	-2.14258600
C	5.72140000	0.73421600	1.21212500
H	3.89236000	0.14117600	2.15068300
C	6.37611900	0.85908700	-0.00611300
H	6.35333500	0.64038400	-2.13472100
H	6.23089000	1.01500100	2.12314900
F	7.61082000	1.32099600	-0.01118100
C	-3.49590000	-1.63562100	0.01666700
H	-2.96868300	-2.58394700	0.02721700

54

ace-OPh-SiMe2 nitrilium 10a opt

C	-4.38377400	2.14243900	-0.00000100
C	-3.38654100	3.07716400	-0.00012500
C	-2.05395900	2.62661000	-0.00007600
C	-1.68588400	1.28449400	0.00008300
C	-2.74694200	0.35742400	0.00022100
C	-4.05489300	0.75831900	0.00018000
H	-5.42871400	2.42549200	-0.00003000
C	-1.11933800	3.67288800	-0.00020500
C	-0.30463300	0.94103500	0.00010500

H	-4.84726400	0.02144200	0.00029600
C	0.59853800	1.99029300	-0.00002500
C	0.21324700	3.35408000	-0.00017500
H	1.66193700	1.78615300	-0.00001200
H	0.97959600	4.11898900	-0.00026900
C	-1.86940000	4.98501800	-0.00033600
H	-1.60889900	5.58092500	0.87520100
H	-1.60886300	5.58079100	-0.87595300
C	-3.37950100	4.59041600	-0.00033100
H	-3.89321500	4.98719100	0.87571300
H	-3.89314800	4.98693500	-0.87652900
O	-2.29894200	-0.94912800	0.00043600
C	0.00747200	-1.83863400	1.57358900
H	-0.80588400	-2.55929400	1.48274500
H	0.92283600	-2.38957800	1.79013800
H	-0.21046100	-1.18315700	2.41767300
C	-3.22647500	-1.98727800	0.00038000
C	-3.66489600	-2.49520300	-1.20998800
C	-3.66491100	-2.49529500	1.21070200
C	-4.56418500	-3.55259800	-1.20330400
H	-3.30942500	-2.05810000	-2.13469500
C	-4.56419900	-3.55269400	1.20392600
H	-3.30946000	-2.05825400	2.13544600
C	-5.01157100	-4.08111500	0.00028900
H	-4.91705600	-3.96139200	-2.14078500
H	-4.91708100	-3.96155900	2.14137200
H	-5.71148300	-4.90575700	0.00025100
C	0.00718300	-1.83898300	-1.57278900
H	0.92268600	-2.38955100	-1.78969400
H	-0.80580000	-2.56000000	-1.48141200
H	-0.21147400	-1.18376300	-2.41688400
Si	0.14686200	-0.86316700	0.00028100
N	2.04905100	-0.64872300	0.00012000
C	3.19289600	-0.58751000	-0.00001300
C	4.60814300	-0.50780400	-0.00020500
C	5.29170400	-0.47169400	-1.22102600
C	5.29207500	-0.47190400	1.22041500
C	6.66833300	-0.40052900	-1.21998500
H	4.74479300	-0.49977000	-2.15371600
C	6.66870400	-0.40073800	1.21896600
H	4.74544800	-0.50014800	2.15326600
C	7.32945900	-0.36723000	-0.00060700
H	7.23904500	-0.37156000	-2.13757900
H	7.23969300	-0.37193100	2.13639200
F	8.64826600	-0.30016800	-0.00079900

50

naph-OPh-SiMe2 nitrilium 11a opt

C	4.47126100	2.65678200	-0.00849700
C	3.48687900	3.60171300	-0.01618800
C	2.12220200	3.21721500	-0.01767500
C	1.79674000	1.84388900	-0.01068800

C	2.85435600	0.91161600	-0.00325600
C	4.16473400	1.27430700	-0.00196900
H	5.51062800	2.95558700	-0.00748600
C	1.05712100	4.14690100	-0.02629200
C	0.45092400	1.38351600	-0.01159600
H	4.95320600	0.53486000	0.00370100
C	-0.54210700	2.33614100	-0.02061500
C	-0.23961600	3.71469300	-0.02801800
H	-1.58529800	2.05035600	-0.02272700
H	-1.04900300	4.43251900	-0.03520000
O	2.38967400	-0.39787300	0.00195200
C	0.31930600	-1.42867900	-1.58813300
H	0.99783400	-2.27288700	-1.45799000
H	-0.64255500	-1.81709700	-1.91792700
H	0.72159600	-0.79231800	-2.37796500
C	3.31876900	-1.44632200	0.00837100
C	3.75934900	-1.93884600	1.22277200
C	3.76285100	-1.95015900	-1.20010800
C	4.66746800	-2.98887000	1.22164900
H	3.40053100	-1.49962000	2.14458600
C	4.67094700	-3.00009300	-1.18662600
H	3.40656600	-1.51956500	-2.12697400
C	5.11970700	-3.51917000	0.02061600
H	5.02312200	-3.38931100	2.16131700
H	5.02928800	-3.40927000	-2.12149400
H	5.82748600	-4.33693400	0.02545300
C	0.31779300	-1.40505400	1.60402500
H	-0.64509400	-1.78438700	1.94127400
H	0.99267000	-2.25391000	1.48542300
H	0.72425000	-0.75851500	2.38339500
H	3.73513900	4.65511200	-0.02143500
H	1.28523700	5.20558200	-0.03186700
Si	0.19940200	-0.46820400	0.00085800
N	-1.76060000	-0.38314500	-0.00194000
C	-2.90545300	-0.42292600	-0.00051400
C	-4.32443000	-0.46463400	0.00148300
C	-5.01075300	-0.38257400	1.21810700
C	-5.00852000	-0.58724200	-1.21298600
C	-6.38891400	-0.42396200	1.21954900
H	-4.46487100	-0.28748800	2.14682900
C	-6.38664900	-0.62871200	-1.21001000
H	-4.46100500	-0.64865300	-2.14355600
C	-7.04975900	-0.54644200	0.00584600
H	-6.96046300	-0.36380500	2.13496200
H	-6.95647000	-0.72281000	-2.12364800
F	-8.37031500	-0.58623500	0.00797800

54

ace-SPh-SiMe2 nitrilium 10b opt

C	-4.37064000	2.28011300	0.84190100
C	-3.45203600	3.02344700	0.14779200
C	-2.18394000	2.47197600	-0.10741300

C	-1.78400900	1.19816000	0.30952200
C	-2.77182900	0.44798100	0.99366800
C	-4.01451700	0.97697200	1.25725100
H	-5.35968500	2.65774800	1.06647400
C	-1.36540200	3.33311200	-0.85787600
C	-0.46007800	0.76640000	0.00106900
H	-4.74314100	0.37046200	1.78098200
C	0.32073200	1.63041000	-0.74798500
C	-0.11211700	2.90260700	-1.19597900
H	1.32708800	1.34115400	-1.01988900
H	0.55866600	3.51247700	-1.78770700
C	-2.12850700	4.60421500	-1.14552600
H	-1.60137300	5.47516000	-0.75475400
H	-2.23536600	4.75597000	-2.22037400
C	-3.51024500	4.40745900	-0.45470500
H	-3.68152100	5.15767300	0.31819900
H	-4.33337700	4.49195300	-1.16471500
S	-2.35871600	-1.21444700	1.41682600
C	0.60115700	-0.78299100	2.57598400
H	-0.27085800	-0.78715000	3.22608300
H	1.21577100	-1.64974800	2.82892500
H	1.17976400	0.11672100	2.78990800
C	-3.06804600	-2.16390800	0.08425900
C	-3.27550900	-3.51497300	0.33625100
C	-3.35671200	-1.61846900	-1.15953000
C	-3.76478500	-4.32981700	-0.67395900
H	-3.06691700	-3.92436600	1.31750400
C	-3.85564600	-2.44229800	-2.15872900
H	-3.20126500	-0.56396400	-1.34816700
C	-4.05553600	-3.79556500	-1.92131500
H	-3.93174800	-5.38086000	-0.47884300
H	-4.08812400	-2.02119600	-3.12813300
H	-4.44573900	-4.43119400	-2.70476300
C	0.12916000	-2.47545800	-0.12427700
H	1.12556500	-2.79724600	-0.42313800
H	-0.29298200	-3.23974500	0.52994000
H	-0.50037000	-2.42548300	-1.01375500
Si	0.16529600	-0.83304600	0.75870900
N	2.07982900	-0.50194800	0.28958900
C	3.21423900	-0.41042500	0.15919300
C	4.62023800	-0.28060200	-0.00124100
C	5.28404800	-1.07069500	-0.94483800
C	5.31395900	0.63847000	0.79267900
C	6.64858100	-0.93988400	-1.09591300
H	4.73145300	-1.77670900	-1.54981700
C	6.67838000	0.76880100	0.64102400
H	4.78377800	1.23966400	1.51884400
C	7.31898600	-0.02235000	-0.30070300
H	7.20231100	-1.52970700	-1.81284100
H	7.25446500	1.46547300	1.23373700
F	8.62672900	0.10380200	-0.44728800

54
ace-SePh-SiMe2 nitrilium ion 10c opt

Se	-2.32020300	-1.18708800	-1.38060800
C	-0.27349700	0.83841400	0.04477400
C	0.54663800	1.66022000	0.80037700
C	0.17834000	2.94751200	1.25975800
C	-1.05188000	3.43939300	0.92600900
C	-1.91481100	2.62193400	0.17506200
C	-3.15594100	3.24119700	-0.06234600
C	-4.12224100	2.55381100	-0.74619400
C	-3.84168400	1.23429100	-1.16625900
C	-2.62523400	0.63881400	-0.92398500
C	-1.58261400	1.32949100	-0.25373300
C	-2.99505700	-2.05628800	0.20267100
C	-3.62476800	-2.08984800	2.51027800
C	-3.18684300	-1.38102400	1.39912700
H	-4.61257300	0.67110300	-1.67723700
H	-1.17659900	5.58937800	0.83143800
H	-3.78214600	-1.56900000	3.44570600
H	-3.00497500	-0.31623200	1.47093500
C	-3.23784400	-3.42040500	0.10329400
C	-3.66885100	-4.11822300	1.22241800
C	-3.86160100	-3.45485900	2.42621500
H	-4.20334200	-3.99981800	3.29605600
H	1.53831900	1.32220400	1.06868700
H	0.87932100	3.51977800	1.85398100
C	-1.74505500	4.74619400	1.22541600
C	-3.14051200	4.62261200	0.54789100
H	-5.09313300	2.98468200	-0.95379600
H	-1.83272300	4.89992600	2.30167900
H	-3.28341600	5.38468500	-0.21909000
H	-3.95131900	4.74211600	1.26695100
H	-3.10237700	-3.93598900	-0.84003100
H	-3.86376000	-5.17992400	1.14767600
Si	0.32082200	-0.77863900	-0.70851500
C	0.28283800	-2.39698000	0.22264400
H	-0.39057400	-2.32803400	1.07820900
H	-0.09336800	-3.18983900	-0.42570300
H	1.26785300	-2.69075100	0.58207800
C	0.63068600	-0.76889400	-2.55400800
H	-0.05185800	-0.07310000	-3.04448400
H	1.64810000	-0.47046700	-2.80332400
H	0.45081500	-1.76136000	-2.97026600
N	2.24778500	-0.46974900	-0.28688400
C	3.38391600	-0.40244100	-0.15689000
C	4.79215000	-0.30080700	0.00299900
C	5.44044100	-1.10237300	0.94780200
C	5.50338000	0.60471700	-0.79115200
C	6.80702800	-0.99669800	1.09984800
H	4.87429100	-1.79724600	1.55318600
C	6.86988500	0.70972600	-0.63865500

H	4.98491100	1.21571600	-1.51758900
C	7.49486700	-0.09225500	0.30435200
H	7.34922200	-1.59574100	1.81795200
H	7.45933200	1.39496300	-1.23155200
F	8.80449800	0.00997400	0.45226000

50
naph-SePh-SiMe2 nitrilium ion 11c opt

Se	-2.48860400	-0.56238100	-1.38466400
C	-0.37383900	1.05487900	0.30839900
C	0.49059900	1.59259100	1.23600800
C	0.23252300	2.81236600	1.89465400
C	-0.88858500	3.52512800	1.59200000
C	-1.83362100	3.01548500	0.67047700
C	-3.01951600	3.73161600	0.38038200
C	-3.96883200	3.21863000	-0.45591400
C	-3.78722700	1.93858200	-1.01441100
C	-2.64307800	1.23400600	-0.75388300
C	-1.59897700	1.75350900	0.05933000
C	-3.26332700	-1.51422500	0.10366400
C	-3.90251500	-1.72297900	2.39871800
C	-3.38066600	-0.95346900	1.36721400
H	-4.56256800	1.50482100	-1.63243000
H	-4.00392900	-1.29027400	3.38544100
H	-3.07969600	0.06923200	1.55399000
C	-3.66042200	-2.82232200	-0.14107800
C	-4.17442800	-3.58212500	0.90016000
C	-4.29494300	-3.03416500	2.16983200
H	-4.70219500	-3.62639500	2.97832600
H	1.41309900	1.08478600	1.48027600
H	0.94169900	3.18665000	2.62090600
H	-4.87192300	3.77252300	-0.67293100
H	-3.58019100	-3.24623000	-1.13493900
H	-4.48925300	-4.60036500	0.71330200
Si	0.09968800	-0.46041200	-0.71343600
C	-0.00730400	-2.19031900	-0.01942400
H	-0.67975200	-2.20780400	0.83980300
H	-0.41527500	-2.87146400	-0.76819100
H	0.96244600	-2.57183500	0.29557000
C	0.41822300	-0.17639300	-2.53667700
H	-0.19439500	0.65121000	-2.89920500
H	1.45941700	0.05935800	-2.75038800
H	0.14283400	-1.06597400	-3.10539900
N	2.06620800	-0.31999000	-0.28328200
C	3.20693700	-0.32591200	-0.17754900
C	4.62131900	-0.32303200	-0.03736800
C	5.25247200	-1.41082200	0.57454000
C	5.35514700	0.76848900	-0.51262700
C	6.62463500	-1.40643700	0.71279700
H	4.66915300	-2.24742400	0.93471800
C	6.72745800	0.77239000	-0.37385200
H	4.85028900	1.60059000	-0.98444900

C	7.33502300	-0.31481500	0.23641100
H	7.15324300	-2.22524800	1.18045900
H	7.33416800	1.59526800	-0.72508900
F	8.65015100	-0.30982700	0.37107300
H	-3.16410600	4.69887000	0.84624500
H	-1.08331500	4.48087700	2.06316100

63

ace-TeMes-SiMe2 nitrilium ion 10d opt

C	-3.05872700	2.22880900	-0.89853000
C	-1.92272400	1.45917800	-0.78499900
C	-0.73133600	1.98389800	-0.21123900
C	-0.83708500	3.30849300	0.24517800
C	-1.99884200	4.09797200	0.13909800
C	-3.11690300	3.56261400	-0.43755100
H	-3.94758700	1.79782300	-1.34268100
C	0.52128900	1.31362900	-0.00494700
C	0.19730500	3.99854800	0.90494200
H	-4.03576800	4.12529800	-0.54077900
C	1.37503200	3.34566000	1.12734500
C	1.51076200	2.01734800	0.66394000
H	2.20143000	3.81666600	1.64424300
H	2.46199800	1.54393300	0.86057700
C	-0.27459500	5.38627500	1.26324000
H	0.35934400	6.14428800	0.80176900
H	-0.22345300	5.54552500	2.34099500
C	-1.73571500	5.45753200	0.73972300
H	-2.44338000	5.67403200	1.54052800
H	-1.85756400	6.24019900	-0.00995300
Te	-1.94993800	-0.53655400	-1.43194000
C	-3.10562500	-1.49778900	0.07617200
C	-3.01546100	-1.18135800	1.43648800
C	-3.97095300	-2.50692000	-0.37773500
C	-3.82478000	-1.88294100	2.32746100
C	-4.74891000	-3.17767100	0.55677800
C	-4.69810700	-2.87695100	1.91285300
H	-3.76162400	-1.64112900	3.38259900
H	-5.41550200	-3.96078700	0.21294900
Si	0.86280100	-0.42482300	-0.64899400
C	1.19784000	-0.60134900	-2.48458400
H	0.58736800	0.09933300	-3.05554500
H	2.24181700	-0.40482000	-2.72687500
H	0.94746000	-1.60932700	-2.81951200
C	0.70474000	-1.92875900	0.45453100
H	-0.32513900	-2.23903400	0.62098800
H	1.24655500	-2.76155700	-0.00039200
H	1.16028800	-1.72089700	1.42487300
C	-2.10666500	-0.12053600	1.99016800
H	-1.11103800	-0.16567500	1.54939700
H	-2.49890200	0.88036500	1.79735600
H	-2.00399200	-0.23939300	3.06737600
C	-4.08619300	-2.89843500	-1.82807300

H	-4.39588000	-2.05430400	-2.44674600
H	-3.13716900	-3.26538500	-2.22639600
H	-4.82009000	-3.69240000	-1.94800700
C	-5.57861100	-3.59932400	2.89141400
H	-6.59551000	-3.20355400	2.85086700
H	-5.63195400	-4.66307200	2.66019600
H	-5.21526000	-3.48417900	3.91126000
N	2.84139700	-0.42071900	-0.24557600
C	3.96164300	-0.61179400	-0.10116400
C	5.35276800	-0.83781700	0.08453400
C	6.27211300	-0.16155300	-0.72338200
C	5.77571600	-1.73287900	1.07224900
C	7.62172500	-0.38147800	-0.54235800
H	5.92623900	0.52745300	-1.48203000
C	7.12529600	-1.95293100	1.25241700
H	5.05020200	-2.24750700	1.68756300
C	8.02170200	-1.27275400	0.44184600
H	8.36728600	0.11966700	-1.14355100
H	7.49703200	-2.63679500	2.00252500
F	9.31559700	-1.48352300	0.61526400

43

ace 5-Br 6-SiMe2 nitrilium ion 10e opt

C	4.67846500	-0.48655400	0.02650200
C	3.32398300	-0.69954100	0.00999700
C	2.37983900	0.35781800	-0.00253200
C	2.97418500	1.63449200	-0.00039600
C	4.36130100	1.87776100	0.01649200
C	5.22039500	0.81590400	0.03103400
H	5.34531300	-1.33803500	0.03511000
C	0.94370500	0.30097300	-0.01785800
C	2.24323200	2.83831200	-0.01707800
H	6.29472800	0.94397400	0.04435000
C	0.88073600	2.77795800	-0.03581700
C	0.26272600	1.50810100	-0.03464100
H	0.26798400	3.66992500	-0.05043300
H	-0.81836900	1.51090300	-0.04782900
C	3.19698700	4.00636400	-0.01207700
H	3.04974800	4.62811800	-0.89578400
H	3.02306800	4.64392300	0.85531600
C	4.61172700	3.36624400	0.01451900
H	5.17347300	3.66677300	0.89931200
H	5.20415100	3.66034500	-0.85215900
Si	-0.04349300	-1.28445500	-0.00750400
Br	2.72790500	-2.50585900	-0.00252100
C	-0.15840900	-2.21385100	1.60246800
H	-1.07929900	-1.98102500	2.13693900
H	-0.12591400	-3.28966900	1.42684300
H	0.68206600	-1.95391400	2.24460600
C	-0.16762500	-2.23528000	-1.60403800
H	-0.14423900	-3.30879300	-1.41317000
H	-1.08712300	-2.00225700	-2.14082700

H	0.67423000	-1.99144900	-2.25056400
N	-1.81345000	-0.59455800	-0.00630000
C	-2.92833900	-0.32813800	-0.00260800
C	-4.30310800	0.00911400	0.00201500
C	-4.97469000	0.16362100	-1.21728900
C	-4.96324900	0.17637200	1.22586800
C	-6.31407800	0.48713700	-1.21124600
H	-4.44745900	0.03030800	-2.15208500
C	-6.30266500	0.49982000	1.22897800
H	-4.42727400	0.05282200	2.15703400
C	-6.95187500	0.64854600	0.01105900
H	-6.87405100	0.61610200	-2.12673900
H	-6.85407300	0.63828200	2.14827100
F	-8.23445100	0.95799700	0.01545200

43

ace 5-I 6-SiMe2 nitrilium ion 10f opt

C	4.34610800	0.33629500	0.64054000
C	3.10961400	-0.13450100	0.26747700
C	2.03822200	0.73898800	-0.06352600
C	2.40378700	2.10094500	-0.06356200
C	3.66669100	2.59703700	0.31433700
C	4.63763900	1.71507400	0.69323900
H	5.13111000	-0.36896900	0.87765000
C	0.67754500	0.43807500	-0.42031900
C	1.55594500	3.14336300	-0.48915000
H	5.62528700	2.03869600	0.99452300
C	0.30143300	2.83463500	-0.92661000
C	-0.11522500	1.48804500	-0.85915600
H	-0.37985900	3.58968200	-1.29665300
H	-1.13318700	1.29116800	-1.16988600
C	2.28648100	4.45899000	-0.39519200
H	2.37530900	4.91446100	-1.38238200
H	1.74299100	5.16645200	0.23141800
C	3.67632200	4.10115000	0.19597800
H	3.82501700	4.56128800	1.17365600
H	4.49127000	4.44229200	-0.44218700
Si	-0.17734600	-1.19972300	-0.15254300
C	-0.11340800	-1.90449600	1.57034300
H	-1.06073100	-1.75954800	2.09017900
H	0.10343000	-2.97201000	1.54040600
H	0.67233800	-1.42047300	2.14989000
C	-0.39822700	-2.35436800	-1.58845900
H	-1.07039100	-3.17142800	-1.31938300
H	-0.83141100	-1.81087800	-2.43032300
H	0.54855300	-2.77734600	-1.91500700
N	-1.99047100	-0.63423100	-0.10766100
C	-3.11271100	-0.41485000	-0.03073200
C	-4.49661200	-0.13206200	0.06546500
C	-5.36350800	-0.59692300	-0.93129800
C	-4.96854700	0.60581400	1.15825500
C	-6.71014800	-0.32162900	-0.83342500

H	-4.97987200	-1.16596300	-1.76711900
C	-6.31567600	0.87978900	1.25349000
H	-4.28268100	0.95581700	1.91763200
C	-7.15993800	0.41093900	0.25648000
H	-7.41722000	-0.65962300	-1.57782800
H	-6.72601400	1.44511000	2.07831900
F	-8.44974100	0.67378000	0.34892100
I	2.94632400	-2.20884700	0.02096200

39
naph 1-Br 8-SiMe2 nitrilium ion 11e opt

C	4.86961900	0.29245500	0.48011400
C	3.57872200	-0.03076700	0.17772000
C	2.56737400	0.93133800	-0.07788300
C	3.00736500	2.28743100	-0.08259200
C	4.34555900	2.61549100	0.23748700
C	5.25553200	1.64428800	0.53157100
H	5.59640400	-0.48750600	0.65900300
C	1.18591800	0.63827500	-0.34199500
C	2.10074500	3.31444900	-0.43715500
H	6.27814700	1.89544500	0.77695300
C	0.81597100	3.02031500	-0.77356900
C	0.36647900	1.68730500	-0.70191900
H	0.12912200	3.80085600	-1.07123500
H	-0.67405200	1.51225200	-0.93650400
Si	0.39289500	-1.04006700	-0.06673000
Br	3.18132600	-1.88252900	-0.00790900
C	0.41647000	-1.67574700	1.68532300
H	-0.52617500	-1.48435900	2.19717600
H	0.60371800	-2.74980600	1.69871200
H	1.21405800	-1.19181200	2.24881200
C	0.27601200	-2.25096400	-1.47742000
H	0.51754800	-3.25692300	-1.13216800
H	-0.72191900	-2.27262400	-1.91412600
H	0.98643500	-1.98512500	-2.25936300
N	-1.44373200	-0.51739300	-0.07524200
C	-2.57493600	-0.33973700	-0.02167100
C	-3.97030900	-0.10882400	0.04370100
C	-4.77947100	-0.48353600	-1.03620400
C	-4.51159700	0.48847700	1.18905200
C	-6.13734500	-0.25924700	-0.96918700
H	-4.34303400	-0.94377200	-1.91216800
C	-5.86982200	0.71172900	1.25345000
H	-3.87008500	0.77095000	2.01261200
C	-6.65601000	0.33365600	0.17379200
H	-6.80130800	-0.53158200	-1.77745500
H	-6.33256600	1.16942400	2.11642800
F	-7.95636200	0.54800700	0.23667000
H	2.45886800	4.33638800	-0.44956900
H	4.63360600	3.65899200	0.23489900

30
ace-H-SiMe2 cation 5 opt

C	2.48188200	2.01801800	-0.04713600
C	2.54661800	0.65121400	0.01715200
C	1.34574000	-0.08076700	-0.00695400
C	0.06947500	0.49837400	-0.08779100
C	1.21334200	2.62492200	-0.14632100
H	3.37231500	2.63265700	-0.03419200
C	1.58207300	-1.46578500	0.03945600
C	-1.03851100	-0.42682100	-0.09928300
H	1.16040900	3.70403500	-0.21408300
C	-0.76964000	-1.80802500	-0.06967800
C	0.51810400	-2.34235500	0.00175900
H	-1.59720100	-2.50756000	-0.08779600
H	0.65942500	-3.41429400	0.02782600
C	3.06250500	-1.71418200	0.11319900
H	3.30956600	-2.27780400	1.01449700
H	3.38797200	-2.32283500	-0.73216800
C	3.71122200	-0.30372000	0.10478500
H	4.29570800	-0.12445300	1.00754400
H	4.38692400	-0.17578800	-0.74107200
C	-4.06977100	-1.25868800	-0.14699700
H	-4.44097800	-1.51619200	0.85006100
H	-4.91168100	-0.83324300	-0.69759400
H	-3.74290400	-2.16927300	-0.64328500
Si	-2.75970600	0.01665500	0.01138600
C	-3.34704700	1.72097300	0.35389400
H	-4.38436200	1.70468000	0.68833400
H	-2.72670100	2.20781300	1.10769900
H	-3.29612600	2.32356500	-0.55826900
C	0.03740900	1.90334900	-0.17076600
H	-0.89689700	2.43649400	-0.27881600

41

ace-OPh-SiMe2 cation 2a opt

C	-1.46111800	-2.64460600	0.00004800
C	-2.60012500	-1.88190800	0.00007300
C	-2.46426900	-0.48353500	0.00004400
C	-1.24192600	0.15859000	0.00000000
C	-0.11408200	-0.66345800	-0.00001100
C	-0.17224900	-2.02062700	-0.00000500
H	-1.50061200	-3.72575000	0.00007000
C	-3.67104700	0.22226700	-0.00007800
C	-1.15548700	1.56911000	-0.00005000
H	0.72570400	-2.62460700	-0.00002400
C	-2.35470000	2.25929500	-0.00007200
C	-3.61199000	1.59772800	-0.00009600
H	-2.35990300	3.34258400	-0.00009800
H	-4.51250500	2.19845400	-0.00019100
C	-4.79312800	-0.79344700	-0.00036300
H	-5.43183000	-0.66897300	0.87474100
H	-5.43076600	-0.66931000	-0.87630900
C	-4.08403000	-2.18969900	0.00018900
H	-4.36194500	-2.77577300	0.87636500

H	-4.36203300	-2.77654900	-0.87543700
O	1.05588600	0.16116900	-0.00010000
C	1.41235900	2.59995800	1.55186300
H	2.49617200	2.46975300	1.53987300
H	1.20964800	3.66963900	1.64637200
H	0.99852100	2.10319700	2.42970600
C	2.35779300	-0.40828400	-0.00007500
C	2.95328400	-0.66800600	-1.21714800
C	2.95309600	-0.66833400	1.21701700
C	4.22925500	-1.21473500	-1.20653800
H	2.43091300	-0.45291900	-2.14050100
C	4.22905400	-1.21508100	1.20647400
H	2.43059800	-0.45343200	2.14034400
C	4.86212300	-1.48496200	-0.00002300
H	4.72630900	-1.42911000	-2.14279400
H	4.72595800	-1.42973000	2.14274400
H	5.85673400	-1.91017400	-0.00000300
C	1.41294800	2.60008900	-1.55138100
H	1.21090500	3.66990000	-1.64572000
H	2.49668200	2.46922100	-1.53910200
H	0.99907600	2.10371900	-2.42943200
Si	0.64933700	1.96988400	0.00007100

37

naph-OPh-SiMe2 cation 9a opt

C	-1.69308200	0.35497200	-0.01934000
C	-2.80070600	1.22153800	-0.00188600
C	-1.81499000	-1.05652800	0.03855200
C	-0.41006800	0.91608800	-0.09899600
C	-4.07866300	0.62199600	0.08126200
C	-2.55680100	2.61864200	-0.06652000
C	-4.20763800	-0.74191200	0.13931400
C	-1.28160400	3.10406500	-0.14408500
C	-3.07838600	-1.59026000	0.11736900
C	-0.15214500	2.24141600	-0.16293800
H	-4.95760900	1.25449100	0.09836700
H	-3.39772500	3.30007600	-0.05391600
H	-5.19327300	-1.18323100	0.20422300
H	-1.11125800	4.17065400	-0.19350700
H	-3.23197100	-2.66143400	0.16433500
H	0.85379900	2.63318300	-0.22580600
C	0.41075400	-2.63199400	-1.56726400
H	0.09935300	-2.06912400	-2.44768000
H	1.49381800	-2.76684900	-1.58776500
C	0.56223900	-2.51965100	1.53282600
H	0.14876800	-3.52087300	1.67846800
H	0.29895600	-1.92027400	2.40470800
H	-0.04520000	-3.62347300	-1.62572400
H	1.64811500	-2.61637400	1.47548400
Si	-0.12486600	-1.80137500	-0.01579100
C	1.96888400	0.19879600	-0.03090200
C	2.70147800	0.20369000	-1.19886500

C	2.50133000	0.46414200	1.21383600
C	4.05928700	0.48198500	-1.10473800
H	2.22303400	0.00117700	-2.14837100
C	3.85884400	0.74261800	1.28606900
H	1.87169300	0.45763400	2.09448600
C	4.63225500	0.74880100	0.13129100
H	4.66525700	0.49194800	-2.00042000
H	4.31082200	0.95277400	2.24588100
H	5.69001100	0.96542200	0.19601200
O	0.58195100	-0.11461300	-0.11595100

41

ace-SPh-SiMe2 cation 2b opt

C	-2.08807500	-2.19992000	-1.41619300
C	-2.90039600	-1.51355800	-0.54711900
C	-2.42455200	-0.32354100	0.02710600
C	-1.16559500	0.21115200	-0.23224000
C	-0.35119600	-0.53024900	-1.11881900
C	-0.79640300	-1.69414200	-1.70074900
H	-2.40660200	-3.11953900	-1.88909800
C	-3.34145300	0.27601500	0.90458900
C	-0.79423300	1.42424900	0.39891600
H	-0.15190100	-2.24074100	-2.37828600
C	-1.70504200	2.00636000	1.26179800
C	-2.97837700	1.44196800	1.52934600
H	-1.45601900	2.93630600	1.76079400
H	-3.64314200	1.94634100	2.21875300
C	-4.58492300	-0.58134500	0.94950700
H	-4.77208900	-0.93796500	1.96304600
H	-5.46418400	-0.01142800	0.64815100
C	-4.29812300	-1.76221500	-0.02906800
H	-4.36308300	-2.72826000	0.47255800
H	-5.01442700	-1.78548700	-0.85117300
C	2.21621100	2.26778500	1.02199200
H	3.17403700	2.40842900	0.51886200
H	2.02078600	3.16340100	1.61835300
H	2.28894300	1.41545000	1.69690500
C	2.32631700	-0.75879600	-0.24264100
C	3.68496900	-0.74929900	-0.52546200
C	1.81582400	-1.37948000	0.88792700
C	4.55538400	-1.37205700	0.35842000
H	4.05909300	-0.27470500	-1.42434900
C	2.69860200	-2.00541600	1.75536200
H	0.75184000	-1.38120800	1.08764400
C	4.06337600	-1.99783700	1.49481800
H	5.61654700	-1.37614300	0.14874400
H	2.31672300	-2.49922400	2.63892100
H	4.74466600	-2.48775800	2.17759000
C	0.73670100	3.39005400	-1.48279100
H	0.40601600	4.32104300	-1.01524600
H	1.70579600	3.57282500	-1.94867000
H	0.01274000	3.13124000	-2.25610300

Si	0.84043200	2.06979500	-0.19367700
S	1.27463300	0.12949400	-1.39025800

41

ace-SePh-SiMe2 cation 2c opt

Se	-1.25152400	0.15035900	-1.36743800
C	0.92970900	1.38311600	0.51683300
C	1.83688600	1.94741500	1.39580800
C	3.11676800	1.39194300	1.64426300
C	3.48870800	0.25007000	0.98321900
C	2.57676200	-0.33334300	0.08826800
C	3.07341900	-1.49832600	-0.52099400
C	2.27521100	-2.17425800	-1.40888100
C	0.97622500	-1.67967300	-1.68066100
C	0.51324100	-0.53934400	-1.06954100
C	1.30837300	0.19345500	-0.15699900
C	-2.28359300	-0.82475300	-0.05096200
C	-2.48264800	-2.10692700	1.94794100
C	-1.67617500	-1.46906500	1.01526600
H	0.34462900	-2.21717600	-2.37707600
H	4.92689700	-0.98275900	2.00609800
H	-2.02525900	-2.61879500	2.78424300
H	-0.59864900	-1.48199900	1.12084700
C	-3.66104300	-0.80226100	-0.21108400
C	-4.45255500	-1.44015000	0.73447300
C	-3.86465900	-2.08945500	1.81087600
H	-4.48481500	-2.58993000	2.54242400
H	1.57689900	2.85810200	1.92469600
H	3.77803300	1.88167400	2.34740500
C	4.74291400	-0.59168200	1.00467800
C	4.47713000	-1.73888700	-0.01692100
H	2.60916600	-3.07475500	-1.90730800
H	5.61688900	-0.00027800	0.73088200
H	4.56604700	-2.72257900	0.44479000
H	5.18934600	-1.71252100	-0.84256900
H	-4.11715500	-0.30746500	-1.05984300
H	-5.52829300	-1.43526200	0.62059200
Si	-0.71330700	2.09062400	0.02292500
C	-0.62844600	3.52839400	-1.13839900
H	0.08055600	3.34883200	-1.94670600
H	-1.60566900	3.75908400	-1.56424200
H	-0.28782700	4.40784700	-0.58521400
C	-2.03325700	2.21488800	1.31067500
H	-2.08969400	1.31635200	1.92449700
H	-1.80143900	3.06280600	1.96131500
H	-3.00935700	2.40094600	0.85995700

37

naph-SePh-SiMe2 cation 9c opt

C	1.71099100	-0.29463800	0.01512400
C	2.84044900	-1.06208700	0.39537700
C	1.50555000	1.00007100	0.56733100
C	0.81250200	-0.87556900	-0.91741700

C	3.73794000	-0.51819700	1.34580300
C	3.03441300	-2.34266400	-0.17742700
C	3.52158300	0.71733600	1.88723700
C	2.15056100	-2.85367100	-1.08625600
C	2.40294900	1.48104000	1.49150700
C	1.01312800	-2.11072100	-1.46598700
H	4.60157100	-1.10268400	1.63863100
H	3.90475000	-2.91590400	0.11790800
H	4.21172600	1.12139100	2.61555600
H	2.31113800	-3.83129200	-1.51972600
H	2.26995500	2.46540000	1.92753600
H	0.31103400	-2.52438400	-2.17842300
C	0.59964100	3.27845700	-1.35729600
H	1.27986000	2.86239200	-2.10083300
H	-0.26657800	3.70492700	-1.86453300
C	-1.20925700	2.55046800	1.07740200
H	-0.79477100	3.33692000	1.71438000
H	-1.54182200	1.73338300	1.71682500
H	1.12224300	4.09115200	-0.84575000
H	-2.06953200	2.96994800	0.55384600
Si	0.10045600	2.01054700	-0.10750400
C	-2.02981500	-0.52146700	-0.07070200
C	-3.36767100	-0.27889500	-0.34473200
C	-1.61760600	-1.15711100	1.09011300
C	-4.32041000	-0.68050100	0.58180000
H	-3.67128500	0.20450000	-1.26522500
C	-2.58454000	-1.55649900	2.00242900
H	-0.56931700	-1.34619800	1.28453600
C	-3.92942600	-1.31610200	1.75168200
H	-5.36832100	-0.50281100	0.38048800
H	-2.28237900	-2.05958200	2.91140200
H	-4.67541100	-1.63335000	2.46814900
Se	-0.75897500	0.13303100	-1.37546000

50

ace-TeMes-SiMe2 cation 2d opt

C	-1.30782600	-1.50131800	-1.83700400
C	-1.04655400	-0.41494000	-1.03625000
C	-1.98914100	0.04184200	-0.07816500
C	-3.17059400	-0.70080300	-0.00130600
C	-3.44886500	-1.82356700	-0.80236400
C	-2.51853400	-2.22800400	-1.72338000
H	-0.57816000	-1.82523900	-2.56907600
C	-1.84131000	1.14323600	0.80960400
C	-4.21007900	-0.40936700	0.90009100
H	-2.68420900	-3.08194200	-2.36696100
C	-4.06234500	0.65233000	1.75243100
C	-2.87142900	1.41598900	1.69214700
H	-4.82933100	0.91963200	2.46761600
H	-2.78050100	2.25240500	2.37785300
C	-5.31932200	-1.41527600	0.70149100
H	-6.24912300	-0.91723100	0.42522600

H	-5.51643900	-1.96325600	1.62325500
C	-4.81246300	-2.35629700	-0.43120400
H	-4.74690500	-3.39198800	-0.09666300
H	-5.47975400	-2.34492000	-1.29347900
Te	0.72134300	0.71303400	-1.19953200
C	2.22271100	-0.36585800	-0.14189100
C	1.95940700	-1.25071300	0.91069100
C	3.52831400	-0.10367200	-0.58386600
C	3.05052400	-1.87937000	1.50448100
C	4.57375500	-0.75935300	0.05398700
C	4.35781900	-1.65505100	1.09397700
H	2.86508700	-2.56770000	2.32099200
H	5.58786800	-0.56044500	-0.27261300
Si	-0.30087200	2.18337300	0.70434300
C	-0.57716900	3.87867900	0.00519300
H	-1.16111100	3.84403100	-0.91432200
H	-1.13944300	4.46824200	0.73458700
H	0.36373500	4.39477300	-0.18741900
C	0.85545700	2.15484600	2.15313000
H	1.18549600	1.14794800	2.40645300
H	1.73374500	2.77104600	1.95579700
H	0.34037200	2.57464000	3.02151400
C	0.58545500	-1.57233000	1.42921100
H	-0.00513100	-0.67739800	1.62664200
H	0.02272400	-2.18078400	0.71941200
H	0.66235500	-2.12940400	2.36047700
C	3.84939800	0.85719900	-1.70008600
H	3.39229800	0.55018500	-2.64288300
H	3.50881900	1.87178400	-1.47839900
H	4.92441500	0.90615900	-1.85585400
C	5.50555800	-2.37503100	1.73900300
H	5.75194800	-3.27419000	1.17038400
H	6.39654200	-1.74938400	1.76814500
H	5.26046500	-2.68098300	2.75462100

30

ace 5-Br 6-SiMe2 cation 2e opt

C	-0.83482000	-2.40345300	-0.00009300
C	-0.03594300	-1.29818900	-0.00002600
C	-0.51174400	0.02255600	-0.00003900
C	-1.90325500	0.12502300	-0.00013300
C	-2.77613400	-0.97758300	-0.00020300
C	-2.24583300	-2.24098400	-0.00018300
H	-0.41036000	-3.39833700	-0.00008000
C	0.25872900	1.21055900	0.00003000
C	-2.57282400	1.35944900	-0.00016700
H	-2.86865600	-3.12543100	-0.00023300
C	-1.82778800	2.51120200	-0.00010200
C	-0.41502100	2.42013300	-0.00000300
H	-2.29290500	3.48818900	-0.00012500
H	0.14464700	3.34928200	0.00004900
C	-4.05969600	1.09406700	-0.00027600

H	-4.53208000	1.54040200	0.87523300
H	-4.53194900	1.54040300	-0.87585500
C	-4.19564000	-0.45901400	-0.00028800
H	-4.73862100	-0.81490300	-0.87604700
H	-4.73872300	-0.81490500	0.87540700
Si	2.09618600	1.02648700	0.00017500
Br	1.89481100	-1.43475400	0.00009100
C	2.99575200	1.40449700	-1.56071400
H	3.03314600	2.49219700	-1.67604900
H	4.02266100	1.03885500	-1.53472300
H	2.48274400	0.99158900	-2.42842000
C	2.99547900	1.40439500	1.56124600
H	4.02242100	1.03883900	1.53537600
H	3.03275900	2.49208500	1.67671700
H	2.48236400	0.99134300	2.42882100

30

ace 5-I 6-SiMe2 cation 2f opt

C	-0.88728500	-2.36167300	-0.00013300
C	-0.17978300	-1.19270200	-0.00004600
C	-0.77520800	0.08425500	-0.00005300
C	-2.17374700	0.05794300	-0.00015600
C	-2.94490100	-1.11873700	-0.00024700
C	-2.30568300	-2.32948900	-0.00023700
H	-0.38231300	-3.31835900	-0.00012500
C	-0.13130300	1.34736700	0.00003000
C	-2.96032700	1.22299900	-0.00018000
H	-2.84734900	-3.26593800	-0.00030500
C	-2.33437700	2.44214300	-0.00009900
C	-0.92049900	2.48543300	0.00000600
H	-2.89291300	3.36886200	-0.00011300
H	-0.45246700	3.46477000	0.00006800
C	-4.41575000	0.82104500	-0.00030000
H	-4.92719100	1.22195700	0.87522400
H	-4.92705900	1.22200700	-0.87587800
C	-4.40615800	-0.73632600	-0.00034400
H	-4.91293900	-1.14218600	-0.87610800
H	-4.91306700	-1.14223600	0.87532300
Si	1.71542100	1.45670300	0.00016400
C	2.52144100	2.02191800	-1.55904000
H	2.34775300	3.09822100	-1.65687600
H	3.59958200	1.86093800	-1.53998400
H	2.09432100	1.53091200	-2.43238500
C	2.52122300	2.02184100	1.55950900
H	3.59936600	1.86086500	1.54059500
H	2.34751800	3.09813800	1.65737400
H	2.09398200	1.53079000	2.43277000
I	1.93417000	-1.17695600	0.00011100

26

naph 1-Br 8-SiMe2 cation 9e opt

C	-1.54202600	-2.21065700	-0.00012600
C	-0.66069500	-1.17926600	-0.00005900

C	-1.00592400	0.18619100	-0.00007300
C	-2.39426100	0.47568400	-0.00016700
C	-3.32966500	-0.58811000	-0.00023700
C	-2.91953600	-1.89033400	-0.00021700
H	-1.21691100	-3.24166600	-0.00011300
C	-0.05000500	1.23496100	0.00000100
C	-2.79277800	1.83377800	-0.00018900
H	-3.64038300	-2.69602700	-0.00027100
C	-1.86783100	2.83961200	-0.00012100
C	-0.49014700	2.53851600	-0.00002500
H	-2.18603400	3.87306800	-0.00014100
H	0.21639500	3.36165200	0.00003000
Si	1.75312900	0.82562200	0.00014700
Br	1.24069000	-1.55888400	0.00005500
C	2.68099800	1.11351800	-1.56273000
H	2.83520600	2.19145300	-1.67233400
H	3.66288600	0.63969500	-1.54264700
H	2.12384400	0.76308900	-2.43074000
C	2.68072000	1.11345400	1.56320100
H	3.66261300	0.63963500	1.54327400
H	2.83490300	2.19138500	1.67288000
H	2.12341200	0.76298500	2.43109700
H	-3.85141000	2.06209400	-0.00026200
H	-4.38558500	-0.34923800	-0.00030900

31
ace-H-SiMe2-F opt

C	2.56565300	2.10194400	-0.00002200
C	2.71530200	0.74301300	0.00002500
C	1.56545000	-0.06707400	-0.00000200
C	0.25421000	0.42889500	-0.00000200
C	0.13448200	1.84238400	-0.00002000
C	1.25441200	2.63790900	-0.00004400
H	3.41884200	2.76898400	-0.00001100
C	1.88197700	-1.43843600	-0.00001500
C	-0.80742700	-0.52942000	0.00004100
H	1.13352700	3.71410100	-0.00006900
C	-0.47309900	-1.87012100	0.00006600
C	0.86284600	-2.34700200	0.00003900
H	-1.27129700	-2.60397800	0.00012300
H	1.04992800	-3.41386600	0.00004300
C	3.38620400	-1.59667600	-0.00018000
H	3.72006000	-2.15465200	0.87582200
H	3.71981900	-2.15398900	-0.87670400
C	3.94217700	-0.14219900	0.00022800
H	4.56257000	0.05005400	0.87678600
H	4.56326600	0.05037200	-0.87575800
C	-3.10262500	0.86097200	1.54146500
H	-2.57701000	1.81348500	1.62696300
H	-4.17510600	1.06398900	1.54248600
H	-2.86371900	0.26827000	2.42575800
C	-3.10235500	0.86069400	-1.54169400

H	-4.17484800	1.06359100	-1.54322300
H	-2.57676700	1.81322900	-1.62713700
H	-2.86297000	0.26788300	-2.42579200
Si	-2.60959700	-0.05338700	0.00001300
F	-3.41674700	-1.44622900	0.00013500
H	-0.84444700	2.30670400	0.00000500

42

ace-OPh-SiMe2-F 12a opt

C	1.23051600	-2.93561000	0.38129600
C	2.38727500	-2.24106000	0.16995300
C	2.31810100	-0.84039500	0.05177400
C	1.13585700	-0.09351300	0.12944000
C	-0.03590900	-0.85937100	0.35660800
C	0.00893100	-2.22537600	0.48029700
H	1.22184700	-4.01360800	0.48339500
C	3.58232600	-0.26117300	-0.16076700
C	1.21697900	1.32493400	-0.02212400
H	-0.91048000	-2.76695600	0.66098400
C	2.47431000	1.86356900	-0.22974700
C	3.66415200	1.09426000	-0.29891900
H	2.56431100	2.93634400	-0.34916900
H	4.61147800	1.59349900	-0.46235500
C	4.62604700	-1.35520600	-0.18646200
H	5.16645600	-1.35842900	-1.13391000
H	5.36707400	-1.20479200	0.59979700
C	3.82898200	-2.67732500	0.02437000
H	3.95192600	-3.35492400	-0.82159100
H	4.17093300	-3.21221500	0.91131600
O	-1.19277900	-0.13102800	0.50838500
C	-1.33534400	2.34994500	-1.49963900
H	-1.89732200	1.41622200	-1.50277400
H	-2.04990600	3.17570300	-1.51971800
H	-0.73918600	2.39900700	-2.41236200
C	-2.40797500	-0.67930100	0.16864600
C	-3.44884900	-0.53814900	1.07177300
C	-2.60805700	-1.28253600	-1.06656500
C	-4.71116900	-1.00599800	0.73168800
H	-3.25505800	-0.06595000	2.02574600
C	-3.87135800	-1.75111200	-1.39103700
H	-1.77832500	-1.37828000	-1.75528300
C	-4.92576600	-1.61475500	-0.49601300
H	-5.52721300	-0.89627900	1.43383500
H	-4.03410700	-2.21900100	-2.35318600
H	-5.90966000	-1.98037900	-0.75710900
C	-1.17834800	2.61510600	1.59686200
H	-1.25766700	3.65856700	1.90617900
H	-2.18462300	2.21203400	1.47851900
H	-0.68233800	2.05553400	2.39007400
Si	-0.23679600	2.51175400	-0.00842700
F	0.48056000	3.95177600	-0.18223200

38

naph-OPh-SiMe2-F 13a opt

C	0.50663500	3.46357900	-0.22604500
C	1.84071800	3.21647900	-0.09362900
C	2.32087100	1.88560300	-0.01725800
C	1.41456400	0.79477700	-0.07178600
C	0.03660500	1.11211400	-0.21681900
C	-0.41591100	2.39953500	-0.29332300
H	0.14054100	4.47994000	-0.28727200
C	3.70391900	1.61813000	0.11601900
C	1.88677500	-0.55221500	0.01610500
H	-1.47206300	2.59860100	-0.41093600
C	3.24577000	-0.74248600	0.14543200
C	4.15606300	0.33459500	0.19216600
H	3.63712100	-1.74899900	0.21575400
H	5.21481200	0.13261500	0.29212300
O	-0.79135700	0.02293900	-0.32003600
C	-0.18662600	-2.35789100	1.54762300
H	-1.09056800	-1.75079300	1.55880500
H	-0.48083000	-3.40772000	1.61402100
H	0.40297700	-2.12143400	2.43502100
C	-2.14638500	0.16558900	-0.10897300
C	-3.00686600	-0.14303000	-1.14821100
C	-2.62687100	0.54575700	1.13646100
C	-4.37767200	-0.07417100	-0.93379600
H	-2.59374200	-0.43223300	-2.10547700
C	-3.99642700	0.61668400	1.33652100
H	-1.92616100	0.78208000	1.92739900
C	-4.87401100	0.30637200	0.30415000
H	-5.05694500	-0.31673400	-1.74035600
H	-4.37988200	0.90928900	2.30513600
H	-5.94200800	0.36011500	0.46711200
C	-0.07986100	-2.48256600	-1.57202700
H	0.18084000	-3.48311700	-1.92006200
H	-1.15830700	-2.44209700	-1.41397700
H	0.17358900	-1.76734600	-2.35498000
Si	0.83418700	-2.11594200	0.01122400
F	1.96108000	-3.27532200	0.11507200
H	4.39198800	2.45414800	0.15500000
H	2.55341700	4.03014200	-0.04546000

42

ace-SPh-SiMe2-F 12b opt

C	-1.97628100	-2.51074500	-1.31746700
C	-2.79999300	-1.79782600	-0.49228400
C	-2.36766800	-0.55237400	0.00172700
C	-1.12530100	0.04006500	-0.29697800
C	-0.27958800	-0.75466000	-1.12974800
C	-0.70930500	-1.96951000	-1.61977400
H	-2.26396100	-3.47012600	-1.72829300
C	-3.33446000	0.03062800	0.84459400
C	-0.86362600	1.33468500	0.26270000
H	-0.03108300	-2.53335100	-2.24780000

C	-1.83709600	1.87514900	1.08684200
C	-3.06459900	1.24377800	1.40274600
H	-1.66472000	2.85556800	1.51184000
H	-3.76624600	1.73926000	2.06242600
C	-4.52904900	-0.88785700	0.95125900
H	-4.68271000	-1.20428500	1.98390500
H	-5.44307400	-0.38414800	0.63498600
C	-4.18599600	-2.09281100	0.03141400
H	-4.20859600	-3.03841200	0.57430800
H	-4.89654000	-2.18344400	-0.79149400
C	2.21006200	2.09056000	0.72800700
H	2.99542500	1.87696700	0.00296600
H	2.51415600	2.95401600	1.32237800
H	2.13397600	1.22898000	1.39269200
C	2.32706500	-1.04721600	-0.25857800
C	3.71189200	-0.98328800	-0.40329000
C	1.77770500	-1.66995400	0.85624800
C	4.53759100	-1.53038400	0.56472900
H	4.13901600	-0.50232400	-1.27541400
C	2.61484800	-2.21877800	1.81901600
H	0.70414300	-1.72305200	0.97917000
C	3.99315200	-2.15065100	1.68210200
H	5.61158500	-1.47395900	0.44277200
H	2.17924300	-2.69989700	2.68538300
H	4.63869900	-2.57846700	2.43730600
C	0.69017700	2.95784100	-1.90661400
H	0.33322400	3.98222800	-2.02668800
H	1.71352700	2.90230400	-2.27873700
H	0.07329800	2.30915800	-2.52926400
Si	0.58657800	2.48800800	-0.10128700
F	0.11611300	3.85269700	0.63985900
S	1.37039700	-0.25020800	-1.52081600

42

ace-SePh-SiMe2-F 12c opt

Se	-1.38294100	-0.17445200	-1.45505200
C	1.07123300	1.33806200	0.35020400
C	2.08032300	1.84470600	1.15354500
C	3.29564900	1.17864200	1.43771300
C	3.51465700	-0.04013600	0.87150100
C	2.51121200	-0.59342800	0.05129900
C	2.89836900	-1.85309600	-0.44551500
C	2.04190800	-2.54371100	-1.25432000
C	0.78748800	-1.96447100	-1.53748500
C	0.40077300	-0.73691700	-1.04354600
C	1.27896000	0.03692900	-0.22284500
C	-2.32802700	-1.01113500	-0.00719800
C	-2.45400800	-2.19177500	2.07914500
C	-1.69435200	-1.66039800	1.04441000
H	0.08753400	-2.51280700	-2.15504800
H	4.87779400	-1.29083400	1.97838500
H	-1.95500900	-2.69431300	2.89814800

H	-0.61570500	-1.74865700	1.06464500
C	-3.71536300	-0.89915100	-0.02174200
C	-4.46413400	-1.43159000	1.01570500
C	-3.83634600	-2.07927500	2.07195900
H	-4.42135700	-2.49335000	2.88242400
H	1.94772300	2.82666700	1.58883400
H	4.02770900	1.65118500	2.08112100
C	4.68785300	-0.98790400	0.94771000
C	4.27902600	-2.19573500	0.06104600
H	2.29315300	-3.51317300	-1.66582900
H	5.59959600	-0.51163200	0.58472800
H	4.26585100	-3.12775500	0.62769100
H	4.97275100	-2.33986900	-0.76846800
H	-4.21161600	-0.39348100	-0.84212900
H	-5.54254700	-1.33902900	0.99767500
Si	-0.35365400	2.54972100	0.06492500
C	-0.51403800	3.07480700	-1.72212100
H	0.06863700	2.43535500	-2.38609300
H	-1.55049300	3.05185200	-2.05907400
H	-0.13898500	4.09508800	-1.82160200
C	-1.94600200	2.18472900	0.96896000
H	-1.90428000	1.24077100	1.51331300
H	-2.12670600	2.98558900	1.68820400
H	-2.79649300	2.13403300	0.28840300
F	0.19139000	3.88497100	0.80854800

38

naph-SePh-SiMe2-F 13c opt

Se	-0.96884500	0.00710100	-1.45937100
C	1.65946300	0.65190400	0.48815200
C	2.64553000	0.86815500	1.42942700
C	3.52360900	-0.13999500	1.87568500
C	3.44142700	-1.38536900	1.33780500
C	2.45009800	-1.68411300	0.37286300
C	2.39351800	-2.99124000	-0.16492300
C	1.44247300	-3.33899300	-1.07665300
C	0.48110200	-2.38610300	-1.45522300
C	0.50400600	-1.11031500	-0.94898500
C	1.51977500	-0.68009100	-0.03768100
C	-2.19146000	-0.40700600	-0.03850700
C	-2.79431500	-1.39585500	2.06360700
C	-1.85752000	-1.19121500	1.05774100
H	-0.30911400	-2.66647000	-2.13899800
H	-2.52823400	-2.00520700	2.91800600
H	-0.87500100	-1.63798600	1.13743400
C	-3.45782000	0.16432200	-0.12739700
C	-4.38421400	-0.04488000	0.88160400
C	-4.05563800	-0.82530500	1.98270500
H	-4.77944100	-0.98708200	2.77036800
H	2.77818800	1.86176100	1.83578600
H	4.27222500	0.09299900	2.62189400
H	1.40355400	-4.33797000	-1.49003500

H	-3.72055900	0.77466300	-0.98381400
H	-5.36663800	0.40324000	0.80468400
Si	0.79406400	2.25758600	-0.04165700
C	1.01288800	2.63494700	-1.85958300
H	1.33944900	1.76146900	-2.42494800
H	0.08664800	2.99659200	-2.30721200
H	1.77391400	3.41008200	-1.96564600
C	-0.88634500	2.59100500	0.70289100
H	-1.22665400	1.76505900	1.32859200
H	-0.82081900	3.48673100	1.32288400
H	-1.64461800	2.75544200	-0.06332800
F	1.72316100	3.37613400	0.68091100
H	3.12866100	-3.71340000	0.17043600
H	4.12437700	-2.17188200	1.63633200

51

ace-TeMes-SiMe2-F 12d opt

C	-0.04645800	1.47694600	-1.62560800
C	0.47964300	0.39859900	-0.94513700
C	1.73606000	0.51356100	-0.26868500
C	2.30317100	1.80654000	-0.31241700
C	1.74754200	2.90693000	-0.99502100
C	0.57292100	2.74403600	-1.66927300
H	-0.99930700	1.36111500	-2.12778400
C	2.47712300	-0.48238600	0.45848300
C	3.48918100	2.17602100	0.35524500
H	0.10382100	3.55372600	-2.21407400
C	4.14920100	1.23829100	1.08909800
C	3.63304700	-0.07730800	1.10817000
H	5.05786500	1.46776000	1.63168200
H	4.20786600	-0.81777300	1.65051500
C	3.77904700	3.63740800	0.11012400
H	4.75931100	3.77232500	-0.34839900
H	3.79021100	4.19097100	1.05015100
C	2.63356100	4.11716100	-0.82145400
H	2.08071200	4.95240100	-0.39006100
H	3.01667600	4.45507400	-1.78551900
Te	-0.83469500	-1.26221500	-0.79518000
C	-2.44846400	-0.17830900	0.07075400
C	-2.26031400	0.63053200	1.19938900
C	-3.72444600	-0.31805900	-0.49888200
C	-3.36015100	1.31226300	1.71910600
C	-4.78905100	0.37616300	0.06306600
C	-4.62660700	1.20445800	1.16704600
H	-3.21574800	1.93823400	2.59322200
H	-5.77544800	0.26477900	-0.37463900
Si	2.24219800	-2.34996100	0.53734500
C	1.92820000	-3.11994100	-1.13742300
H	1.70879600	-2.38062000	-1.91002100
H	2.84103300	-3.63997200	-1.43543500
H	1.11484900	-3.84554800	-1.11673200
C	1.20572800	-2.97620400	1.94991800

H	0.15792100	-2.69544200	1.88361700
H	1.27388700	-4.06575500	1.99020600
H	1.61513100	-2.58396000	2.88359300
C	-0.93066800	0.81022900	1.87848200
H	-0.35848400	-0.11875400	1.90521800
H	-0.31794300	1.54622900	1.35232200
H	-1.07371900	1.15985900	2.89990000
C	-3.98570300	-1.19969000	-1.69178300
H	-3.37518900	-0.90014900	-2.54523700
H	-3.74576800	-2.24388300	-1.48078800
H	-5.03470400	-1.15009000	-1.97886000
C	-5.79241300	1.96679000	1.73237200
H	-6.01908300	2.83819500	1.11418700
H	-6.68874800	1.34683900	1.76501600
H	-5.58136600	2.31948100	2.74110700
F	3.71665100	-2.88606600	0.93999000

31

ace 5-Br 6-SiFMe2 12e opt

C	-1.27292400	-2.37616900	0.07629000
C	-0.29273300	-1.41663000	-0.00528600
C	-0.59274100	-0.02387700	-0.02929500
C	-1.97944600	0.24348600	0.00477400
C	-2.99253500	-0.73135500	0.08753900
C	-2.64230300	-2.04922500	0.13175900
H	-0.97754100	-3.41624200	0.08859400
C	0.29094500	1.11309400	-0.09232300
C	-2.53240100	1.54025600	-0.05054800
H	-3.37879500	-2.84002900	0.19608800
C	-1.69405500	2.60758400	-0.14074900
C	-0.30057200	2.36418600	-0.15203100
H	-2.06241300	3.62441400	-0.19554900
H	0.33430700	3.23845800	-0.20662800
C	-4.03882400	1.45437100	-0.00633500
H	-4.43782300	2.01550600	0.83942900
H	-4.47619200	1.88588300	-0.90767300
C	-4.34645400	-0.06285500	0.10953900
H	-4.97108200	-0.41306100	-0.71299700
H	-4.87863400	-0.29473100	1.03314500
Si	2.16978900	1.18975300	0.05156500
Br	1.48493000	-2.06597200	-0.13846000
C	2.75775400	2.95497600	-0.08005000
H	2.40249900	3.58117200	0.73937400
H	3.84917100	2.93656800	-0.03155600
H	2.47835700	3.42026000	-1.02616700
C	2.79971600	0.48675800	1.66041700
H	3.73522800	-0.05058300	1.49207300
H	2.99894900	1.29972400	2.36167100
H	2.09749900	-0.20317600	2.12482700
F	2.88403400	0.47119000	-1.19473700

31

ace 5-I 6-SiFMe2 12f opt

C	-0.99059800	-2.38163300	0.24478500
C	-0.22270900	-1.25212200	0.07160100
C	-0.80849200	0.04704800	-0.02329200
C	-2.22258600	0.02156900	0.00359000
C	-3.01202100	-1.13186000	0.17679400
C	-2.39686200	-2.34070100	0.31693200
H	-0.49756000	-3.34160700	0.31004500
C	-0.18945800	1.34499700	-0.15119700
C	-3.03727300	1.16289700	-0.15928600
H	-2.95266300	-3.25928200	0.45608900
C	-2.44479400	2.37219500	-0.34569100
C	-1.03238000	2.43172700	-0.31853600
H	-3.01943100	3.27832900	-0.49147600
H	-0.59742500	3.41630500	-0.42516500
Si	1.60174300	1.86544000	0.12880000
C	-4.47533600	-0.76096200	0.17069100
H	-4.93556300	-0.99654300	1.13150300
H	-5.02248100	-1.32009500	-0.58891800
C	-4.49222400	0.76524200	-0.10776700
H	-4.98428600	0.99383000	-1.05429700
H	-5.02460900	1.31676300	0.66774600
I	1.82561900	-1.62712900	-0.17308600
C	1.76652300	3.72170800	0.05135100
H	2.81966000	3.95809600	0.22159400
H	1.49383800	4.12725500	-0.92378800
H	1.18457000	4.23066300	0.82059800
C	2.23704600	1.29938300	1.78888500
H	2.14037400	2.11595700	2.50788700
H	3.29386600	1.03461100	1.71816300
H	1.70120900	0.43575100	2.17904500
F	2.57169900	1.37052100	-1.05242400

27

naph 1-Br 8-SiFMe2 13e opt

C	-2.39708500	-1.52673100	0.22750400
C	-1.16710300	-0.95954500	0.02681800
C	-0.96732100	0.45161500	-0.03288400
C	-2.16486300	1.23020200	0.03426600
C	-3.42591400	0.62581600	0.24086300
C	-3.54281500	-0.72629700	0.35794000
H	-2.48359500	-2.60352800	0.26149500
C	0.29833000	1.13201800	-0.16340900
C	-2.10758200	2.63680800	-0.12427500
H	-4.50534900	-1.19238000	0.52010800
C	-0.92049200	3.26130400	-0.33301200
C	0.26710200	2.50149000	-0.32584600
H	-0.87221400	4.33299000	-0.47416000
H	1.19444600	3.04654000	-0.43516100
Si	2.05426800	0.48901900	0.13057900
Br	0.24387700	-2.19041600	-0.28090600
C	3.26460400	1.90818300	0.13830500
H	3.05552500	2.63768800	0.92166200

H	4.25245400	1.48435100	0.33475200
H	3.31665900	2.42474800	-0.82089900
C	2.20074200	-0.40092000	1.76319100
H	2.86299800	-1.26292900	1.66151800
H	2.63312500	0.27155200	2.50689600
H	1.24347300	-0.75831800	2.13932100
F	2.59047800	-0.42520100	-1.07582800
H	-4.29792400	1.26539300	0.29790000
H	-3.03495900	3.19533600	-0.08415800

47

(C6F5)3B-NC(C6H4F) BCF adduct 4 opt

B	-0.32607200	-0.05458200	-0.09575100
C	-0.72460700	-1.51690800	-0.69141000
C	-1.58445100	-1.76571200	-1.74996100
C	-0.15693400	-2.64884100	-0.11962400
C	-1.88137400	-3.04598200	-2.19636400
C	-0.41814300	-3.93830000	-0.53544700
C	-1.29671300	-4.13830800	-1.58669500
C	-0.92839400	1.22847000	-0.89189800
C	-2.28716700	1.48230600	-0.75280100
C	-0.23793800	2.14672000	-1.66314000
C	-2.92791200	2.56379200	-1.32410400
C	-0.83883600	3.24767000	-2.25401300
C	-2.19241900	3.45914200	-2.08299700
C	-0.61357000	0.14107700	1.50142400
C	-1.48131000	-0.62720900	2.26339500
C	-0.00585000	1.19017200	2.17794800
C	-1.72256600	-0.38368600	3.60640400
C	-0.21434500	1.46563500	3.51666100
C	-1.08376700	0.66744400	4.23799100
F	1.08187000	2.01743700	-1.88961900
F	-0.12063000	4.09773500	-2.98082400
F	-2.78248300	4.50530100	-2.64164400
F	-4.23107700	2.75039800	-1.16114600
F	-3.03777400	0.62888000	-0.05018200
F	0.69828800	-2.50909500	0.90719100
F	0.15905900	-4.97890500	0.05522400
F	-1.56496100	-5.36505700	-2.00711100
F	-2.18098000	-0.77209000	-2.41090400
F	-2.71747000	-3.22727500	-3.21088500
F	-2.14332100	-1.65254500	1.72554800
F	-2.56156000	-1.14999000	4.29193900
F	-1.30237400	0.90894900	5.52198500
F	0.40246800	2.48209000	4.10941900
F	0.83528500	2.00546400	1.52068000
N	1.24646700	-0.02494100	-0.21964100
C	2.38387600	-0.01696500	-0.26447700
C	3.80257900	0.03306800	-0.33575900
C	4.40764000	1.17556300	-0.86434700
C	4.55811000	-1.04737400	0.12314200
C	5.78500400	1.23618600	-0.93523800

H	3.79602400	1.99813800	-1.21032900
C	5.93538200	-0.98489400	0.05190800
H	4.06469400	-1.91989200	0.53015400
C	6.51951900	0.15518500	-0.47610600
H	6.29685600	2.09952000	-1.33638400
H	6.56140400	-1.79655300	0.39465900
F	7.84468400	0.21389800	-0.54509400

34

B(C6F5)3 opt

B	0.00000000	0.00000000	0.02209400
C	-0.00450000	1.56471800	0.01755300
C	0.96493800	2.31240400	0.68351000
C	-0.97206900	2.30146200	-0.66279700
C	0.97736700	3.69367400	0.68892900
C	-0.97982900	3.68258100	-0.69689700
C	0.00000000	4.38104300	-0.01153500
C	1.35733600	-0.77846200	0.01755300
C	1.52013100	-1.99186300	0.68351000
C	2.47915900	-0.30889500	-0.66279700
C	2.71013200	-2.69326100	0.68892900
C	3.67912300	-0.99273400	-0.69689700
C	3.79409400	-2.19052100	-0.01153500
C	-1.35283600	-0.78625600	0.01755300
C	-2.48507000	-0.32054100	0.68351000
C	-1.50709000	-1.99256700	-0.66279700
C	-3.68749900	-1.00041200	0.68892900
C	-2.69929500	-2.68984700	-0.69689700
C	-3.79409400	-2.19052100	-0.01153500
F	2.42376000	0.83448100	-1.34287400
F	4.71568400	-0.51568600	-1.36971700
F	4.93545500	-2.85123400	-0.02485600
F	2.82411100	-3.83637300	1.34815100
F	0.51100500	-2.52001500	1.37269600
F	-1.93456200	1.68179700	-1.34287400
F	-1.91124400	4.34174500	-1.36971700
F	0.00151300	5.69984700	-0.02485600
F	1.92689400	1.70255000	1.37269600
F	1.91034100	4.36393900	1.34815100
F	-2.43789900	0.81746400	1.37269600
F	-4.73445300	-0.52756500	1.34815100
F	-4.93696800	-2.84861300	-0.02485600
F	-2.80443900	-3.82605900	-1.36971700
F	-0.48919800	-2.51627800	-1.34287400

35

(C6F5)3B-F opt in DCM

B	-0.00336400	0.00218600	0.77516900
C	-1.53406400	0.38224000	0.29556600
C	-2.31736200	-0.30333200	-0.61644900
C	-2.15049500	1.48606300	0.86939700
C	-3.61988100	0.05504400	-0.93083600
C	-3.44479300	1.88022400	0.58809500

C	-4.19030300	1.15306200	-0.32143200
C	0.43382000	-1.51340900	0.29793600
C	1.40266800	-1.84540900	-0.63289600
C	-0.19877000	-2.60078700	0.88509000
C	1.74523800	-3.15115300	-0.95036200
C	0.10982300	-3.91786800	0.60164400
C	1.09728500	-4.19653900	-0.32531300
C	1.09549800	1.13581900	0.30032900
C	0.90150500	2.15130300	-0.61964100
C	2.35595900	1.11753200	0.88161600
C	1.86764900	3.09457800	-0.93562300
C	3.34899700	2.03624500	0.59898800
C	3.10044000	3.03961000	-0.31913700
F	-1.19814300	-2.40452500	1.75675200
F	-0.53693800	-4.92025200	1.19814900
F	1.41179400	-5.45631800	-0.61565700
F	2.69276100	-3.40678200	-1.85370400
F	2.07306300	-0.89760100	-1.30779800
F	-1.47194500	2.26312700	1.72525400
F	-3.97929600	2.95479500	1.16979800
F	-5.43914800	1.51143900	-0.60778700
F	-1.84448000	-1.37377200	-1.27468200
F	-4.32636500	-0.65076600	-1.81518600
F	-0.25841000	2.27562400	-1.28462700
F	1.61791500	4.05382500	-1.82832900
F	4.04032900	3.93606600	-0.60757600
F	4.54311400	1.96279300	1.18856900
F	2.68202700	0.14597400	1.74579600
F	-0.00655000	0.00300900	2.20239900

22

Et3B opt

B	-0.01197100	-0.37202000	0.20879100
C	-0.03097900	1.10496500	0.74916300
H	-0.95488100	1.33506500	1.28524300
H	0.80191300	1.32411100	1.42120700
C	0.06863100	2.03192300	-0.48220300
H	0.03807700	3.08200900	-0.19009000
H	1.00050900	1.86758300	-1.02650400
H	-0.75454300	1.85769800	-1.17852800
C	1.33492300	-1.18296300	0.11509900
H	1.25145300	-2.01620000	-0.58904200
H	1.40387300	-1.66101200	1.10644000
C	2.62222200	-0.39575900	-0.13968800
H	2.72340800	0.44068700	0.55379200
H	3.50851000	-1.02247500	-0.03211900
H	2.63677900	0.01528900	-1.15062400
C	-1.32993800	-1.05284000	-0.32015900
H	-1.20955700	-1.06830800	-1.41475600
H	-1.31952300	-2.11528600	-0.04933100
C	-2.66815100	-0.41387300	0.04341400
H	-2.70923200	0.62882900	-0.27799800

H	-3.50872800	-0.93348100	-0.41857400
H	-2.82845700	-0.42312400	1.12316700

23
Et3B-F opt

B	-0.08072900	-0.23815400	0.48089600
C	-0.18017900	1.38649400	0.70765700
H	-1.23545200	1.68379800	0.77430500
H	0.25319700	1.61454700	1.69138000
C	0.51619900	2.24178400	-0.35169200
H	0.38351200	3.31995000	-0.20071100
H	1.59291200	2.04798300	-0.35979400
H	0.14369100	2.00556200	-1.35434900
C	1.48270200	-0.77462500	0.49457600
H	1.47300600	-1.78338000	0.92873900
H	2.08349400	-0.16323700	1.18405600
C	2.19713300	-0.84315200	-0.85950100
H	2.17768700	0.12121500	-1.37531200
H	3.24738000	-1.14945000	-0.77926300
H	1.70088300	-1.55783500	-1.52182700
C	-0.86732100	-0.73380400	-0.87656200
H	-0.38530400	-0.34857800	-1.78509500
H	-0.78623600	-1.82893700	-0.94027100
C	-2.34684600	-0.34465300	-0.89050000
H	-2.46187500	0.74120900	-0.96278600
H	-2.91113400	-0.78805200	-1.72001000
H	-2.82270400	-0.65385300	0.04417600
F	-0.76217000	-0.85138200	1.62538000

13
4-Fluorobenzonitrile opt

C	-0.35893900	1.20935200	0.00000000
C	1.02451900	1.21288900	0.00000000
C	1.68843100	0.00000000	0.00000000
C	1.02451900	-1.21288900	0.00000000
C	-0.35893900	-1.20935200	0.00000000
C	-1.05295500	0.00000000	0.00000000
H	-0.90765800	2.14111100	0.00000000
H	1.59187000	2.13308000	0.00000000
H	1.59187000	-2.13308000	0.00000000
H	-0.90765800	-2.14111100	0.00000000
C	-2.48832500	0.00000000	0.00000000
N	-3.63582800	0.00000000	0.00000000
F	3.02361100	0.00000000	0.00000000

3. References

[S1] N. Lühmann, *PhD thesis*, University of Oldenburg **2011**.

[S2] J. Beckmann, T. G. Do, S. Grabowsky, E. Hupf, E. Lork, S. Mebs, *Z. Anorg. Allg. Chem.* **2013**, 2233.

[S3] N. Lühmann, H. Hirao, S. Shaik, T. Müller, *Organometallics* **2011**, 30, 4087.

[S4] J. C. W. Chien, W. M. Tsai, M. D. Rausch, *J. Am. Chem. Soc.* **1991**, 113, 8570.

[S5] B. Su, J. F. Hartwig, *Angew. Chem. Int. Ed.* **2018**, *57*, 10163.

[S6] N. Kordts, S. Künzler, S. Rathjen, T. Sieling, H. Großekappenberg, M. Schmidtmann, T. Müller, *Chem. Eur. J.* **2017**, *23*, 10068.

[S7] A. Schäfer, M. Reißmann, A. Schäfer, W. Saak, D. Haase, T. Müller, *Angew. Chem. Int. Ed.* **2011**, *50*, 12636.

[S8] N. Tanaka, T. Kasai, *Bull. Chem. Soc. Jpn.* **1981**, *54*, 3020.

[S9] A. Schäfer, A. Schäfer, T. Müller, *Dalton Trans.* **2010**, *39*, 9296.

[S10] G. M. Sheldrick, University of Göttingen (Germany) **2014**.

[S11] G. M. Shedrick, *Acta Crystallogr. Sect. C* **2015**, *71*, 3.

[S12] M. J. T. Frisch, G. W.; Cheeseman, J. R.; Scalmani, G.; Caricato, M.; Hratchian, H. P.; Li, X.; Barone, V.; Bloino, J.; Zheng, G.; Vreven, T.; Montgomery, J. A.; Petersson, G., G. E. S. A.; Scuseria, H. B.; Nakatsuji, H.; Izmaylov, A. F.; Martin, R. L.;, J. L. P. Sonnenberg, J. E.; Heyd, J. J.; Brothers, E.; Ogliaro, F.; Bearpark, M.; Robb,, B. K. M. A.; Mennucci, K. N.; Staroverov, V. N.; Kobayashi, R.; Normand, J.;, A. G. Rendell, R.; Zakrzewski, V. G.; Hada, M.; Ehara, M.; Toyota, K.; Fukuda, and J. I. R.; Hasegawa, M.;Nakajima, T.; Honda, Y.; Kitao, O.; Nakai, H., *Gaussian 09*.

[S13] Y. Zhao, N. E. Schultz and D. G. Truhlar, *J. Chem. Theory Comput.* **2006**, *2*, 364.

[S14] (a) Krishnan, R.; Blinkley, J. S.; Seeger, R.; Pople, J.A. *J. Chem. Phys.* **1980**, *72*, 650; (b) McLean, A. D.; Chandler, G. S*. J. Chem. Phys.* **1980**, *72*, 5639; (c) Curtiss, L. A.; McGrath, M. P.; Blandeau, J-P.; Davis, N. E.; Binning, R. C.; Radom, Jr. L. *J.*

Chem. Phys. **1995**, *103*, 6104; (d) Weigend, F.; Ahlrichs, R. *Phys. Chem. Chem. Phys.* **2005**, 3297.
